# Skeletal restructuring of oxetanes through photoinduced oxygen deletion

**DOI:** 10.1038/s41467-026-76238-4

**Published:** 2026-07-30

**Authors:** Ying-Qi Zhang, Shuo-Han Li, Ming Joo Koh

**Affiliations:** Department of Chemistry, National University of Singapore, 4 Science Drive 2, Singapore, 117544 Republic of Singapore

**Keywords:** Synthetic chemistry methodology, Photochemistry

## Abstract

Deleting an atom from a heterocycle to reduce ring size or remodel the skeleton represents a unique approach to navigate untapped regions of the chemical space. However, compared with well-established nitrogen-deletion manifolds, the precise recognition and excision of an oxygen atom from a functionalized cyclic ether remains challenging. Here we report a catalyst-free photoinduced oxygen deletion strategy that achieves highly chemo- and regioselective restructuring of oxetanes. The reaction involves a deoxygenative ring deconstruction step to generate an acyclic diiodide intermediate using iodoform under photoirradiation, followed by intramolecular reductive coupling or cyclization/functionalization. This method enables efficient ring contraction or skeletal remodeling of oxetanes, granting streamlined access to families of cyclopropane and benzoheterocycle frameworks in a straightforward fashion. Excellent functional group tolerance is demonstrated through late-stage functionalization and simplified syntheses of pharmaceuticals, which are expected to make a profound impact on drug discovery by reducing the synthetic burden of assembling bioactive molecules.

## Abstract (web summary)

Atom deletion offers a powerful strategy for skeletal editing and accessing new chemical space. Here the authors report a catalyst-free photoinduced strategy that excises oxygen from oxetanes, enabling chemo- and regioselective ring contraction or skeletal remodelling.

## Introduction

Oxetanes have amassed significant attention as privileged motifs in medicinal chemistry^1^. Engendered by their intrinsic properties such as low molecular weight, high polarity and rigid three-dimensionality^2^, these four-membered scaffolds are extensively used as bioisosteres of carbonyl and *gem*-dimethyl groups^3^ and have been incorporated into pharmaceutical candidates to improve biophysical and biochemical profiles^2,3^. Beyond their role as pharmacophores, oxetanes also serve as convenient and versatile feedstocks to build up molecular complexity. Rapid developments over the past two decades have contributed to libraries of functionalized oxetanes becoming commercially available for use as building blocks in mainstream organic synthesis^4^. The rich chemistry of oxetanes in the literature mostly revolves around classical ring expansions, ring openings and C-2 functionalizations in which the oxygen atom in the strained ring serves as a coordination site for Lewis or Brønsted acids, generating an activated species to trigger downstream transformations^5^. Oxetanes bearing a C-3 leaving group have also been reported to undergo site-selective reactions to install useful functionalities at the C-3 position^6^. In all these reported studies, the oxygen atom is typically retained within the cyclic or acyclic target product backbone. On the other hand, skeletal modification to the oxetane ring has not reached the same level of advancement. The lack of intellectual deployment owes largely to the inherent challenge of strain-induced ring rupture^7^ and the presumed difficulty of identifying skeletal editing^8^ disconnections to achieve the desired outcome.

**Fig. 1:**
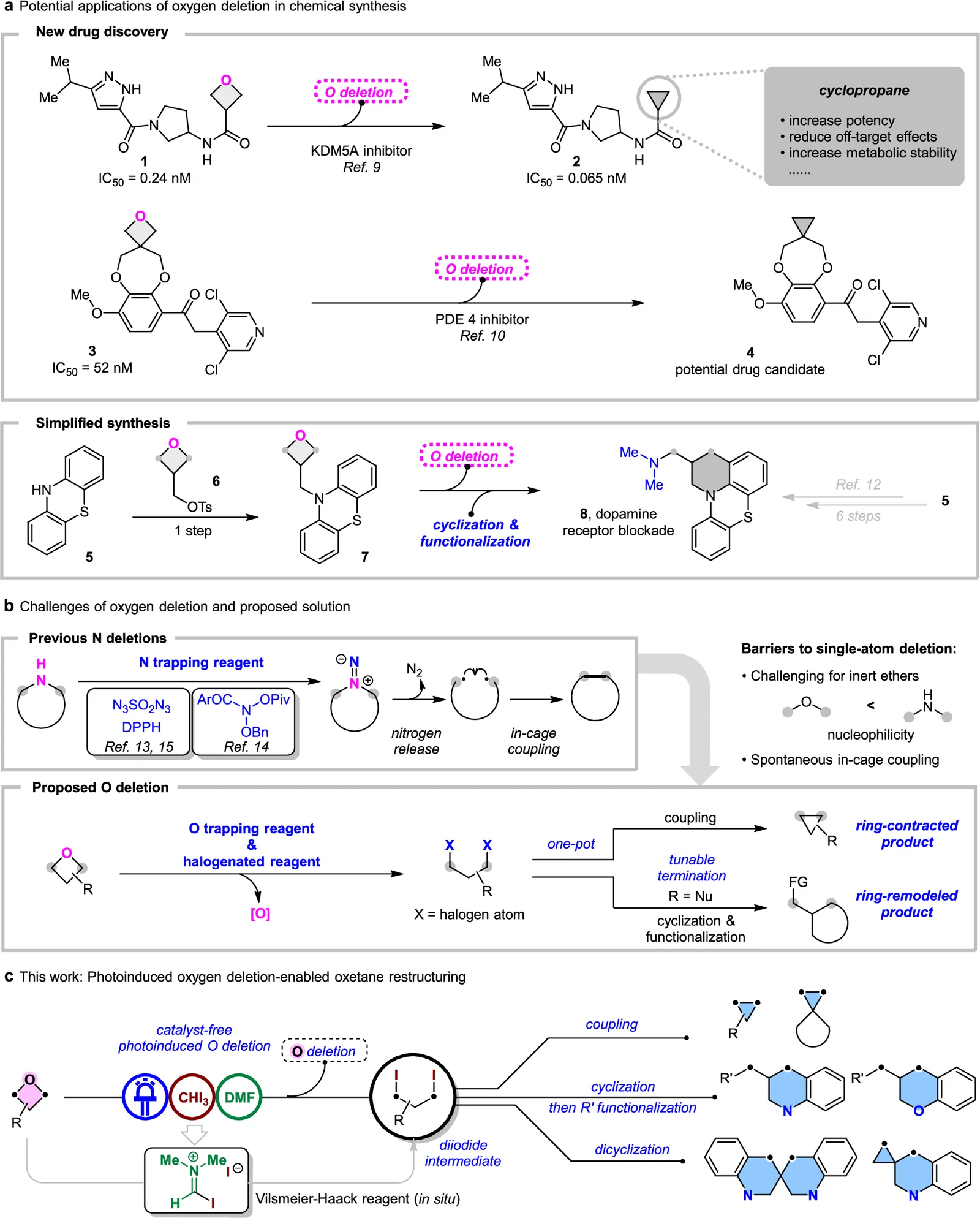
Oxygen deletion as a blueprint for oxetane restructuring. **a** Potential applications of oxygen deletion in chemical synthesis. An ideal one-step conversion of KDM5A inhibitor **1** to a more potent derivative **2** through oxygen deletion. In a similar fashion, access to **4** could be potentially achieved by oxygen-deletion of PDE 4 inhibitor **3**. Dopamine receptor blockade **8** could be rapidly assembled from readily available **5** and **6** using oxygen deletion as the key step. **b** The challenges of oxygen deletion in cyclic ethers. Conventional nitrogen deletion strategies are not applicable to O-heterocycles, which are less reactive than amines. Spontaneous in-cage coupling also limits the synthetic utility. A potential solution is the synergistic use of suitable oxygen-trapping and halogenation reagents to generate dihalide intermediates, which can be manipulated in a controlled manner to achieve ring contraction or remodeling. **c** Skeletal restructuring of oxetanes via catalyst-free photoinduced oxygen deletion involving ring cleavage into diiodide intermediates followed by various terminating recyclizations. IC_50_ half maximal inhibitory concentration, KDM5A lysine-specific demethylase 5A, PD phosphodiesterase, Ts tosyl, DPPH *O*-diphenylphosphinyl hydroxylamine, Ar aryl, Piv pivaloyl, Bn benzyl, FG functional group, DMF *N*,*N*-dimethylformamide.

From a synthetic standpoint, skeletal transformations of oxetanes represent an untapped class of reactions that are potentially enabling in chemical synthesis, offering remarkable advantages in simplifying access to prized heterocyclic scaffolds^5^. In this regard, we envisioned that the selective excision of oxygen from a functionalized oxetane coupled with a subsequent transformation could provide an unexplored yet appealing diversification platform to upgrade oxetanes into divergent categories of drug-like cyclic molecules with important applications (Fig. 1a). For example, the late-stage elimination of oxygen from the oxetane core of lysine-specific demethylase 5 A (KDM5A) inhibitor **1** (IC_50_ = 0.24 nM) would lead to a substantially more potent inhibitor **2** (IC_50_ = 0.065 nM; 3.7-fold enhancement) bearing a cyclopropane without restarting the synthesis from scratch (Fig. 1a, top)^9^. By analogy, extending this oxygen-deleting ring contraction strategy to other biologically active oxetanes such as phosphodiesterase (PDE) 4 inhibitor **3**^10^ would deliver **4**, a drug candidate that could potentially possess improved potency, pharmacokinetics and conformational stability due to the beneficial features of strained cyclopropanes in medicinal chemistry^11^.

Besides ring contractions, the deletion of oxygen in an oxetane (net cleavage of two C − O bonds) could be creatively combined with an intramolecular nucleophilic cyclization and functionalization to promote skeletal remodeling into new heterocyclic pharmacophores (Fig. 1a, bottom). For instance, the chemoselective deoxygenation of oxetane **7** bearing a strategically positioned phenothiazine motif (assembled in one step from commercially available **5** and **6**) could be coupled with sequential site-selective cyclization and amination (net formation of a C − C bond and a C − N bond) to afford dopamine receptor blockade **8** in less steps than the previously reported synthesis^12^, highlighting the utility of the proposed oxygen deletion/cyclization/functionalization regime in truncating lengthy synthetic routes.

To date, most methods for single-atom deletions focus on the excision of nitrogen atoms to facilitate ring contraction or coupling^13–15^. These transformations typically rely on the high nucleophilicity of secondary amine substrates, enabling preferential recognition by nitrogen-trapping reagents and the formation of isodiazene species. Subsequent extrusion of N_2_ generates biradical intermediates that undergo in-cage coupling to afford the corresponding [*n* − 1] ring-contracted products^16^ (Fig. 1b, top). In contrast, oxygen-atom deletions in cyclic molecules are less investigated due to several challenges. Compared with amines, cyclic ethers are chemically more inert and substantially less nucleophilic/basic^17^, thereby increasing the difficulty of selective recognition and binding of chemical reagents to effect oxygen excision. Furthermore, unlike nitrogen deletions, which could leverage the propensity of a reactive isodiazene to undergo thermodynamically favored N_2_ extrusion to drive the reaction forward, such a mechanism is untenable for oxygen deletions due to the lack of compatible reagents and pathways. Notably, the spontaneous in-cage coupling of biradicals^16^ inadvertently limits the nitrogen deletion process to ring contractions in the context of cyclic amines. We sought to design a mechanistically distinct strategy that could promote selective excision of oxygen from functionalized cyclic ethers such as oxetanes by first inducing chemoselective dihalogenative ring deconstruction to form an electrophilic dihalide intermediate (Fig. 1b, bottom). Without being isolated, this species could be subjected to reductive coupling to give the ring-contracted cyclopropane product or undergo intramolecular nucleophilic cyclization and further functionalization in the presence of suitable reagents to reconstruct a new heterocyclic skeleton. This reaction manifold offers greater versatility and tunability in the assembly of structurally diverse cyclic frameworks of interest from readily available oxetane feedstocks.

Here, we describe a photoinduced divergent editing strategy that enables the straightforward skeletal restructuring of functionalized oxetanes to cyclopropanes or benzoheterocycles^18^ via oxygen deletion (Fig. 1c). Iodoform reacts with *N*,*N*-dimethylformamide (DMF) to generate an oxophilic Vilsmeier-Haack reagent in situ under photoirradiation, which selectively targets and reacts with the oxetane to afford the key diiodide intermediate. Taking advantage of the high reactivity of alkyl iodides, subsequent transformations could be induced to deliver a broad spectrum of ring-contracted or ring-modeled products. The excellent functional group compatibility of the deoxygenative restructuring method was demonstrated through the streamlined syntheses of heterocyclic pharmaceuticals and late-stage editing of complex bioactive molecules.

## Results

**Fig. 2:**
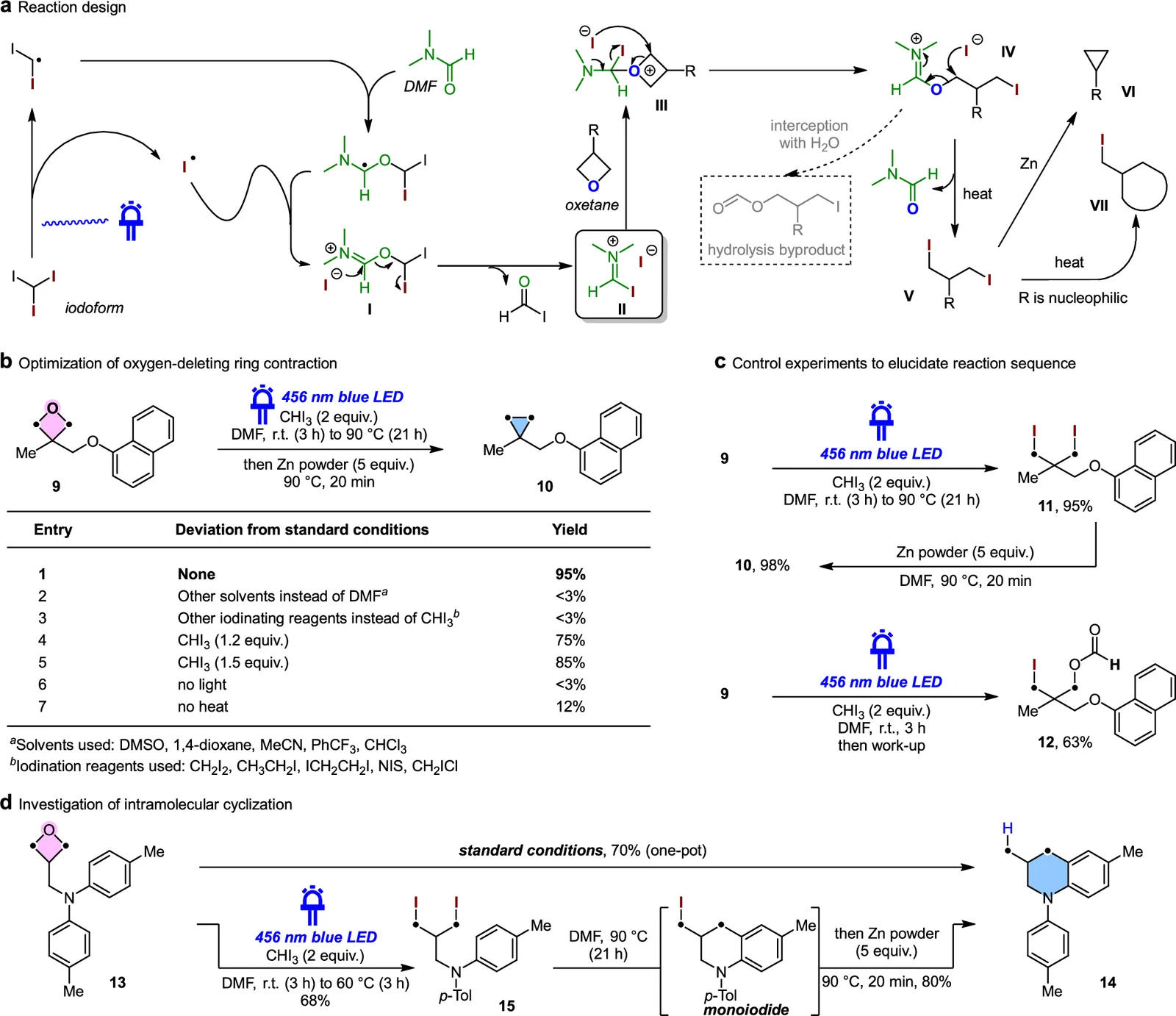
Mechanistic hypothesis and reaction development. **a** Reaction design for the ring contraction and remodeling of oxetanes via photoinduced oxygen deletion. **b** Optimization of reaction conditions for the ring contraction of **9**. Yields of **10** were determined by ^1^H NMR analysis of the crude reaction mixture. 1,3,5-Trimethoxybenzene was used as an internal standard. **c** Control experiments to elucidate the reaction sequence by probing the diiodide intermediate **11** and hydrolysis by-product **12**. **d** Investigation of the ring remodeling process using oxetane **13** tethered to a nucleophilic motif. DMSO, dimethyl sulfoxide; NIS, *N*-iodosuccinimide; *p*-Tol, *para*-tolyl.

Our initial reaction design was motivated by previous investigations on the visible light-driven formation of reactive Vilsmeier-Haack species from commercially available haloform and DMF. By leveraging the oxophilicity of these intermediates, chemoselective conversion of alcohols to haloalkanes could be achieved under mild photocatalytic^19^ or catalyst-free^20^ conditions. Given the pronounced nucleophilicity of oxetanes compared to other cyclic or acyclic ethers^17^, we postulated that an oxetane substrate could selectively intercept the Vilsmeier-Haack reagent **II** generated in situ from a mixture of iodoform (CHI_3_) and DMF via **I** under blue light irradiation (Fig. 2a). The resulting adduct **III** would be susceptible to strain-release nucleophilic ring opening by I¯ and iminium formation to give **IV**. Subsequent heat-mediated nucleophilic substitution with I¯ would furnish the requisite diiodide intermediate **V** and extrude DMF. At this stage, a dichotomy of reaction modes could be attained depending on the structure of the oxetane employed. Treating **V** with an exogenous reductant such as Zn powder could mediate intramolecular reductive coupling^21^ to deliver the ring-contracted cyclopropane **VI**. Alternatively, **V** could be trapped by a pre-installed nucleophilic motif within the substrate, triggering intramolecular cyclization to afford ring-remodeled **VII** bearing a useful alkyl iodide handle that could be subjected to further derivatization.

3,3-Disubstituted oxetane **9** was selected as the model substrate to evaluate the optimal reaction conditions (Supplementary Table 1) for oxygen-deleting ring contraction (Fig. 2b). In the presence of CHI_3_ in DMF under 456 nm blue LED irradiation, followed by heating to 90 ^o^C and addition of Zn powder, the desired cyclopropane **10** was obtained in 95% yield (entry 1). Replacing DMF and CHI_3_ with other solvents or iodination reagents failed to provide the desired product (entries 2 and 3). Reducing the loading of CHI_3_ also led to diminished yields (entries 4 and 5). Exclusion of light or heat was detrimental to the reaction (entries 6 and 7) due to low conversion of **9** to the key diiodide intermediate, which is consistent with the mechanistic hypothesis outlined in Fig. 2a. The isolation of **11** and its ensuing transformation to **10** further supports the proposed intermediacy of the diiodide species (Fig. 2c). In a separate control experiment, alkyl formate **12** was detected when the reaction was quenched with water prior to heating, providing evidence for the formation of the putative iminium species **IV** (Fig. 2a).

To test the feasibility of skeletal remodeling, oxetane **13** tethered to a nucleophilic methylaniline group was subjected to the standard reaction conditions (Fig. 2d). Gratifyingly, tetrahydroquinoline **14** was directly secured in 70% yield. To elucidate the reaction sequence, we carried out the photoinduced dihalogenative ring deconstruction step and successfully isolated diiodide **15** in 68% yield at a lower temperature (60 ^o^C). Upon heating to 90 ^o^C, **15** underwent Friedel-Crafts-type 6-*exo*-tet cyclization via reaction between the electron-rich arene and one of the iodoalkane moieties to give a monoiodide intermediate, which was further treated with Zn powder to promote reductive protonation^21^ and afford **14** in 80% yield. Overall, the net transformation of **13** to **14** underscores the viability of designing analogous remodeling processes to construct different heterocyclic cores by harnessing the power of intramolecular cyclizations with suitably positioned functional groups. It is worth mentioning that the use of bromination reagents^22^ led to an appreciable diminution in the yield of the ring-contracted product **10** and completely inhibited the formation of the ring-modeled product **14**, underscoring the importance of generating reactive diiodide intermediates (see Fig. 2a) for high efficiency (Supplementary Fig. 14).

**Fig. 3:**
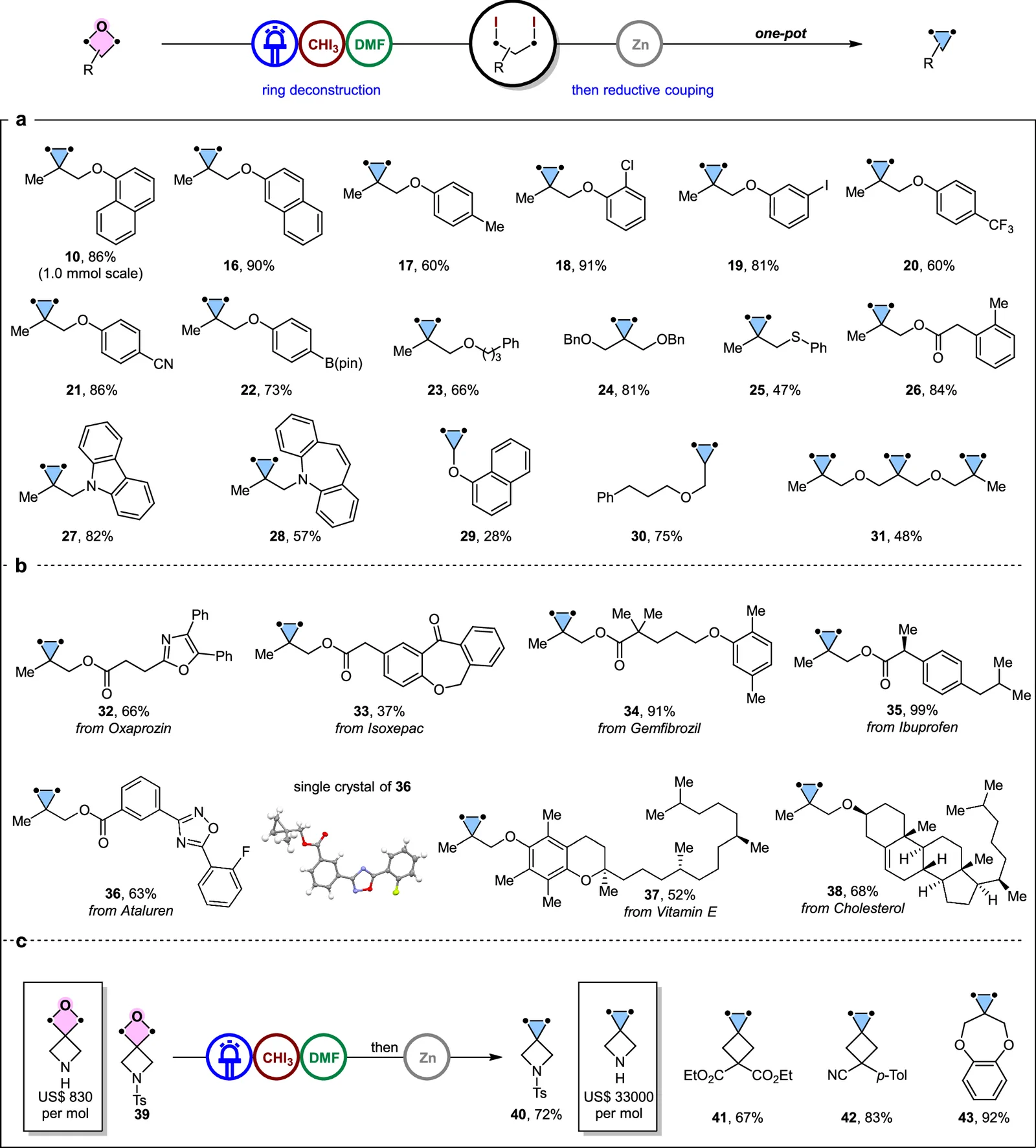
Scope of photoinduced oxygen-deleting ring contraction. Various functionalized oxetanes were compatible substrates for ring contraction via chemoselective oxygen deletion. General reaction conditions: Substrate (0.2 mmol) and CHI_3_ (0.4 mmol) in DMF (2 mL), r.t., 456 nm (20 W), 3 h, N_2_; then, LED off, 90 °C, 21 h; then, zinc powder (1.0 mmol), 90 °C, 20 min. Yields denote isolated yields. **a** Functional group tolerance with mono- and disubstituted oxetanes. **b** Oxygen deletion of drug-derived oxetanes. **c** Access to strained spirocycles by ring contraction of oxetanes. The prices shown here were obtained from Combi-Blocks. B(pin), pinacolato boronic ester.

Next, we investigated the generality of the photoinduced ring contraction method by surveying a variety of functionalized oxetanes (Fig. 3). 3,3-Disubstituted oxetanes bearing arene, haloarene, cyano, boronic ester, alkyl ether, thioether, ester, and *N*-heterocyclic moieties smoothly underwent oxygen deletion to yield the corresponding cyclopropanes **16** − **28** (Fig. 3a). Monosubstituted oxetanes (**29,** **30**) and tris-oxetane (**31**) also served as effective substrates for ring contraction. To demonstrate functional group compatibility, oxetanes tethered to complex drug molecules such as oxaprozin, isoxepac, gemfibrozil, ibuprofen, ataluren, vitamin E and cholesterol (**32** − **38**) were chemoselectively edited without interference by other O- and N-containing functionalities embedded within the substrates (Fig. 3b). The synthesis of product **38** containing an alkene motif illustrates the potential of this strategy as a complementary approach for achieving selective cyclopropane installation in polyene substrates.

We further took advantage of the oxygen deletion strategy to construct medicinally interesting strained spirocyclic compounds, which are gaining increasing attention as potential non-classical bioisosteres^23,24^ (Fig. 3c). Excision of oxygen from commercially available and inexpensive **39** afforded **40** containing a prized 5-azaspiro[2.3]hexane^25^ nucleus that would be more difficult and costly to access by other synthetic means, such as alkene cyclopropanation. A series of functionalized spirocyclic oxetanes were successfully transformed to give **41** − **43** in a similar fashion. However, substrates containing unprotected N − H amines were found to be incompatible, likely due to undesired reaction between the nucleophilic N − H group and the in situ-generated Vilsmeier-Haack reagent (Supplementary Information Section 4).

**Fig. 4:**
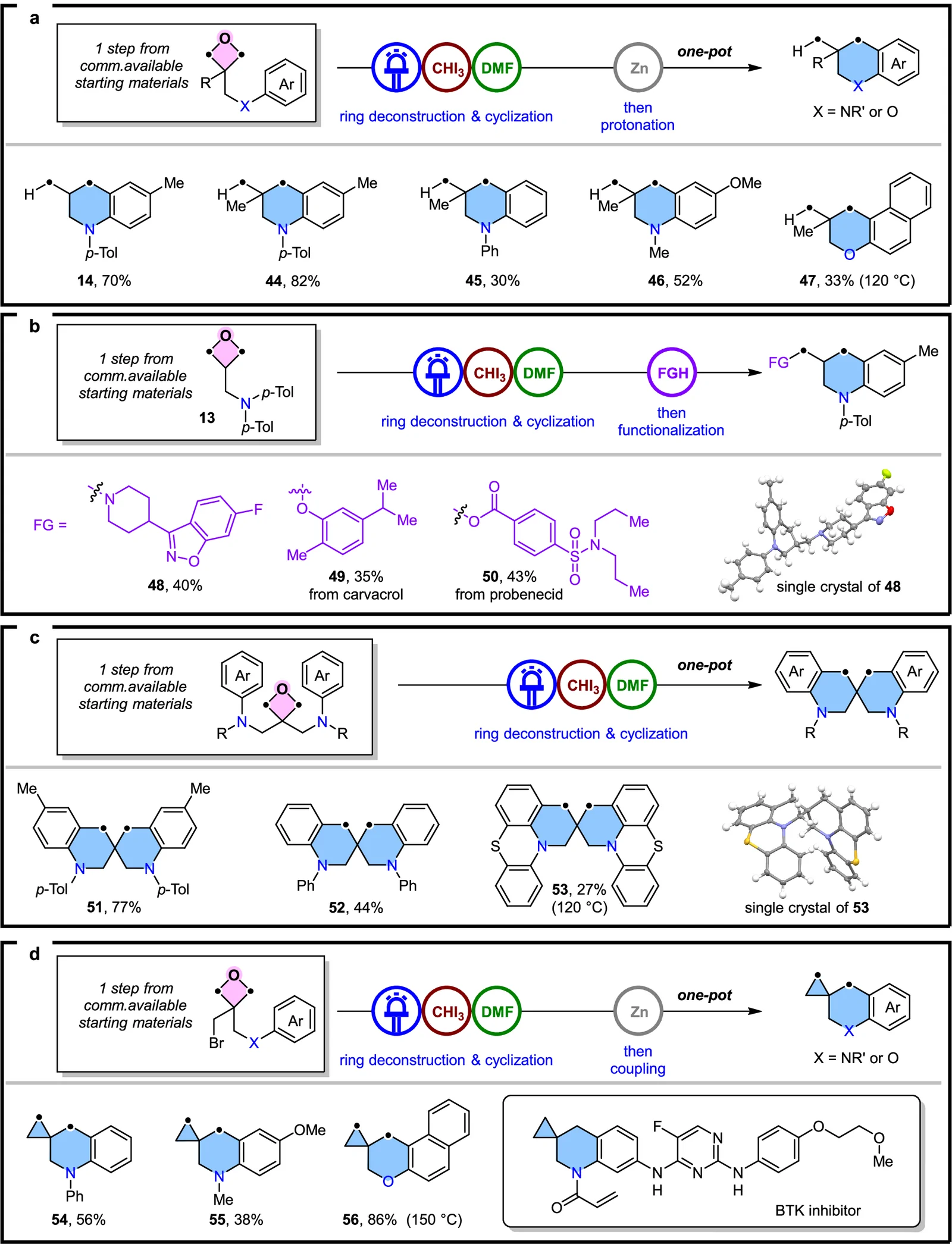
Scope of photoinduced oxygen-deleting ring remodeling. General reaction conditions: Substrate (0.1 mmol) and CHI_3_ (0.2 mmol) in DMF (1 mL), r.t., 456 nm (20 W), 3 h, N_2_; then, LED off, 90 °C, 21 h; then, zinc powder (0.5 mmol) or nucleophiles (0.5 mmol with bases). Yields denote isolated yields. **a** Oxetanes were directly transformed into tetrahydroquinolines through oxygen deletion followed by cyclization and reductive protonation. **b** Incorporation of an additional functionality could be realized by switching Zn to various nucleophiles, delivering tetrahydroquinolines of greater complexity and diversity. **c** Rapid assembly of spirocyclic bis-tetrahydroquinolines was achieved by combining oxygen deletion with double cyclization. **d** Strained spirocyclic benzoheterocycles were obtained by remodeling of oxetanes tethered to alkyl bromide moieties. BTK, Bruton’s tyrosine kinase; comm., commercially.

Beyond ring contraction reactions, we envisioned that the synthetic value of the oxygen deletion manifold could be substantially expanded by exploring the extent of skeletal remodeling (Fig. 4). As illustrated in Fig. 2d, pre-functionalized oxetanes such as **13** could be restructured by making use of intramolecular nucleophilic cyclizations to trap the electrophilic alkyl iodide intermediate formed after removal of oxygen, unlocking opportunities to prepare a wide spectrum of useful benzoheterocyclic scaffolds such as **14**. Inspired by this conception, we subjected a range of 3,3-disubstituted oxetanes carrying methylaniline units to the established conditions for oxygen deletion/cyclization/reductive protonation and successfully isolated **44** − **46** in a single vessel (Fig. 4a). Notably, formation of cyclopropane products was not observed in these reactions, indicating that the Friedel-Crafts cyclization proceeded to completion due to the nucleophilic aniline moiety. Interestingly, chroman **47** and cyclopropane **16** (Fig. 3) originated from the same oxetane substrate that bears a relatively less nucleophilic methylnaphthol moiety. Performing the standard reaction at 90 ^o^C only afforded **16**, and a higher reaction temperature (120 ^o^C) was necessary to promote cyclization prior to Zn treatment. However, elevated temperatures led to increased side reactions and substrate decomposition, which likely account for the diminished yield of **47**. These findings highlight the profound influence of temperature on the reaction outcome.

Instead of protonation, we could also tune the reaction system to install an additional functional group by promoting nucleophilic substitution of the alkyl iodide handle (see **VII** in Fig. 2a), which would further increase molecular complexity. To this end, we successfully incorporated an amine (**48**), a phenol (**49**) and a carboxylic acid (**50**) to secure architecturally sophisticated tetrahydroquinolines (Fig. 4b). Interestingly, congested spirocyclic bis-tetrahydroquinolines (**51** − **53**) which might be useful as ionophore building blocks^26^ could be obtained through double intramolecular cyclization (Fig. 4c). Strained spirocyclic benzoheterocycles are privileged motifs found in many therapeutically active compounds^27^. By using oxetanes substituted with bromoalkane moieties at the 3-position, we could manipulate the reaction to orchestrate sequential oxygen deletion/cyclization/reductive coupling to assemble cyclopropyl-spirocycles **54** − **56** in a single operation (Fig. 4d). The examples in Fig. 4 highlight the present protocol as an enabling tool for upgrading simple oxetane substrates, readily accessed from cheap starting materials in one step, into structurally diverse drug-like heterocycles.

**Fig. 5:**
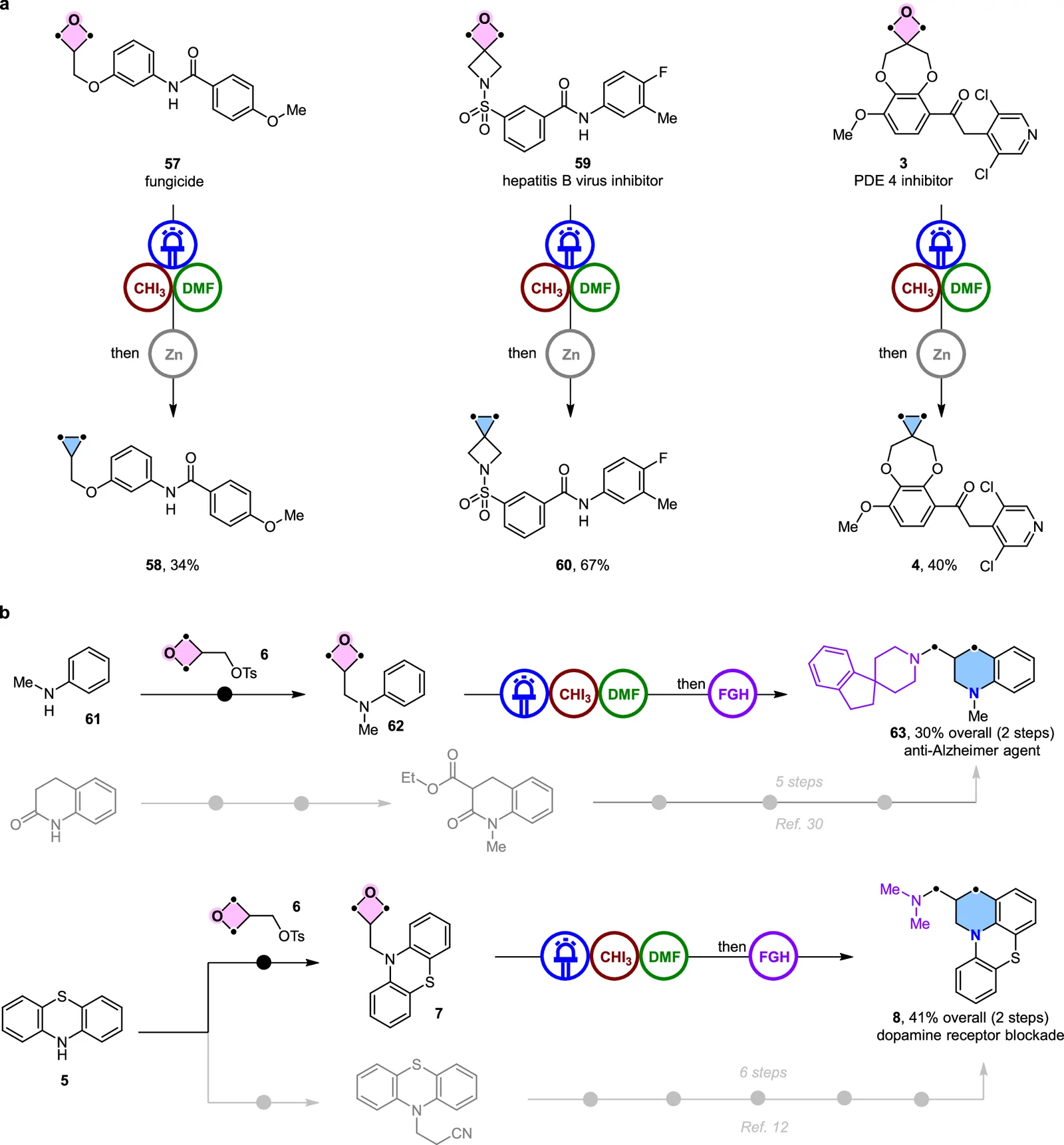
Application to late-stage editing and complex molecule synthesis. **a** Precise oxygen deletion of fungicide **57**, hepatitis B virus inhibitor **59** and PDE 4 inhibitor **3** afforded the corresponding cyclopropane analogs as potentially efficacious drug candidates. **b** Skeletal remodeling of functionalized oxetanes provided a distinct approach that decreased the synthetic burden of obtaining drug molecules such as anti-Alzheimer agent **63** and dopamine receptor blockade **8**.

Late-stage editing of multifunctional bioactive molecules was performed to demonstrate utility of the photoinduced oxygen deletion regime (Fig. 5a). Fungicide candidate **57**^28^, hepatitis B virus inhibitor **59**^29^ and PDE 4 inhibitor **3**^10^ were conveniently converted to the desired products **58,** **60** and **4**, respectively, through precise ring contraction of the oxetane motif. This approach bypasses multi-step de novo synthesis and offers an expeditious route to generate libraries of drug-like cyclopropane analogs. We also applied the oxygen deletion strategy for skeletal remodeling to redesign truncated synthetic routes to tetrahydroquinoline drug targets (Fig. 5b). In contrast to traditional synthetic approaches for functionalized tetrahydroquinolines, our restructuring approach harnesses functionalized oxetanes as cheap and readily available building blocks to generate acyclic diiodide intermediates with two electrophilic sites, offering tunable reactivity modes by controlling the sequence of intramolecular cyclization and downstream functionalization through the addition of appropriate reagents. For example, the restructuring of oxetane **62** (one-step synthesis from commercially available **61** and **6**) by photoinduced oxygen deletion/cyclization/amination directly delivered the desired anti-Alzheimer agent **63**. The overall two-step process compared favorably with the alternative five-step synthesis in the literature^30^. The same strategy was used to simplify the synthesis of dopamine receptor blockade **8**. Starting from 10*H*-phenothiazine **5**, nucleophilic substitution with **6** furnished the key oxetane intermediate **7**, which was edited to give **8** in 41% yield over two steps (vs. previously reported six-step route^12^). Through purposeful remodeling of easily accessible oxetane feedstocks, significant savings in step count, resources, and time could be accomplished.

## Discussion

In summary, we have developed an efficient photoinduced oxygen deletion manifold that provides the premise for achieving skeletal restructuring of oxetanes. This catalyst-free method can be tuned to effect ring contraction or remodeling, accelerating access to medicinally relevant cyclopropanes, tetrahydroquinolines or chromans with potential applications for drug discovery. Despite the results reported herein, the corresponding oxygen deletion-driven transformations involving less reactive non-strained cyclic ethers^31^ remain difficult, and research is ongoing in our laboratories to address this challenge. We expect our work to inspire the future development of the atom deletion editing logic to reduce the synthetic burden of complex molecule synthesis and explore uncharted regions of the chemical space.

## Methods

### General procedures for photoinduced ring-contraction of oxetanes to access cyclopropanes (method A)

In a glovebox under nitrogen atmosphere, a 4 mL vial equipped with a magnetic stir bar was charged with oxetane substrate (0.2 mmol, 1.0 equiv.) and CHI_3_ (157.5 mg, 0.4 mmol, 2.0 equiv.) in anhydrous DMF (2 mL). The reaction vial was sealed and taken out of the glovebox. The mixture was allowed to stir at room temperature under 20 W blue LED (456 nm) illumination for 3 hours. Then, the blue LED was turned off, and the mixture was allowed to stir at 90 °C for 21 hours. After cooling to room temperature, zinc powder (65.4 mg, 1.0 mol, 5.0 equiv.) was added in one portion. Then, the mixture was allowed to stir at 90 °C for 20 min. After completion of the reaction, the crude mixture was purified by silica gel chromatography to provide the product.

### General procedures for photoinduced remodeling of oxetanes to access tetrahydroquinolines (method B)

In a glovebox under nitrogen atmosphere, a 4 mL vial equipped with a magnetic stir bar was charged with oxetane substrate (0.1 mmol, 1.0 equiv.) and CHI_3_ (78.7 mg, 0.2 mmol, 2.0 equiv.) in anhydrous DMF (1 mL). The reaction vial was sealed and taken out of the glovebox. The mixture was allowed to stir at room temperature under 20 W blue LED (456 nm) illumination for 3 hours. Then, the blue LED was turned off and the mixture was allowed to stir at 90 °C for 21 hours. After cooling to room temperature, additives were added in one portion for further protonation [zinc powder (32.7 mg, 0.5 mmol, 5.0 equiv.)] or functionalization [nucleophiles (0.5 mmol, 5.0 equiv.) with base]. After completion of the reaction, the crude mixture was purified by silica gel chromatography to provide the product.

## Supplementary information


Supplementary Information
Transparent Peer Review file


## Data Availability

Crystallographic data are available free of charge from the Cambridge Crystallographic Data Centre under reference nos. CCDC-2537914 (**36**), 2537939 (**48**), and 2537921 (**53**). All other data are available in the main text or the supplementary information. All data supporting the findings of this manuscript are also available from the corresponding author upon request.
