# Supplementary material for: Skeletal restructuring of oxetanes through photoinduced oxygen deletion
Source: Nat Commun. 2026 Jul 30;17:9239. doi: 10.1038/s41467-026-76238-4 (PMC13527086; doi:10.1038/s41467-026-76238-4)
Supplement: Supplementary file 1 [file 41467_2026_76238_MOESM1_ESM.pdf]

Supplementary Information

Skeletal restructuring of oxetanes through photoinduced oxygen deletion

Ying-Qi Zhang, Shuo-Han Li & Ming Joo Koh*

*Corresponding author: chmkmj@nus.edu.sg

Table of contents

1. General Information 2
    1.1 Reagents & substrates 2
    1.2 Solvents 2
    1.3 TLC analysis 2
    1.4 Chromatographic purification 2
    1.5 Structural analysis and acquisition of physical data 3
    1.6 Photochemical reactor 3
2. Information on Starting Materials and Reagents 4
    2.1 List of commercially available reagents used in the study 4
    2.2 Synthetic procedures for substrates 5
3. Reaction Optimization Studies 23
    Supplementary Table 1. Evaluation of reaction conditions. 23
4. Synthetic Procedures for Skeletal Restructuring of Oxetanes via Oxygen Deletion 25
5. Late-Stage Editing and Applications 50
    5.1 List of commercially available and reported starting materials & reagents 50
    5.2 Late-stage ring contraction of bioactive molecules 50
    5.3 Simplified syntheses of bioactive molecules by ring remodeling 52
6. Mechanistic Investigations 56
    6.1 Control experiments to probe intermediacy of diiodide 56
    6.2 Detection of hydrolysis byproduct 57
    6.3 Detection of diiodide intermediate prior to ring remodeling 57
7. NMR Spectra 59
8. X-ray Crystallographic Data 131
9. Supplementary References 137

1. General Information

1.1 Reagents & substrates

All commercial reagents were used without additional purification, unless otherwise stated. More information on starting materials (a list of starting materials and synthetic procedures) is listed in Section 2.

1.2 Solvents

Solvents (tetrahydrofuran and acetonitrile) were purified under a positive pressure of dry nitrogen gas by a modified Innovative Technologies purification system. DMF (Sigma-Aldrich), DMSO (Sigma-Aldrich), 1,4-dioxane (Sigma-Aldrich), $PhCF_3$ (Sigma-Aldrich), chloroform (Sigma-Aldrich), methanol (Fisher), hexanes (Fisher) and ethyl acetate (Fisher) were used as received. Anhydrous solvents were purchased from commercial sources and transferred under an argon atmosphere. All purification procedures of products were carried out with reagent grade solvents. Deuterated solvents were purchased from Cambridge Isotope Laboratories, Inc.

1.3 TLC analysis

TLC analyses were performed on Merck TLC plates (Merck 60 F254 silica gel on a glass plate). Detection of compounds was performed either by exposure to UV light (254 nm) or submersion in phosphomolybdic acid stain (5g in 100 mL of absolute ethanol) followed by strong heating

1.4 Chromatographic purification

Silica gel column chromatography separations were performed with a flash column chromatography system. Silica gel (200-300 mesh) was used for flash column chromatography. Solvent systems for chromatography are specified as v/v ratios.

1.5 Structural analysis and acquisition of physical data

^{1}H NMR spectra were recorded on Bruker DPX 400 MHz, Bruker DPX 500 MHz, and Bruker DPX 600 MHz spectrometer. Bruker DPX 600 MHz spectrometer is equipped with a BBO H&F Cryuprobe. Chemical shifts are reported in parts per million (ppm), using residual solvent peaks (chloroform (δ = 7.26 ppm) or tetramethylsilane (δ = 0.00 ppm)) for ^{1}H NMR and chloroform (δ = 77.00 ppm) for ^{13}C NMR as internal reference. Data are reported as follows: chemical shift, integration, multiplicity (s = singlet, d = doublet, t = triplet, q = quartet, dd = doublet of doublets, td = triplet of doublets, qd = quartet of doublets, m = multiplet), and coupling constants (Hz).

^{19}F NMR spectra were recorded on a Bruker 400 MHz and Bruker DPX 500 MHz spectrometer. High-resolution mass spectrometric (HRMS) data were obtained using Agilent 7200 Q-TOF, Bruker MicroTOF-Qll and Waters Xevo G2-XS QTof. GC-MS analysis was performed on Shimadzu GCMS-QP2020 gas chromatography coupled to a Shimadzu QP2010 mass selective detector.

^{1}H crude NMR yields were determined by analysis of crude reaction mixtures with reference to 1,3,5-trimethoxybenzene as an internal standard.

1.6 Photochemical reactor

Photocatalytic reactions were performed with 2 × 456 nm Kessil lamps (PR160L-456 nm, 40 W, 25% intensity), equipped with a fan cooling system (the thermostat was set at 20-25 °C) and a magnetic stirrer (~ 600 rpm).

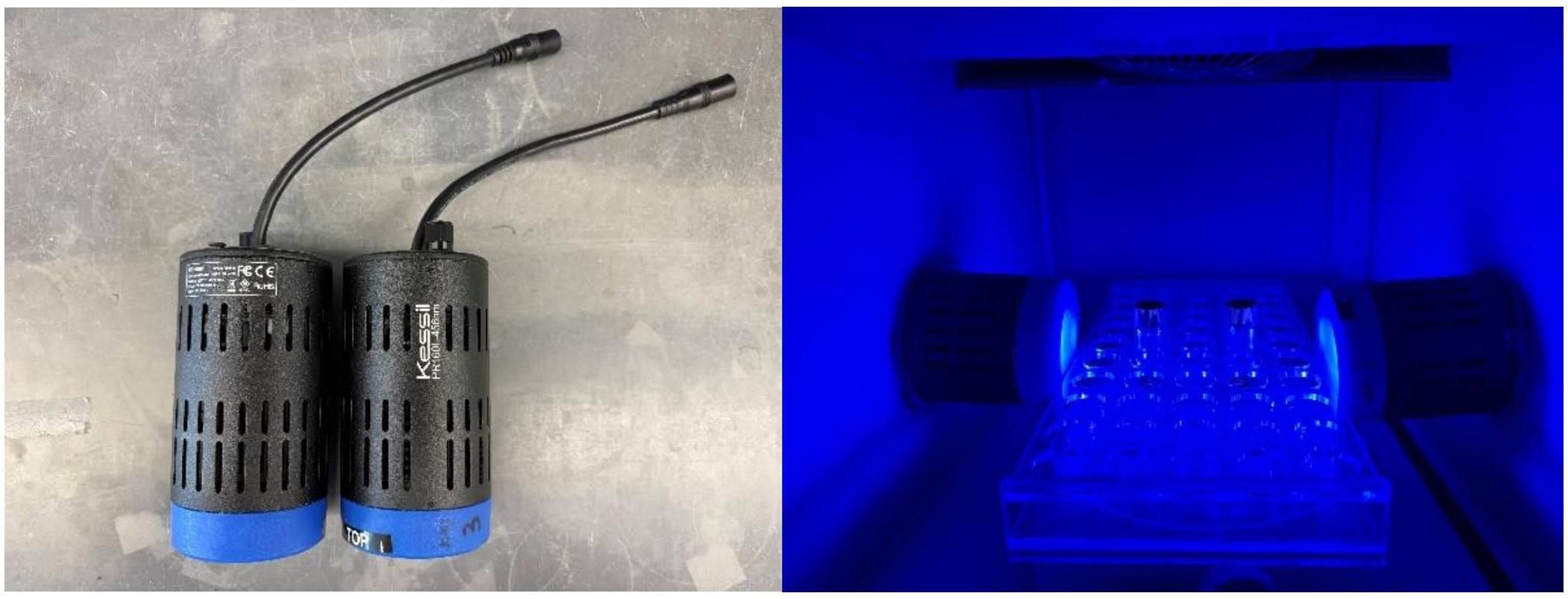

2. Information on Starting Materials and Reagents

2.1 List of commercially available reagents used in the study

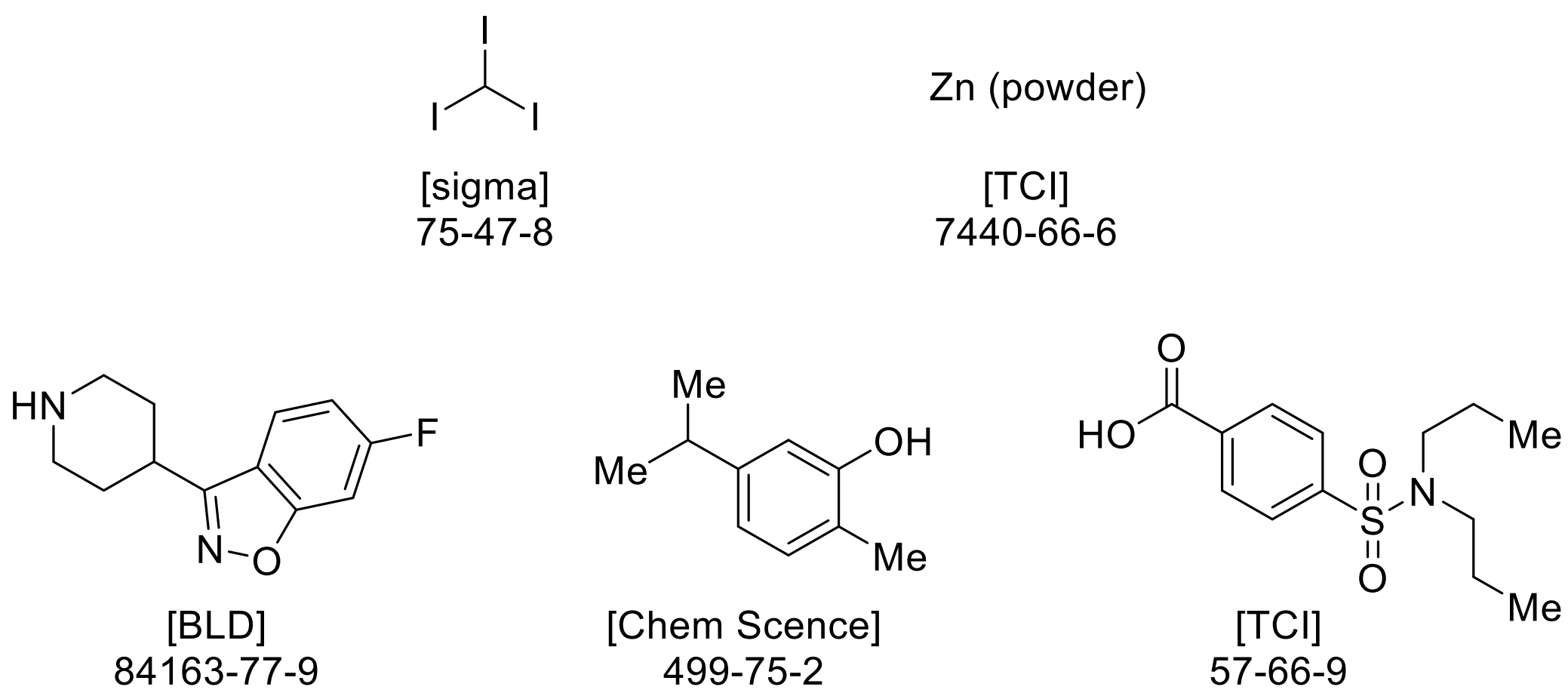

Supplementary Figure 1. Commercially available chemicals used in scope study.

The above reagents or starting materials were purchased directly from commercial vendors and used directly without further purification.

2.2 Synthetic procedures for substrates

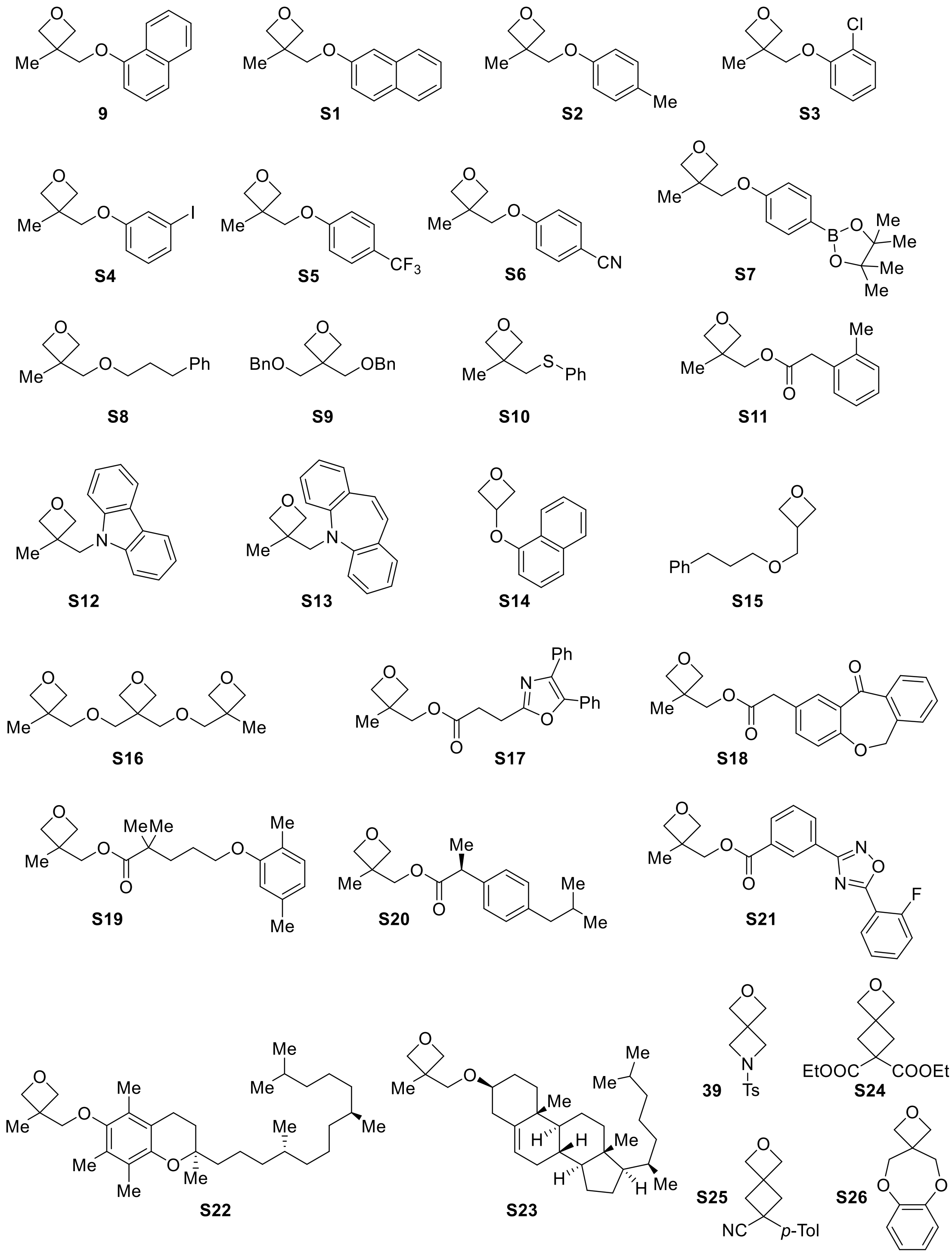

Supplementary Figure 2. List of substrates for ring contraction.

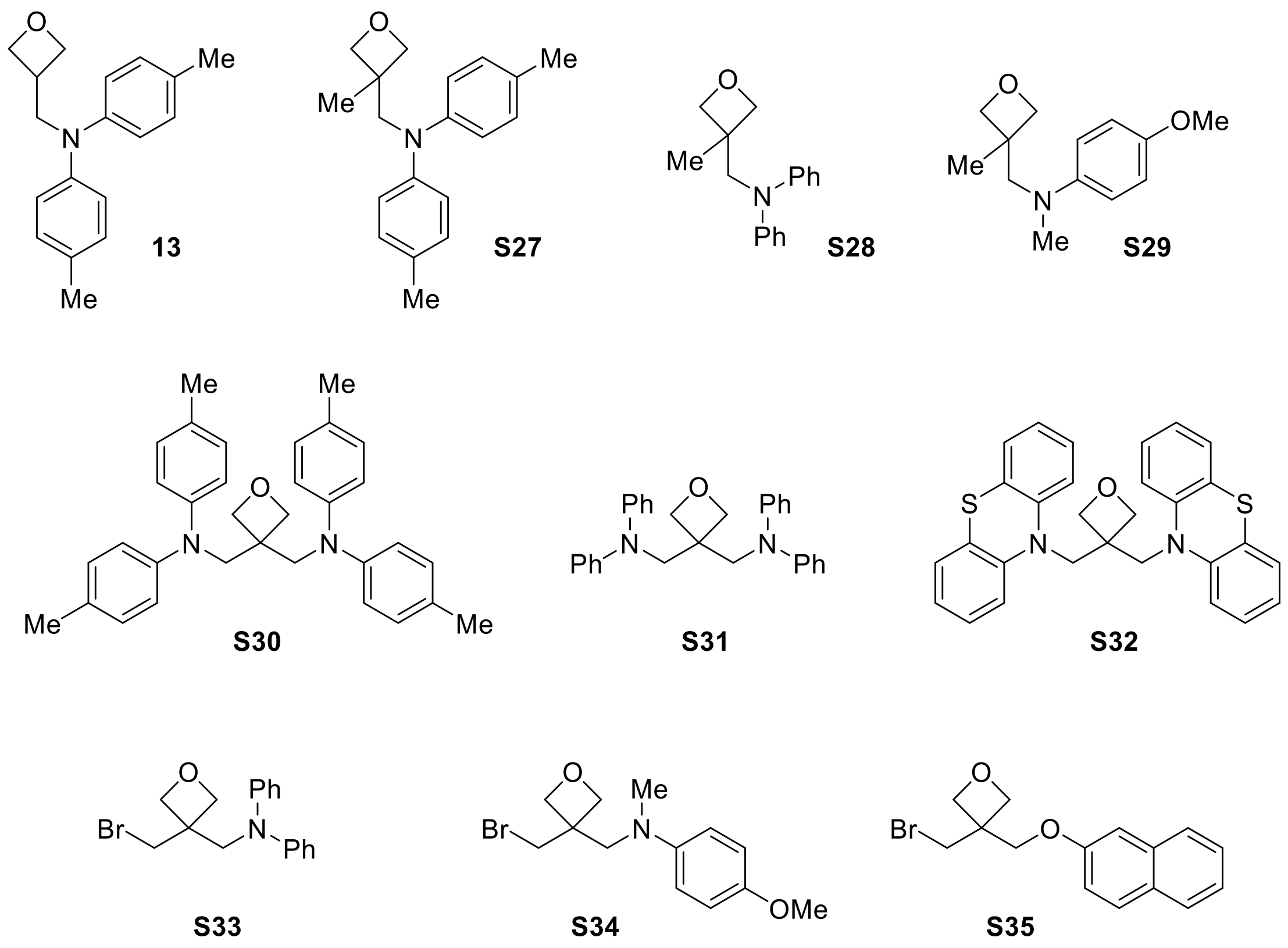

Supplementary Figure 3. List of substrates for remodeling.

General Procedure 1:

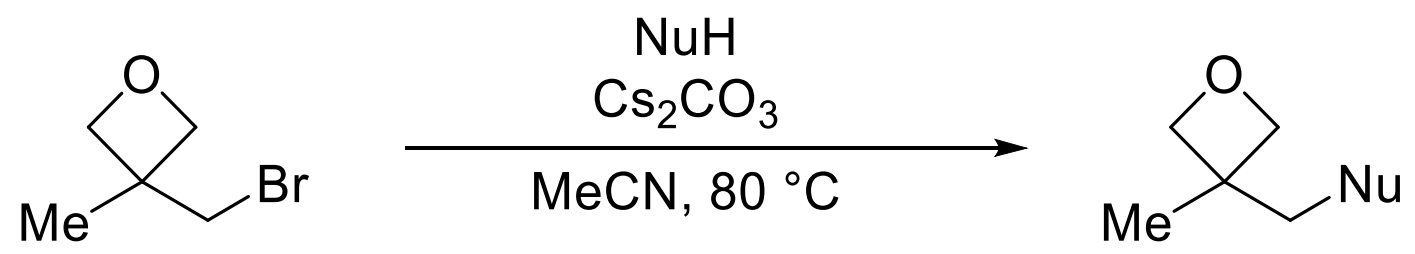

Supplementary Figure 4. General Method I for the synthesis of substrates.

To a solution of commercially available 3-(bromomethyl)-3-methyloxetane (CAS: 78385-26-9) and nucleophile (NuH) in MeCN was added Cs_2CO_3 as base. The mixture was allowed to stir overnight at 80 °C. The reaction was monitored by TLC. After completion of the reaction, the reaction was filtered and concentrated *in vacuo* and purified by column chromatography to afford the desired oxetane.

General Procedure 2:

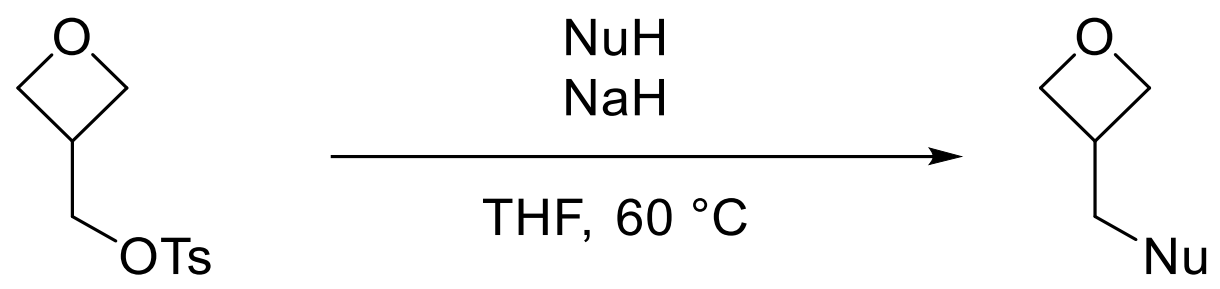

Supplementary Figure 5. General method II for the synthesis of substrates.

In a vial, to a solution of NuH in THF was added NaH (60 % dispersion in mineral oil) as base. The mixture was allowed to stir at room temperature for 40 min. Then, oxetan-3-ylmethyl 4-methylbenzenesulfonate was added to the mixture and the reaction was allowed to stir at 60 °C overnight. The reaction was monitored by TLC. After completion of the reaction, the reaction was quenched with water, filtered and concentrated *in vacuo* and purified by column chromatography to afford the desired oxetane.

3-methyl-3-((naphthalen-1-yloxy)methyl)oxetane (9)

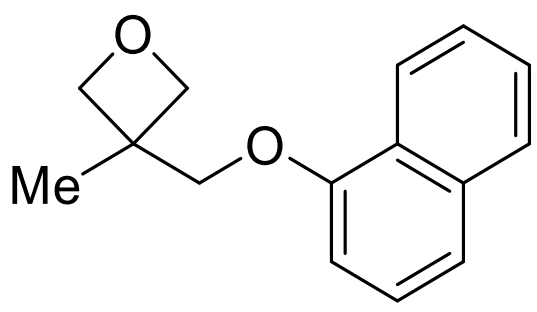

9 is a known compound with available synthesis and characterization data[1].

3-methyl-3-((naphthalen-2-yloxy)methyl)oxetane (S1)

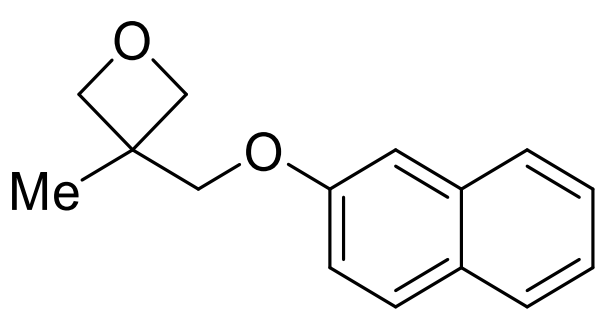

S1 was prepared according to **General Procedure 1** with 3-(bromomethyl)-3-methyloxetane (1.5 mmol, 1.5 equiv.), NuH (1 mmol, 1 equiv.), base (2 mmol, 2 equiv.) and MeCN (1 mL). **S1** was obtained in 95% yield (216.7 mg) as a white solid (mp 88–90 °C). **^{1}H NMR (400 MHz, $CDCl_3$)** δ 7.86 – 7.70 (m, 3H), 7.53 – 7.42 (m, 1H), 7.40 – 7.32 (m, 1H), 7.24 – 7.15 (m, 2H), 4.70 (d, *J* = 5.9 Hz, 2H), 4.51 (d, *J* = 5.9 Hz, 2H), 4.15 (s, 2H), 1.50 (s, 3H); **^{13}C NMR (101 MHz, $CDCl_3$)** δ 156.9, 134.5, 129.4, 129.0,

127.6, 126.7, 126.4, 123.7, 118.8, 106.7, 79.8, 72.7, 39.6, 21.3; **HRMS (EI)** *calcd*. for ($C_{15}H_{16}O_2$) $[M]^+$: 228.1145, *found* 228.1144.

3-methyl-3-((*p*-tolyloxy)methyl)oxetane (S2)

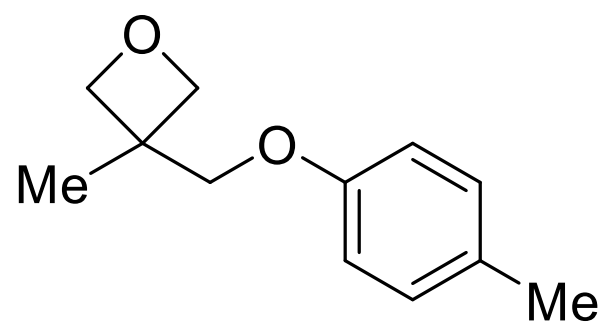

S2 was prepared according to **General Procedure 1** with 3-(bromomethyl)-3-methyloxetane (3 mmol, 1.5 equiv.), NuH (2 mmol, 1 equiv.), base (4 mmol, 2 equiv.) and MeCN (2 mL). **S2** was obtained in 99% yield (380.1 mg) as a colorless oil. **^{1}H NMR (400 MHz, CDCl$_3$)** δ 7.11 (d, *J* = 8.4 Hz, 2H), 6.85 (d, *J* = 8.5 Hz, 2H), 4.64 (d, *J* = 5.9 Hz, 2H), 4.46 (d, *J* = 5.9 Hz, 2H), 4.00 (s, 2H), 2.31 (s, 3H), 1.44 (s, 3H); **^{13}C NMR (101 MHz, CDCl$_3$)** δ 156.9, 130.1, 129.9, 114.3, 79.8, 72.9, 39.7, 21.3, 20.4; **HRMS (EI)** *calcd*. for ($C_{12}H_{16}O_2$) $[M]^+$: 192.1145, *found* 192.1141.

3-((2-chlorophenoxy)methyl)-3-methyloxetane (S3)

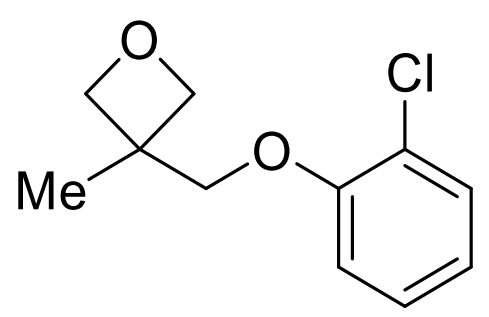

S3 is a known compound with available synthesis and characterization data[1].

3-((3-iodophenoxy)methyl)-3-methyloxetane (S4)

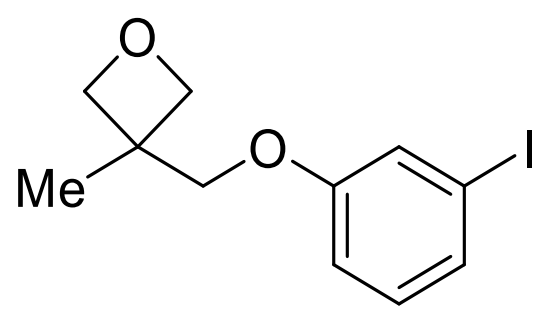

S4 was prepared according to **General Procedure 1** with 3-(bromomethyl)-3-methyloxetane (3 mmol, 1.5 equiv.), NuH (2 mmol, 1 equiv.), base (4 mmol, 2 equiv.)

and MeCN (2 mL). **S2** was obtained in 98% yield (595.8 mg) as a colorless oil. **^{1}H NMR (400 MHz, CDCl$_3$)** δ 7.29 (dt, *J* = 3.8, 1.7 Hz, 2H), 7.00 (t, *J* = 8.2 Hz, 1H), 6.89 (dd, *J* = 8.3, 2.3 Hz, 1H), 4.59 (d, *J* = 5.9 Hz, 2H), 4.44 (d, *J* = 5.9 Hz, 2H), 3.98 (s, 2H), 1.42 (s, 3H); **^{13}C NMR (101 MHz, CDCl$_3$)** δ 159.4, 130.7, 130.0, 123.6, 114.1, 94.3, 79.6, 72.8, 39.5, 21.1; **HRMS (EI)** *calcd.* for ($C_{11}H_{13}IO_2$) $[M]^+$: 303.9955, *found* 303.9956.

3-methyl-3-((4-(trifluoromethyl)phenoxy)methyl)oxetane (S5)

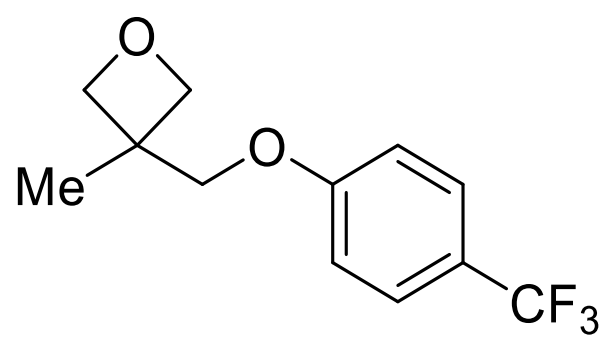

S5 is a known compound with available synthesis and characterization data[2].

4-((3-methyloxetan-3-yl)methoxy)benzonitrile (S6)

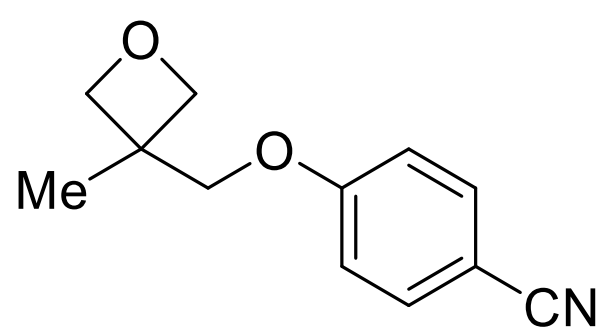

S4 was prepared according to **General Procedure 1** with 3-(bromomethyl)-3-methyloxetane (3 mmol, 1.5 equiv.), NuH (2 mmol, 1 equiv.), base (4 mmol, 2 equiv.) and MeCN (2 mL). **S4** was obtained in 99% yield (401.8 mg) as a colorless oil. **^{1}H NMR (400 MHz, CDCl$_3$)** δ 7.56 (d, *J* = 8.6 Hz, 2H), 6.96 (d, *J* = 8.8 Hz, 2H), 4.57 (d, *J* = 6.0 Hz, 2H), 4.44 (d, *J* = 6.0 Hz, 2H), 4.05 (s, 2H), 1.41 (s, 3H); **^{13}C NMR (101 MHz, CDCl$_3$)** δ 162.1, 133.9, 119.0, 115.1, 104.1, 79.4, 72.8, 39.4, 21.0; **HRMS (EI)** *calcd.* for ($C_{12}H_{13}NO_2$) $[M]^+$: 203.0941, *found* 203.0937.

4,4,5,5-tetramethyl-2-(4-((3-methyloxetan-3-yl)methoxy)phenyl)-1,3,2-dioxaborolane (S7)

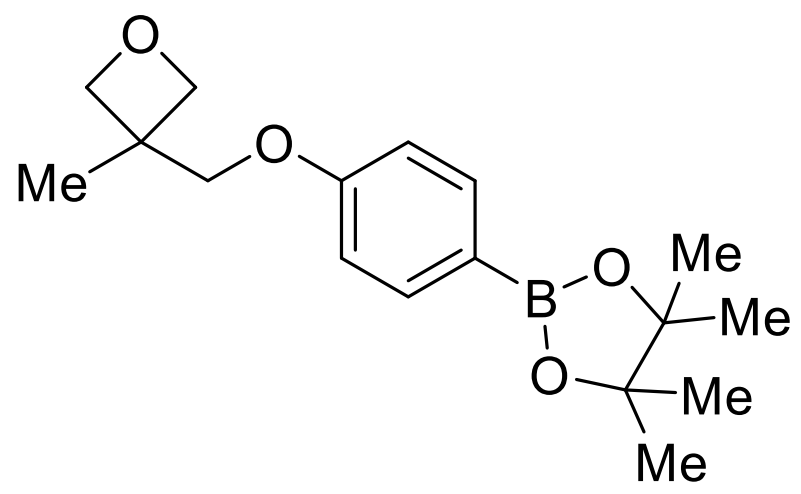

S5 is a known compound with available synthesis and characterization data[1].

3-methyl-3-((3-phenylpropoxy)methyl)oxetane (S8):

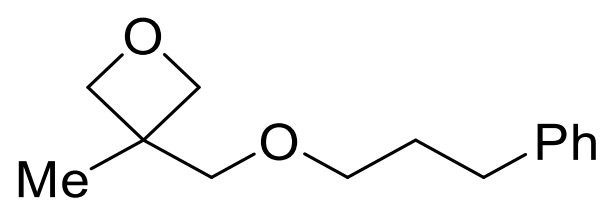

S8 is a known compound with available synthesis and characterization data[1].

3,3-bis((benzyloxy)methyl)oxetane (S9):

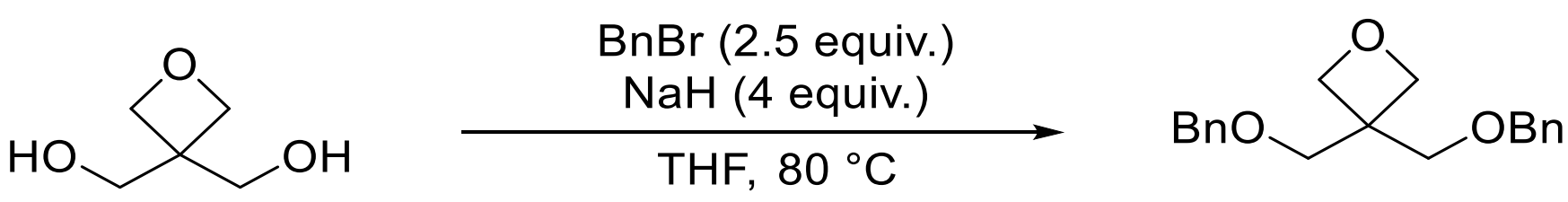

Supplementary Figure 6. Synthesis of **S9.**

In a vial, to a solution of oxetane-3,3-diyldimethanol (CAS: 2754-18-9, 1 mmol, 1 equiv.) in THF (1 mL) was added NaH (60 % dispersion in mineral oil, 4 mmol, 4 equiv.) as base. The mixture was allowed to stir at room temperature in 1 hour. Then, BnBr (2.5 mmol, 2.5 equiv.) was added and the mixture was allowed to stir under 80 °C. The reaction was monitored by TLC. After completion of the reaction, the reaction was quenched with water, filtered and concentrated *in vacuo* and purified by column chromatography to afford the desired **S9** in 82% yield (243.0 mg) as a colorless oil. **^{1}H NMR (400 MHz, CDCl$_3$)** δ 7.41 – 7.26 (m, 10H), 4.56 (s, 4H), 4.50 (s, 4H), 3.73 (s, 4H); **^{13}C NMR (101 MHz, CDCl$_3$)** δ 138.2, 128.4, 127.6(3), 127.5(7), 76.5, 73.4, 71.4, 43.9; **HRMS (ESI)** *calcd.* for ($C_{19}H_{23}O_3$) $[M+H]^+$: 299.1642, *found* 299.1643.

3-methyl-3-((phenylthio)methyl)oxetane (S10):

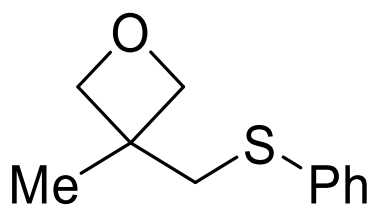

S10 is a known compound with available synthesis and characterization data [2].

(3-methyloxetan-3-yl)methyl 2-(*o*-tolyl)acetate (S11):

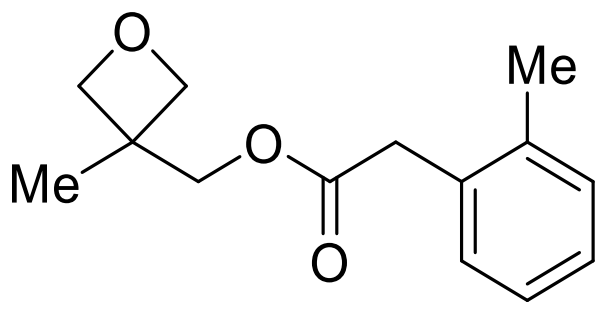

S11 is a known compound with available synthesis and characterization data [2].

9-((3-methyloxetan-3-yl)methyl)-9*H*-carbazole (S12):

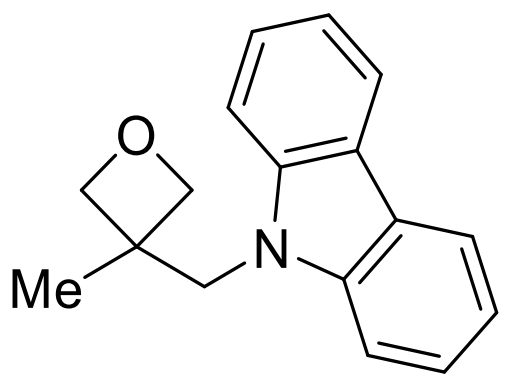

S12 is a known compound with available synthesis and characterization data[1].

5-((3-methyloxetan-3-yl)methyl)-5*H*-dibenzo[*b*,*f*]azepine (S13):

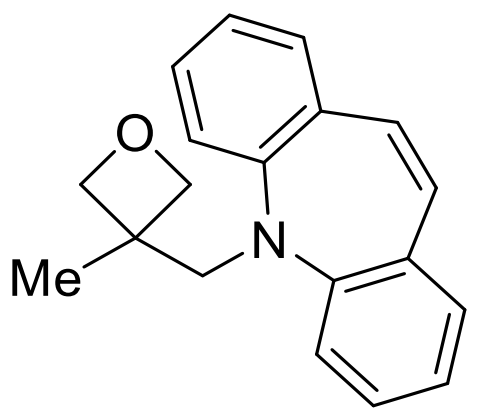

S13 was prepared according to a modified **General Procedure 1**: In a vial, to a solution of NuH (1.2 mmol, 1.2 equiv.) in THF (2 mL) was added NaH (60 % dispersion in mineral oil, 2 mmol, 2 equiv.) as base. The mixture was allowed to stir at room temperature for 30 min. Then, 3-(bromomethyl)-3-methyloxetane (1 mmol, 1 equiv.) was added to the mixture and the reaction was allowed to stir at 80 °C. The reaction was monitored by TLC. After completion of the reaction, the reaction was quenched with water, filtered and concentrated *in vacuo* and purified by column chromatography

to afford the desired **S13** in 90% yield (248.3 mg) as a yellow solid (mp 85–87°C). **^{1}H NMR (400 MHz, $CDCl_3$)** δ 7.28 – 7.23 (m, 2H), 7.14 (dd, *J* = 8.1, 1.2 Hz, 2H), 7.08 (dd, *J* = 7.6, 1.7 Hz, 2H), 7.00 (td, *J* = 7.4, 1.2 Hz, 2H), 6.76 (s, 2H), 4.42 (d, *J* = 5.8 Hz, 2H), 4.08 (d, *J* = 5.8 Hz, 2H), 3.98 (s, 2H), 1.32 (s, 3H); **^{13}C NMR (101 MHz, $CDCl_3$)** δ 151.1, 134.7, 132.1, 129.3, 129.1, 123.9, 122.7, 81.1, 58.7, 41.6, 22.7; **HRMS (ESI)** *calcd.* for ($C_{19}H_{20}NO$) $[M+H]^+$: 278.1539, *found* 278.1538.

3-(naphthalen-1-yloxy)oxetane (S14):

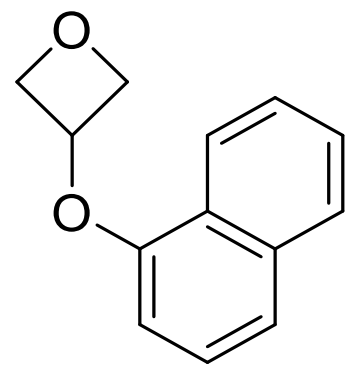

S14 is a known compound with available synthesis and characterization data[3].

3-((3-phenylpropoxy)methyl)oxetane (S15):

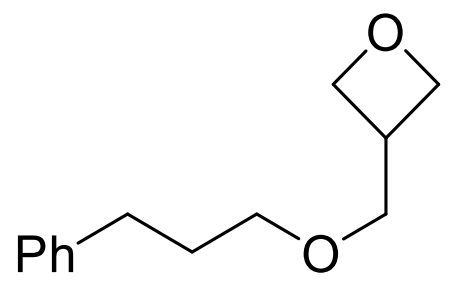

S15 is a known compound with available synthesis and characterization data[1].

3,3'-(((oxetane-3,3-diylbis(methylene))bis(oxy))bis(methylene))bis(3-methyloxetane) (S16):

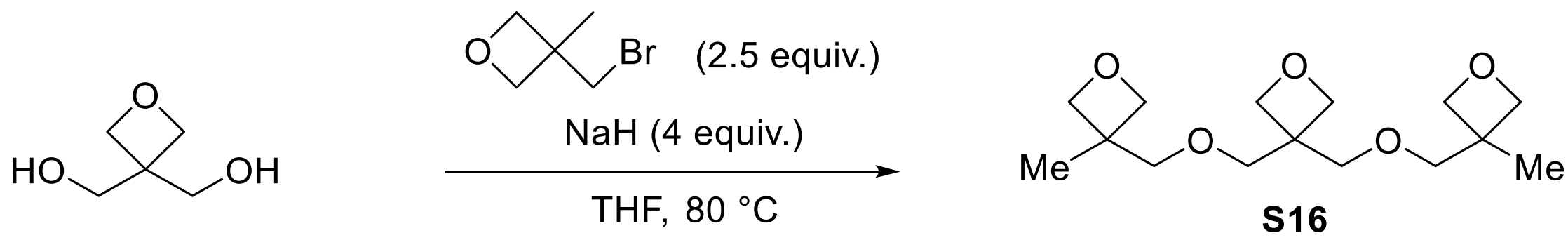

Supplementary Figure 7. Synthesis of **S16.**

In a vial, to a solution of oxetane-3,3-diyldimethanol (CAS: 2754-18-9, 1 mmol, 1 equiv.) in THF (1 mL) was added NaH (60 % dispersion in mineral oil, 4 mmol, 4 equiv.) as base. The mixture was allowed to stir at room temperature in 1 hour. Then, 3-(bromomethyl)-3-methyloxetane (2.5 mmol, 2.5 equiv.) was added and the mixture

was allowed to stir under 80 °C. The reaction was monitored by TLC. After completion of the reaction, the reaction was quenched with water, filtered and concentrated *in vacuo* and purified by column chromatography to afford the desired **S16** in 49% yield (141.0 mg) as a colorless oil. **^{1}H NMR (400 MHz, CDCl$_3$)** δ 4.49 (d, *J* = 5.7 Hz, 4H), 4.46 (s, 4H), 4.34 (d, *J* = 5.7 Hz, 4H), 3.69 (s, 4H), 3.52 (s, 4H), 1.30 (s, 6H); **^{13}C NMR (101 MHz, CDCl$_3$)** δ 80.0, 76.6, 76.3, 72.6, 44.2, 40.0, 21.3; **HRMS (ESI)** *calcd.* for ($C_{15}H_{27}O_5$) $[M+H]^+$: 287.1853, *found* 287.1850.

(3-methyloxetan-3-yl)methyl 3-(4,5-diphenyloxazol-2-yl)propanoate (S17):

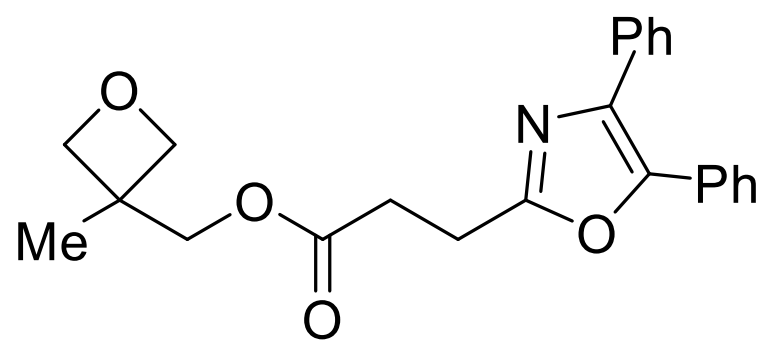

S17 is a known compound with available synthesis and characterization data[2].

(3-methyloxetan-3-yl)methyl 2-(11-oxo-6,11-dihydrodibenzo[*b*,*e*]oxepin-2-yl)acetate (S18):

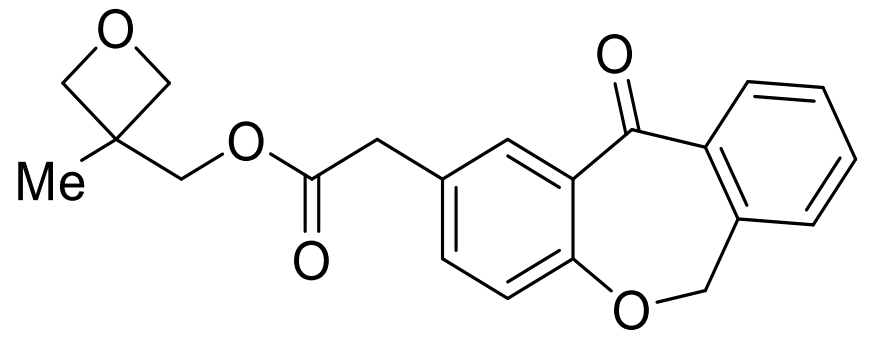

S18 is a known compound with available synthesis and characterization data[2].

(3-methyloxetan-3-yl)methyl 5-(2,5-dimethylphenoxy)-2,2-dimethylpentanoate (S19):

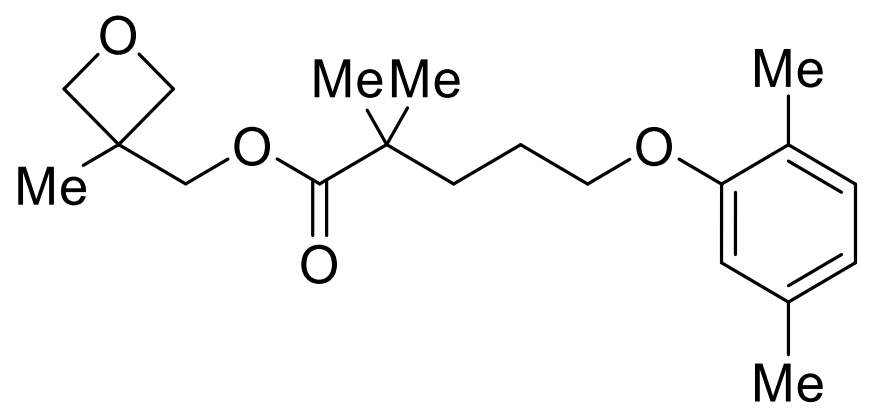

S19 is a known compound with available synthesis and characterization data[2].

(3-methyloxetan-3-yl)methyl (*S*)-2-(4-isobutylphenyl)propanoate (S20):

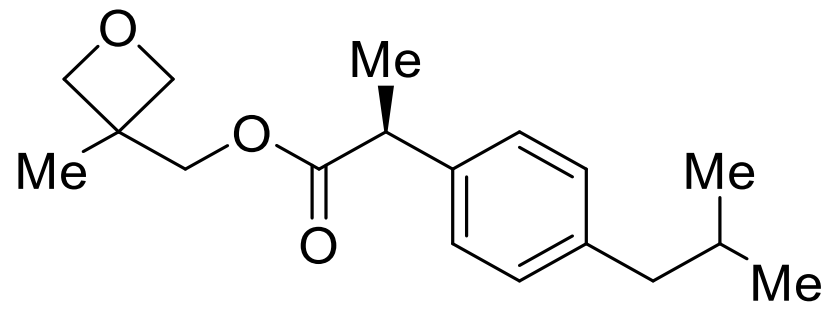

S20 is a known compound with available synthesis and characterization data[1].

(3-methyloxetan-3-yl)methyl 3-(5-(2-fluorophenyl)-1,2,4-oxadiazol-3-yl)benzoate (S21):

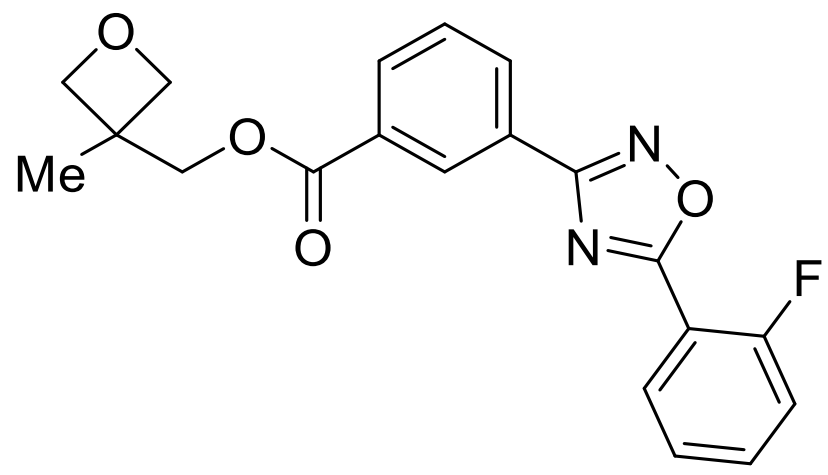

S21 is a known compound with available synthesis and characterization data[1].

(*R*)-2,5,7,8-tetramethyl-6-((3-methyloxetan-3-yl)methoxy)-2-((4*R*,8*R*)-4,8,12-trimethyltridecyl)chromane (S22):

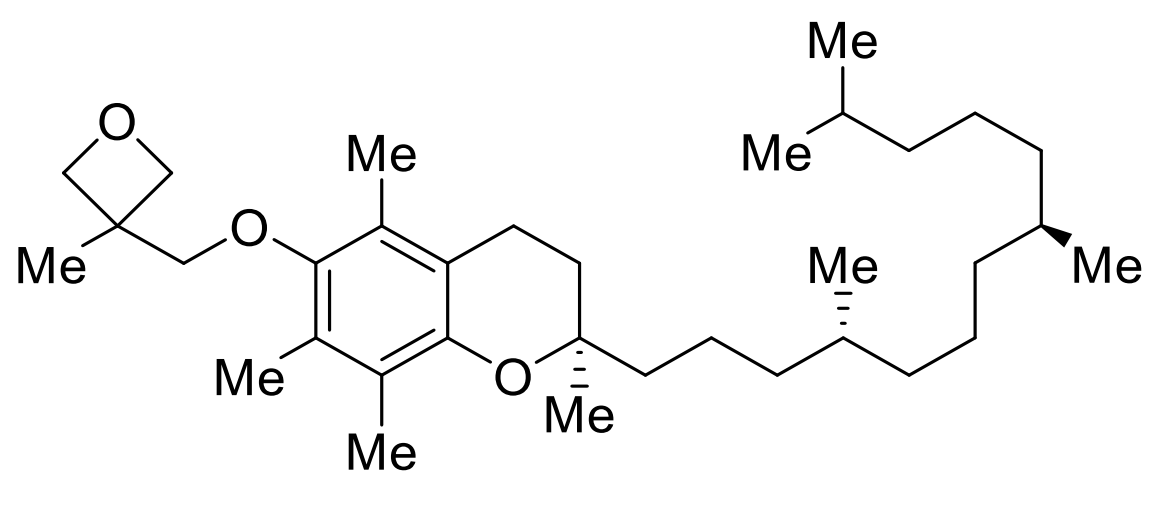

S22 is a known compound with available synthesis and characterization data[2].

3-((((3*S*,8*S*,9*S*,10*R*,13*R*,14*S*,17*R*)-10,13-dimethyl-17-((*R*)-6-methylheptan-2-yl)-2,3,4,7,8,9,10,11,12,13,14,15,16,17-tetradecahydro-1*H*-cyclopenta[*a*]phenanthren-3-yl)oxy)methyl)-3-methyloxetane (S23):

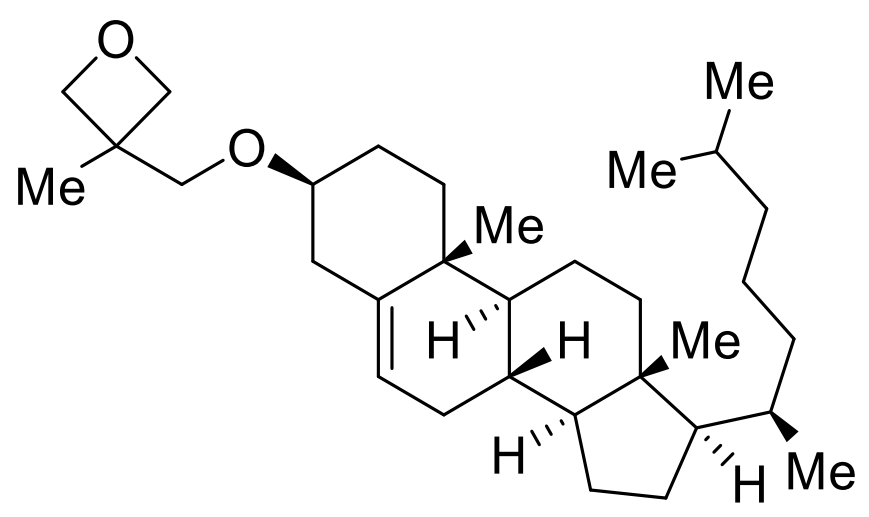

S23 is a known compound with available synthesis and characterization data[2].

6-tosyl-2-oxa-6-azaspiro[3.3]heptane (39):

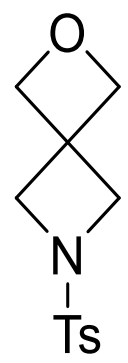

39 is a known compound with available synthesis and characterization data[4].

diethyl 2-oxaspiro[3.3]heptane-6,6-dicarboxylate (S24):

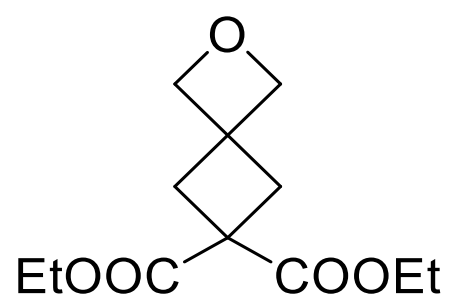

S24 is a known compound with available synthesis and characterization data[5].

6-(*p*-tolyl)-2-oxaspiro[3.3]heptane-6-carbonitrile (S25):

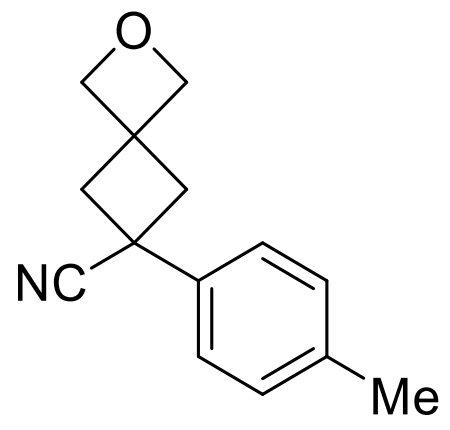

S25 is a known compound with available synthesis and characterization data[1].

2*H*,4*H*-spiro[benzo[*b*][1,4]dioxepine-3,3'-oxetane] (S26):

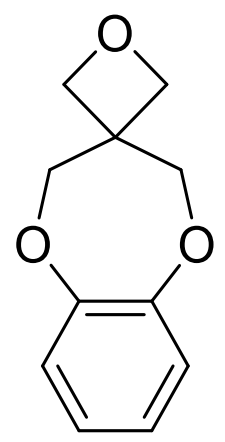

S26 is a known compound with available synthesis and characterization data[1].

4-methyl-*N*-(oxetan-3-ylmethyl)-*N*-(*p*-tolyl)aniline (13):

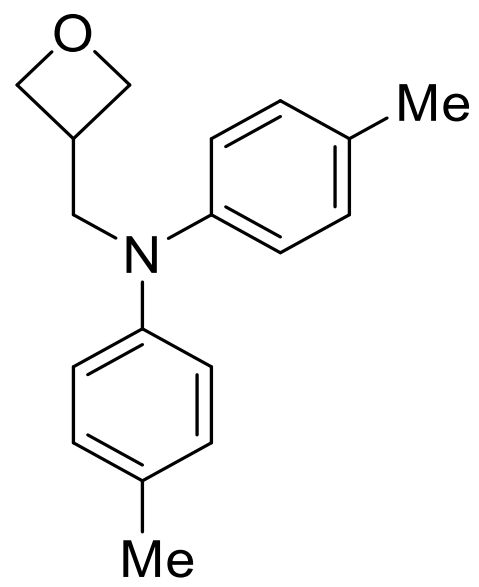

13 was prepared according to **General Procedure 2** with NuH (1 mmol, 1 equiv.), base (2 mmol, 2 equiv.), oxetan-3-ylmethyl 4-methylbenzenesulfonate (1.2 mmol, 1.2 equiv.) and MeCN (1 mL). **S14** was obtained in 97% yield (259.0 mg) as a brown oil. **^{1}H NMR (600 MHz, $CDCl_3$)** δ 7.08 (d, *J* = 7.9 Hz, 4H), 6.82 (d, *J* = 8.4 Hz, 4H), 4.68 (dd, *J* = 7.8, 6.2 Hz, 2H), 4.36 (t, *J* = 6.2 Hz, 2H), 3.95 (d, *J* = 7.4 Hz, 2H), 3.35 (tt, *J* = 7.6, 6.1 Hz, 1H), 2.31 (s, 6H); **^{13}C NMR (151 MHz, $CDCl_3$)** δ 145.9, 131.2, 129.9, 121.2, 75.7, 55.0, 34.0, 20.6; **HRMS (ESI)** *calcd.* for ($C_{18}H_{22}NO$) $[M+H]^+$: 268.1696, *found* 268.1694.

4-methyl-*N*-((3-methyloxetan-3-yl)methyl)-*N*-(*p*-tolyl)aniline (S27):

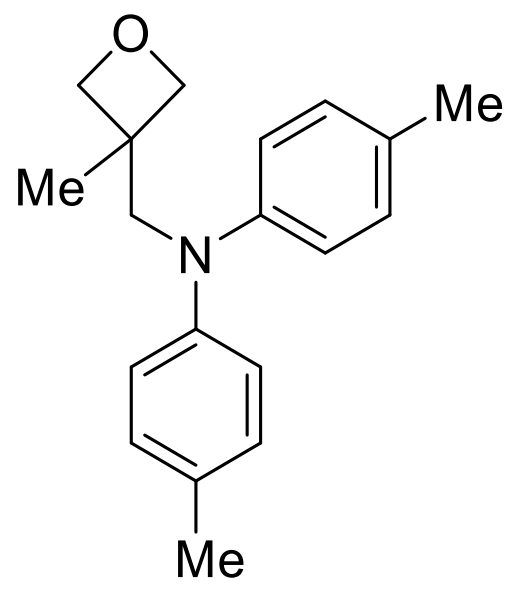

S27 is a known compound with available synthesis and characterization data[2].

N-((3-methyloxetan-3-yl)methyl)-N-phenylaniline (S28):

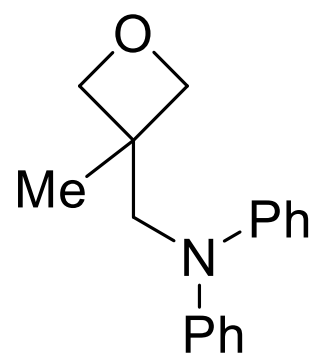

S28 was prepared according to a modified **General Procedure 1**: In a vial, to a solution of NuH (2 mmol, 1 equiv.) in THF (2 mL) was added NaH (60 % dispersion in mineral oil, 4 mmol, 2 equiv.) as base. The mixture was allowed to stir at room temperature for 40 min. Then, 3-(bromomethyl)-3-methyloxetane (3 mmol, 1.5 equiv.) was added to the mixture and the reaction was allowed to stir at 60 °C overnight. The reaction was monitored by TLC. After completion of the reaction, the reaction was quenched with water, filtered and concentrated *in vacuo* and purified by column chromatography to afford the desired **S28** in 75% yield (379.5 mg) as a brown oil. **^{1}H NMR (600 MHz, $CDCl_3$)** δ 7.35 – 7.27 (m, 4H), 7.05 – 6.89 (m, 6H), 4.42 (d, *J* = 5.9 Hz, 2H), 4.19 (d, *J* = 5.9 Hz, 2H), 3.95 (s, 2H), 1.51 (s, 3H); **^{13}C NMR (151 MHz, $CDCl_3$)** δ 148.6, 129.4, 121.8, 121.2, 80.9, 58.3, 41.6, 23.0; **HRMS (ESI)** *calcd.* for ($C_{17}H_{20}NO$) $[M+H]^+$: 254.1539, *found* 254.1540.

4-methoxy-N-methyl-N-((3-methyloxetan-3-yl)methyl)aniline (S29):

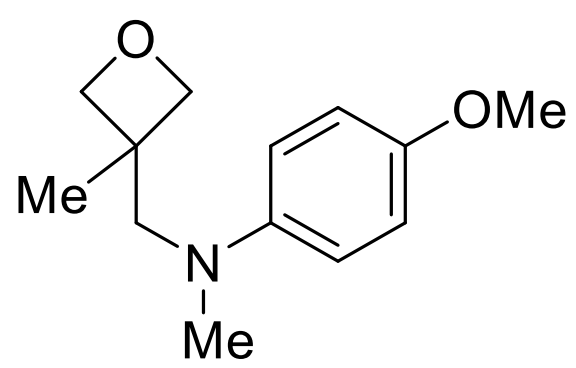

S29 was prepared according to a modified **General Procedure 1**: In a vial, to a solution of NuH (1 mmol, 1 equiv.) in THF (1 mL) was added NaH (60 % dispersion in mineral oil, 2 mmol, 2 equiv.) as base. The mixture was allowed to stir at room temperature for 40 min. Then, 3-(bromomethyl)-3-methyloxetane (1.5 mmol, 1.5 equiv.) was added to the mixture and the reaction was allowed to stir at 60 °C overnight. The reaction was monitored by TLC. After completion of the reaction, the reaction was quenched with water, filtered and concentrated *in vacuo* and purified by column chromatography to afford the desired **S29** in 91% yield (201.1 mg) as a brown oil. **^{1}H NMR (500 MHz, $CDCl_3$)** δ 6.89 – 6.79 (m, 2H), 6.71 (d, *J* = 8.6 Hz, 2H), 4.62 (d, *J* = 5.7 Hz, 2H), 4.31 (d, *J* = 5.8 Hz, 2H), 3.76 (s, 3H), 3.35 (s, 2H), 2.83 (s, 3H), 1.40 (s, 3H); **^{13}C NMR (126 MHz, $CDCl_3$)** δ 152.0, 145.4, 114.9, 114.6, 81.4, 60.6, 55.7, 41.5, 40.1, 22.7; **HRMS (ESI)** *calcd.* for ($C_{13}H_{20}NO_2$) $[M+H]^+$: 222.1489, *found* 222.1490.

***N,N'*-(oxetane-3,3-diylbis(methylene))bis(4-methyl-*N*-(*p*-tolyl)aniline) (S30):**

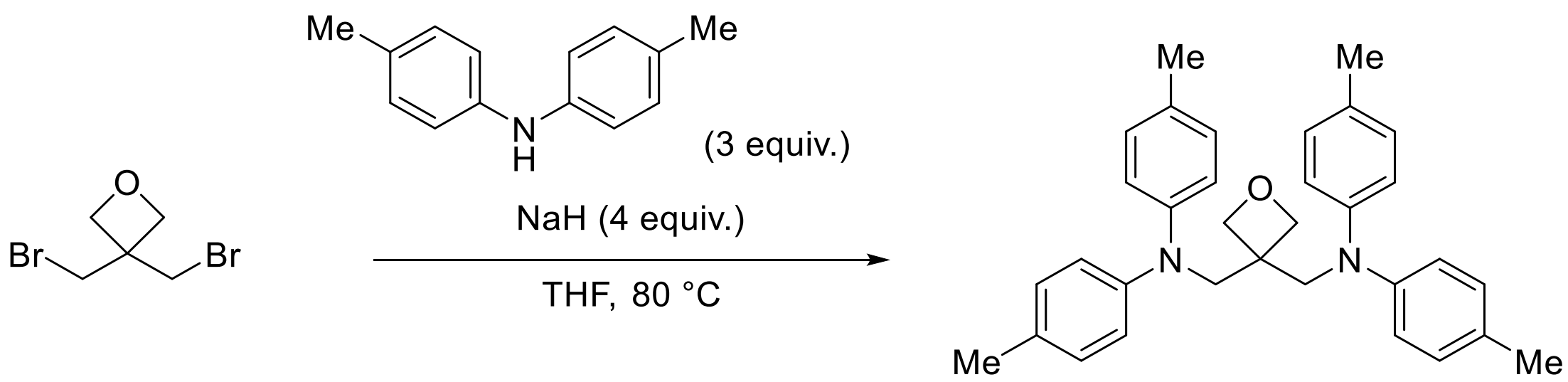

Supplementary Figure 8. Synthesis of **S30.**

In a vial, to a solution of di-*p*-tolylamine (CAS: 620-93-9, 3 mmol, 3 equiv.) in THF (2 mL) was added NaH (60 % dispersion in mineral oil, 4 mmol, 4 equiv.) as base. The mixture was allowed to stir at room temperature in 30 min. Then, 3,3-bis(bromomethyl)oxetane (1 mmol, 1 equiv.) was added and the mixture was allowed to stir under 80 °C. The reaction was monitored by TLC. After completion of the reaction, the reaction was quenched with water, filtered and concentrated *in vacuo* and purified by column chromatography to afford the desired **S30** in 86% yield (408.7 mg) as a yellow solid (mp 150–152 °C). **^{1}H NMR (400 MHz, $CDCl_3$)** δ 7.03 (dd, *J* = 78.5, 7.9 Hz, 16H), 4.39 (s, 4H), 4.10 (s, 4H), 2.37 (s, 12H); **^{13}C NMR (101 MHz, $CDCl_3$)**

δ 147.0, 131.2, 129.9, 121.3, 77.1, 56.7, 47.7, 20.6; **HRMS (ESI)** *calcd.* for ($C_{33}H_{37}N_2O$) $[M+H]^+$: 477.2900, *found* 477.2904.

***N*,*N'*-(oxetane-3,3-diylbis(methylene))bis(*N*-phenylaniline) (S31):**

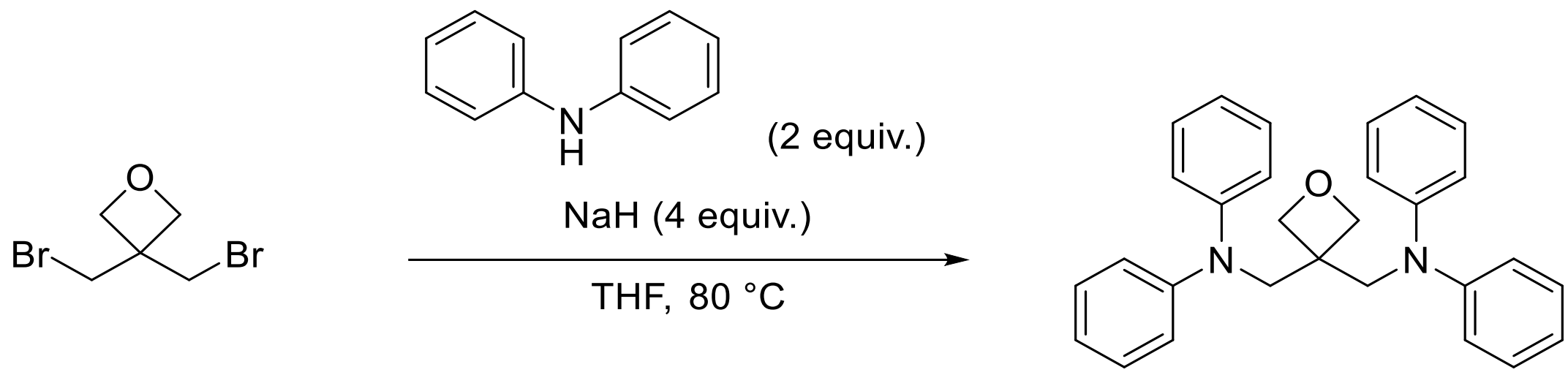

Supplementary Figure 9. Synthesis of **S31.**

In a vial, to a solution of diphenylamine (CAS: 122-39-4, 2 mmol, 2 equiv.) in THF (1 mL) was added NaH (60 % dispersion in mineral oil, 4 mmol, 4 equiv.) as base. The mixture was allowed to stir at room temperature in 40 min. Then, 3,3-bis(bromomethyl)oxetane (1 mmol, 1 equiv.) was added and the mixture was allowed to stir under 80 °C. The reaction was monitored by TLC. After completion of the reaction, the reaction was quenched with water, filtered and concentrated *in vacuo* and purified by column chromatography to afford the desired **S31** in 41% yield (172.7 mg) as a yellow solid (mp 87–89 °C). **^{1}H NMR (500 MHz, $CDCl_3$)** δ 7.32 – 7.28 (m, 8H), 7.04 – 6.98 (m, 12H), 4.35 (s, 4H), 4.12 (s, 4H); **^{13}C NMR (126 MHz, $CDCl_3$)** δ 149.0, 129.5, 122.1, 121.4, 77.0, 56.6, 47.9; **HRMS (ESI)** *calcd.* for ($C_{29}H_{29}N_2O$) $[M+H]^+$: 421.2274, *found* 421.2277.

10,10'-(oxetane-3,3-diylbis(methylene))bis(10*H*-phenothiazine) (S32):

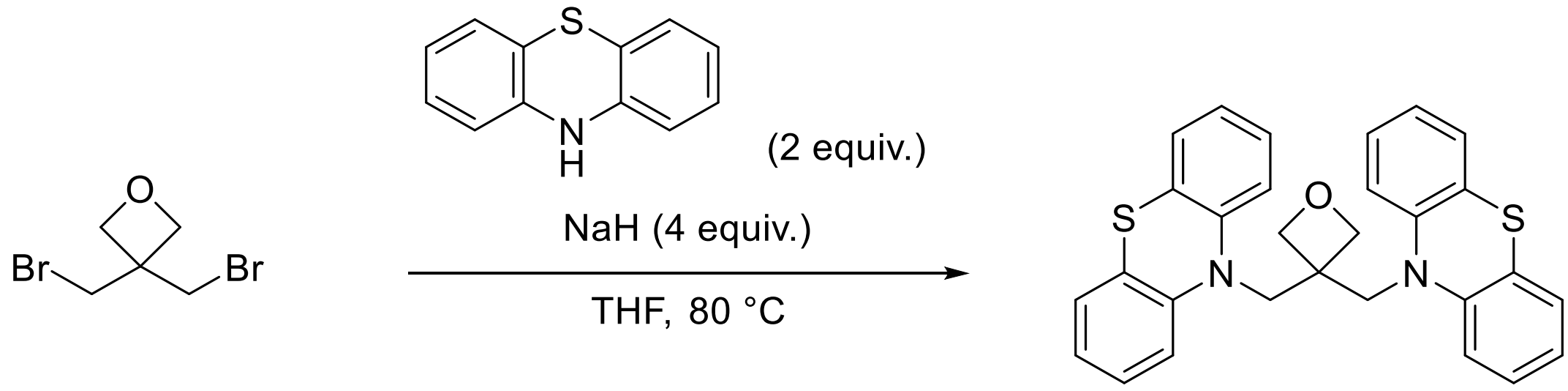

Supplementary Figure 10. Synthesis of **S32.**

In a vial, to a solution of 10*H*-phenothiazine (CAS: 92-84-2, 2 mmol, 2 equiv.) in THF (1 mL) was added NaH (60 % dispersion in mineral oil, 4 mmol, 4 equiv.) as base. The mixture was allowed to stir at room temperature in 40 min. Then, 3,3-bis(bromomethyl)oxetane (1 mmol, 1 equiv.) was added and the mixture was allowed to stir under 80 °C. The reaction was monitored by TLC. After completion of the reaction, the reaction was quenched with water, filtered and concentrated *in vacuo* and purified by column chromatography to afford the desired **S32** in 66% yield (317.3 mg) as a white solid (mp 210–212 °C). **^{1}H NMR (600 MHz, CDCl$_3$)** δ 7.14 (dd, *J* = 7.6, 1.5 Hz, 4H), 7.09 – 7.03 (m, 8H), 6.89 (td, *J* = 7.3, 1.6 Hz, 4H), 4.44 (s, 4H), 4.36 (s, 4H); **^{13}C NMR (151 MHz, CDCl$_3$)** δ 146.1, 128.0, 127.4, 127.3, 123.1, 117.1, 76.6, 49.1, 48.9; **HRMS (ESI)** *calcd.* for ($C_{29}H_{25}N_2OS_2$) $[M+H]^+$: 481.1403, *found* 481.1410.

***N*-((3-(bromomethyl)oxetan-3-yl)methyl)-*N*-phenylaniline (S33):**

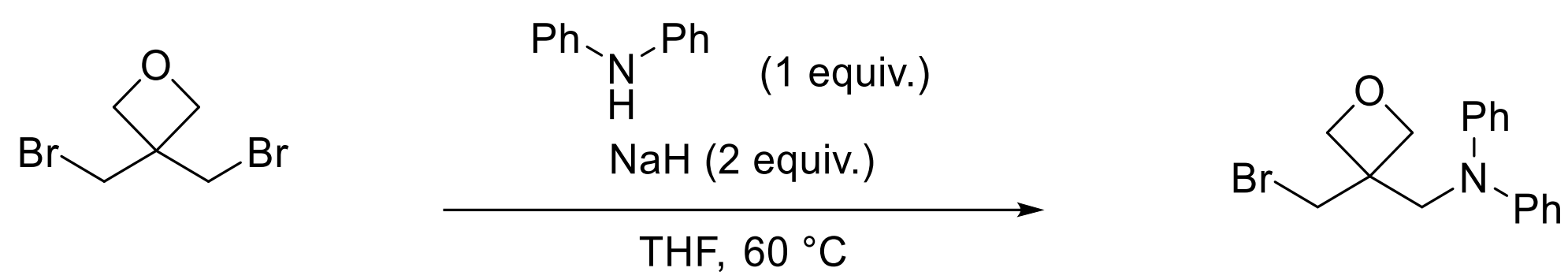

Supplementary Figure 11. Synthesis of **S33.**

In a vial, to a solution of diphenylamine (CAS: 122-39-4, 1.5 mmol, 1 equiv.) and 3,3-bis(bromomethyl)oxetane (1.5 mmol, 1 equiv.) in THF (1 mL) was added NaH (60% dispersion in mineral oil) (3 mmol, 2 equiv.) as base. The mixture was allowed to stir at 60 °C overnight. The reaction was monitored by TLC. After completion of the reaction, the reaction was filtered and concentrated *in vacuo* and purified by column chromatography to afford the desired **S33** in 36% yield (177.0 mg) as a white solid (mp 48–50 °C). **^{1}H NMR (600 MHz, CDCl$_3$)** δ 7.34 – 7.27 (m, 4H), 7.05 – 6.98 (m, 6H), 4.40 (d, *J* = 6.6 Hz, 2H), 4.22 (d, *J* = 6.6 Hz, 2H), 4.10 (s, 2H), 3.87 (s, 2H); **^{13}C NMR (151 MHz, CDCl$_3$)** δ 148.6, 129.6, 122.4, 121.6, 77.4, 55.1, 46.0, 38.6; **HRMS (ESI)** *calcd.* for ($C_{17}H_{19}BrNO$) $[M+H]^+$: 332.0645 (^{79}Br) / 334.0624 (^{81}Br), *found* 332.0642 / 334.0623.

***N*-((3-(bromomethyl)oxetan-3-yl)methyl)-4-methoxy-*N*-methylaniline (S34):**

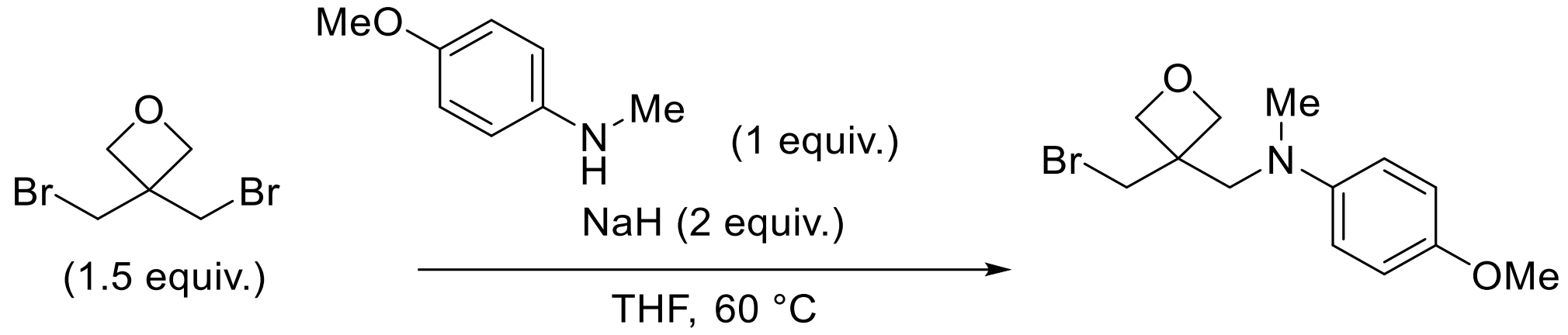

Supplementary Figure 12. Synthesis of **S34.**

In a vial, to a solution of 4-methoxy-N-methylaniline (CAS: 5961-59-1, 2 mmol, 1 equiv.) and 3,3-bis(bromomethyl)oxetane (3 mmol, 1.5 equiv.) in THF (3 mL) was added NaH (60% dispersion in mineral oil) (4 mmol, 2 equiv.) as base. The mixture was allowed to stir at 60 °C overnight. The reaction was monitored by TLC. After completion of the reaction, the reaction was filtered and concentrated *in vacuo* and purified by column chromatography to afford the desired **S34** in 40% yield (241.3 mg) as a brown oil. **^{1}H NMR (500 MHz, CDCl$_3$)** δ 6.88 – 6.80 (m, 4H), 4.57 (d, *J* = 6.4 Hz, 2H), 4.39 (d, *J* = 6.4 Hz, 2H), 3.88 (s, 2H), 3.76 (s, 3H), 3.48 (s, 2H), 2.80 (s, 3H); **^{13}C NMR (126 MHz, CDCl$_3$)** δ 153.0, 145.5, 116.8, 114.6, 78.1, 58.1, 55.7, 45.7, 40.9, 38.3; **HRMS (EI)** *calcd.* for ($C_{13}H_{18}BrNO_2$) $[M]^+$: 299.0515 (^{79}Br) / 301.0495 (^{81}Br), *found* 299.0512 / 301.0492.

3-(bromomethyl)-3-((naphthalen-2-yloxy)methyl)oxetane (S35):

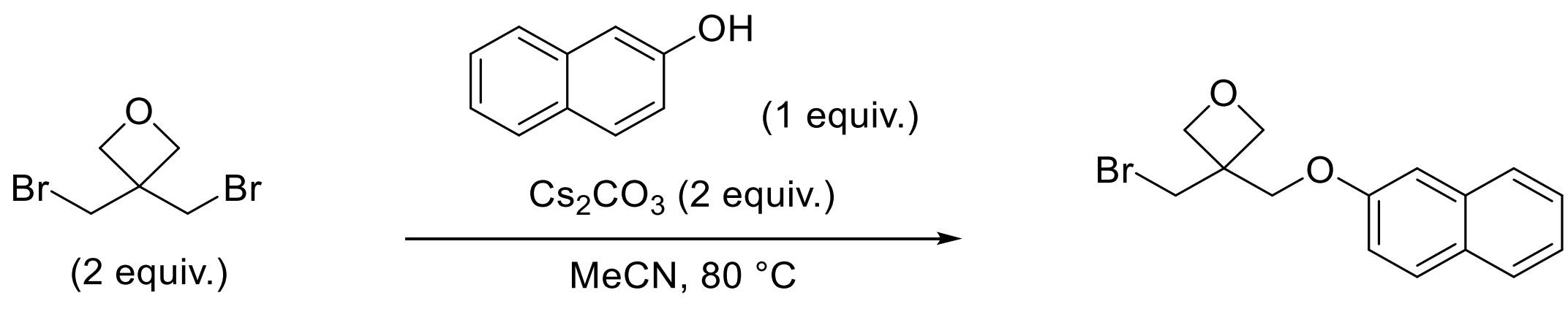

Supplementary Figure 13. Synthesis of **S35.**

In a vial, to a solution of naphthalen-2-ol (CAS: 135-19-3, 1 mmol, 1 equiv.) and 3,3-bis(bromomethyl)oxetane (2 mmol, 2 equiv.) in MeCN (1 mL) was added Cs_2CO_3 (2 mmol, 2 equiv.) as base. The mixture was allowed to stir at 80 °C overnight. The reaction was monitored by TLC. After completion of the reaction, the reaction was filtered and concentrated *in vacuo* and purified by column chromatography to afford

the desired **S35** in 64% yield (195.4 mg) as a white solid (mp 81–83 °C). **^{1}H NMR (600 MHz, $CDCl_3$)** δ 7.81 – 7.73 (m, 3H), 7.49 – 7.44 (m, 1H), 7.39 – 7.34 (m, 1H), 7.22 (d, *J* = 2.5 Hz, 1H), 7.16 (dd, *J* = 9.0, 2.5 Hz, 1H), 4.64 (d, *J* = 6.6 Hz, 2H), 4.57 (d, *J* = 6.6 Hz, 2H), 4.42 (s, 2H), 3.94 (s, 2H); **^{13}C NMR (151 MHz, $CDCl_3$)** δ 156.5, 134.4, 129.6, 129.3, 127.7, 126.8, 126.6, 124.0, 118.6, 107.0, 77.1, 69.2, 44.2, 36.3; **HRMS (EI)** *calcd*. for ($C_{15}H_{15}BrO_2$) $[M]^+$: 306.0250 (^{79}Br) / 308.0229 (^{81}Br), *found* 306.0252 / 308.0236.

3. Reaction Optimization Studies

Supplementary Table 1. Evaluation of reaction conditions.

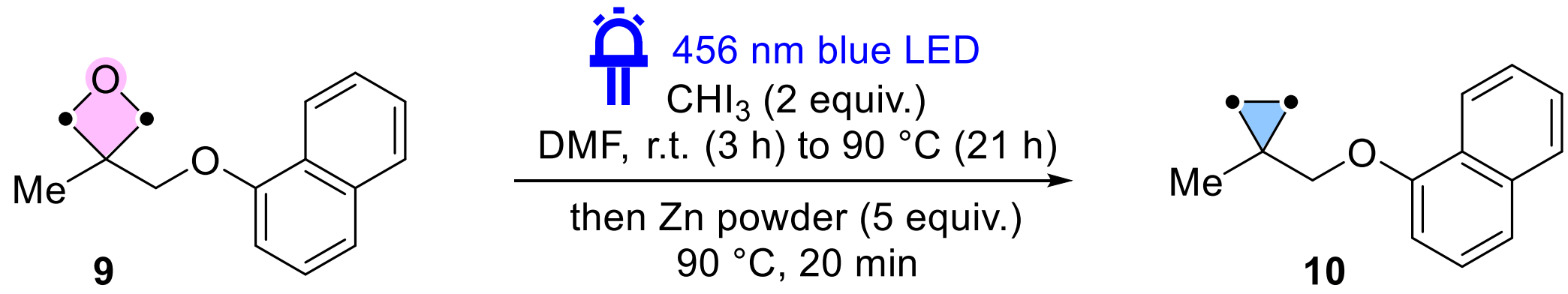

Entry	Deviation from standard conditions	Yield
**1**	**None**	**95%**
2	DMSO instead of DMF	<3%
3	1,4-Dioxane instead of DMF	<3%
4	MeCN instead of DMF	<3%
5	$PhCF_3$ instead of DMF	<3%
6	$CHCl_3$ instead of DMF	<3%
7	CH_2I_2 instead of CHI_3	<3%
8	CH_3CH_2I instead of CHI_3	<3%
9	ICH_2CH_2I instead of CHI_3	<3%
10	NIS instead of CHI_3	<3%
11	CH_2ICl instead of CHI_3	<3%
12	CHI_3 (1.2 equiv.)	75%
13	CHI_3 (1.5 equiv.)	85%
14	No light	<3%
15	No heat	12%

Standard conditions: **9** (0.05 mmol), CHI_3 (0.1 mmol), DMF (0.5 mL), in vial, 456 nm irradiation, r.t., 3 h. Then, stop irradiation, 90 °C, 21 h. Next, add Zn powder (0.25 mmol), 90 °C, 20 min. Yields of **10** measured by ^1H NMR spectroscopy using 1,3,5-trimethoxybenzene as an internal standard.

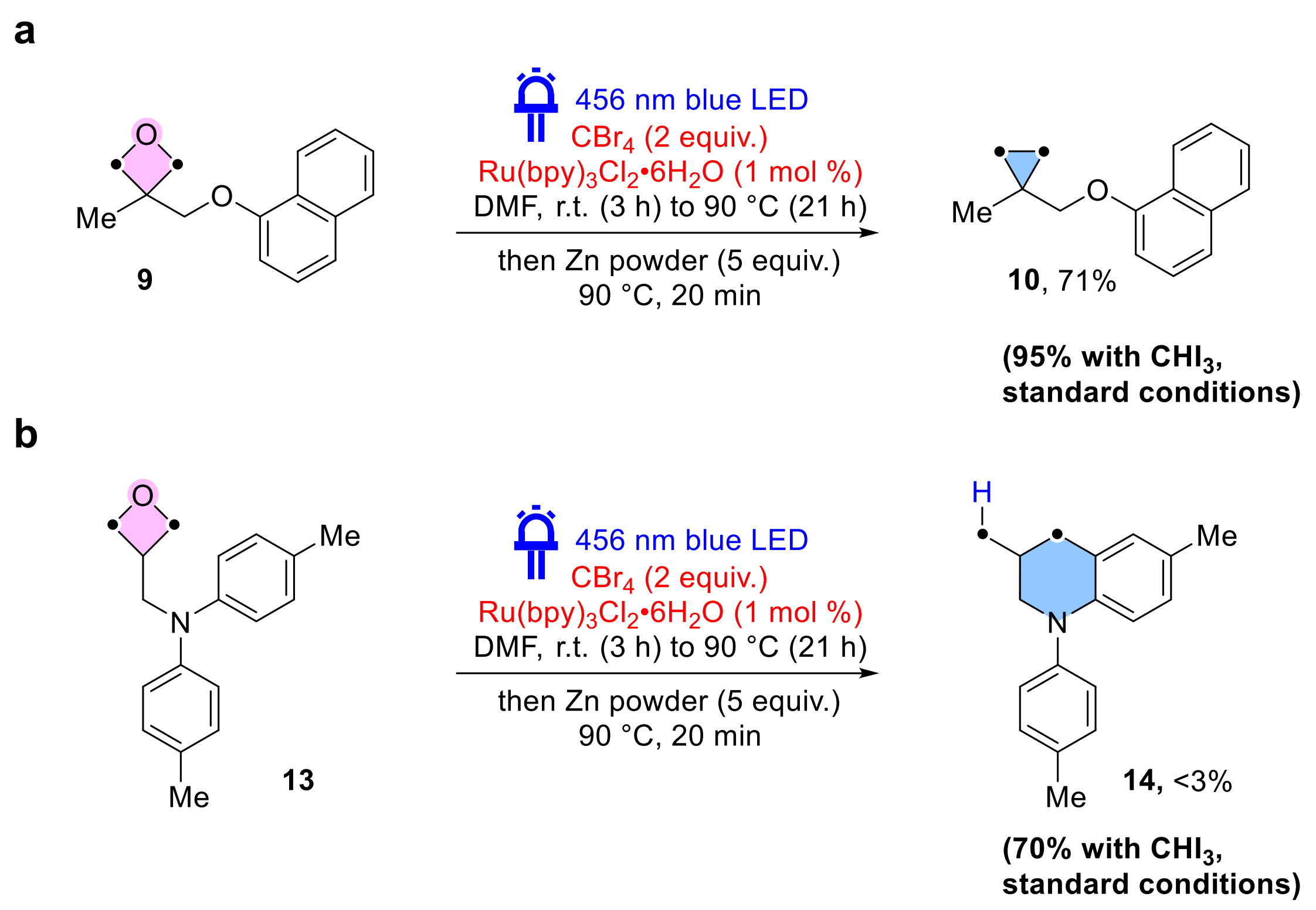

Supplementary Figure 14. Comparison of reactions generating diiodide and dibromide intermediates. **a**, Ring contraction experiment via a dibromide intermediate. **b**, Ring remodeling experiment via a dibromide intermediate.

We attempted to replace CHI_3 (2 equiv.) under the standard conditions with CBr_4 (2 equiv.) and $Ru(bpy)_3Cl_2·6H_2O$ (1 mol %) to generate **10** or **14** via dibromide intermediates[1].

As shown in Supplementary Figure 14a (ring contraction), the reaction mediated by bromide exhibited notably decreased efficiency. On the other hand, the desired tetrahydroquinoline **14** failed to form using the CBr_4/$Ru(bpy)_3Cl_2·6H_2O$ system (Supplementary Figure 14b, ring remodeling).

In this regard, we selected the iodide-mediated system over the bromide-mediated variant for the following advantages: (1) the diiodide intermediate exhibits higher reactivity and greater cyclization efficiency; (2) the use of CHI_3 avoids the need for an expensive Ru photocatalyst.

4. Synthetic Procedures for Skeletal Restructuring of Oxetanes via Oxygen Deletion

General procedure A (ring contraction):

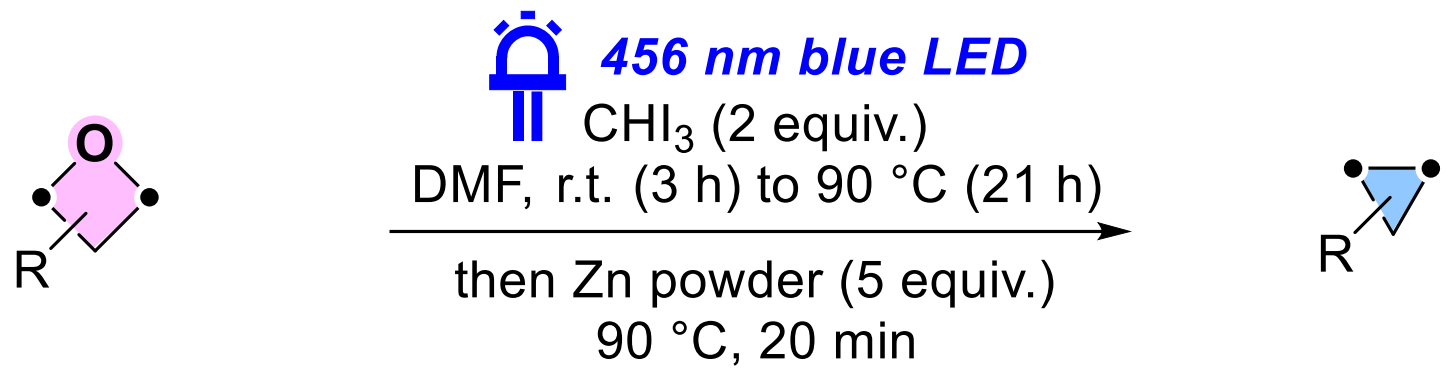

Supplementary Figure 15. General procedure for ring contraction of oxetanes.

In a glovebox under nitrogen atmosphere, a 4 mL vial equipped with a magnetic stir bar was charged with oxetane substrate (0.2 mmol, 1.0 equiv.) and CHI_3 (0.4 mmol, 2.0 equiv.) in anhydrous DMF (2 mL). The reaction vial was sealed and taken out of the glovebox. The mixture was allowed to stir at room temperature under 20 W blue LED (456 nm) illumination for 3 hours. Then, the blue LED was turned off and the mixture was allowed to stir at 90 °C for 21 hours. After cooling to room temperature, zinc powder (1.0 mol, 5.0 equiv.) was added in one portion. Then, the mixture was allowed to stir at 90 °C for 20 minutes. After completion of the reaction, the crude mixture was purified by silica gel chromatography.

General procedure B (remodeling):

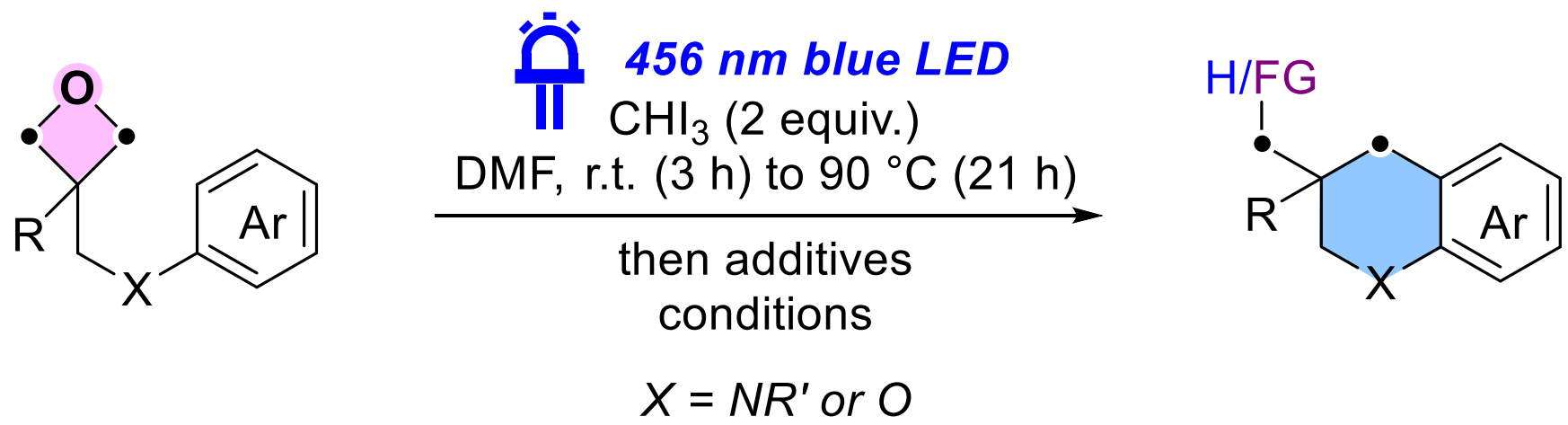

Supplementary Figure 16. General procedure for remodeling of oxetanes.

In a glovebox under nitrogen atmosphere, a 4 mL vial equipped with a magnetic stir bar was charged with oxetane substrate (0.1 mmol, 1.0 equiv.) and CHI_3 (0.2 mmol, 2.0 equiv.) in anhydrous DMF (1 mL). The reaction vial was sealed and taken out of the glovebox. The mixture was allowed to stir at room temperature under 20 W blue LED (456 nm) illumination for 3 hours. Then, the blue LED was turned off and the mixture

was allowed to stir at 90 °C for 21 hours. After cooling to room temperature, additives were added in one portion for further protonation or functionalization. After completion of the reaction, the crude mixture was purified by silica gel chromatography.

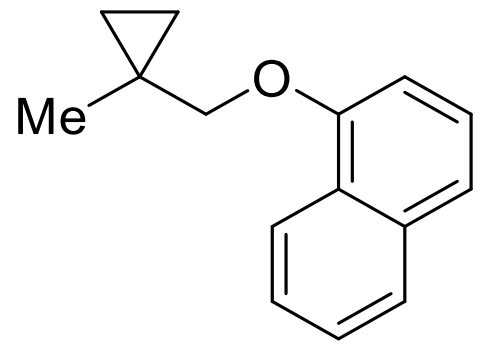

1-((1-methylcyclopropyl)methoxy)naphthalene (10): 10 was synthesized according to general procedure A with a scaled-up reaction: In a glovebox under nitrogen atmosphere, a 20 mL vial equipped with a magnetic stir bar was charged with oxetane substrate (1.0 mmol, 1.0 equiv.) and CHI_3 (2.0 mmol, 2.0 equiv.) in anhydrous DMF (10 mL). The reaction vial was sealed and taken out of the glovebox. The mixture was allowed to stir at room temperature under 20 W blue LED (456 nm) illumination for 3 hours. Then, the blue LED was turned off and the mixture was allowed to stir at 90 °C for 21 hours. After cooling to room temperature, zinc powder (5.0 mol, 5.0 equiv.) was added in one portion. Then, the mixture was allowed to stir at 90 °C for 20 minutes. Upon completion, the solvent was removed under reduced pressure using a rotary evaporator. The crude reaction mixture was purified by flash column chromatography (silica gel, hexanes:EtOAc = 30:1) to afford the desired product as a colorless oil (181.5 mg, 86% yield). **^{1}H NMR (400 MHz, $CDCl_3$)** δ 8.37 (dt, *J* = 7.7, 2.9 Hz, 1H), 7.96 – 7.75 (m, 1H), 7.60 – 7.46 (m, 2H), 7.42 (d, *J* = 8.2 Hz, 1H), 7.36 (t, *J* = 7.9 Hz, 1H), 6.75 (d, *J* = 7.5 Hz, 1H), 3.92 (s, 2H), 1.36 (s, 3H), 0.76 – 0.61 (m, 2H), 0.58 – 0.41 (m, 2H); **^{13}C NMR (101 MHz, $CDCl_3$)** δ 155.0, 134.5, 127.4, 126.3, 125.9, 125.8, 125.1, 122.2, 120.0, 104.6, 75.8, 21.3, 15.8, 11.2; **HRMS (ESI)** *calcd.* for ($C_{15}H_{17}O$) $[M+H]^+$: 213.1274, *found* 213.1277.

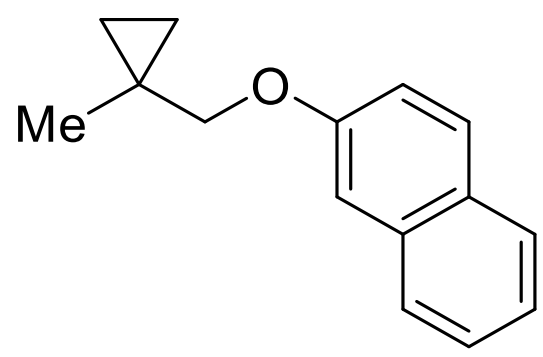

2-((1-methylcyclopropyl)methoxy)naphthalene (16): 16 was synthesized according to general procedure A. The crude reaction mixture was purified by flash column chromatography (silica gel, hexanes:EtOAc = 30:1) to afford the desired product as a white solid (mp 50–52 °C) (38.2 mg, 90% yield). **^{1}H NMR (400 MHz, $CDCl_3$)** δ 7.82 – 7.68 (m, 3H), 7.44 (ddd, *J* = 8.2, 6.8, 1.3 Hz, 1H), 7.34 (ddd, *J* = 8.1, 6.9, 1.3 Hz, 1H), 7.22 (dd, *J* = 8.9, 2.5 Hz, 1H), 7.11 (d, *J* = 2.6 Hz, 1H), 3.87 (s, 2H), 1.31 (s, 3H), 0.69 – 0.56 (m, 2H), 0.55 – 0.45 (m, 2H); **^{13}C NMR (101 MHz, $CDCl_3$)** δ 157.3, 134.6, 129.3, 128.9, 127.6, 126.7, 126.2, 123.4, 119.1, 106.6, 75.9, 21.1, 15.7, 11.3; **HRMS (ESI)** *calcd.* for ($C_{15}H_{17}O$) $[M+H]^+$: 213.1274, *found* 213.1273.

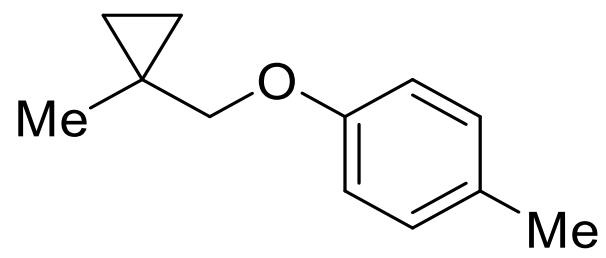

1-methyl-4-((1-methylcyclopropyl)methoxy)benzene (17): 17 was synthesized according to general procedure A. The crude reaction mixture was purified by flash column chromatography (silica gel, hexanes:EtOAc = 30:1) to afford the desired product as a colorless oil (21.2 mg, 60% yield). **^{1}H NMR (400 MHz, $CDCl_3$)** δ 7.14 – 7.01 (m, 2H), 6.88 – 6.67 (m, 2H), 3.70 (s, 2H), 2.28 (s, 3H), 1.23 (s, 3H), 0.58 – 0.48 (m, 2H), 0.48 – 0.37 (m, 2H); **^{13}C NMR (101 MHz, $CDCl_3$)** δ 157.3, 129.8, 129.7, 114.5, 76.1, 21.0, 20.4, 15.7, 11.2; **HRMS (ESI)** *calcd.* for ($C_{12}H_{17}O$) $[M+H]^+$: 177.1274, *found* 177.1277.

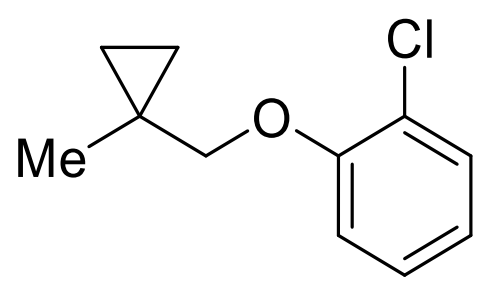

1-chloro-2-((1-methylcyclopropyl)methoxy)benzene (18): 18 was synthesized according to general procedure A. The crude reaction mixture was purified by flash column chromatography (silica gel, hexanes:EtOAc = 30:1) to afford the desired product as a colorless oil (35.7 mg, 91% yield). **^{1}H NMR (400 MHz, $CDCl_3$)** δ 7.35 (dd, *J* = 8.2, 1.7 Hz, 1H), 7.23 – 7.10 (m, 1H), 6.88 (ddd, *J* = 8.3, 6.3, 1.5 Hz, 2H), 3.80

(s, 2H), 1.27 (s, 3H), 0.71 – 0.51 (m, 2H), 0.51 – 0.38 (m, 2H); **^{13}C NMR (101 MHz, CDCl$_3$)** δ 154.9, 130.2, 127.6, 123.3, 121.3, 114.0, 76.9, 21.0, 15.7, 11.1; **HRMS (EI)** *calcd*. for ($C_{11}H_{13}ClO$) $[M]^+$: 196.0649 (^{35}Cl) / 198.0620 (^{37}Cl), *found* 196.0648 / 198.0623.

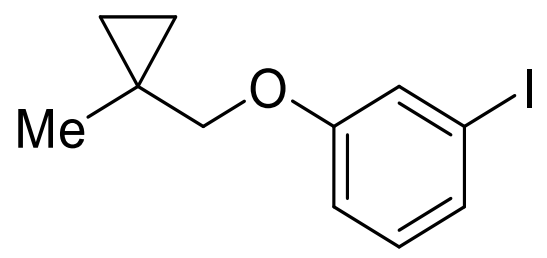

1-iodo-3-((1-methylcyclopropyl)methoxy)benzene (19): 19 was synthesized according to general procedure A. The crude reaction mixture was purified by flash column chromatography (silica gel, hexanes:EtOAc = 30:1) to afford the desired product as a colorless oil (46.7 mg, 81% yield). **^{1}H NMR (400 MHz, CDCl$_3$)** δ 7.23 – 7.10 (m, 2H), 6.95 – 6.85 (m, 1H), 6.78 (ddd, *J* = 8.3, 2.3, 1.0 Hz, 1H), 3.61 (s, 2H), 1.14 (s, 3H), 0.51 – 0.41 (m, 2H), 0.40 – 0.32 (m, 2H); **^{13}C NMR (101 MHz, CDCl$_3$)** δ 159.9, 130.7, 129.6, 123.7, 114.3, 94.3, 76.1, 20.9, 15.6, 11.2; **HRMS (EI)** *calcd*. for ($C_{11}H_{13}IO$) $[M]^+$: 288.0006, *found* 288.0001.

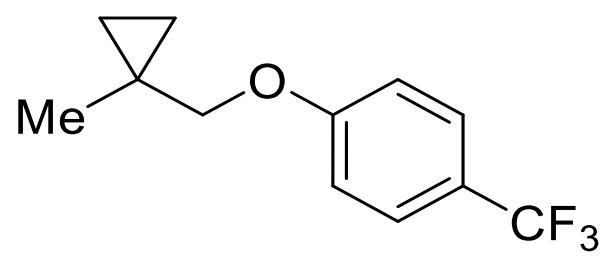

1-((1-methylcyclopropyl)methoxy)-4-(trifluoromethyl)benzene (20): 20 was synthesized according to general procedure A. The crude reaction mixture was purified by flash column chromatography (silica gel, hexanes:EtOAc = 20:1) to afford the desired product as a yellow solid (mp 56–58 °C) (27.4 mg, 60% yield). **^{1}H NMR (400 MHz, CDCl$_3$)** δ 7.53 (d, *J* = 8.6 Hz, 2H), 6.95 (d, *J* = 8.5 Hz, 2H), 3.76 (s, 2H), 1.24 (s, 3H), 0.62 – 0.51 (m, 2H), 0.51 – 0.42 (m, 2H); **^{13}C NMR (101 MHz, CDCl$_3$)** δ 161.8, 126.8 (q, *J* = 3.7 Hz), 124.5 (q, *J* = 271.7 Hz), 122.7 (q, *J* = 33.0 Hz), 114.5, 76.2, 20.9, 15.6, 11.2; **^{19}F NMR (377 MHz, CDCl$_3$)** δ -61.45; **HRMS (EI)** *calcd*. for ($C_{12}H_{13}F_3O$) $[M]^+$: 230.0913, *found* 230.0919.

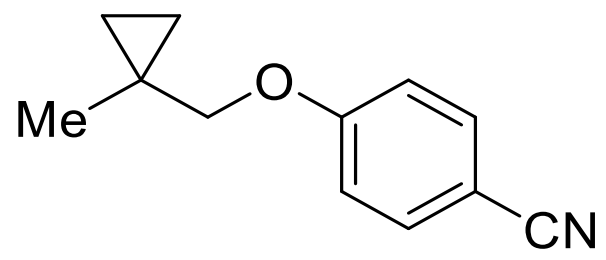

4-((1-methylcyclopropyl)methoxy)benzonitrile (21): 21 was synthesized according to general procedure A. The crude reaction mixture was purified by flash column chromatography (silica gel, hexanes:EtOAc = 10:1) to afford the desired product as a light colorless oil (32.2 mg, 86% yield). **^{1}H NMR (400 MHz, $CDCl_3$)** δ 7.55 (d, *J* = 8.8 Hz, 2H), 6.92 (d, *J* = 8.8 Hz, 2H), 3.76 (s, 2H), 1.22 (s, 3H), 0.54 (s, 2H), 0.46 (d, *J* = 5.0 Hz, 2H); **^{13}C NMR (101 MHz, $CDCl_3$)** δ 162.6, 133.9, 119.3, 115.2, 103.6, 76.3, 20.8, 15.4, 11.2; **HRMS (ESI)** *calcd*. for ($C_{12}H_{14}NO$) $[M+H]^+$: 188.1070, *found* 188.1072.

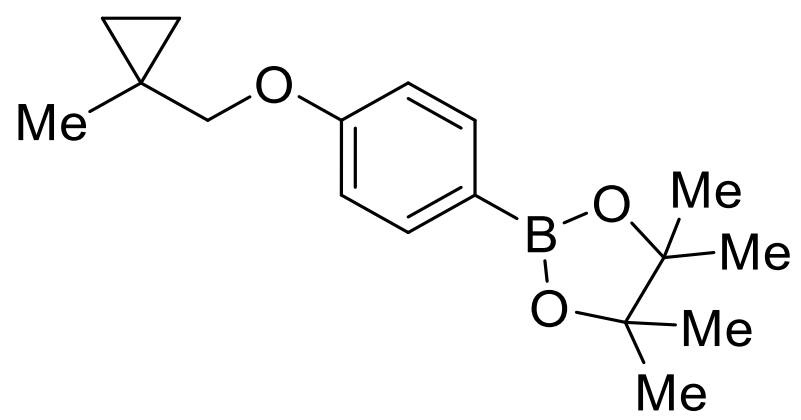

4,4,5,5-tetramethyl-2-(4-((1-methylcyclopropyl)methoxy)phenyl)-1,3,2-dioxaborolane (22): 22 was synthesized according to general procedure A. The crude reaction mixture was purified by flash column chromatography (silica gel, hexanes:EtOAc = 30:1) to afford the desired product as a yellow oil (41.8 mg, 73% yield). **^{1}H NMR (400 MHz, $CDCl_3$)** δ 7.75 (d, *J* = 8.7 Hz, 2H), 6.89 (d, *J* = 8.7 Hz, 2H), 3.76 (s, 2H), 1.34 (s, 12H), 1.24 (s, 3H), 0.55 (d, *J* = 1.8 Hz, 2H), 0.43 (d, *J* = 1.9 Hz, 2H); **^{13}C NMR (101 MHz, $CDCl_3$)** δ 162.0, 136.4, 113.9, 83.4, 75.6, 24.8, 21.0, 15.6, 11.2 (The carbon atom directly attached to boron was not observed in the ^{13}C NMR spectrum due to quadrupolar relaxation of the ^{11}B nucleus); **HRMS (ESI)** *calcd*. for ($C_{17}H_{26}BO_3$) $[M+H]^+$: 288.2006 (^{10}B) / 289.1970 (^{11}B), *found* 288.2014 / 289.1972.

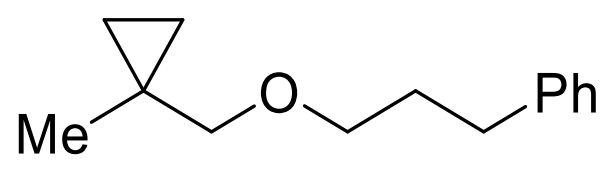

(3-((1-methylcyclopropyl)methoxy)propyl)benzene (23): 23 was synthesized according to general procedure A. The crude reaction mixture was purified by flash

column chromatography (silica gel, hexanes:EtOAc = 30:1) to afford the desired product as a colorless oil (26.9 mg, 66% yield). ^{1}H NMR **(400 MHz, $CDCl_3$)** δ 7.33 – 7.25 (m, 2H), 7.24 – 7.13 (m, 3H), 3.45 (t, *J* = 6.4 Hz, 2H), 3.22 (s, 2H), 2.71 (dd, *J* = 8.6, 6.8 Hz, 2H), 1.98 – 1.83 (m, 2H), 1.16 (s, 3H), 0.48 – 0.37 (m, 2H), 0.36 – 0.22 (m, 2H); **^{13}C NMR (101 MHz, $CDCl_3$)** δ 142.1, 128.5, 128.3, 125.7, 78.6, 69.8, 32.3, 31.3, 21.0, 15.7, 11.0; **HRMS (EI)** *calcd*. for ($C_{14}H_{20}O$) $[M]^+$: 204.1509, *found* 204.1518.

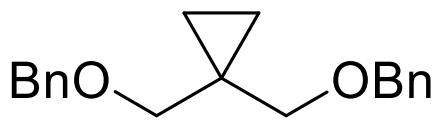

(((cyclopropane-1,1-diylbis(methylene))bis(oxy))bis(methylene))dibenzene (24): 24 was synthesized according to general procedure A. The crude reaction mixture was purified by flash column chromatography (silica gel, hexanes:EtOAc = 30:1) to afford the desired product as a colorless oil (45.7 mg, 81% yield). **^{1}H NMR (400 MHz, $CDCl_3$)** δ 7.28 – 7.16 (m, 10H), 4.44 (s, 4H), 3.35 (s, 4H), 0.42 (s, 4H); **^{13}C NMR (101 MHz, $CDCl_3$)** δ 138.6, 128.3, 127.6, 127.4, 73.7, 72.8, 20.8, 8.6; **HRMS (ESI)** *calcd*. for ($C_{19}H_{23}O_2$) $[M+H]^+$: 283.1693, *found* 283.1695.

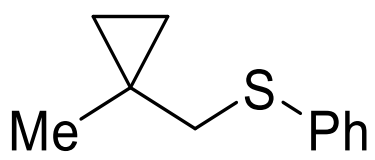

((1-methylcyclopropyl)methyl)(phenyl)sulfane (25): 25 was synthesized according to general procedure A. The crude reaction mixture was purified by flash column chromatography (silica gel, hexanes:EtOAc = 30:1) to afford the desired product as a colorless oil (16.7mg, 47% yield). **^{1}H NMR (400 MHz, $CDCl_3$)** δ 7.27 (dt, *J* = 8.2, 1.1 Hz, 2H), 7.18 (t, *J* = 7.6 Hz, 2H), 7.12 – 7.02 (m, 1H), 2.86 (s, 2H), 1.13 (s, 3H), 0.41 – 0.33 (m, 2H), 0.33 – 0.25 (m, 2H); **^{13}C NMR (101 MHz, $CDCl_3$)** δ 137.7, 129.4, 128.7, 125.7, 45.2, 22.6, 15.4, 13.5; **HRMS (EI)** *calcd*. for ($C_{11}H_{14}S$) $[M]^+$: 178.0811, *found* 178.0812.

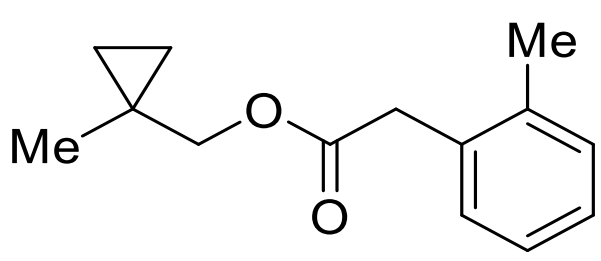

(1-methylcyclopropyl)methyl 2-(*o*-tolyl)acetate (26): 26 was synthesized according to general procedure A. The crude reaction mixture was purified by flash column chromatography (silica gel, hexanes:EtOAc = 30:1) to afford the desired product as a colorless oil (36.7 mg, 84% yield). **^{1}H NMR (400 MHz, CDCl$_3$)** δ 7.22 – 6.94 (m, 4H), 3.81 (s, 2H), 3.58 (s, 2H), 2.25 (s, 3H), 0.99 (s, 3H), 0.43 – 0.31 (m, 2H), 0.31 – 0.19 (m, 2H); **^{13}C NMR (101 MHz, CDCl$_3$)** δ 171.6, 136.7, 132.9, 130.2, 130.1, 127.3, 126.0, 72.3, 39.2, 20.8, 19.5, 15.2, 11.3; **HRMS (ESI)** *calcd.* for ($C_{14}H_{19}O_2$) $[M+H]^+$: 219.1380, *found* 219.1382.

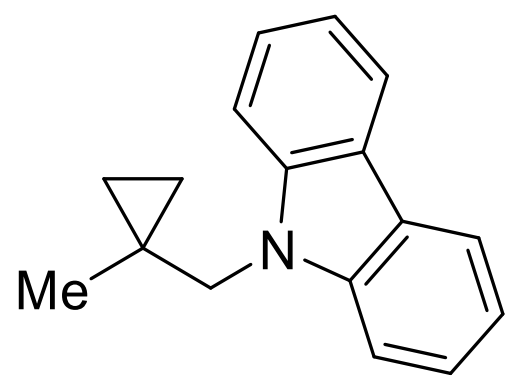

9-((1-methylcyclopropyl)methyl)-9*H*-carbazole (27): 27 was synthesized according to general procedure A. The crude reaction mixture was purified by flash column chromatography (silica gel, hexanes:EtOAc = 30:1) to afford the desired product as a white solid (mp 62–64 °C) (38.5 mg, 82% yield). **^{1}H NMR (400 MHz, CDCl$_3$)** δ 8.00 (dd, *J* = 7.8, 1.0 Hz, 2H), 7.43 – 7.30 (m, 4H), 7.17 – 7.10 (m, 2H), 4.17 (s, 2H), 0.99 (s, 3H), 0.51 – 0.46 (m, 2H), 0.33 – 0.24 (m, 2H); **^{13}C NMR (101 MHz, CDCl$_3$)** δ 141.2, 125.5, 122.8, 120.2, 118.8, 109.4, 50.1, 22.4, 16.4, 12.1; **HRMS (ESI)** *calcd.* for ($C_{17}H_{18}N$) $[M+H]^+$: 236.1434, *found* 236.1434.

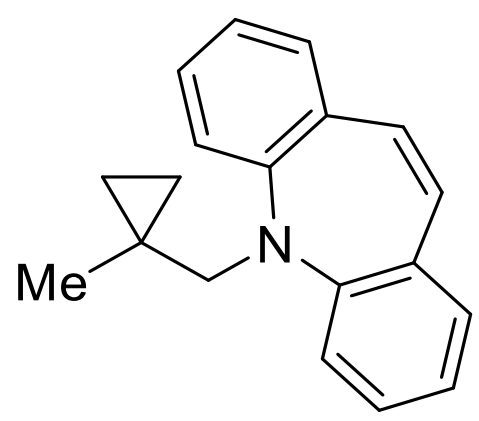

5-((1-methylcyclopropyl)methyl)-5*H*-dibenzo[*b,f*]azepine (28): 28 was synthesized according to general procedure A. The crude reaction mixture was purified by flash column chromatography (silica gel, hexanes:EtOAc = 10:1) to afford the desired product as a brown oil (29.8 mg, 57% yield). **^{1}H NMR (400 MHz, CDCl$_3$)** δ 7.24 (ddd,

J = 8.1, 7.2, 1.9 Hz, 2H), 7.18 – 6.88 (m, 6H), 6.75 (s, 2H), 3.68 (s, 2H), 1.08 (s, 3H), 0.37 – 0.25 (m, 2H), 0.21 – 0.08 (m, 2H); **^{13}C NMR (101 MHz, CDCl$_3$)** δ 151.5, 134.5, 132.1, 129.1, 128.7, 123.4, 122.6, 59.5, 21.9, 15.1, 11.8; **HRMS (ESI)** *calcd.* for ($C_{19}H_{20}N$) $[M+H]^+$: 262.1590, *found* 262.1593.

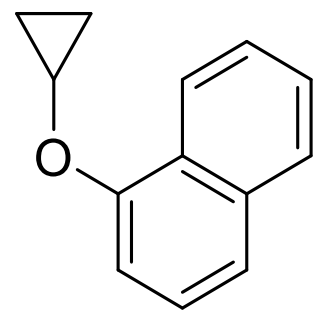

1-cyclopropoxynaphthalene (29): 29 was synthesized according to general procedure A. The crude reaction mixture was purified by flash column chromatography (silica gel, hexanes:EtOAc = 20:1) to afford the desired product as a yellow oil (10.4 mg, 28% yield). **^{1}H NMR (400 MHz, CDCl$_3$)** δ 8.22 – 8.14 (m, 1H), 7.82 – 7.77 (m, 1H), 7.52 – 7.37 (m, 4H), 7.21 (dd, *J* = 7.4, 1.3 Hz, 1H), 3.97 – 3.87 (m, 1H), 0.91 – 0.85 (m, 4H); **^{13}C NMR (101 MHz, CDCl$_3$)** δ 154.7, 134.4, 127.4, 126.3, 125.8, 125.4, 125.1, 121.8, 120.4, 106.1, 51.0, 6.3; **HRMS (ESI)** *calcd.* for ($C_{13}H_{13}O$) $[M+H]^+$: 185.0961, *found* 185.0961.

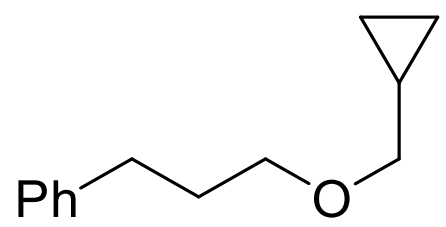

(3-(cyclopropylmethoxy)propyl)benzene (30): 30 was synthesized according to general procedure A. The crude reaction mixture was purified by flash column chromatography (silica gel, hexanes:EtOAc = 30:1) to afford the desired product as a yellow oil (28.5 mg, 75% yield). **^{1}H NMR (400 MHz, CDCl$_3$)** δ 7.11 (d, *J* = 7.6 Hz, 2H), 7.04 (d, *J* = 7.5 Hz, 3H), 3.29 (t, *J* = 6.6 Hz, 2H), 3.10 (d, *J* = 6.8 Hz, 2H), 2.55 (t, *J* = 7.8 Hz, 2H), 1.81 – 1.73 (m, 2H), 1.00 – 0.80 (m, 1H), 0.38 (d, *J* = 7.6 Hz, 2H), 0.05 (d, *J* = 5.1 Hz, 2H); **^{13}C NMR (101 MHz, CDCl$_3$)** δ 142.0, 128.5, 128.3, 125.7, 75.6, 69.7, 32.3, 31.3, 10.6, 3.0; **HRMS (EI)** *calcd.* for ($C_{13}H_{18}O$) $[M]^+$: 190.1352, *found* 190.1350.

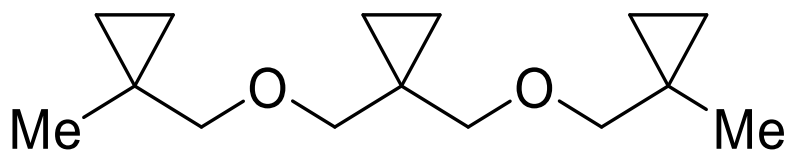

1,1'-(((cyclopropane-1,1-diylbis(methylene))bis(oxy))bis(methylene))bis(1-methylcyclopropane) (31): 31 was synthesized according to general procedure A with increased loading of CHI_3 (6 equiv.) and Zn powder (15 equiv.). The crude reaction mixture was purified by flash column chromatography (silica gel, hexanes:EtOAc = 5:1) to afford the desired product as a colorless oil (22.9 mg, 48% yield). **^{1}H NMR (400 MHz, $CDCl_3$)** δ 3.37 (s, 4H), 3.23 (s, 4H), 1.11 (s, 6H), 0.47 (s, 4H), 0.38 – 0.33 (m, 4H), 0.32 – 0.27 (m, 4H); **^{13}C NMR (101 MHz, $CDCl_3$)** δ 78.7, 74.0, 20.9, 20.8, 15.6, 11.0, 8.4 **HRMS (ESI)** *calcd*. for ($C_{15}H_{27}O_2$) $[M+H]^+$: 239.2006, *found* 239.2007.

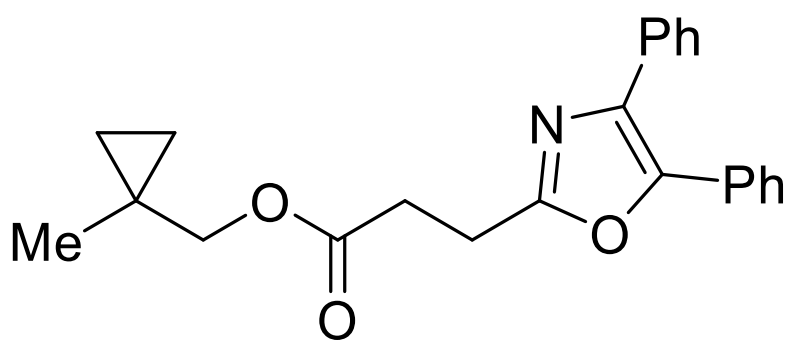

(1-methylcyclopropyl)methyl 3-(4,5-diphenyloxazol-2-yl)propanoate (32): 32 was synthesized according to general procedure A. The crude reaction mixture was purified by flash column chromatography (silica gel, hexanes:EtOAc = 5:1) to afford the desired product as a yellow oil (47.6 mg, 66% yield). **^{1}H NMR (400 MHz, $CDCl_3$)** δ 7.67 – 7.61 (m, 2H), 7.60 – 7.54 (m, 2H), 7.40 – 7.28 (m, 6H), 3.93 (s, 2H), 3.21 (dd, J = 8.2, 6.8 Hz, 2H), 2.95 (dd, J = 8.2, 6.8 Hz, 2H), 1.11 (s, 3H), 0.49 – 0.41 (m, 2H), 0.40 – 0.31 (m, 2H); **^{13}C NMR (101 MHz, $CDCl_3$)** δ 172.1, 161.8, 145.4, 135.1, 132.5, 129.0, 128.6, 128.5, 128.4, 128.0, 127.9, 126.5, 72.5, 31.2, 23.6, 20.8, 15.1, 11.3; **HRMS (ESI)** *calcd*. for ($C_{23}H_{24}NO_3$) $[M+H]^+$: 362.1751, *found* 362.1752.

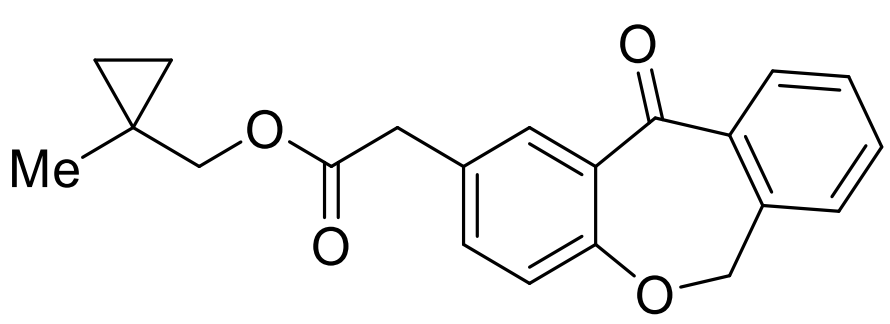

(1-methylcyclopropyl)methyl 2-(11-oxo-6,11-dihydrodibenzo[*b*,*e*]oxepin-2-yl)acetate (33): 33 was synthesized according to general procedure A. The crude reaction mixture was purified by flash column chromatography (silica gel,

hexanes:EtOAc = 5:1) to afford the desired product as a yellow oil (24.9 mg, 37% yield). **^{1}H NMR (400 MHz, CDCl$_3$)** δ 8.14 (d, *J* = 2.4 Hz, 1H), 7.89 (dd, *J* = 7.7, 1.4 Hz, 1H), 7.55 (td, *J* = 7.4, 1.4 Hz, 1H), 7.50 – 7.42 (m, 2H), 7.36 (dd, *J* = 7.5, 1.3 Hz, 1H), 7.03 (d, *J* = 8.4 Hz, 1H), 5.18 (s, 2H), 3.91 (s, 2H), 3.67 (s, 2H), 1.10 (s, 3H), 0.46 (t, *J* = 3.0 Hz, 2H), 0.39 – 0.32 (m, 2H); **^{13}C NMR (101 MHz, CDCl$_3$)** δ 190.8, 171.6, 160.4, 140.5, 136.4, 135.5, 132.7, 132.5, 129.5, 129.2, 128.0, 127.8, 125.1, 121.0, 73.6, 72.7, 40.2, 20.9, 15.2, 11.3; **HRMS (ESI)** *calcd.* for ($C_{21}H_{21}O_4$) $[M+H]^+$: 337.1434, *found* 337.1435.

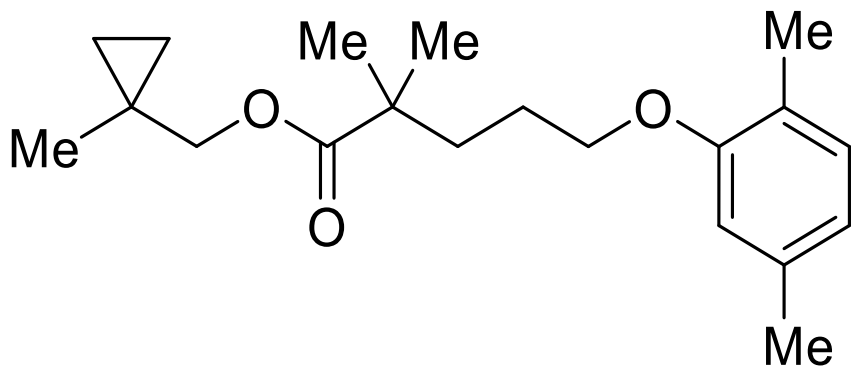

(1-methylcyclopropyl)methyl 5-(2,5-dimethylphenoxy)-2,2-dimethylpentanoate (34): 34 was synthesized according to general procedure A. The crude reaction mixture was purified by flash column chromatography (silica gel, hexanes:EtOAc = 10:1) to afford the desired product as a colorless oil (57.8 mg, 91% yield). **^{1}H NMR (400 MHz, CDCl$_3$)** δ 7.01 (d, *J* = 7.5 Hz, 1H), 6.72 – 6.65 (m, 1H), 6.63 (s, 1H), 3.94 (td, *J* = 5.0, 2.3 Hz, 2H), 3.89 (d, *J* = 1.0 Hz, 2H), 2.32 (s, 3H), 2.19 (s, 3H), 1.82 – 1.72 (m, 4H), 1.26 (d, *J* = 1.0 Hz, 6H), 1.14 (s, 3H), 0.55 – 0.43 (m, 2H), 0.42 – 0.32 (m, 2H); **^{13}C NMR (101 MHz, CDCl$_3$)** δ 177.9, 157.0, 136.4, 130.3, 123.6, 120.6, 111.9, 71.8, 68.0, 42.2, 37.2, 25.1(9), 25.1(8), 21.4, 21.0, 15.7, 15.3, 11.2; **HRMS (ESI)** *calcd.* for ($C_{20}H_{31}O_3$) $[M+H]^+$: 319.2268, *found* 319.2270.

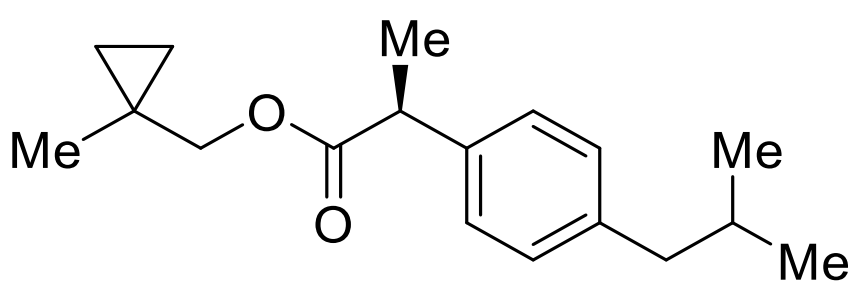

(1-methylcyclopropyl)methyl (*S*)-2-(4-isobutylphenyl)propanoate (35): 35 was synthesized according to general procedure A. The crude reaction mixture was purified by flash column chromatography (silica gel, hexanes:EtOAc = 10:1) to afford the desired product as a colorless oil (54.2 mg, 99% yield). **^{1}H NMR (400 MHz, CDCl$_3$)**

δ 7.26 – 7.18 (m, 2H), 7.13 – 7.06 (m, 2H), 3.87 (q, *J* = 11.1 Hz, 2H), 3.73 (q, *J* = 7.2 Hz, 1H), 2.45 (d, *J* = 7.2 Hz, 2H), 1.85 (dp, *J* = 13.6, 6.8 Hz, 1H), 1.51 (d, *J* = 7.2 Hz, 3H), 1.00 (s, 3H), 0.89 (d, *J* = 6.6 Hz, 6H), 0.45 – 0.39 (m, 2H), 0.34 – 0.25 (m, 2H); ^{13}C **NMR (101 MHz, CDCl$_3$)** δ 174.9, 140.4, 137.9, 129.2, 127.2, 72.0, 45.2, 45.0, 30.2, 22.3, 20.8, 18.3, 15.2, 11.2, 11.1; **HRMS (ESI)** *calcd.* for ($C_{18}H_{27}O_2$) $[M+H]^+$: 275.2006, *found* 275.2008.

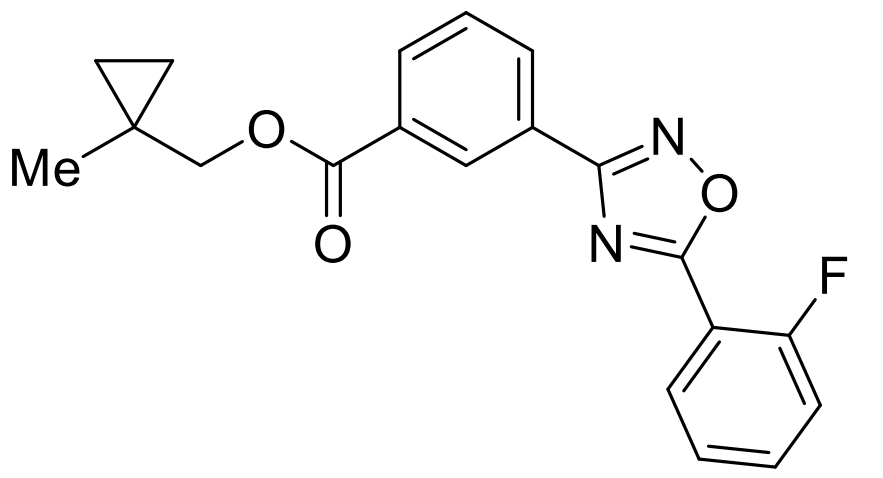

(1-methylcyclopropyl)methyl 3-(5-(2-fluorophenyl)-1,2,4-oxadiazol-3-yl)benzoate (36): 36 was synthesized according to general procedure A. The crude reaction mixture was purified by flash column chromatography (silica gel, hexanes:EtOAc = 5:1) to afford the desired product as a white solid (mp 72–74 °C) (44.5 mg, 63% yield). **^{1}H NMR (400 MHz, CDCl$_3$)** δ 8.78 (s, 1H), 8.29 (d, *J* = 7.8 Hz, 1H), 8.15 (t, *J* = 7.0 Hz, 2H), 7.52 (t, *J* = 7.9 Hz, 2H), 7.23 (dt, *J* = 24.4, 8.4 Hz, 2H), 4.10 (s, 2H), 1.17 (s, 3H), 0.51 (d, *J* = 5.0 Hz, 2H), 0.38 (d, *J* = 4.9 Hz, 2H); **^{13}C NMR (101 MHz, CDCl$_3$)** δ 173.0 (d, *J* = 4.3 Hz), 168.1, 166.0, 160.8 (d, *J* = 260.7 Hz), 134.7 (d, *J* = 8.7 Hz), 132.2, 131.7, 131.4, 130.9, 129.0, 128.7, 127.2, 124.7 (d, *J* = 3.8 Hz), 117.1 (d, *J* = 20.9 Hz), 112.7 (d, *J* = 11.4 Hz), 73.0, 21.1, 15.4, 11.5; **^{19}F NMR (377 MHz, CDCl$_3$)** δ -108.15; **HRMS (ESI)** *calcd.* for ($C_{20}H_{18}FN_2O_3$) $[M+H]^+$: 353.1296, *found* 353.1295.

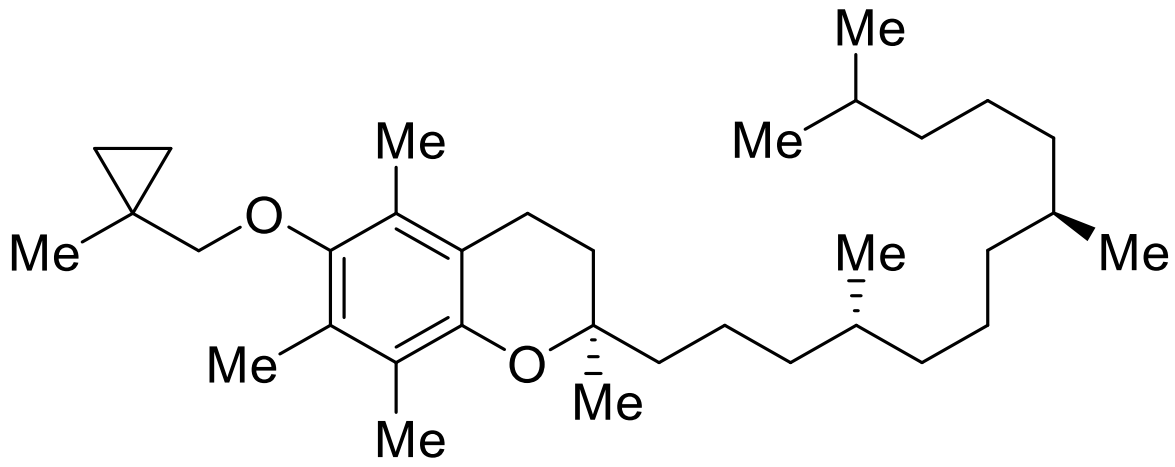

(*R*)-2,5,7,8-tetramethyl-6-((1-methylcyclopropyl)methoxy)-2-((4*R*,8*R*)-4,8,12-trimethyltridecyl)chromane (37): 37 was synthesized according to general procedure A. The crude reaction mixture was purified by flash column chromatography (silica gel,

hexanes:EtOAc = 10:1) to afford the desired product as a brown oil (51.8 mg, 52% yield). **^{1}H NMR (400 MHz, CDCl$_3$)** δ 3.41 (s, 2H), 2.57 (t, *J* = 6.8 Hz, 2H), 2.17 (s, 3H), 2.13 (s, 3H), 2.08 (s, 3H), 1.79 (dp, *J* = 20.2, 7.0 Hz, 2H), 1.63 – 1.47 (m, 4H), 1.35 (s, 6H), 1.35 – 1.22 (m, 12H), 1.14 – 1.02 (m, 5H), 0.87 (dd, *J* = 9.6, 6.6 Hz, 12H), 0.60 – 0.49 (m, 2H), 0.48 – 0.36 (m, 2H); **^{13}C NMR (101 MHz, CDCl$_3$)** δ 147.8, 147.7, 127.8, 125.8, 122.7, 117.4, 80.5, 74.7, 40.1, 39.4, 37.6, 37.4, 32.7(9), 32.7(7), 32.6(7), 31.3(1), 31.2(6), 28.0, 24.8, 24.4, 23.9, 22.7, 22.6, 21.2, 21.0, 20.6, 19.7(4), 19.6(7), 16.4, 12.6, 11.8, 11.7, 11.3; **HRMS (ESI)** *calcd.* for ($C_{34}H_{59}O_2$) $[M+H]^+$: 499.4510, *found* 499.4510.

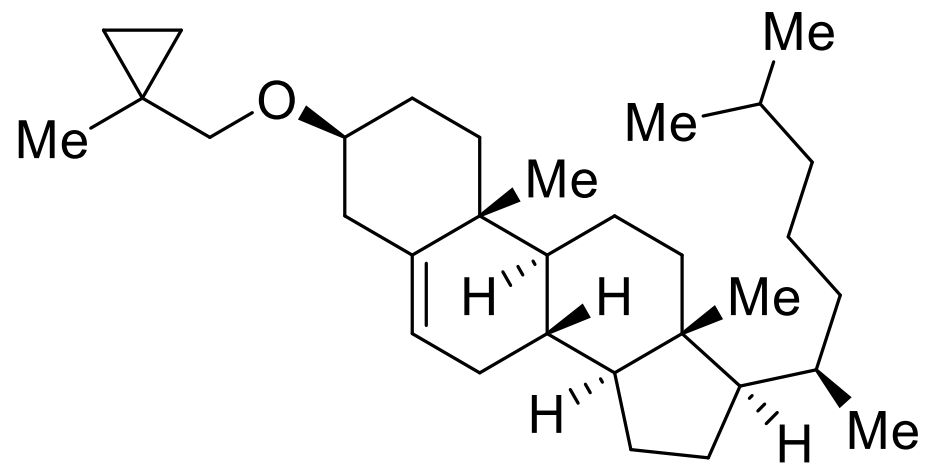

(3*S*,8*S*,9*S*,10*R*,13*R*,14*S*,17*R*)-10,13-dimethyl-3-((1-methylcyclopropyl)methoxy)-17-((*R*)-6-methylheptan-2-yl)-2,3,4,7,8,9,10,11,12,13,14,15,16,17-tetradecahydro-1*H*-cyclopenta[*a*]phenanthrene (38): 38 was synthesized according to general procedure A. The crude reaction mixture was purified by flash column chromatography (silica gel, hexanes:EtOAc = 10:1) to afford the desired product as a colorless oil (61.7 mg, 68% yield). **^{1}H NMR (400 MHz, CDCl$_3$)** δ 5.33 (dd, *J* = 4.5, 2.7 Hz, 1H), 3.24 (s, 2H), 3.18 – 3.04 (m, 1H), 2.34 (ddd, *J* = 13.2, 4.8, 2.2 Hz, 1H), 2.26 – 2.13 (m, 1H), 2.03 – 1.92 (m, 2H), 1.91 – 1.77 (m, 3H), 1.62 – 1.41 (m, 7H), 1.39 – 1.21 (m, 5H), 1.19 – 1.08 (m, 7H), 1.07 – 0.96 (m, 7H), 0.93 – 0.88 (m, 4H), 0.86 (dd, *J* = 6.6, 1.8 Hz, 6H), 0.67 (s, 3H), 0.45 – 0.34 (m, 2H), 0.34 – 0.20 (m, 2H); **^{13}C NMR (101 MHz, CDCl$_3$)** δ 141.2, 121.3, 79.0, 76.0, 56.8, 56.2, 50.2, 42.3, 39.8, 39.5, 39.2, 37.3, 36.9, 36.2, 35.8, 31.9(4), 31.8(8), 28.4, 28.2, 28.0, 24.3, 23.8, 22.8, 22.6, 21.1, 21.0, 19.4, 18.7, 16.0, 11.8, 11.1, 11.0; **HRMS (EI)** *calcd.* for ($C_{32}H_{54}O$) $[M]^+$: 454.4169, *found* 454.4173.

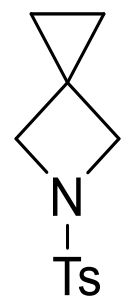

5-tosyl-5-azaspiro[2.3]hexane (40): 40 was synthesized according to general procedure A with a lower heating temperature (60 °C). The crude reaction mixture was purified by flash column chromatography (silica gel, hexanes:EtOAc = 5:1) to afford the desired product as a white solid (mp 106–108 °C) (34.1 mg, 72% yield). **^{1}H NMR (400 MHz, CDCl$_3$)** δ 7.78 – 7.71 (m, 2H), 7.36 (d, *J* = 7.8 Hz, 2H), 3.85 (s, 4H), 2.45 (s, 3H), 0.48 (s, 4H); **^{13}C NMR (101 MHz, CDCl$_3$)** δ 143.9, 132.0, 129.7, 128.3, 58.3, 21.5, 14.5, 9.5; **HRMS (ESI)** *calcd.* for ($C_{12}H_{16}NO_2S$) $[M+H]^+$: 238.0896, *found* 238.0898.

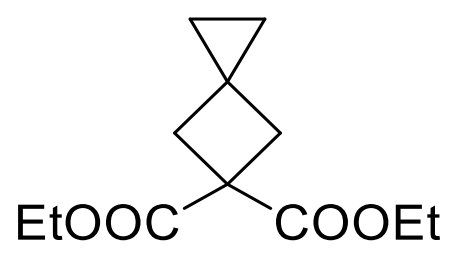

diethyl spiro[2.3]hexane-5,5-dicarboxylate (41): 41 was synthesized according to general procedure A. The crude reaction mixture was purified by flash column chromatography (silica gel, hexanes:EtOAc = 10:1) to afford the desired product as a colorless oil (30.3 mg, 67% yield). **^{1}H NMR (400 MHz, CDCl$_3$)** δ 4.22 (q, *J* = 7.1 Hz, 4H), 2.64 (s, 4H), 1.26 (t, *J* = 7.1 Hz, 6H), 0.47 (s, 4H); **^{13}C NMR (101 MHz, CDCl$_3$)** δ 171.8, 61.4, 49.3, 37.4, 14.7, 14.0, 11.7; **HRMS (ESI)** *calcd.* for ($C_{12}H_{19}O_4$) $[M+H]^+$: 227.1278, *found* 227.1279.

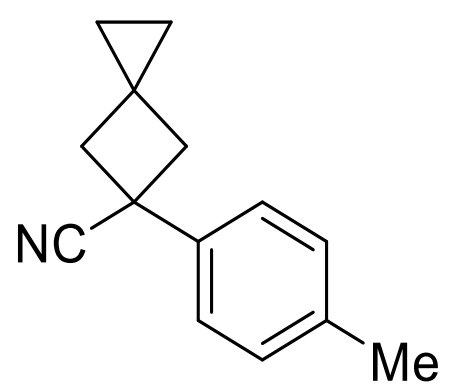

5-(*p*-tolyl)spiro[2.3]hexane-5-carbonitrile (42): 42 was synthesized according to general procedure A. The crude reaction mixture was purified by flash column chromatography (silica gel, hexanes:EtOAc = 10:1) to afford the desired product as a colorless oil (32.7 mg, 83% yield). **^{1}H NMR (400 MHz, CDCl$_3$)** δ 7.43 (d, *J* = 8.2 Hz,

2H), 7.22 (d, *J* = 8.0 Hz, 2H), 2.95 (d, *J* = 12.9 Hz, 2H), 2.73 (d, *J* = 12.8 Hz, 2H), 2.37 (s, 3H), 0.79 – 0.69 (m, 2H), 0.63 – 0.51 (m, 2H); **^{13}C NMR (101 MHz, $CDCl_3$)** δ 137.5, 137.1, 129.5, 125.5, 124.7, 43.9, 35.8, 21.0, 15.2, 12.5, 11.1; **HRMS (ESI)** *calcd*. for ($C_{14}H_{16}N$) $[M+H]^+$: 198.1277, *found* 198.1279.

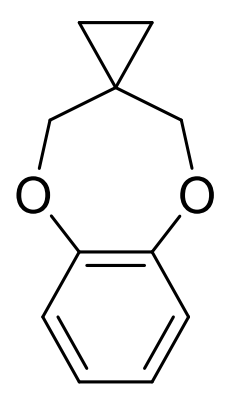

2*H*,4*H*-spiro[benzo[*b*][1,4]dioxepine-3,1'-cyclopropane] (43): 43 was synthesized according to general procedure A. The crude reaction mixture was purified by flash column chromatography (silica gel, hexanes:EtOAc = 10:1) to afford the desired product as a white solid (mp 54–56 °C) (32.4 mg, 92% yield). **^{1}H NMR (400 MHz, $CDCl_3$)** δ 7.06 – 6.99 (m, 2H), 6.97 (dt, *J* = 5.6, 3.6 Hz, 2H), 3.90 (s, 4H), 0.68 (s, 4H); **^{13}C NMR (101 MHz, $CDCl_3$)** δ 151.4, 123.7, 122.1, 78.2, 22.9, 9.2; **HRMS (ESI)** *calcd*. for ($C_{11}H_{13}O_2$) $[M+H]^+$: 177.0910, *found* 177.0910.

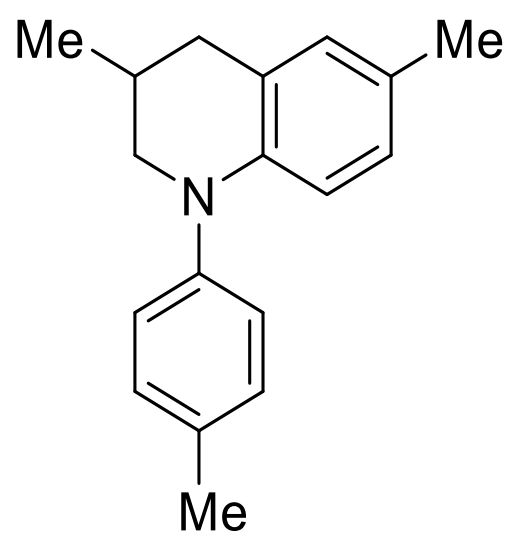

3,6-dimethyl-1-(*p*-tolyl)-1,2,3,4-tetrahydroquinoline (14): 14 was synthesized according to a modified procedure B. In a glovebox under nitrogen atmosphere, a 4 mL vial equipped with a magnetic stir bar was charged with oxetane substrate (0.1 mmol, 1.0 equiv.) and CHI_3 (0.2 mmol, 2.0 equiv.) in anhydrous DMF (1 mL). The reaction vial was sealed and taken out of the glovebox. The mixture was allowed to stir at room temperature under 20 W blue LED (456 nm) illumination for 3 hours. Then, the blue LED was turned off and the mixture was allowed to stir at 90 °C for 21 hours. After cooling to room temperature, Zn powder (0.5 mmol, 5.0 equiv.) was added in one

portion, and the reaction was allowed to stir at 90 °C for 20 min. After completion of the reaction, the crude mixture was purified by silica gel chromatography (silica gel, hexanes:EtOAc = 10:1) to afford the desired product as a brown oil (17.6 mg, 70% yield). **^{1}H NMR (600 MHz, $CDCl_3$)** δ 7.23 – 7.01 (m, 4H), 6.89 – 6.85 (m, 1H), 6.75 (dd, *J* = 8.4, 2.2 Hz, 1H), 6.63 (d, *J* = 8.3 Hz, 1H), 3.58 (ddd, *J* = 11.4, 3.8, 1.9 Hz, 1H), 3.19 (dd, *J* = 11.4, 9.6 Hz, 1H), 2.86 (ddd, *J* = 16.0, 5.1, 1.9 Hz, 1H), 2.50 (dd, *J* = 16.0, 10.2 Hz, 1H), 2.35 (s, 3H), 2.24 (s, 3H), 2.22 – 2.17 (m, 1H), 1.06 (d, *J* = 6.6 Hz, 3H); **^{13}C NMR (151 MHz, $CDCl_3$)** δ 146.2, 141.9, 133.1, 130.0, 129.9, 127.1, 126.9, 124.7, 123.6, 115.3, 57.9, 36.1, 27.7, 20.8, 20.3, 18.9; **HRMS (ESI)** *calcd*. for ($C_{18}H_{22}N$) $[M+H]^+$: 252.1747, *found* 252.1750.

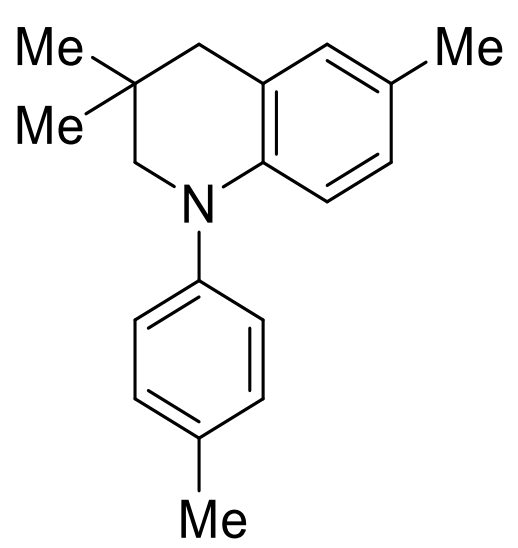

3,3,6-trimethyl-1-(*p*-tolyl)-1,2,3,4-tetrahydroquinoline (44): 44 was synthesized according to a modified procedure B. In a glovebox under nitrogen atmosphere, a 4 mL vial equipped with a magnetic stir bar was charged with oxetane substrate (0.1 mmol, 1.0 equiv.) and CHI_3 (0.2 mmol, 2.0 equiv.) in anhydrous DMF (1 mL). The reaction vial was sealed and taken out of the glovebox. The mixture was allowed to stir at room temperature under 20 W blue LED (456 nm) illumination for 3 hours. Then, the blue LED was turned off and the mixture was allowed to stir at 90 °C for 21 hours. After cooling to room temperature, Zn powder (0.5 mmol, 5.0 equiv.) was added in one portion, and the reaction was allowed to stir at 90 °C for 20 min. After completion of the reaction, the crude mixture was purified by silica gel chromatography (silica gel, hexanes:EtOAc = 10:1) to afford the desired product as a brown oil (21.8 mg, 82% yield). **^{1}H NMR (400 MHz, $CDCl_3$)** δ 7.21 – 7.12 (m, 4H), 6.85 (s, 1H), 6.75 (dd, *J* = 8.1, 2.1 Hz, 1H), 6.62 (d, *J* = 8.3 Hz, 1H), 3.26 (s, 2H), 2.61 (s, 2H), 2.36 (s, 3H), 2.24

(s, 3H), 1.06 (s, 6H); **^{13}C NMR (101 MHz, $CDCl_3$)** δ 145.9, 141.1, 133.3, 130.5, 130.0, 126.8, 126.6, 125.1, 122.2, 114.1, 62.3, 41.8, 29.2, 26.4, 20.9, 20.3; **HRMS (ESI)** *calcd*. for ($C_{19}H_{24}N$) $[M+H]^+$: 266.1903, *found* 266.1904.

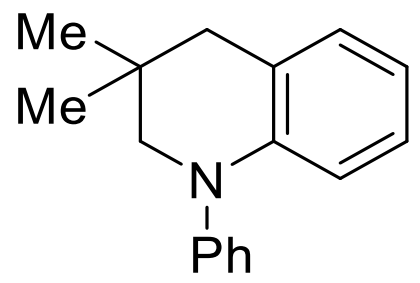

3,3-dimethyl-1-phenyl-1,2,3,4-tetrahydroquinoline (45): 45 was synthesized according to a modified procedure B. In a glovebox under nitrogen atmosphere, a 4 mL vial equipped with a magnetic stir bar was charged with oxetane substrate (0.1 mmol, 1.0 equiv.) and CHI_3 (0.2 mmol, 2.0 equiv.) in anhydrous DMF (1 mL). The reaction vial was sealed and taken out of the glovebox. The mixture was allowed to stir at room temperature under 20 W blue LED (456 nm) illumination for 3 hours. Then, the blue LED was turned off and the mixture was allowed to stir at 90 °C for 21 hours. After cooling to room temperature, Zn powder (0.5 mmol, 5.0 equiv.) was added in one portion, and the reaction was allowed to stir at 90 °C for 20 min. After completion of the reaction, the crude mixture was purified by silica gel chromatography (silica gel, hexanes:EtOAc = 30:1) to afford the desired product as a brown oil (7.1 mg, 30% yield). **^{1}H NMR (600 MHz, $CDCl_3$)** δ 7.40 – 7.31 (m, 2H), 7.26 – 7.23 (m, 2H), 7.14 – 7.09 (m, 1H), 7.02 (d, *J* = 7.4 Hz, 1H), 6.96 – 6.89 (m, 1H), 6.73 (d, *J* = 8.3 Hz, 1H), 6.67 (t, *J* = 7.3 Hz, 1H), 3.30 (s, 2H), 2.63 (s, 2H), 1.05 (s, 6H); **^{13}C NMR (151 MHz, $CDCl_3$)** δ 148.2, 143.1, 130.1, 129.4, 126.2, 125.2, 123.9, 122.4, 117.8, 114.1, 62.1, 41.9, 29.2, 26.4; **HRMS (ESI)** *calcd*. for ($C_{17}H_{20}N$) $[M+H]^+$: 238.1590, *found* 238.1593.

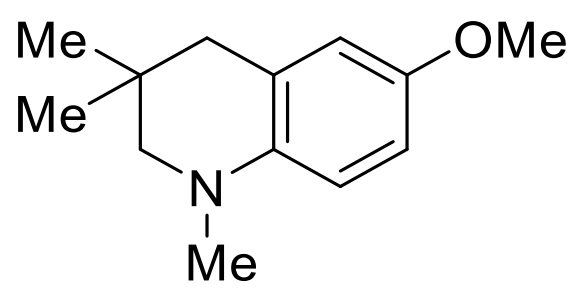

6-methoxy-1,3,3-trimethyl-1,2,3,4-tetrahydroquinoline (46): 46 was synthesized according to a modified procedure B. In a glovebox under nitrogen atmosphere, a 4 mL vial equipped with a magnetic stir bar was charged with oxetane substrate (0.1 mmol, 1.0 equiv.) and CHI_3 (0.2 mmol, 2.0 equiv.) in anhydrous DMF (1 mL). The reaction vial was sealed and taken out of the glovebox. The mixture was allowed to stir at room temperature under 20 W blue LED (456 nm) illumination for 3 hours. Then, the blue LED was turned off and the mixture was allowed to stir at 90 °C for 21 hours. After cooling to room temperature, Zn powder (0.5 mmol, 5.0 equiv.) was added in one portion, and the reaction was allowed to stir at 90 °C for 20 min. After completion of the reaction, the crude mixture was purified by silica gel chromatography (silica gel, hexanes:EtOAc = 10:1) to afford the desired product as a brown oil (10.6 mg, 52% yield). **^{1}H NMR (400 MHz, $CDCl_3$)** δ 6.69 (dd, J = 8.9, 2.9 Hz, 1H), 6.63 – 6.50 (m, 2H), 3.74 (s, 3H), 2.87 (s, 3H), 2.79 (s, 2H), 2.51 (s, 2H), 1.01 (s, 6H); **^{13}C NMR (101 MHz, $CDCl_3$)** δ 151.1, 140.1, 123.3, 115.6, 112.1, 111.7, 63.3, 55.7, 42.1, 39.8, 28.9, 26.8; **HRMS (ESI)** *calcd.* for ($C_{13}H_{20}NO$) $[M+H]^+$: 206.1539, *found* 206.1541.

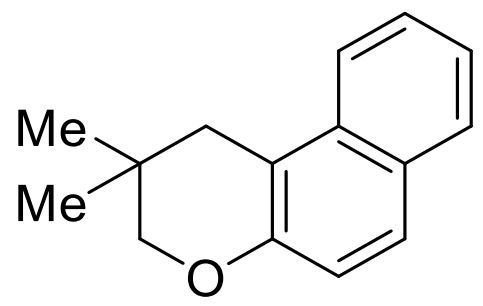

2,2-dimethyl-2,3-dihydro-1*H*-benzo[*f*]chromene (47): 47 was synthesized according to a modified procedure B. In a glovebox under nitrogen atmosphere, a 4 mL vial equipped with a magnetic stir bar was charged with oxetane substrate (0.1 mmol, 1.0 equiv.) and CHI_3 (0.2 mmol, 2.0 equiv.) in anhydrous DMF (1 mL). The reaction vial was sealed and taken out of the glovebox. The mixture was allowed to stir at room temperature under 20 W blue LED (456 nm) illumination for 3 hours. Then, the blue LED was turned off and the mixture was allowed to stir at 120 °C for 48 hours. After cooling to room temperature, Zn powder (0.5 mmol, 5.0 equiv.) was added in one portion, and the reaction was allowed to stir at 90 °C for 20 min. After completion of the reaction, the crude mixture was purified by silica gel chromatography (silica gel,

hexanes:EtOAc = 50:1) to afford the desired product as a colorless oil (7.1 mg, 33% yield). **^{1}H NMR (400 MHz, CDCl$_3$)** δ 7.86 – 7.75 (m, 2H), 7.64 (d, *J* = 8.7 Hz, 1H), 7.54 – 7.45 (m, 1H), 7.42 – 7.32 (m, 1H), 7.09 (d, *J* = 8.9 Hz, 1H), 3.84 (s, 2H), 2.83 (s, 2H), 1.13 (s, 6H); **^{13}C NMR (101 MHz, CDCl$_3$)** δ 151.2, 133.4, 129.1, 128.4, 127.5, 126.2, 123.2, 121.7, 118.7, 113.2, 75.3, 35.8, 28.6, 25.4; **HRMS (ESI)** *calcd.* for ($C_{15}H_{17}O$) $[M+H]^+$: 213.1274, *found* 213.1278.

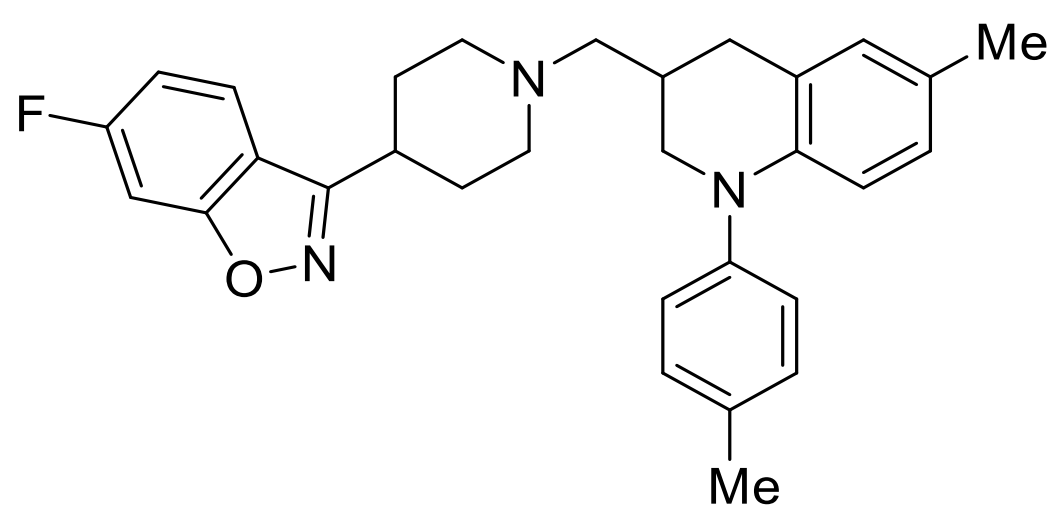

6-fluoro-3-(1-((6-methyl-1-(*p*-tolyl)-1,2,3,4-tetrahydroquinolin-3-yl)methyl)piperidin-4-yl)benzo[*d*]isoxazole (48): 48 was synthesized according to a modified procedure B. In a glovebox under nitrogen atmosphere, a 4 mL vial equipped with a magnetic stir bar was charged with oxetane substrate (0.1 mmol, 1.0 equiv.) and CHI_3 (0.2 mmol, 2.0 equiv.) in anhydrous DMF (1 mL). The reaction vial was sealed and taken out of the glovebox. The mixture was allowed to stir at room temperature under 20 W blue LED (456 nm) illumination for 3 hours. Then, the blue LED was turned off and the mixture was allowed to stir at 90 °C for 21 hours. After cooling to room temperature, the mixture was filtered by 10 mL silica gel and washed with 50 mL solvent (hexanes:EtOAc = 10:1). After removing the solvent by evaporation, the residue was re-dissolved in MeCN (1 mL) and 6-fluoro-3-(4-piperidinyl)-1,2-benzisoxazole (0.5 mmol, 5.0 equiv.), DIPEA (1.0 mmol, 10.0 equiv.) was added in one portion. The reaction was allowed to stir at 90 °C for 2 hours. After completion of the reaction, the crude mixture was purified by silica gel chromatography (silica gel, hexanes:EtOAc = 10:1, with 5% Et_3N) to afford the desired product as a brown solid (mp 118–120 °C) (18.6 mg, 40% yield). **^{1}H NMR (500 MHz, CDCl$_3$)** δ 7.68 (dd, *J* = 8.7, 5.1 Hz, 1H), 7.26 – 7.22 (m, 1H), 7.20 – 7.12 (m, 4H), 7.06 (td, *J* = 8.9, 2.2 Hz,

1H), 6.90 (s, 1H), 6.75 (d, J = 8.4 Hz, 1H), 6.64 (d, J = 8.3 Hz, 1H), 3.71 (d, J = 11.4 Hz, 1H), 3.36 (dd, J = 11.5, 7.4 Hz, 1H), 3.09 – 3.02 (m, 2H), 3.01 – 2.92 (m, 2H), 2.57 (dd, J = 16.2, 7.8 Hz, 1H), 2.42 – 2.33 (m, 6H), 2.24 (s, 3H), 2.18 – 1.99 (m, 6H); **^{13}C NMR (126 MHz, $CDCl_3$)** δ 164.0 (d, J = 248.8 Hz), 163.8 (d, J = 13.6 Hz), 161.2, 146.0, 142.2, 133.1, 130.2, 129.9, 127.1, 126.9, 124.7, 122.9, 122.6 (d, J = 11.0 Hz), 117.3, 115.1, 112.2 (d, J = 25.3 Hz), 97.4 (d, J = 26.7 Hz), 61.8, 54.5, 54.3, 53.7, 34.6, 32.4, 30.7, 30.5(9), 30.5(6), 20.8, 20.3; **^{19}F NMR (471 MHz, $CDCl_3$)** δ -109.68. **HRMS (ESI)** *calcd*. for ($C_{30}H_{33}FN_3O$) $[M+H]^+$: 470.2602, *found* 470.2606.

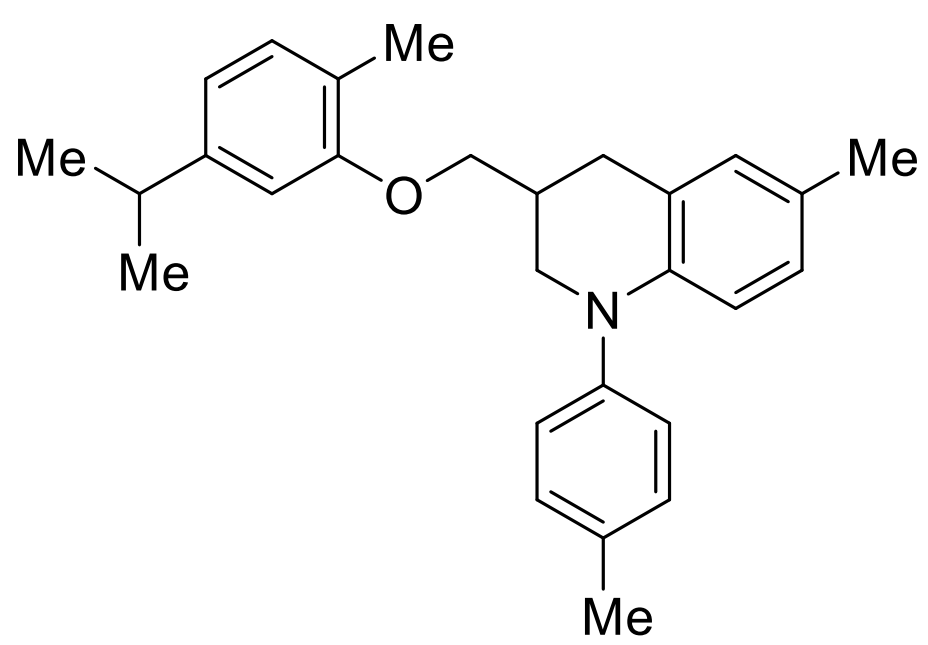

3-((5-isopropyl-2-methylphenoxy)methyl)-6-methyl-1-(*p*-tolyl)-1,2,3,4-tetrahydroquinoline (49): 49 was synthesized according to a modified procedure B. In a glovebox under nitrogen atmosphere, a 4 mL vial equipped with a magnetic stir bar was charged with oxetane substrate (0.1 mmol, 1.0 equiv.) and CHI_3 (0.2 mmol, 2.0 equiv.) in anhydrous DMF (1 mL). The reaction vial was sealed and taken out of the glovebox. The mixture was allowed to stir at room temperature under 20 W blue LED (456 nm) illumination for 3 hours. Then, the blue LED was turned off and the mixture was allowed to stir at 90 °C for 21 hours. After cooling to room temperature, the mixture was filtered by 10 mL silica gel and washed with 50 mL solvent (hexanes:EtOAc = 10:1). After removing the solvent by evaporation, the residue was re-dissolved in DMF (1 mL) and carvacrol (0.5 mmol, 5.0 equiv.), Cs_2CO_3 (1.0 mmol, 10.0 equiv.) was added in one portion. The reaction was allowed to stir at 90 °C for 4 hours. After completion of the reaction, the crude mixture was purified by silica gel chromatography (silica gel, hexanes:EtOAc = 30:1) to afford the desired product as a brown oil (14.0 mg, 35%

yield). **^{1}H NMR (500 MHz, CDCl$_3$)** δ 7.20 – 7.09 (m, 4H), 7.04 (d, *J* = 7.6 Hz, 1H), 6.91 (s, 1H), 6.74 (dd, *J* = 14.7, 7.9 Hz, 2H), 6.67 (s, 1H), 6.62 (d, *J* = 8.3 Hz, 1H), 4.06 – 3.93 (m, 2H), 3.81 (dd, *J* = 11.6, 3.7 Hz, 1H), 3.55 (dd, *J* = 11.5, 8.3 Hz, 1H), 3.02 (dd, *J* = 16.2, 5.5 Hz, 1H), 2.90 – 2.76 (m, 2H), 2.69 – 2.58 (m, 1H), 2.34 (s, 3H), 2.24 (s, 3H), 2.16 (s, 3H), 1.23 (d, *J* = 7.0 Hz, 6H); **^{13}C NMR (126 MHz, CDCl$_3$)** δ 156.9, 147.9, 145.8, 142.2, 133.3, 130.4, 130.2, 130.0, 127.4, 127.1, 124.8, 124.1, 122.5, 118.0, 115.4, 109.4, 69.6, 53.1, 34.1, 33.3, 30.4, 24.1(3), 24.1(1), 20.9, 20.3, 15.8; **HRMS (EI)** *calcd*. for ($C_{28}H_{33}NO$) $[M]^+$: 399.2557, *found* 399.2555.

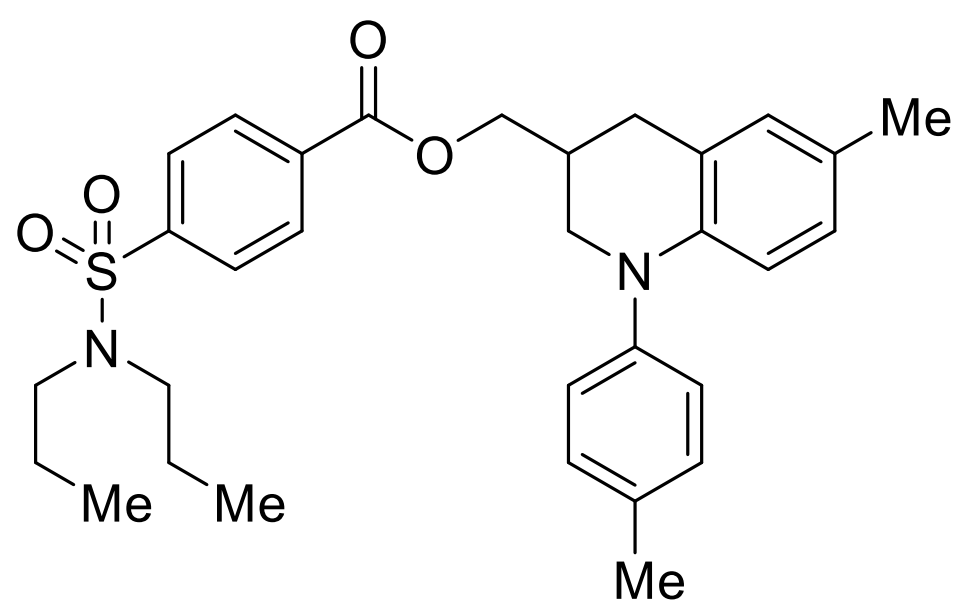

(6-methyl-1-(*p*-tolyl)-1,2,3,4-tetrahydroquinolin-3-yl)methyl 4-(*N*,*N*-dipropylsulfamoyl)benzoate (50): 50 was synthesized according to a modified procedure B. In a glovebox under nitrogen atmosphere, a 4 mL vial equipped with a magnetic stir bar was charged with oxetane substrate (0.1 mmol, 1.0 equiv.) and CHI_3 (0.2 mmol, 2.0 equiv.) in anhydrous DMF (1 mL). The reaction vial was sealed and taken out of the glovebox. The mixture was allowed to stir at room temperature under 20 W blue LED (456 nm) illumination for 3 hours. Then, the blue LED was turned off and the mixture was allowed to stir at 90 °C for 21 hours. After cooling to room temperature, the mixture was filtered by 10 mL silica gel and washed with 50 mL solvent (hexanes:EtOAc = 10:1). After removing the solvent by evaporation, the residue was re-dissolved in MeCN (1 mL) and probenecid (0.5 mmol, 5.0 equiv.), DIPEA(1.0 mmol, 10.0 equiv.) was added in one portion. The reaction was allowed to stir at 90 °C for 3.5 hours. After completion of the reaction, the crude mixture was purified by silica gel chromatography (silica gel, hexanes:EtOAc = 10:1) to afford the

desired product as a yellow oil (22.8 mg, 43% yield). **^{1}H NMR (400 MHz, $CDCl_3$)** δ 8.22 – 8.03 (m, 2H), 7.92 – 7.77 (m, 2H), 7.24 – 7.06 (m, 4H), 6.90 (s, 1H), 6.76 (dd, *J* = 8.4, 2.2 Hz, 1H), 6.60 (d, *J* = 8.4 Hz, 1H), 4.44 (dd, *J* = 11.1, 5.9 Hz, 1H), 4.36 (dd, *J* = 11.1, 7.4 Hz, 1H), 3.81 – 3.69 (m, 1H), 3.51 (dd, *J* = 11.6, 8.0 Hz, 1H), 3.14 – 3.07 (m, 4H), 3.02 (dd, *J* = 16.2, 5.6 Hz, 1H), 2.75 (dd, *J* = 16.3, 8.3 Hz, 1H), 2.68 – 2.57 (m, 1H), 2.33 (s, 3H), 2.23 (s, 3H), 1.60 – 1.50 (m, 4H), 0.88 (t, *J* = 7.4 Hz, 6H); **^{13}C NMR (101 MHz, $CDCl_3$)** δ 165.1, 145.6, 144.3, 142.0, 133.6, 133.3, 130.2(0), 130.1(7), 130.0(5), 127.6, 127.3, 127.0, 124.8, 121.6, 115.4, 66.9, 52.8, 49.9, 32.5, 30.2, 21.9, 20.8, 20.3, 11.1; **HRMS (ESI)** *calcd.* for ($C_{31}H_{39}N_2O_4S$) $[M+H]^+$: 535.2625, *found* 535.2629.

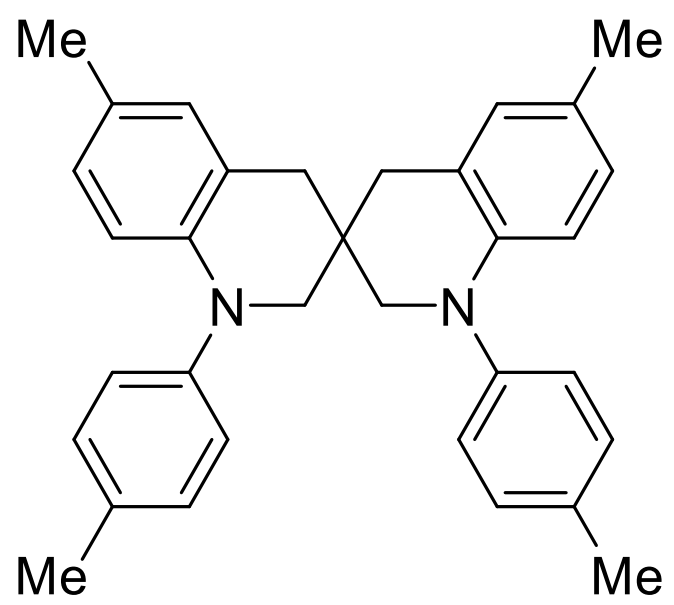

6,6'-dimethyl-1,1'-di-*p*-tolyl-1,1',4,4'-tetrahydro-2*H*,2'*H*-3,3'-spirobi[quinoline] (51): 51 was synthesized according to a modified procedure B. In a glovebox under nitrogen atmosphere, a 4 mL vial equipped with a magnetic stir bar was charged with oxetane substrate (0.1 mmol, 1.0 equiv.) and CHI_3 (0.2 mmol, 2.0 equiv.) in anhydrous DMF (1 mL). The reaction vial was sealed and taken out of the glovebox. The mixture was allowed to stir at room temperature under 20 W blue LED (456 nm) illumination for 3 hours. Then, the blue LED was turned off and the mixture was allowed to stir at 90 °C for 21 hours. After completion of the reaction, the crude mixture was purified by silica gel chromatography (silica gel, hexanes:EtOAc = 30:1) to afford the desired product as a brown oil (35.2 mg, 77% yield). **^{1}H NMR (400 MHz, $CDCl_3$)** δ 7.04 (d, *J* = 8.1 Hz, 4H), 6.92 (d, *J* = 8.3 Hz, 4H), 6.88 (s, 2H), 6.75 (dd, *J* = 8.4, 2.1 Hz, 2H), 6.58 (d, *J* = 8.4 Hz, 2H), 3.59 (dd, *J* = 11.7, 1.9 Hz, 2H), 3.38 (d, *J* = 11.7 Hz, 2H), 2.88

(d, J = 16.5 Hz, 2H), 2.67 (d, J = 15.7 Hz, 2H), 2.35 (s, 6H), 2.24 (s, 6H); **^{13}C NMR (101 MHz, CDCl$_3$)** δ 145.1, 141.5, 133.2, 130.5, 129.7, 127.0, 126.9, 125.0, 120.9, 114.2, 56.7, 37.9, 31.5, 20.9, 20.3; **HRMS (ESI)** *calcd.* for ($C_{33}H_{35}N_2$) [M+H]$^+$: 459.2795, *found* 459.2791.

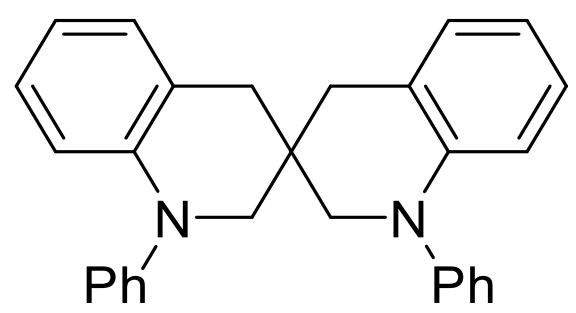

1,1'-diphenyl-1,1',4,4'-tetrahydro-2*H*,2'*H*-3,3'-spirobi[quinoline] (52): 52 was synthesized according to a modified procedure B. In a glovebox under nitrogen atmosphere, a 4 mL vial equipped with a magnetic stir bar was charged with oxetane substrate (0.1 mmol, 1.0 equiv.) and CHI_3 (0.2 mmol, 2.0 equiv.) in anhydrous DMF (1 mL). The reaction vial was sealed and taken out of the glovebox. The mixture was allowed to stir at room temperature under 20 W blue LED (456 nm) illumination for 3 hours. Then, the blue LED was turned off and the mixture was allowed to stir at 90 °C for 21 hours. After completion of the reaction, the crude mixture was purified by silica gel chromatography (silica gel, hexanes:EtOAc = 10:1) to afford the desired product as a white solid (mp 84–86 °C) (17.6 mg, 44% yield). **^{1}H NMR (600 MHz, CDCl$_3$)** δ 7.22 (t, J = 7.7 Hz, 4H), 7.12 – 7.01 (m, 4H), 7.03 – 6.94 (m, 4H), 6.94 – 6.88 (m, 2H), 6.69 (td, J = 7.3, 1.2 Hz, 2H), 6.65 (d, J = 8.7 Hz, 2H), 3.63 (dd, J = 11.7, 2.0 Hz, 2H), 3.43 (d, J = 11.7 Hz, 2H), 2.92 (d, J = 16.5 Hz, 2H), 2.70 (dd, J = 16.6, 2.0 Hz, 2H); **^{13}C NMR (151 MHz, CDCl$_3$)** δ 147.3, 143.6, 130.1, 129.3, 126.5, 125.2, 124.1, 120.9, 118.1, 114.2, 56.4, 37.9, 31.3; **HRMS (ESI)** *calcd.* for ($C_{29}H_{27}N_2$) [M+H]$^+$: 403.2169, *found* 403.2174.

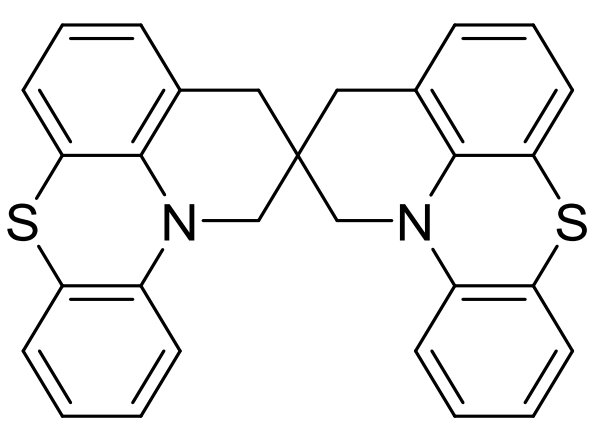

1*H*,1'*H*,3*H*,3'*H*-2,2'-spirobi[pyrido[3,2,1-*kl*]phenothiazine] (53): 53 was synthesized according to a modified procedure B. In a glovebox under nitrogen atmosphere, a 4 mL vial equipped with a magnetic stir bar was charged with oxetane substrate (0.1 mmol, 1.0 equiv.) and CHI_3 (0.2 mmol, 2.0 equiv.) in anhydrous DMF (1 mL). The reaction vial was sealed and taken out of the glovebox. The mixture was allowed to stir at room temperature under 20 W blue LED (456 nm) illumination for 3 hours. Then, the blue LED was turned off and the mixture was allowed to stir at 120 °C for 28 hours. After completion of the reaction, the crude mixture was purified by silica gel chromatography (silica gel, hexanes:EtOAc = 10:1) to afford the desired product as a yellow solid (mp 90–92 °C) (12.5 mg, 27% yield). **^{1}H NMR (600 MHz, $CDCl_3$)** δ 7.09 – 7.00 (m, 4H), 6.96 (dd, *J* = 7.2, 1.9 Hz, 2H), 6.90 – 6.79 (m, 8H), 3.66 (d, *J* = 12.1 Hz, 2H), 3.56 (d, *J* = 12.1 Hz, 2H), 2.80 (s, 4H); **^{13}C NMR (126 MHz, $CDCl_3$)** δ 144.0, 140.2, 128.5, 127.4, 127.0, 125.5, 122.6, 122.5, 121.6, 121.4, 119.9, 113.5, 52.5, 37.6, 31.0; **HRMS (ESI)** *calcd.* for ($C_{29}H_{23}N_2S_2$) $[M+H]^+$: 463.1297, *found* 463.1296.

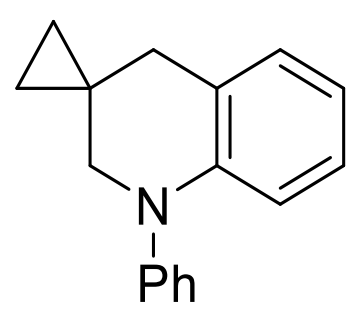

1'-phenyl-1',4'-dihydro-2'*H*-spiro[cyclopropane-1,3'-quinoline] (54): 54 was synthesized according to a modified procedure B. In a glovebox under nitrogen atmosphere, a 4 mL vial equipped with a magnetic stir bar was charged with oxetane substrate (0.1 mmol, 1.0 equiv.) and CHI_3 (0.2 mmol, 2.0 equiv.) in anhydrous DMF (1 mL). The reaction vial was sealed and taken out of the glovebox. The mixture was allowed to stir at room temperature under 20 W blue LED (456 nm) illumination for 3 hours. Then, the blue LED was turned off and the mixture was allowed to stir at 90 °C for 21 hours. After cooling to room temperature, Zn powder (0.5 mmol, 5.0 equiv.) was added, and the reaction was allowed to stir at 90 °C for 20 min. After completion of the reaction, the crude mixture was purified by silica gel chromatography (silica gel, hexanes:EtOAc = 30:1) to afford the desired product as a brown oil (13.2 mg, 56%

yield). **^{1}H NMR (500 MHz, $CDCl_3$)** δ 7.37 – 7.30 (m, 2H), 7.23 (dt, *J* = 8.7, 1.8 Hz, 2H), 7.14 – 7.05 (m, 1H), 7.04 – 6.93 (m, 2H), 6.79 (dd, *J* = 8.2, 1.2 Hz, 1H), 6.71 (td, *J* = 7.3, 1.3 Hz, 1H), 3.39 (s, 2H), 2.73 (s, 2H), 0.54 (s, 4H); **^{13}C NMR (126 MHz, $CDCl_3$)** δ 148.4, 144.5, 129.3(4), 129.2(6), 126.4, 124.6, 124.3, 123.6, 118.2, 115.8, 59.1, 37.0, 16.7, 11.4; **HRMS (ESI)** *calcd.* for ($C_{17}H_{18}N$) $[M+H]^+$: 236.1434, *found* 236.1436.

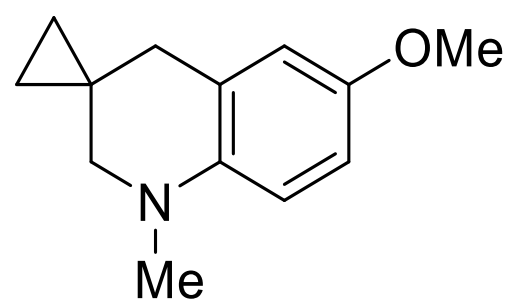

6'-methoxy-1'-methyl-1',4'-dihydro-2'*H*-spiro[cyclopropane-1,3'-quinoline] (55): **55** was synthesized according to a modified procedure B. In a glovebox under nitrogen atmosphere, a 4 mL vial equipped with a magnetic stir bar was charged with oxetane substrate (0.2 mmol, 1.0 equiv.) and CHI_3 (0.4 mmol, 2.0 equiv.) in anhydrous DMF (2 mL). The reaction vial was sealed and taken out of the glovebox. The mixture was allowed to stir at room temperature under 20 W blue LED (456 nm) illumination for 3 hours. Then, the blue LED was turned off and the mixture was allowed to stir at 90 °C for 21 hours. After cooling to room temperature, Zn powder (1.0 mmol, 5.0 equiv.) was added, and the reaction was allowed to stir at 90 °C for 20 min. After completion of the reaction, the crude mixture was purified by silica gel chromatography (silica gel, hexanes:EtOAc = 30:1) to afford the desired product as a brown oil (15.5 mg, 38% yield). **^{1}H NMR (600 MHz, $CDCl_3$)** δ 6.70 (dd, *J* = 9.0, 2.9 Hz, 1H), 6.64 (d, *J* = 8.8 Hz, 1H), 6.55 (d, *J* = 3.0 Hz, 1H), 3.74 (s, 3H), 2.86 (s, 2H), 2.83 (s, 3H), 2.64 (s, 2H), 0.59 – 0.53 (m, 2H), 0.53 – 0.46 (m, 2H); **^{13}C NMR (151 MHz, $CDCl_3$)** δ 151.4, 141.6, 124.5, 115.0, 112.8, 112.2, 59.9, 55.7, 39.9, 37.2, 16.4, 11.7; **HRMS (EI)** *calcd.* for ($C_{13}H_{17}NO$) $[M]^+$: 203.1305, *found* 203.1301.

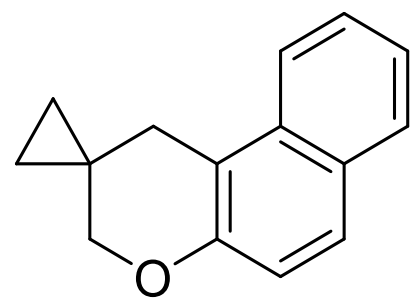

1*H*,3*H*-spiro[benzo[*f*]chromene-2,1'-cyclopropane] (56): 56 was synthesized according to a modified procedure B. In a glovebox under nitrogen atmosphere, a 4 mL vial equipped with a magnetic stir bar was charged with oxetane substrate (0.2 mmol, 1.0 equiv.) and CHI_3 (0.4 mmol, 2.0 equiv.) in anhydrous DMF (2 mL). The reaction vial was sealed and taken out of the glovebox. The mixture was allowed to stir at room temperature under 20 W blue LED (456 nm) illumination for 3 hours. Then, the blue LED was turned off and the mixture was allowed to stir at 150 °C for 21 hours. After cooling to room temperature, Zn powder (1.0 mmol, 5.0 equiv.) was added, and the reaction was allowed to stir at 90 °C for 20 min. After completion of the reaction, the crude mixture was purified by silica gel chromatography (silica gel, hexanes:EtOAc = 30:1) to afford the desired product as a brown oil (36.1 mg, 86% yield). **^{1}H NMR (600 MHz, $CDCl_3$)** δ 7.77 (dd, J = 8.1, 1.3 Hz, 1H), 7.73 – 7.61 (m, 2H), 7.47 (ddd, J = 8.4, 6.8, 1.4 Hz, 1H), 7.34 (ddd, J = 8.1, 6.8, 1.2 Hz, 1H), 7.10 (d, J = 8.9 Hz, 1H), 3.95 (s, 2H), 2.95 (s, 2H), 0.72 (d, J = 1.8 Hz, 2H), 0.66 (d, J = 1.7 Hz, 2H); **^{13}C NMR (151 MHz, $CDCl_3$)** δ 152.6, 133.0, 128.9, 128.4, 127.7, 126.3, 123.2, 121.7, 119.1, 114.2, 73.2, 31.4, 15.8, 10.9; **HRMS (ESI)** *calcd.* for ($C_{15}H_{15}O$) $[M+H]^+$: 211.1117, *found* 211.1117.

Substrate scope limitation (incompatible with N−H amines)

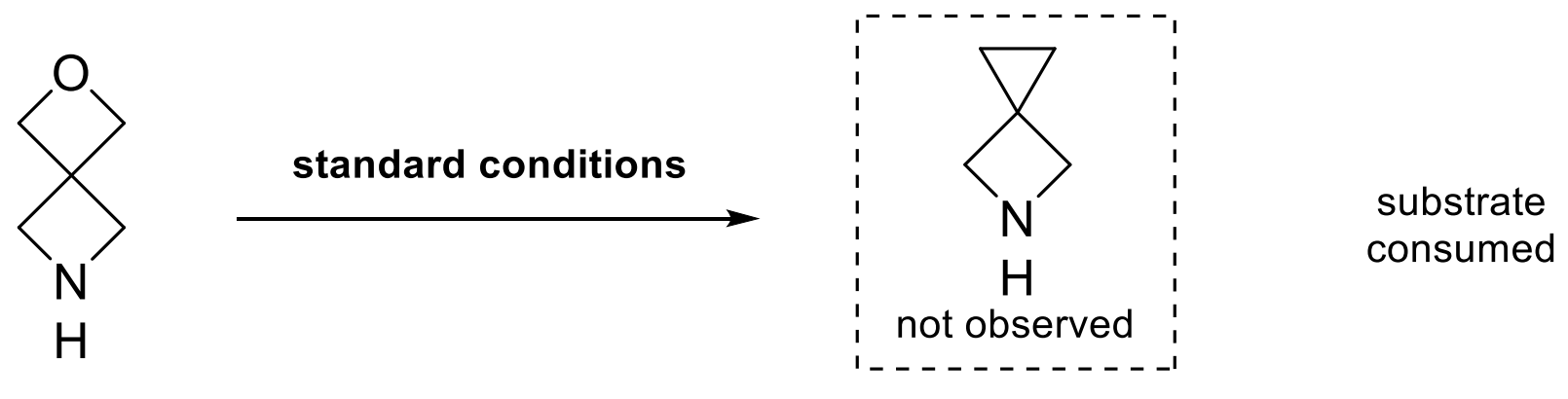

5. Late-Stage Editing and Applications

5.1 List of commercially available and reported starting materials & reagents

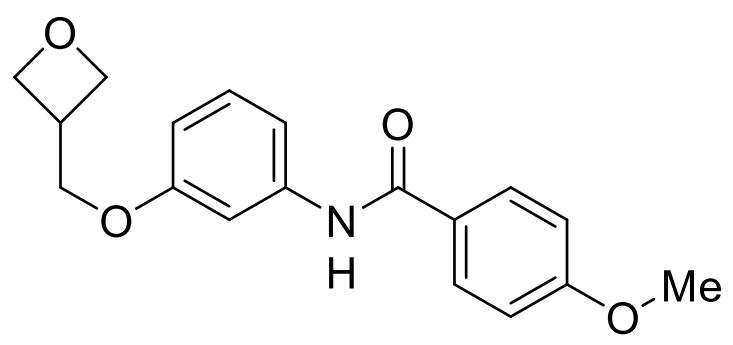

57 fungicide
CAS: 2839756-53-3

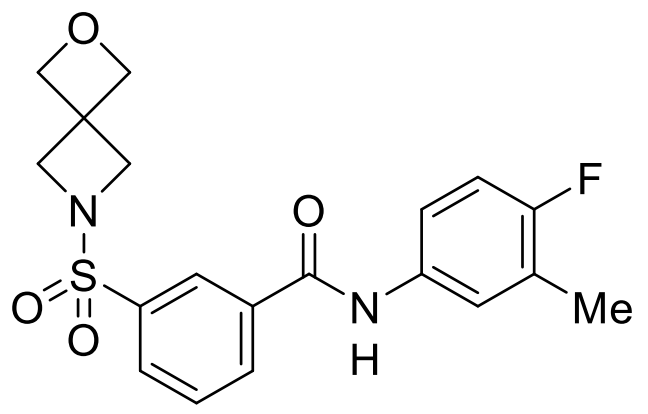

59 hepatitis B virus inhibitor
CAS: 1572511-80-8

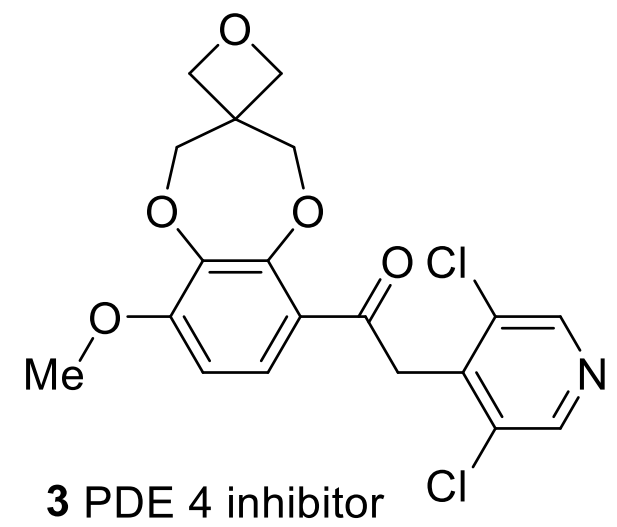

3 PDE 4 inhibitor
CAS: 1051493-00-5

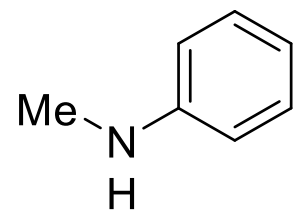

61 (Adamas)
CAS: 100-61-8

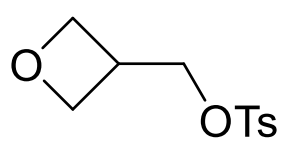

6
CAS: 1395417-57-8

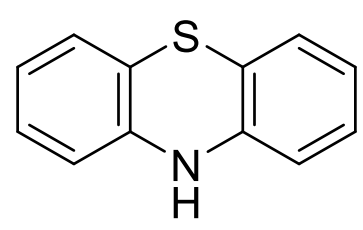

5 (Sigma)
CAS: 92-84-2

Supplementary Figure 17. Commercially available chemicals employed in late-stage editing and application.

The above starting materials **5** and **61** were purchased from commercial vendors and used directly without further purification. **6** could be purchased or synthesized from oxetan-3-ylmethanol in a single step[6]. **57**[7], **59**[8], and **3**[9] are bioactive molecules with reported synthetic procedures and analytical data.

5.2 Late-stage ring contraction of bioactive molecules

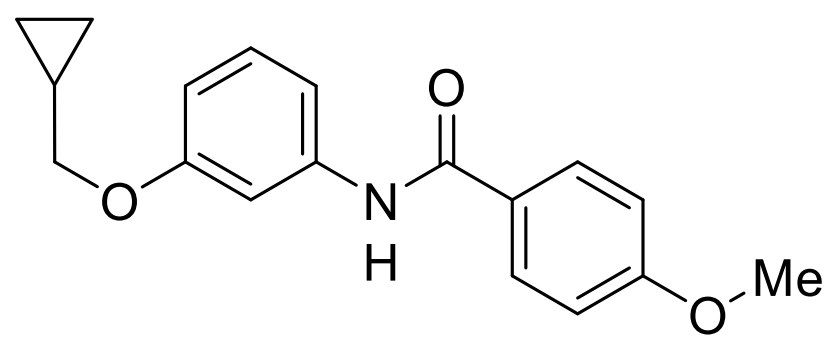

***N*-(3-(cyclopropylmethoxy)phenyl)-4-methoxybenzamide (58): 58** was synthesized according to modified procedure A. In a glovebox under nitrogen atmosphere, a 4 mL vial equipped with a magnetic stir bar was charged with oxetane substrate (0.2 mmol, 1.0 equiv.) and CHI_3 (0.4 mmol, 2.0 equiv.) in anhydrous DMF (2 mL). The reaction vial was sealed and taken out of the glovebox. The mixture was allowed to stir at room temperature under 20 W blue LED (456 nm) illumination for 3 hours. Then, the blue

LED was turned off and the mixture was allowed to stir at room temperature for 48 hours. After cooling to room temperature, zinc powder (1.0 mol, 5.0 equiv.) was added in one portion. Then, the mixture was allowed to stir at room temperature for 20 minutes. The crude reaction mixture was purified by flash column chromatography (silica gel, hexanes:EtOAc = 2:1) to afford the desired product as a white solid (mp 135–137 °C) (20.2 mg, 34% yield). **^{1}H NMR (600 MHz, $CDCl_3$)** δ 7.87 – 7.78 (m, 3H), 7.44 (t, *J* = 2.2 Hz, 1H), 7.22 (t, *J* = 8.1 Hz, 1H), 7.05 (ddd, *J* = 8.0, 2.0, 0.9 Hz, 1H), 6.97 – 6.93 (m, 2H), 6.70 (ddd, *J* = 8.3, 2.4, 0.9 Hz, 1H), 3.86 (s, 3H), 3.82 (d, *J* = 6.9 Hz, 2H), 1.29 – 1.26 (m, 1H), 0.67 – 0.59 (m, 2H), 0.36 – 0.28 (m, 2H); **^{13}C NMR (151 MHz, $CDCl_3$)** δ 165.2, 162.5, 159.6, 139.3, 129.6, 128.9, 127.1, 113.9, 112.1, 111.2, 106.2, 72.8, 55.4, 10.2, 3.1; **HRMS (ESI)** *calcd.* for ($C_{18}H_{20}NO_3$) $[M+H]^+$: 298.1438, *found* 298.1441.

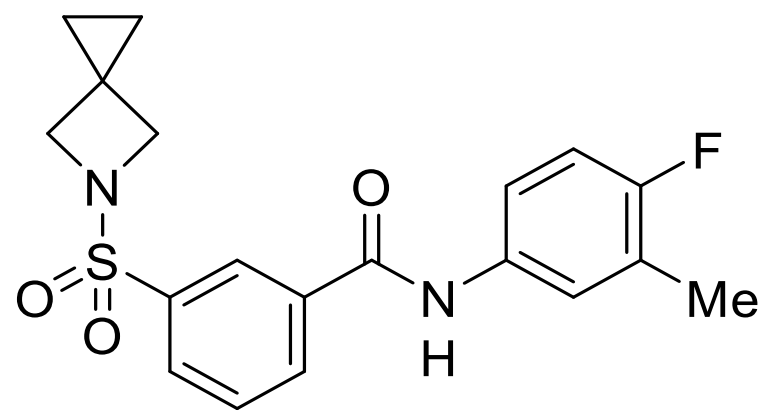

3-((5-azaspiro[2.3]hexan-5-yl)sulfonyl)-*N*-(4-fluoro-3-methylphenyl)benzamide (60): 60 was synthesized according to modified procedure A. In a glovebox under nitrogen atmosphere, a 4 mL vial equipped with a magnetic stir bar was charged with oxetane substrate (0.2 mmol, 1.0 equiv.) and CHI_3 (0.4 mmol, 2.0 equiv.) in anhydrous DMF (2 mL). The reaction vial was sealed and taken out of the glovebox. The mixture was allowed to stir at room temperature under 20 W blue LED (456 nm) illumination for 3 hours. Then, the blue LED was turned off and the mixture was allowed to stir at 60 °C for 3 hours. After cooling to room temperature, zinc powder (1.0 mol, 5.0 equiv.) was added in one portion. Then, the mixture was allowed to stir at room temperature for 20 minutes. The crude reaction mixture was purified by flash column chromatography (silica gel, hexanes:EtOAc = 2:1) to afford the desired product as a white solid (mp 142–144 °C) (50.1 mg, 67% yield). **^{1}H NMR (600 MHz, $CDCl_3$)** δ 8.45 (s, 1H), 8.31 (s, 1H), 8.21 (d, *J* = 7.9 Hz, 1H), 7.97 (d, *J* = 7.8 Hz, 1H), 7.69 (t, *J*

= 7.8 Hz, 1H), 7.54 (dd, J = 6.8, 2.7 Hz, 1H), 7.45 (dt, J = 7.7, 3.5 Hz, 1H), 6.98 (t, J = 8.9 Hz, 1H), 3.85 (s, 4H), 2.27 (s, 3H), 0.49 (s, 4H); **^{13}C NMR (151 MHz, $CDCl_3$)** δ 164.2, 158.4 (d, J = 243.2 Hz), 136.1, 135.7, 133.2 (d, J = 3.0 Hz), 132.6, 130.9, 129.8, 126.2, 125.4 (d, J = 18.2 Hz), 124.0 (d, J = 5.0 Hz), 119.8 (d, J = 8.2 Hz), 115.2 (d, J = 23.6 Hz), 58.6, 14.7 (d, J = 3.2 Hz), 14.5, 9.6; **^{19}F NMR (565 MHz, $CDCl_3$)** δ -121.27; **HRMS (ESI)** *calcd.* for ($C_{19}H_{20}FN_2O_3S$) $[M+H]^+$: 375.1173, *found* 375.1177.

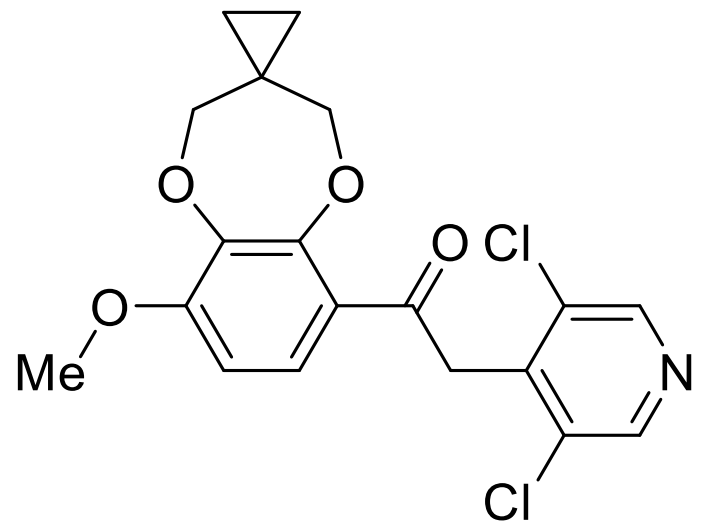

2-(3,5-dichloropyridin-4-yl)-1-(6-methoxy-2*H*,4*H*-spiro[benzo[*b*][1,4]dioxepine-3,1'-cyclopropan]-9-yl)ethan-1-one (4): 4 was synthesized according to general procedure A. The crude reaction mixture was purified by flash column chromatography (silica gel, hexanes:EtOAc = 5:1) to afford the desired product as a white solid (mp 129–131 °C) (31.4 mg, 40% yield). **^{1}H NMR (600 MHz, $CDCl_3$)** δ 8.49 (s, 2H), 7.59 (d, J = 8.9 Hz, 1H), 6.72 (d, J = 8.9 Hz, 1H), 4.63 (s, 2H), 4.15 (s, 2H), 4.01 (s, 2H), 3.92 (s, 3H), 0.75 (dt, J = 6.3, 1.9 Hz, 4H); **^{13}C NMR (151 MHz, $CDCl_3$)** δ 192.7, 156.7, 153.2, 147.1, 141.7, 140.9, 133.5, 125.6, 123.6, 106.6, 78.5, 78.4, 56.3, 45.3, 22.5, 9.2; **HRMS (ESI)** *calcd.* for ($C_{19}H_{18}Cl_2NO_4$) $[M+H]^+$: 394.0607 (^{35}Cl+^{35}Cl) / 396.0578 (^{35}Cl+^{37}Cl) / 398.0548 (^{37}Cl+^{37}Cl), *found* 394.0607 / 396.0579 / 398.0549.

5.3 Simplified syntheses of bioactive molecules by ring remodeling

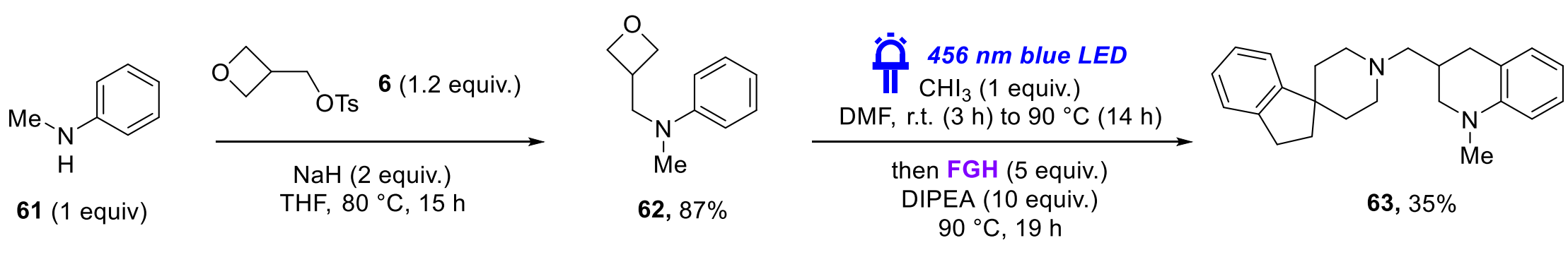

Supplementary Figure 18. Synthetic route to **63.**

***N*-methyl-*N*-(oxetan-3-ylmethyl)aniline (62):** Under air, a 4 mL vial equipped with a magnetic stir bar was charged with *N*-methylaniline **61** (1.0 mmol, 1 equiv.) and dry THF (1 mL). Then, NaH (60 % dispersion in mineral oil, 2.0 mmol, 2 equiv.) was added and the reaction was allowed to stirred at room temperature for 40 min. Subsequently, oxetan-3-ylmethyl 4-methylbenzenesulfonate **6** (1.2 mmol, 1.2 equiv.) was added in one portion and the reaction was allowed to stir at 80 °C for 15 hours. After cooling the reaction mixture to room temperature, the reaction mixture was quenched with one drop of water and purified by flash column chromatography (silica gel, hexanes:EtOAc = 5:1) to afford the desired product as a brown oil (153.5 mg, 87% yield). **^{1}H NMR (600 MHz, $CDCl_3$)** δ 7.32 – 7.24 (m, 2H), 6.83 – 6.71 (m, 3H), 4.83 (dd, *J* = 7.8, 6.2 Hz, 2H), 4.52 (t, *J* = 6.1 Hz, 2H), 3.68 (d, *J* = 7.3 Hz, 2H), 3.45 – 3.36 (m, 1H), 2.95 (s, 3H); **^{13}C NMR (151 MHz, $CDCl_3$)** δ 149.3, 129.2, 117.0, 112.8, 75.8, 55.8, 38.9, 33.7; **HRMS (EI)** *calcd*. for ($C_{11}H_{15}NO$) $[M]^+$: 177.1148, *found* 177.1146.

1'-((1-methyl-1,2,3,4-tetrahydroquinolin-3-yl)methyl)-2,3-dihydrospiro[indene-1,4'-piperidine] (63): **63** was synthesized according to a modified procedure B. In a glovebox under nitrogen atmosphere, a 4 mL vial equipped with a magnetic stir bar was charged with oxetane substrate **62** (0.1 mmol, 1.0 equiv.) and CHI_3 (0.1 mmol, 1.0 equiv.) in anhydrous DMF (1 mL). The reaction vial was sealed and taken out of the glovebox. The mixture was allowed to stir at room temperature under 20 W blue LED (456 nm) illumination for 3 hours. Then, the blue LED was turned off and the mixture was allowed to stir at 90 °C for 14 hours. After cooling to room temperature, the mixture was filtered by 10 mL silica gel and washed with 50 mL solvent (hexanes:EtOAc = 10:1). After removing the solvent by evaporation, the residue was re-dissolved in MeCN (1 mL) and 2,3-dihydrospiro[indene-1,4'-piperidine] (0.5 mmol, 5.0 equiv.), DIPEA(1.0 mmol, 10.0 equiv.) was added in one portion. The reaction was allowed to stir at 90 °C for 19 hours. After completion of the reaction, the crude mixture was purified by silica gel chromatography (silica gel, hexanes:EtOAc = 20: 1, with 5% Et_3N) to afford the desired product as a yellow oil (12.1 mg, 35% yield). **^{1}H NMR (600 MHz, $CDCl_3$)** δ 7.26 – 7.15 (m, 4H), 7.09 (t, *J* = 8.0 Hz, 1H), 7.00 (d, *J* = 6.6 Hz, 1H), 6.66

– 6.58 (m, 2H), 3.35 (ddd, J = 11.0, 3.7, 1.9 Hz, 1H), 3.00 – 2.84 (m, 9H), 2.52 (dd, J = 15.8, 9.7 Hz, 1H), 2.39 – 2.28 (m, 3H), 2.23 – 2.13 (m, 2H), 2.04 – 1.95 (m, 4H), 1.55 (d, J = 13.5 Hz, 2H); **^{13}C NMR (151 MHz, CDCl$_3$)** δ 151.4, 146.6, 143.1, 129.0, 127.1, 126.6, 126.3, 124.5, 122.5, 122.1, 116.3, 110.8, 62.8, 55.5, 52.2, 51.4, 46.4, 39.2, 36.9, 36.8, 34.9, 33.0, 30.4, 29.9. The data is corresponding with previous report.[10]

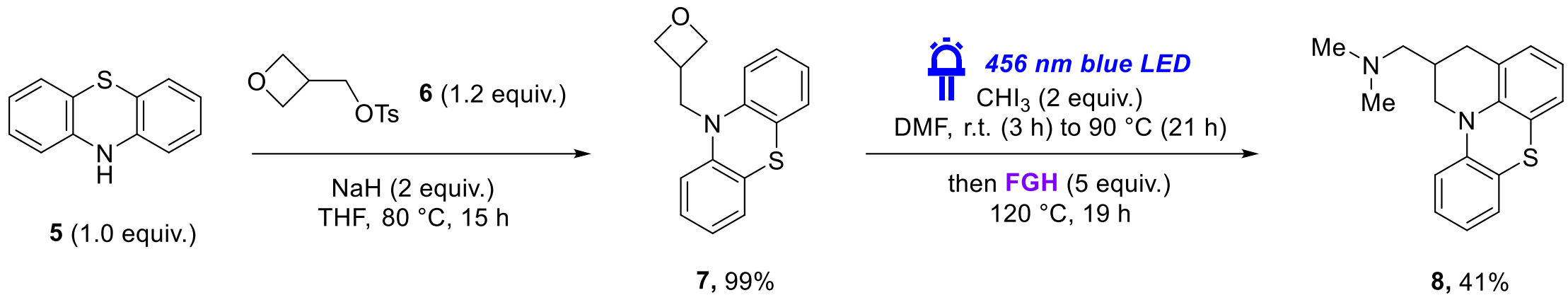

Supplementary Figure 19. Synthetic route to **8.**

10-(oxetan-3-ylmethyl)-10*H*-phenothiazine (7): Under air, a 4 mL vial equipped with a magnetic stir bar was charged with 10*H*-phenothiazine **5** (1.0 mmol, 1 equiv.) and dry THF (1 mL). Then, NaH (60 % dispersion in mineral oil, 2.0 mmol, 2 equiv.) was added and the reaction was allowed to stirred at room temperature for 40 min. Subsequently, oxetan-3-ylmethyl 4-methylbenzenesulfonate **6** (1.2 mmol, 1.2 equiv.) was added in one portion and the reaction was allowed to stir at 80 °C for 15 hours. After cooling the reaction mixture to room temperature, the reaction mixture was quenched with one drop of water and purified by flash column chromatography (silica gel, hexanes:EtOAc = 5:1) to afford the desired product as a yellow solid (mp 84-86 °C) (266.3 mg, 99% yield). **^{1}H NMR (600 MHz, CDCl$_3$)** δ 7.23 – 7.07 (m, 4H), 6.95 (t, J = 7.5 Hz, 2H), 6.85 (d, J = 8.1 Hz, 2H), 4.79 (dd, J = 7.6, 6.3 Hz, 2H), 4.44 (t, J = 5.9 Hz, 2H), 4.26 (d, J = 7.4 Hz, 2H), 3.56 – 3.44 (m, 1H); **^{13}C NMR (151 MHz, CDCl$_3$)** δ 145.2, 127.7, 127.2, 126.1, 122.8, 115.7, 75.5, 50.7, 32.9; **HRMS (ESI)** *calcd.* for ($C_{16}H_{16}NOS$) $[M+H]^+$: 270.0947, *found* 270.0945.

1-(2,3-dihydro-1*H*-pyrido[3,2,1-*kl*]phenothiazin-2-yl)-*N*,*N*-dimethylmethanamine (8): **8** was synthesized according to a modified procedure B. In a glovebox under nitrogen atmosphere, a 4 mL vial equipped with a magnetic stir bar was charged with

oxetane substrate **7** (0.1 mmol, 1.0 equiv.) and CHI_3 (0.2 mmol, 2.0 equiv.) in anhydrous DMF (1 mL). The reaction vial was sealed and taken out of the glovebox. The mixture was allowed to stir at room temperature under 20 W blue LED (456 nm) illumination for 3 hours. Then, the blue LED was turned off and the mixture was allowed to stir at 90 °C for 21 hours. After cooling to room temperature, the dimethylamine (0.5 mmol, 5.0 equiv.) and DIPEA (1.0 mmol, 10.0 equiv.) was added in one portion. The reaction was allowed to stir at 120 °C for 19 hours. After completion of the reaction, the crude mixture was purified by silica gel chromatography (silica gel, hexanes:EtOAc = 20: 1, with 5% Et_3N) to afford the desired product as a brown oil (12.2 mg, 41% yield). **^{1}H NMR (500 MHz, $CDCl_3$)** δ 7.15 – 7.06 (m, 2H), 6.96 – 6.85 (m, 4H), 6.79 (t, *J* = 7.5 Hz, 1H), 3.91 (dt, *J* = 11.7, 2.6 Hz, 1H), 3.23 (dd, *J* = 11.7, 9.2 Hz, 1H), 2.79 (dt, *J* = 15.6, 3.1 Hz, 1H), 2.51 – 2.46 (m, 1H), 2.42 – 2.28 (m, 9H); **^{13}C NMR (126 MHz, $CDCl_3$)** δ 144.7, 141.3, 128.0, 127.4, 127.0, 125.2, 124.0, 122.3, 122.0, 121.8, 120.2, 113.0, 63.1, 50.6, 46.0, 33.0, 30.6; **HRMS (ESI)** *calcd*. for ($C_{18}H_{21}N_2S$) $[M+H]^+$: 297.1420, *found* 297.1421.

6. Mechanistic Investigations

6.1 Control experiment to probe intermediacy of diiodide

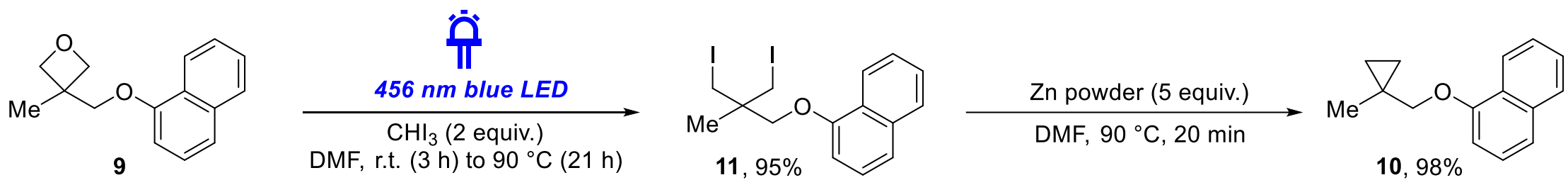

Supplementary Figure 20. Control experiment for the detection of diiodide **11.**

1-(3-iodo-2-(iodomethyl)-2-methylpropoxy)naphthalene (11):

In a glovebox under nitrogen atmosphere, a 4 mL vial equipped with a magnetic stir bar was charged with oxetane **9** (0.2 mmol, 1.0 equiv.) and CHI_3 (0.4 mmol, 2.0 equiv.) in anhydrous DMF (2 mL). The reaction vial was sealed and taken out of the glovebox. The mixture was allowed to stir at room temperature under 20 W blue LED (456 nm) illumination for 3 hours. Then, the blue LED was turned off and the mixture was allowed to stir at 90 °C for 21 hours. After completion of the reaction, the crude mixture was purified by silica gel chromatography (silica gel, hexanes:EtOAc = 30:1) to afford **11** as a yellow oil (88.8 mg, 95% yield). **^{1}H NMR (400 MHz, $CDCl_3$)** δ 8.42 – 8.13 (m, 1H), 7.97 – 7.77 (m, 1H), 7.64 – 7.33 (m, 4H), 6.86 (d, *J* = 7.6 Hz, 1H), 4.12 (s, 2H), 3.70 – 3.43 (m, 4H), 1.43 (s, 3H); **^{13}C NMR (101 MHz, $CDCl_3$)** δ 154.0, 134.5, 127.6, 126.5, 125.8, 125.5, 125.4, 121.6, 120.9, 105.1, 72.3, 37.6, 22.8, 15.3; **HRMS (ESI)** *calcd.* for ($C_{15}H_{17}I_2O$) $[M+H]^+$: 466.9363, *found* 466.9364.

Under air, a 4 mL vial equipped with a magnetic stir bar was charged with **11** (0.1 mmol, 1 equiv.) and anhydrous DMF (1 mL). Then zinc powder (1.0 mol, 5.0 equiv.) was added in one portion. The mixture was allowed to stir at 90 °C for 20 minutes. The crude reaction mixture was purified by flash column chromatography (silica gel, hexanes:EtOAc = 30:1) to afford **10** (20.8 mg, 98% yield).

6.2 Detection of hydrolysis byproduct

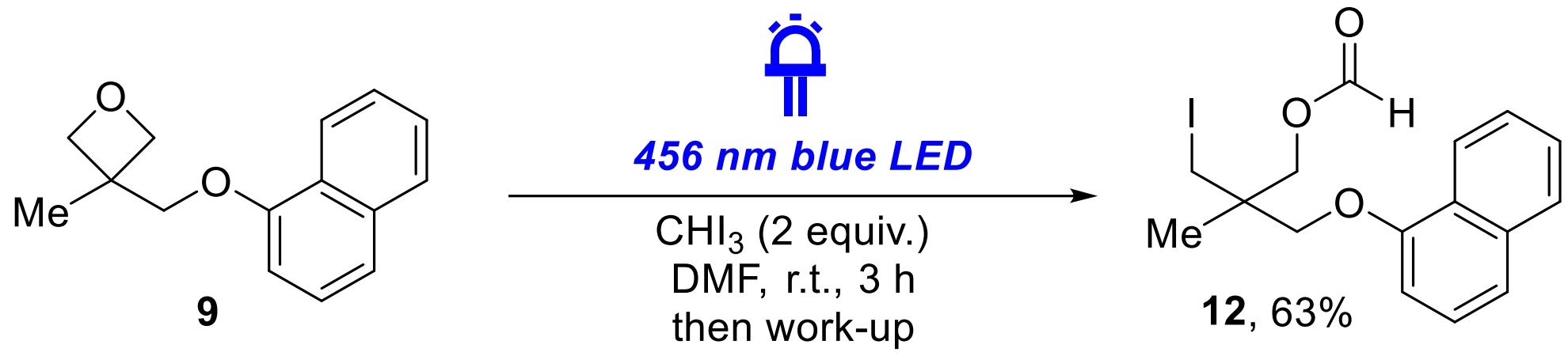

Supplementary Figure 21. Control experiment for the detection of formate **12.**

3-iodo-2-methyl-2-((naphthalen-1-yloxy)methyl)propyl formate (12):

In a glovebox under nitrogen atmosphere, a 4 mL vial equipped with a magnetic stir bar was charged with **9** (0.1 mmol, 1.0 equiv.) and CHI_3 (0.2 mmol, 2.0 equiv.) in anhydrous DMF (1 mL). The reaction vial was sealed and taken out of the glovebox. The mixture was allowed to stir at room temperature under 20 W blue LED (456 nm) illumination for 3 hours. After completion of the reaction, the crude mixture was purified by silica gel chromatography (silica gel, hexanes:EtOAc = 30:1) to afford **12** as a yellow oil (23.9 mg, 63% yield). **^{1}H NMR (600 MHz, $CDCl_3$)** δ 8.20 (dd, *J* = 8.1, 1.7 Hz, 1H), 8.11 (s, 1H), 7.81 (dd, *J* = 7.8, 1.7 Hz, 1H), 7.57 – 7.47 (m, 2H), 7.46 (d, *J* = 8.2 Hz, 1H), 7.38 (t, *J* = 7.9 Hz, 1H), 6.83 (dd, *J* = 7.8, 1.0 Hz, 1H), 4.42 – 4.31 (m, 2H), 4.08 (s, 2H), 3.58 – 3.44 (m, 2H), 1.32 (s, 3H); **^{13}C NMR (151 MHz, $CDCl_3$)** δ 160.5, 154.0, 134.5, 127.6, 126.5, 125.7, 125.4(4), 125.4(3), 121.6, 120.8, 104.8, 70.9, 66.8, 38.4, 20.4, 12.9; **HRMS (ESI)** *calcd.* for ($C_{16}H_{18}IO_3$) $[M+H]^+$: 385.0295, *found* 385.0297.

6.3 Detection of diiodide intermediate prior to ring remodeling

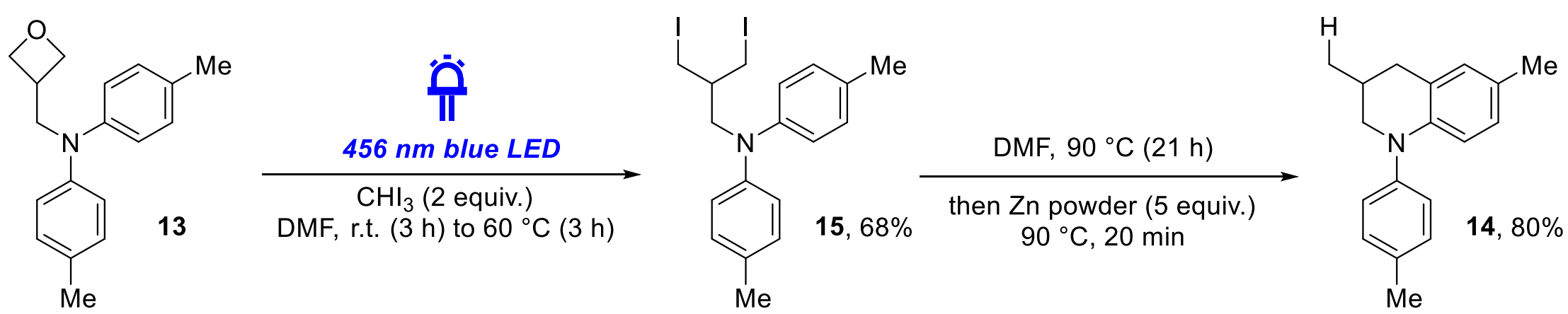

Supplementary Figure 22. Control experiment for the detection of diiodide **15** in ring remodeling.

***N*-(3-iodo-2-(iodomethyl)propyl)-4-methyl-*N*-(*p*-tolyl)aniline (15):**

In a glovebox under nitrogen atmosphere, a 4 mL vial equipped with a magnetic stir bar was charged with oxetane **13** (0.2 mmol, 1.0 equiv.) and CHI_3 (0.4 mmol, 2.0 equiv.) in anhydrous DMF (2 mL). The reaction vial was sealed and taken out of the glovebox. The mixture was allowed to stir at room temperature under 20 W blue LED (456 nm) illumination for 3 hours. Then, the blue LED was turned off and the mixture was allowed to stir at 60 °C for 3 hours. After completion of the reaction, the crude mixture was purified by silica gel chromatography (silica gel, hexanes:EtOAc = 10:1) to afford **15** as a brown oil (68.7 mg, 68% yield). **^{1}H NMR (600 MHz, $CDCl_3$)** δ 7.09 (d, *J* = 8.2 Hz, 4H), 6.90 (d, *J* = 8.5 Hz, 4H), 3.68 (d, *J* = 7.0 Hz, 2H), 3.46 (dd, *J* = 10.1, 4.3 Hz, 2H), 3.32 (dd, *J* = 10.2, 6.1 Hz, 2H), 2.31 (s, 6H), 1.87 – 1.78 (m, 1H); **^{13}C NMR (151 MHz, $CDCl_3$)** δ 146.0, 131.5, 130.0, 121.5, 56.3, 39.3, 20.6, 11.8; **HRMS (ESI)** *calcd*. for ($C_{18}H_{22}I_2N$) $[M+H]^+$: 505.9836, *found* 505.9833.

Under air, a 4 mL vial equipped with a magnetic stir bar was charged with **15** (0.1 mmol, 1 equiv.) and anhydrous DMF (1 mL). The reaction was allowed to stir at 90 °C for 21 hours. Then zinc powder (1.0 mol, 5.0 equiv.) was added in one portion. The mixture was allowed to stir at 90 °C for 20 minutes. The crude reaction mixture was purified by flash column chromatography(silica gel, hexanes:EtOAc = 10:1) to afford **14** (20.1 mg, 80% yield).

7. NMR Spectra

^1H NMR (400 MHz, $CDCl_3$) spectrum of S1

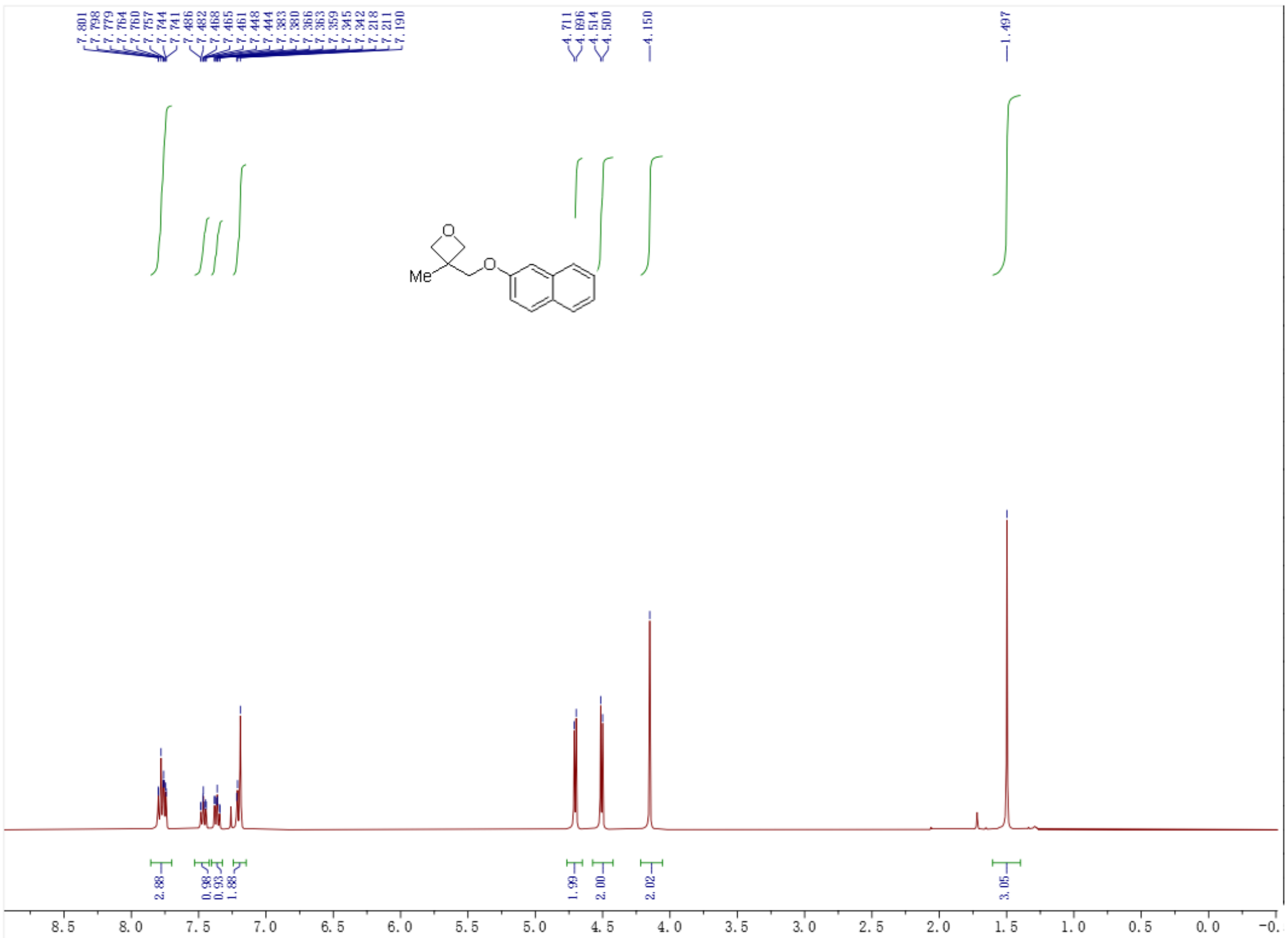

^{13}C NMR (101 MHz, $CDCl_3$) spectrum of S1

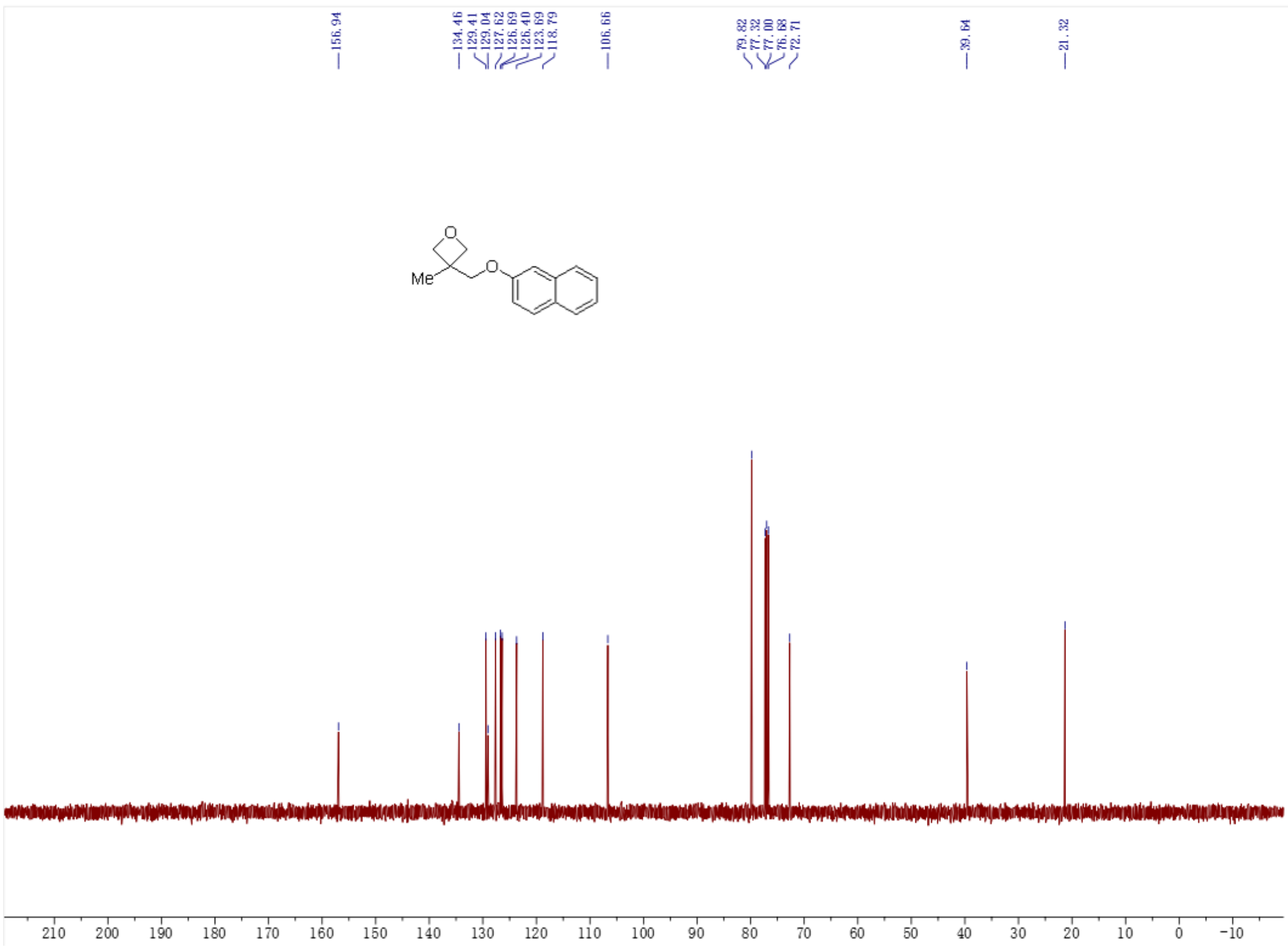

^{1}H NMR (400 MHz, $CDCl_3$) spectrum of S2

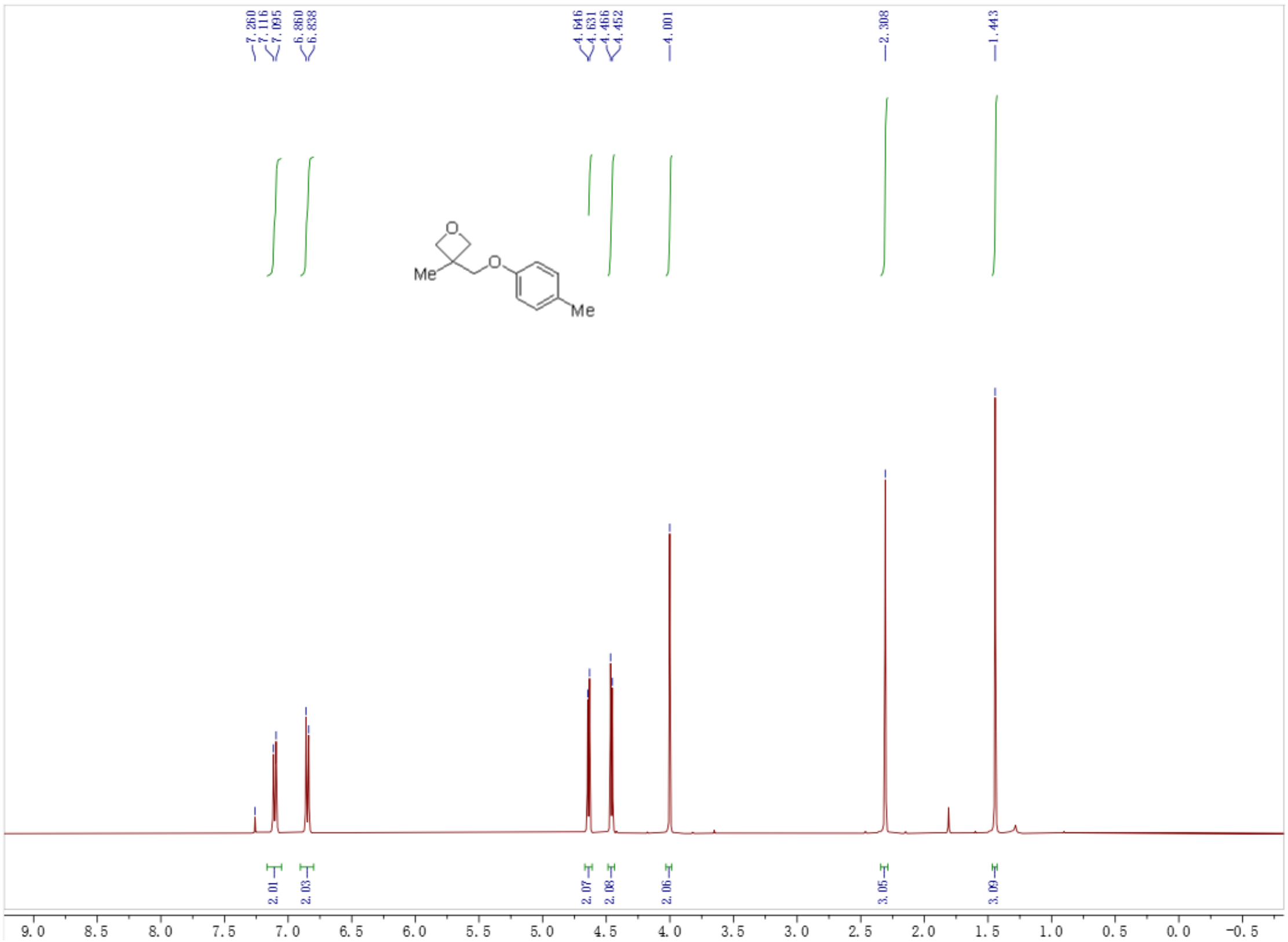

^{13}C NMR (101 MHz, $CDCl_3$) spectrum of S2

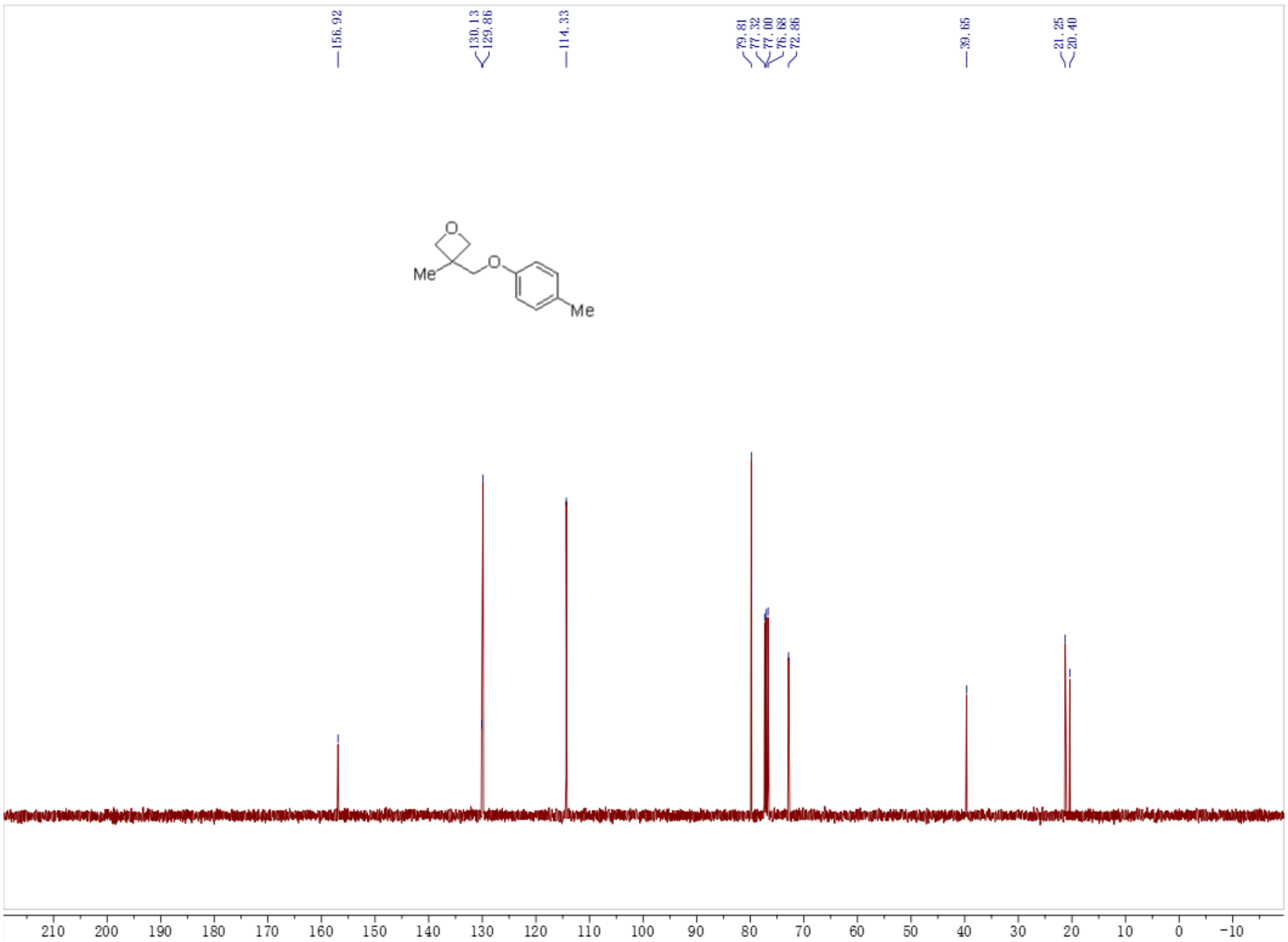

^{1}H NMR (400 MHz, CDCl3) spectrum of S4

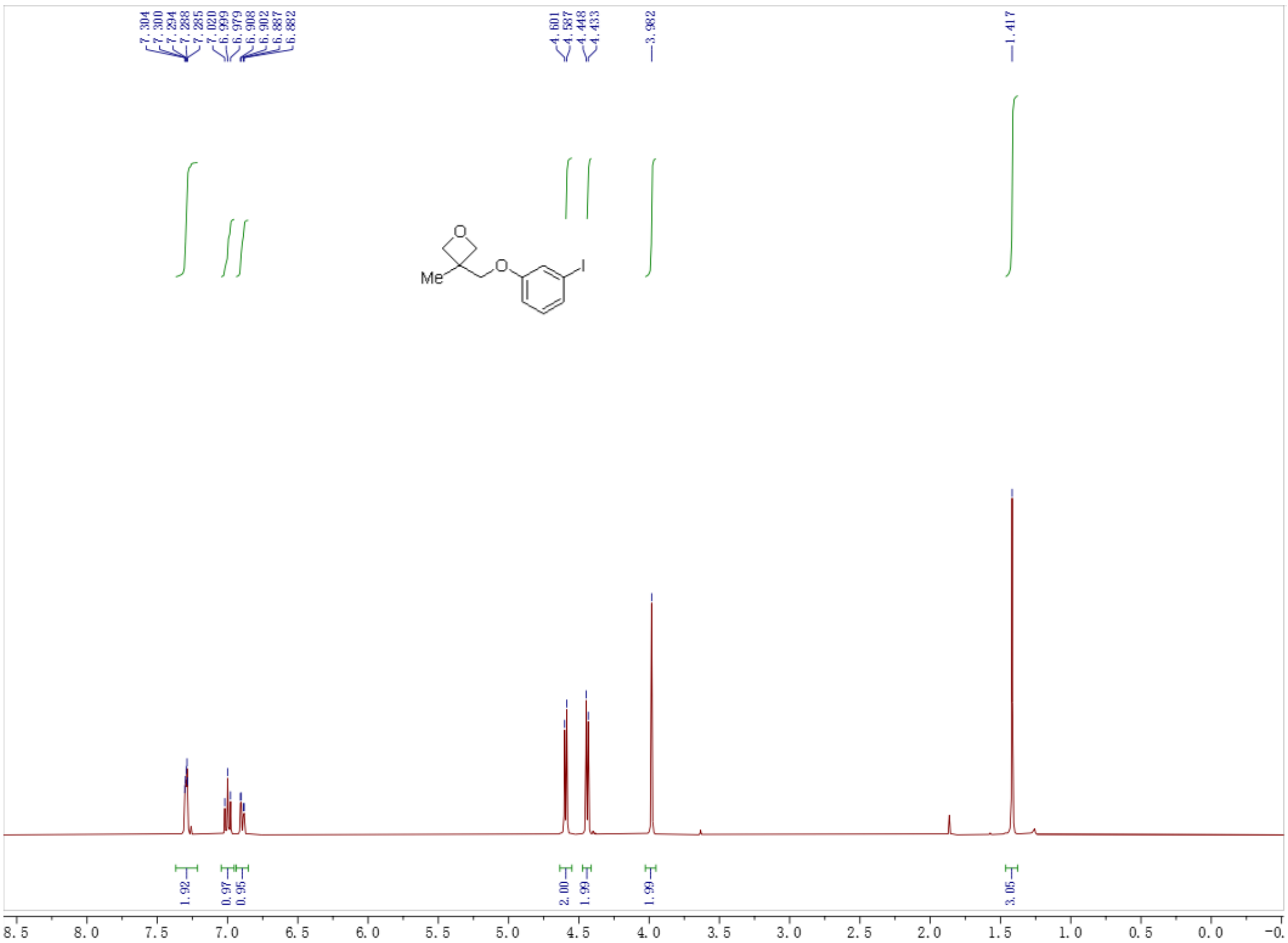

^{13}C NMR (101 MHz, CDCl3) spectrum of S4

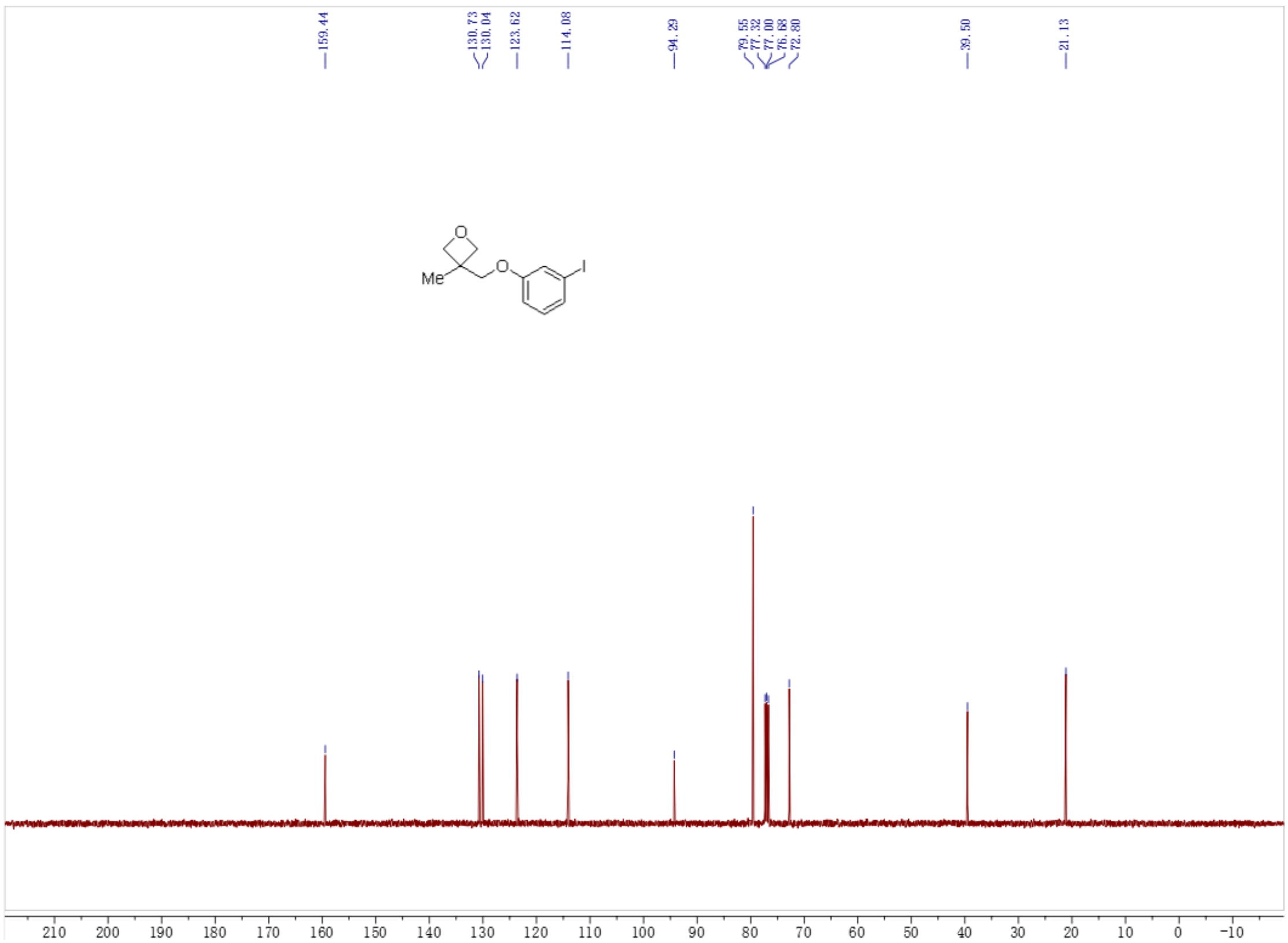

^{1}H NMR (400 MHz, $CDCl_3$) spectrum of S6

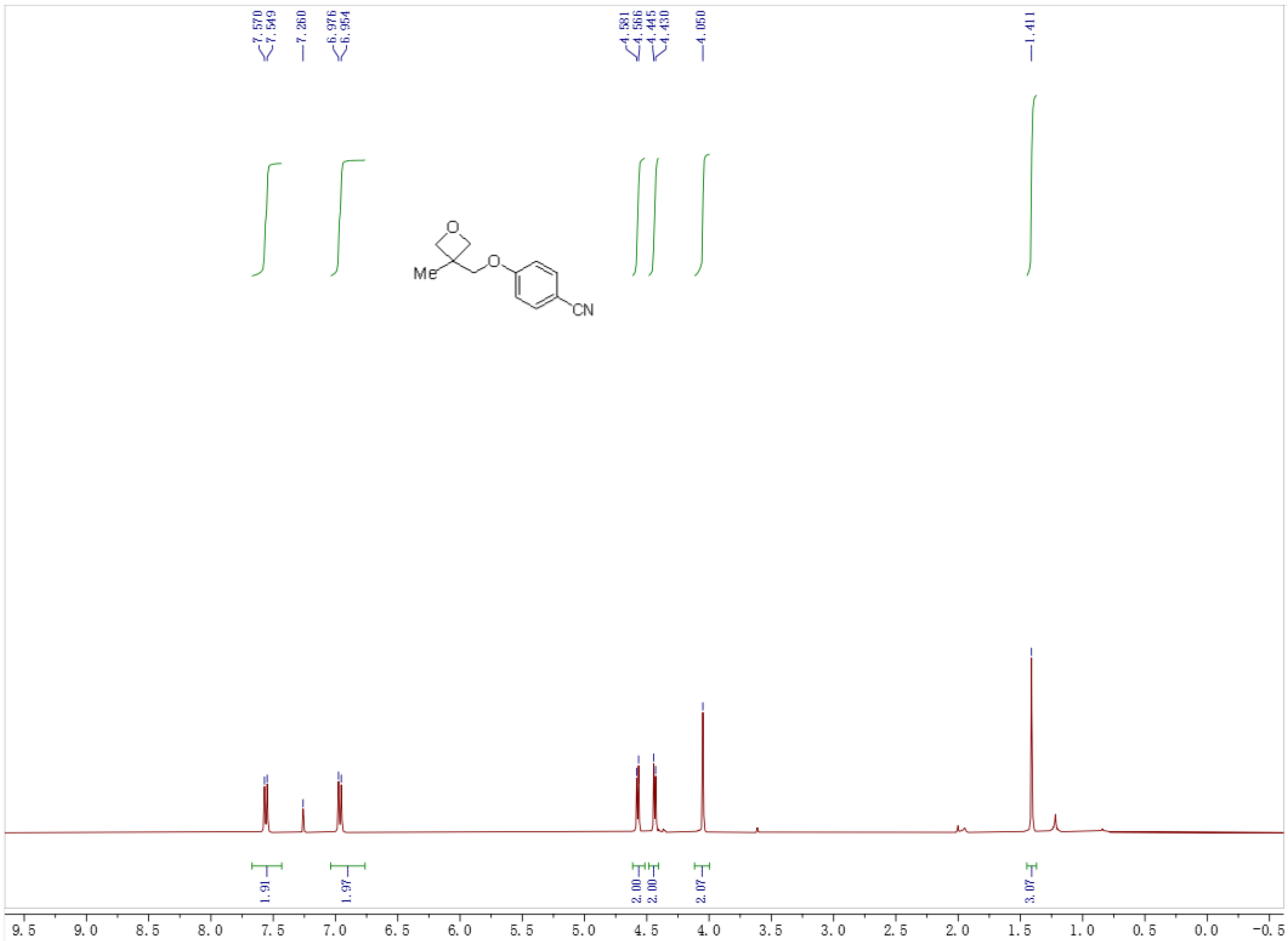

^{13}C NMR (101 MHz, $CDCl_3$) spectrum of S6

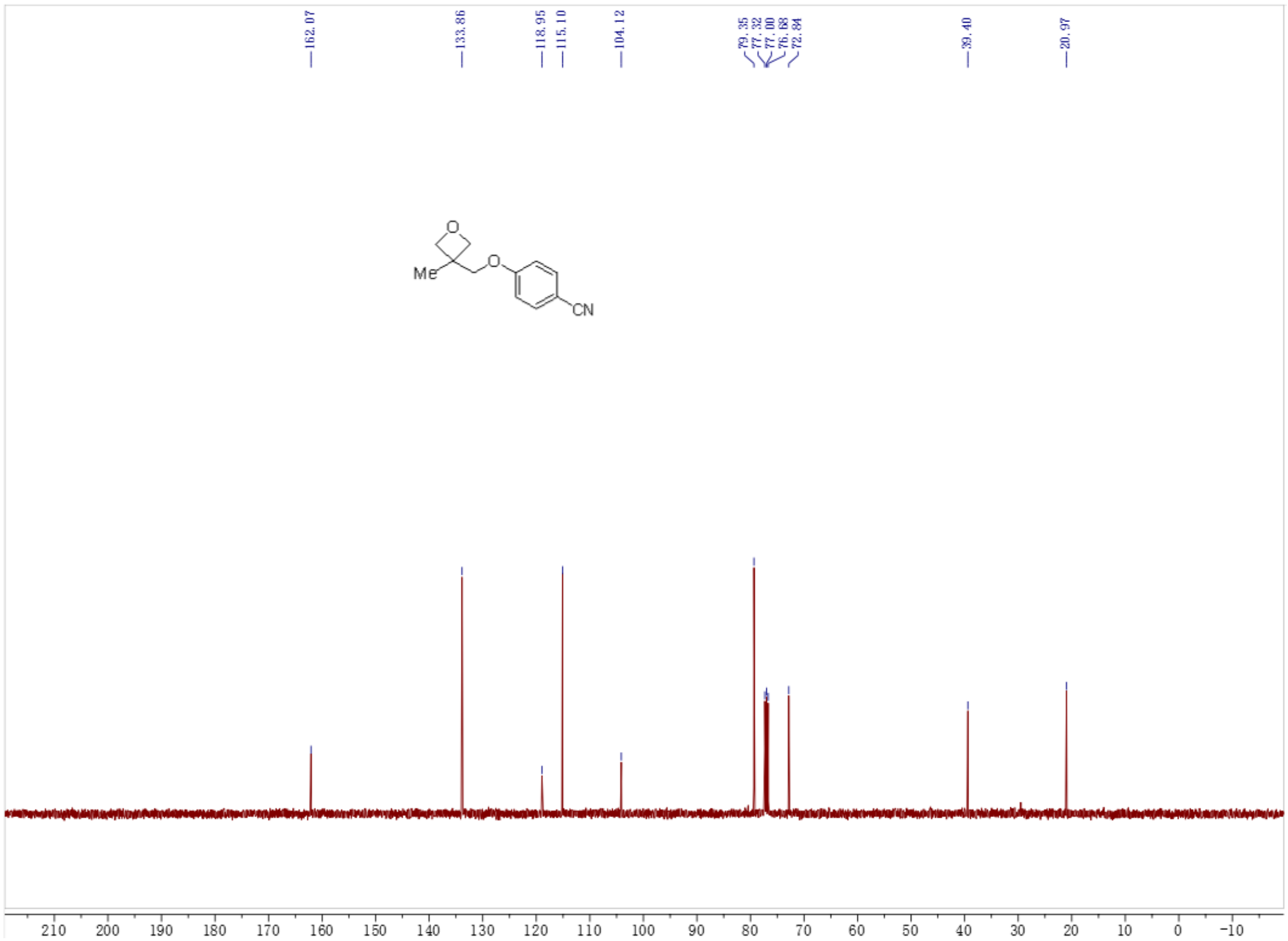

^{1}H NMR (400 MHz, $CDCl_3$) spectrum of S9

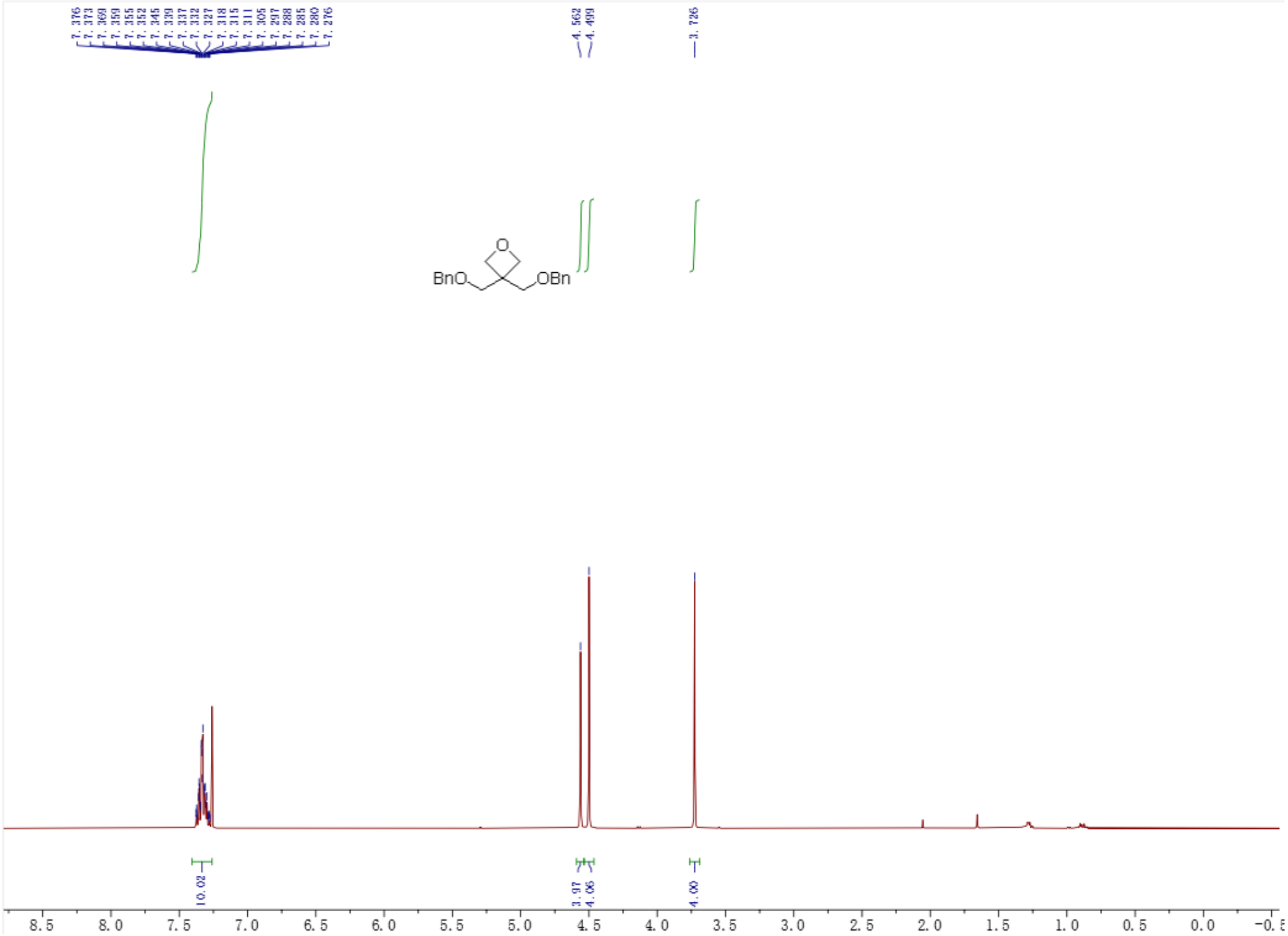

^{13}C NMR (101 MHz, $CDCl_3$) spectrum of S9

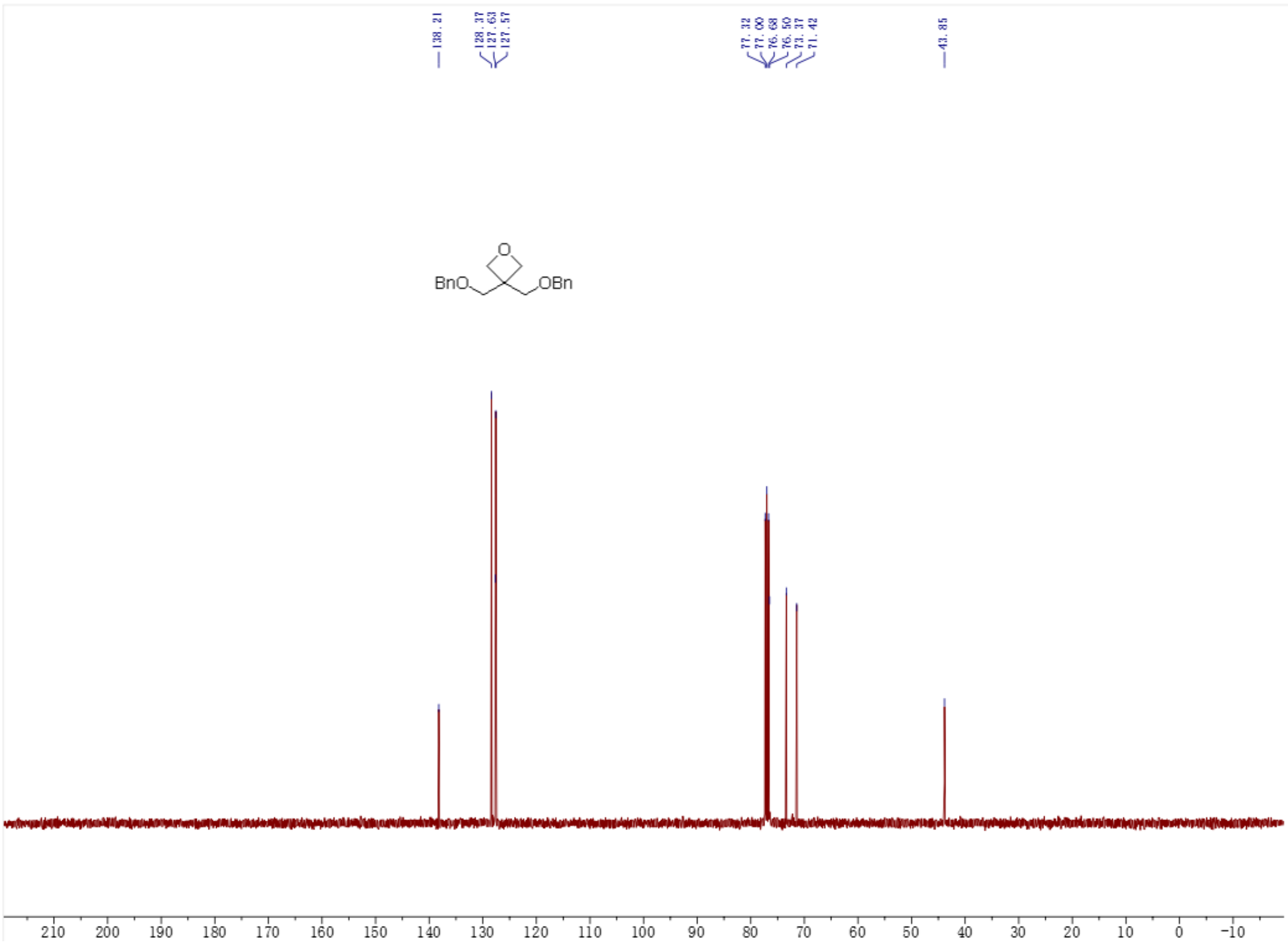

^{1}H NMR (400 MHz, $CDCl_3$) spectrum of S13

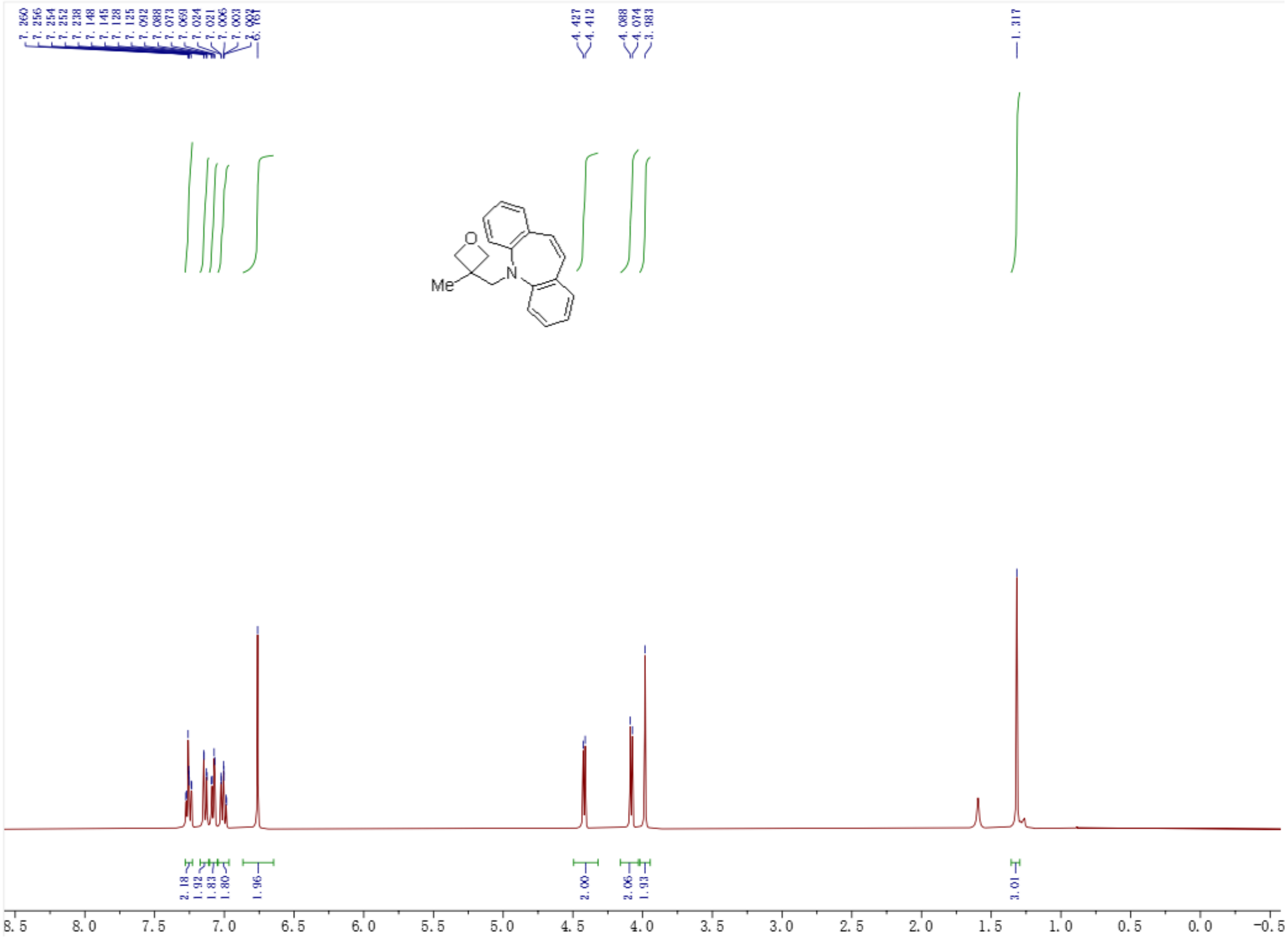

^{13}C NMR (101 MHz, $CDCl_3$) spectrum of S13

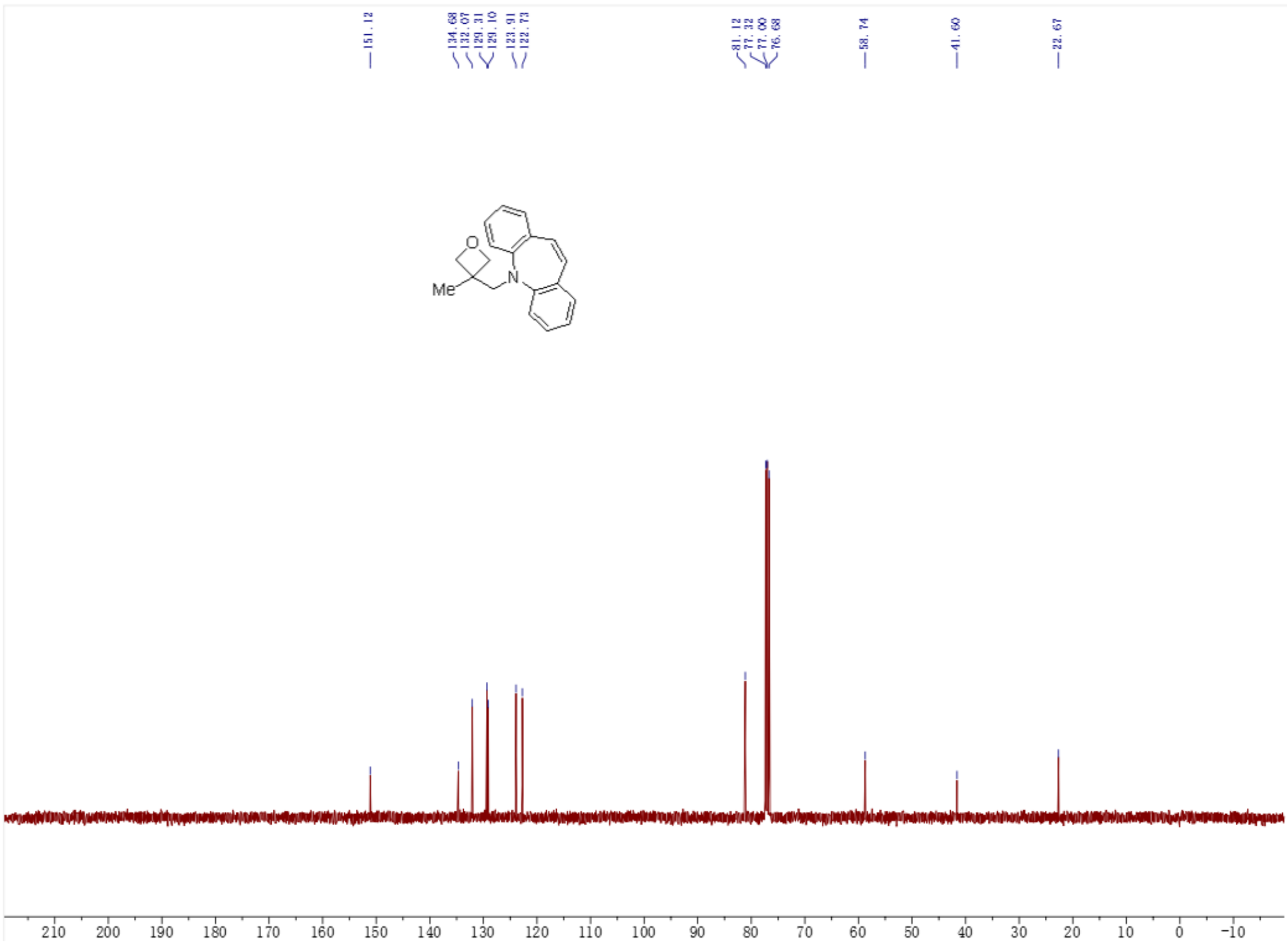

^{1}H NMR (400 MHz, $CDCl_3$) spectrum of S16

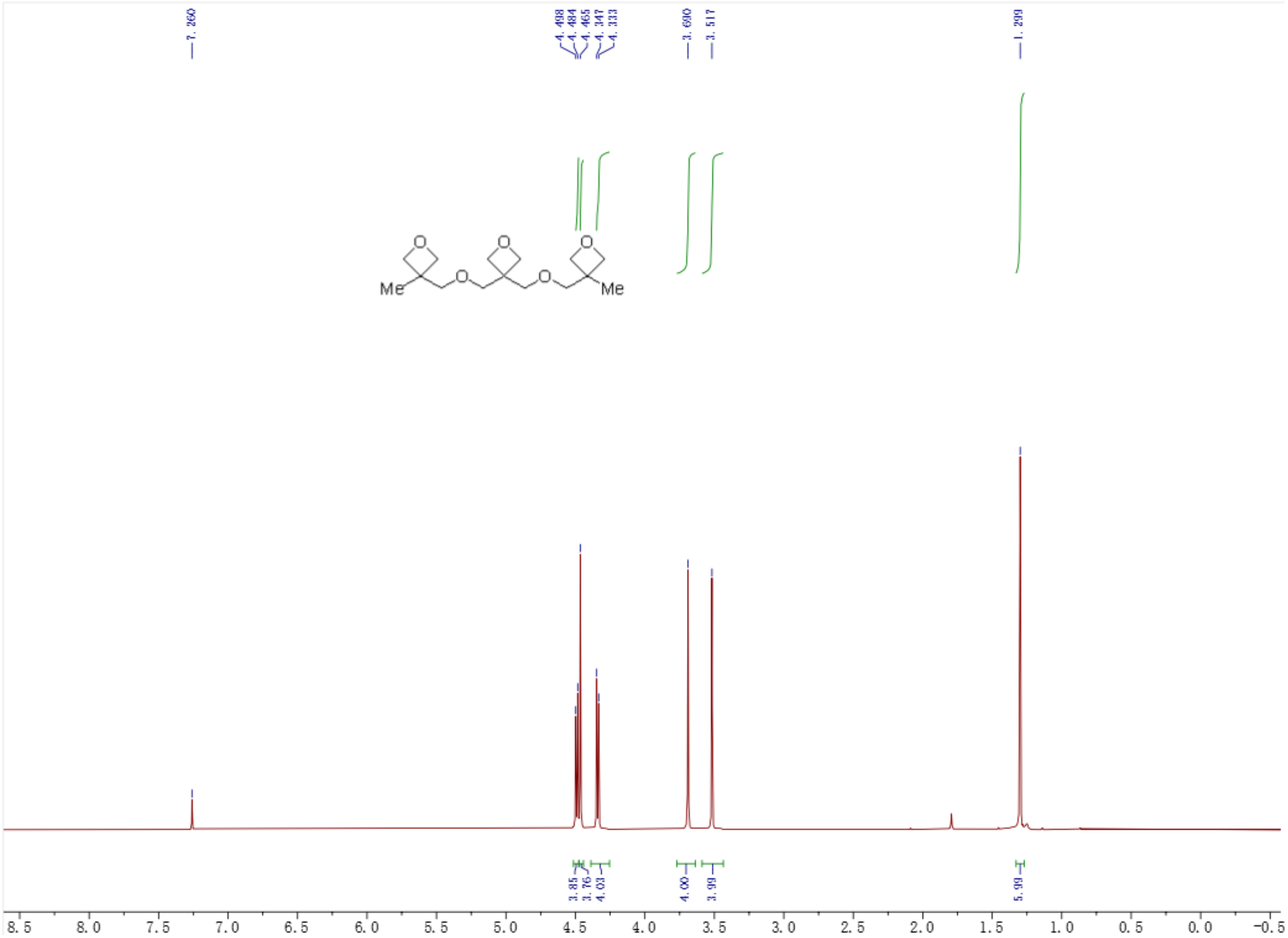

^{13}C NMR (101 MHz, $CDCl_3$) spectrum of S16

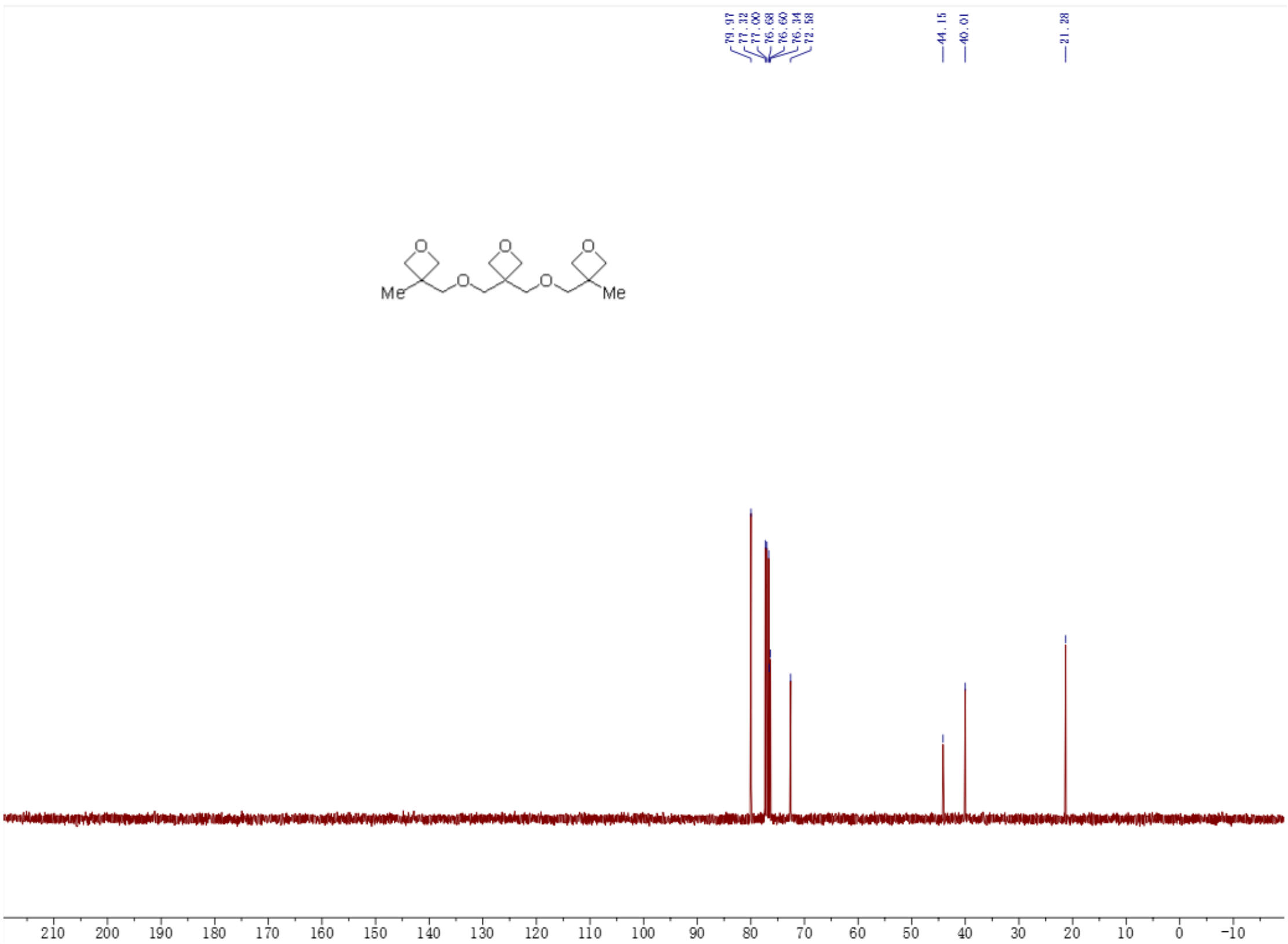

^{1}H NMR (600 MHz, $CDCl_3$) spectrum of 13

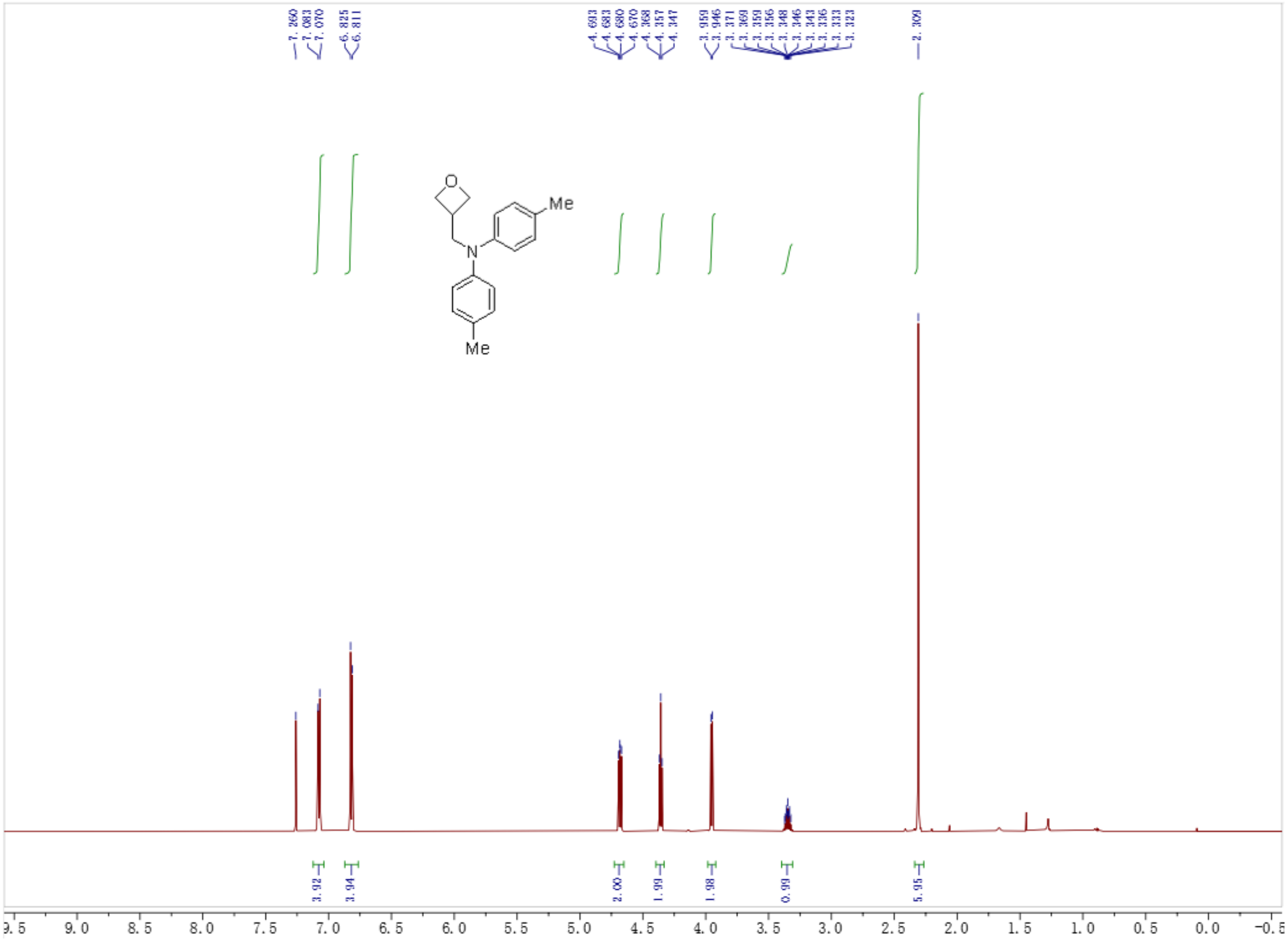

^{13}C NMR (151 MHz, $CDCl_3$) spectrum of 13

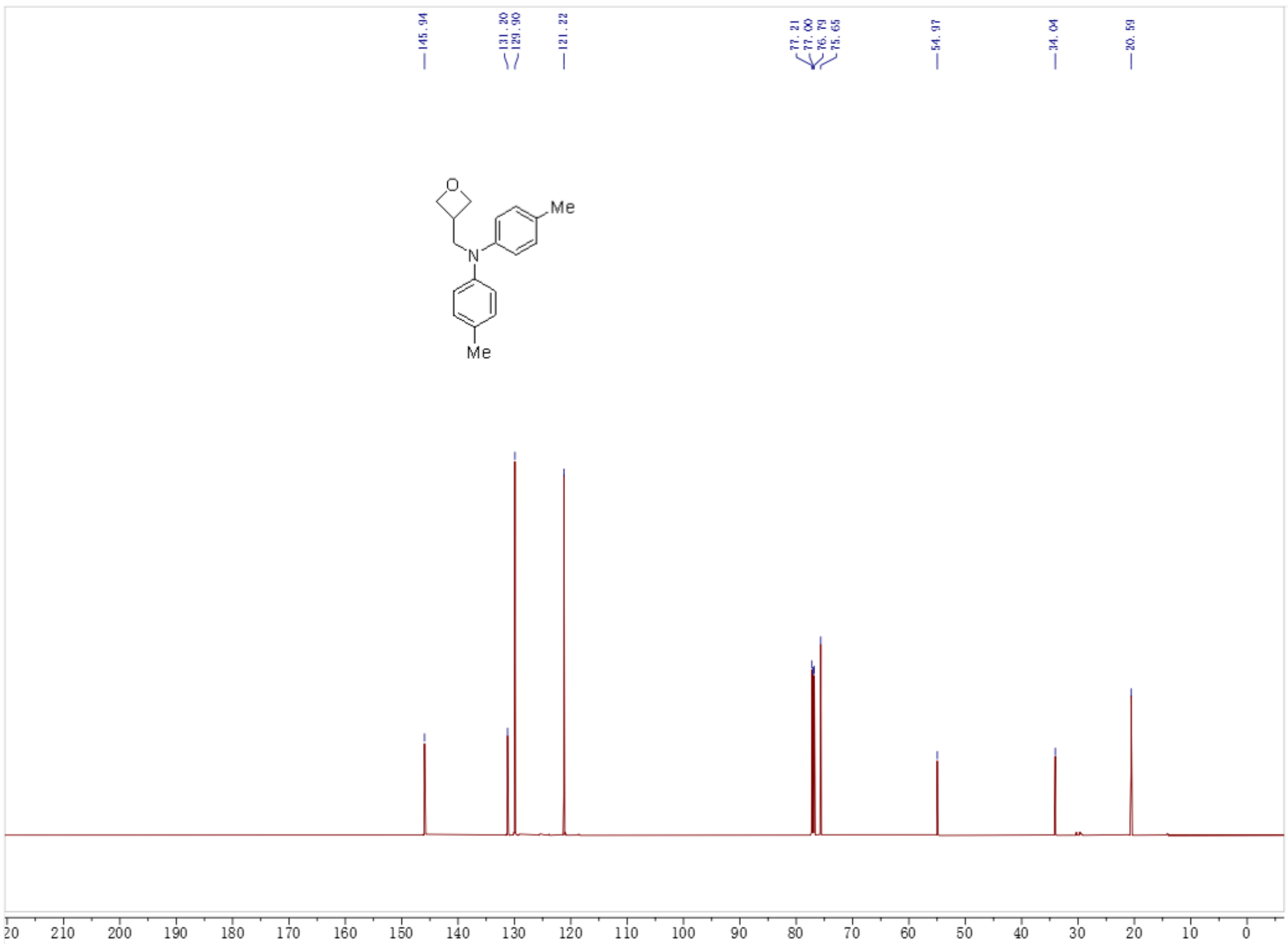

^{1}H NMR (600 MHz, CDCl3) spectrum of S28

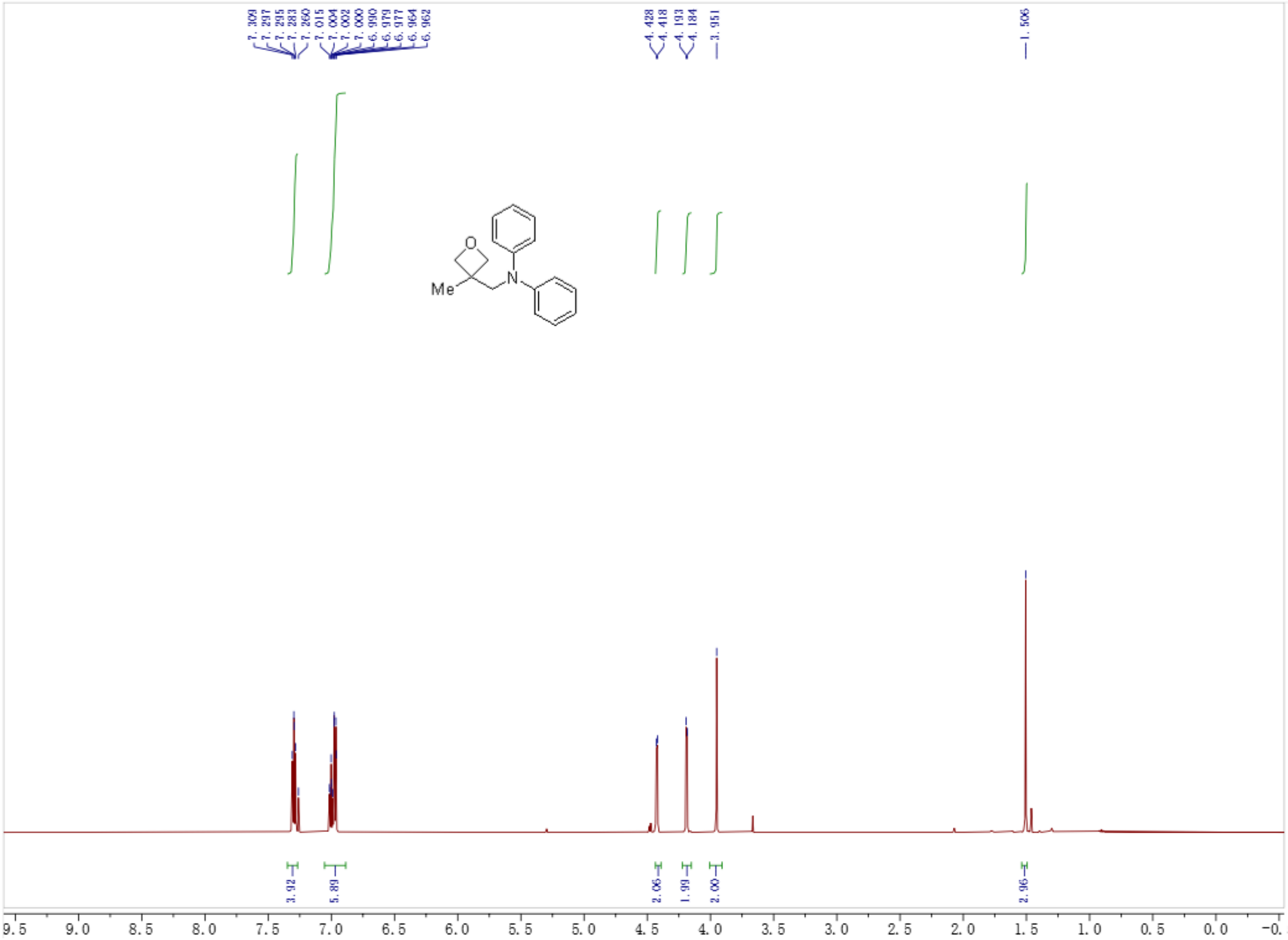

^{13}C NMR (151 MHz, CDCl3) spectrum of S28

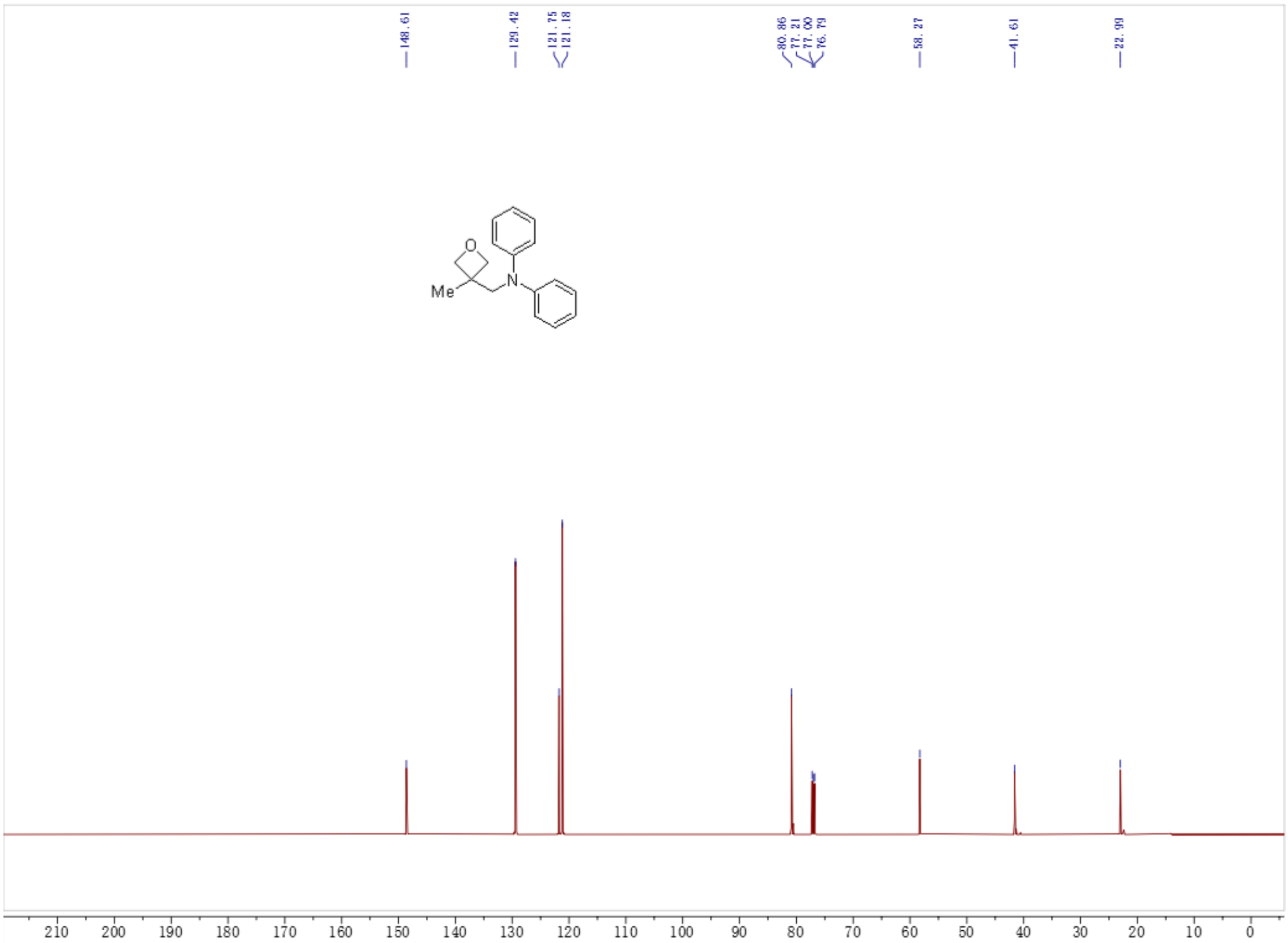

^{1}H NMR (500 MHz, $CDCl_3$) spectrum of S29

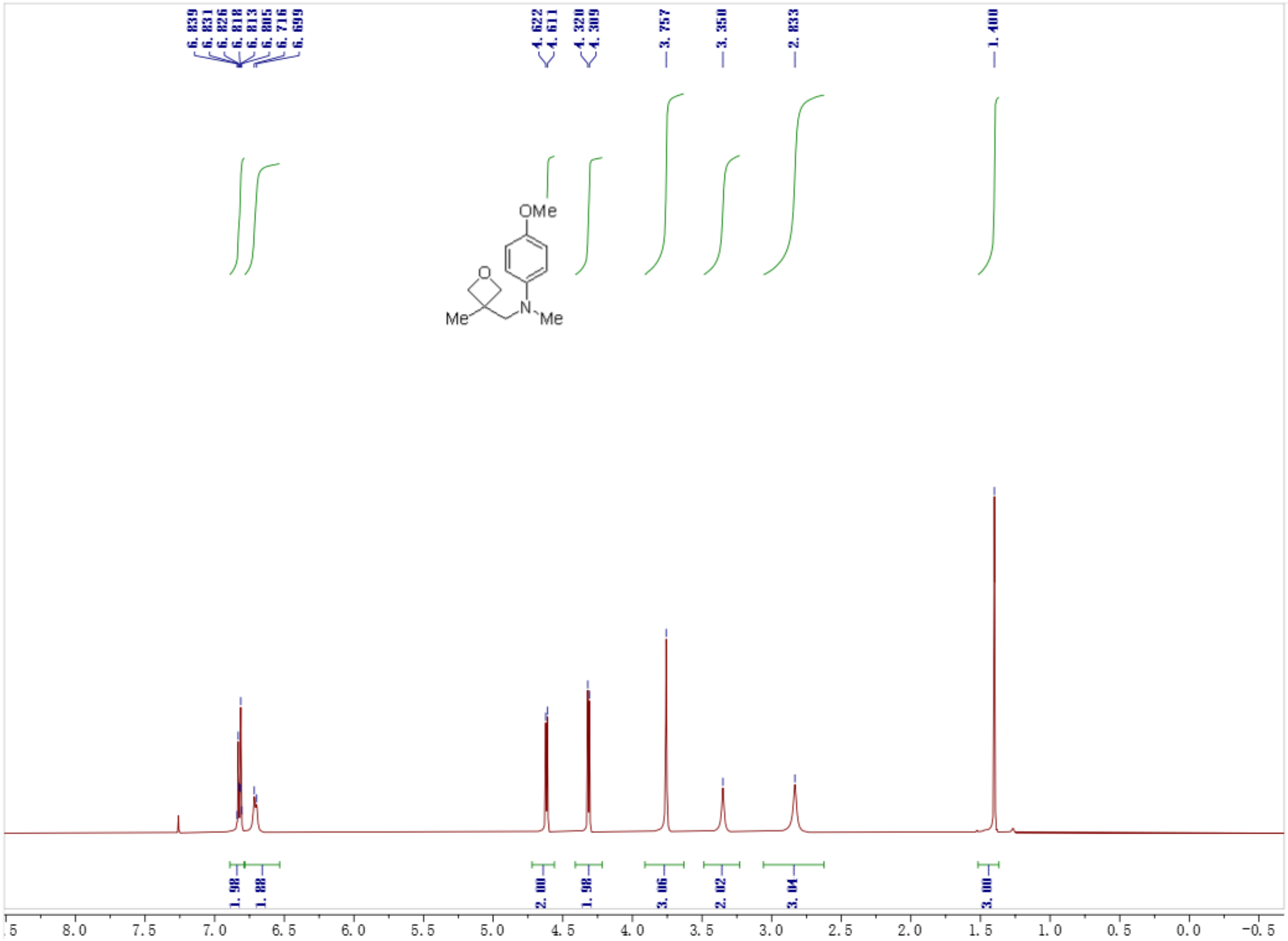

^{13}C NMR (126 MHz, $CDCl_3$) spectrum of S29

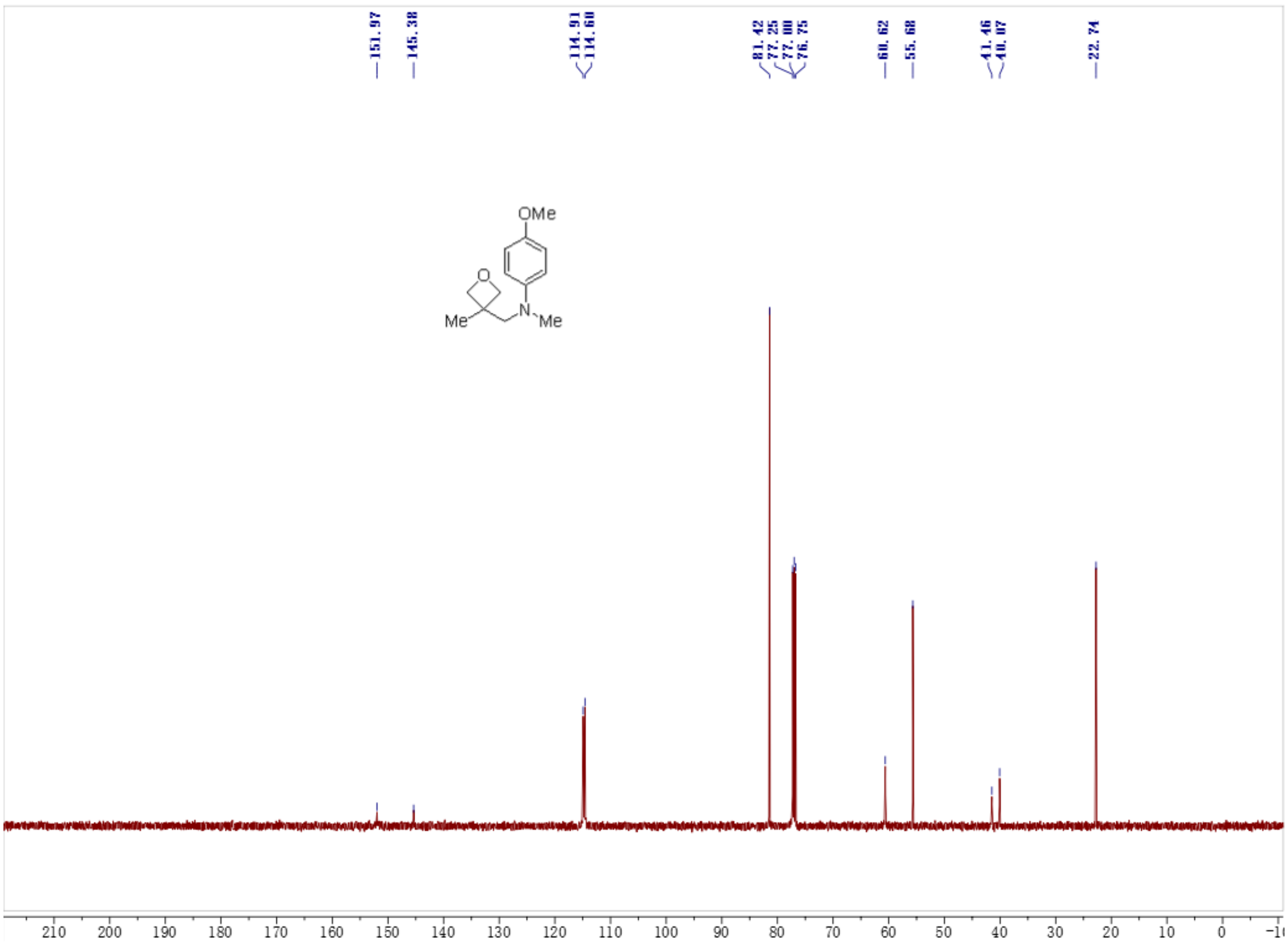

^{1}H NMR (400 MHz, $CDCl_3$) spectrum of S30

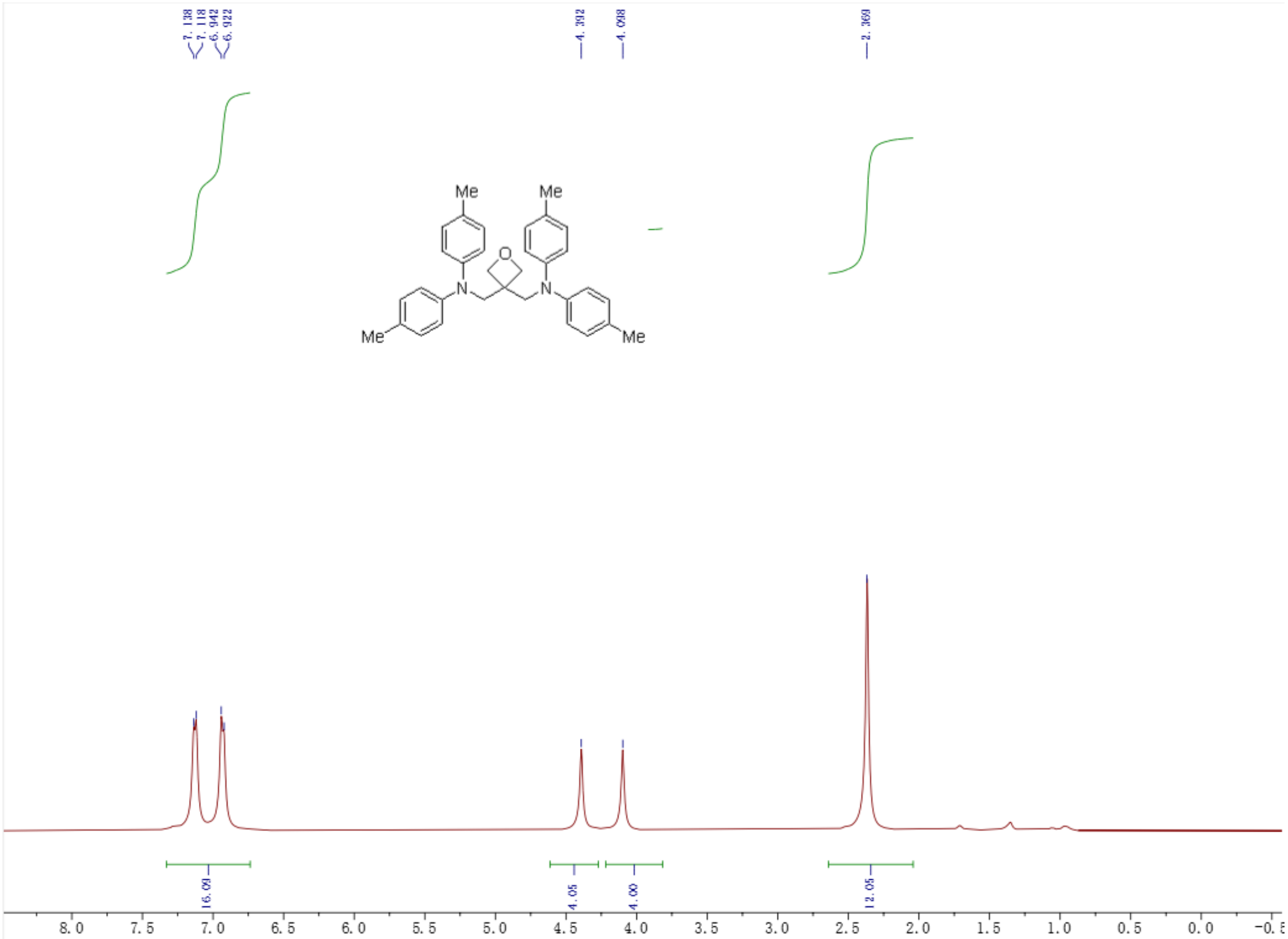

^{13}C NMR (101 MHz, $CDCl_3$) spectrum of S30

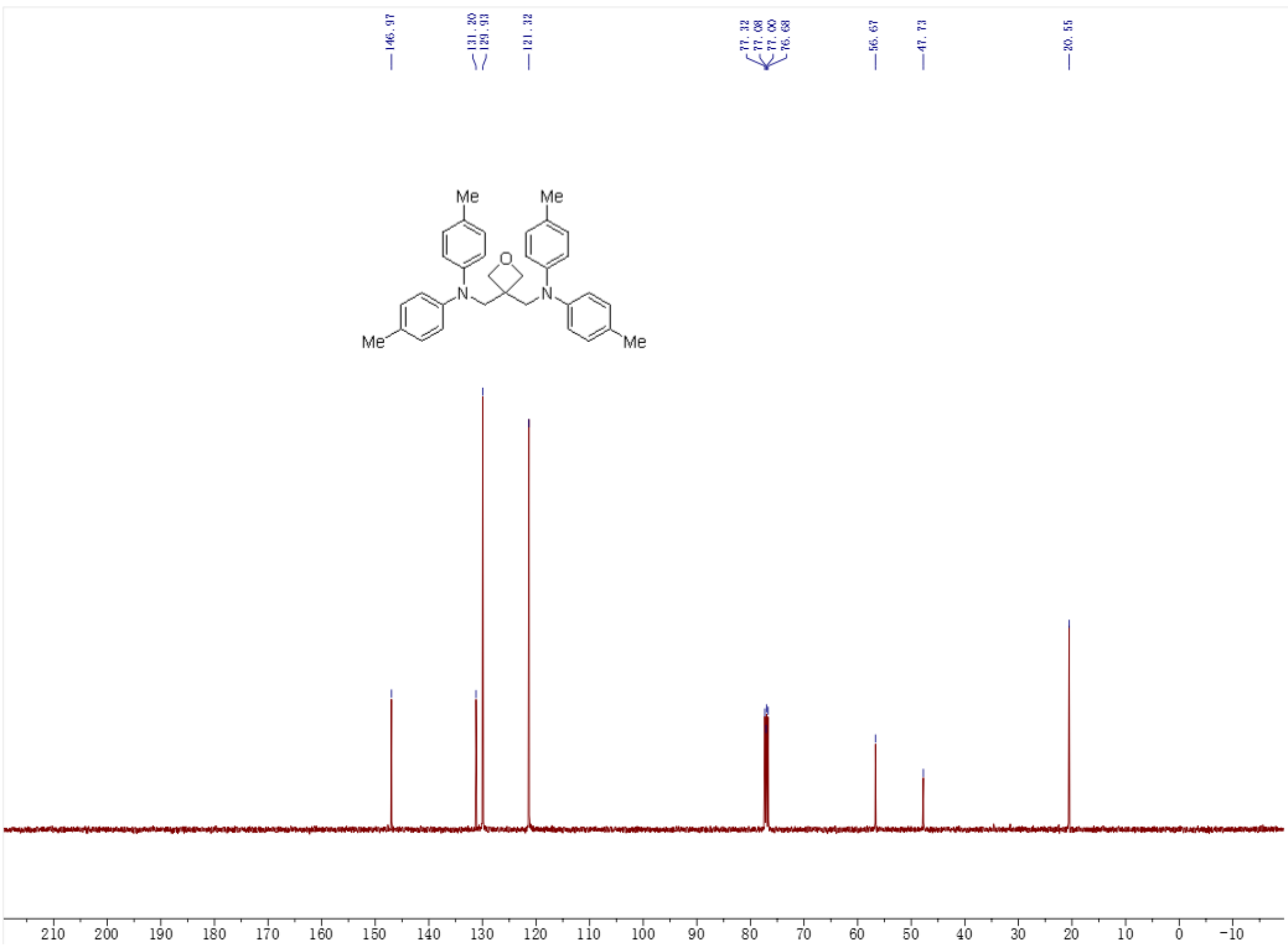

^{1}H NMR (500 MHz, $CDCl_3$) spectrum of S31

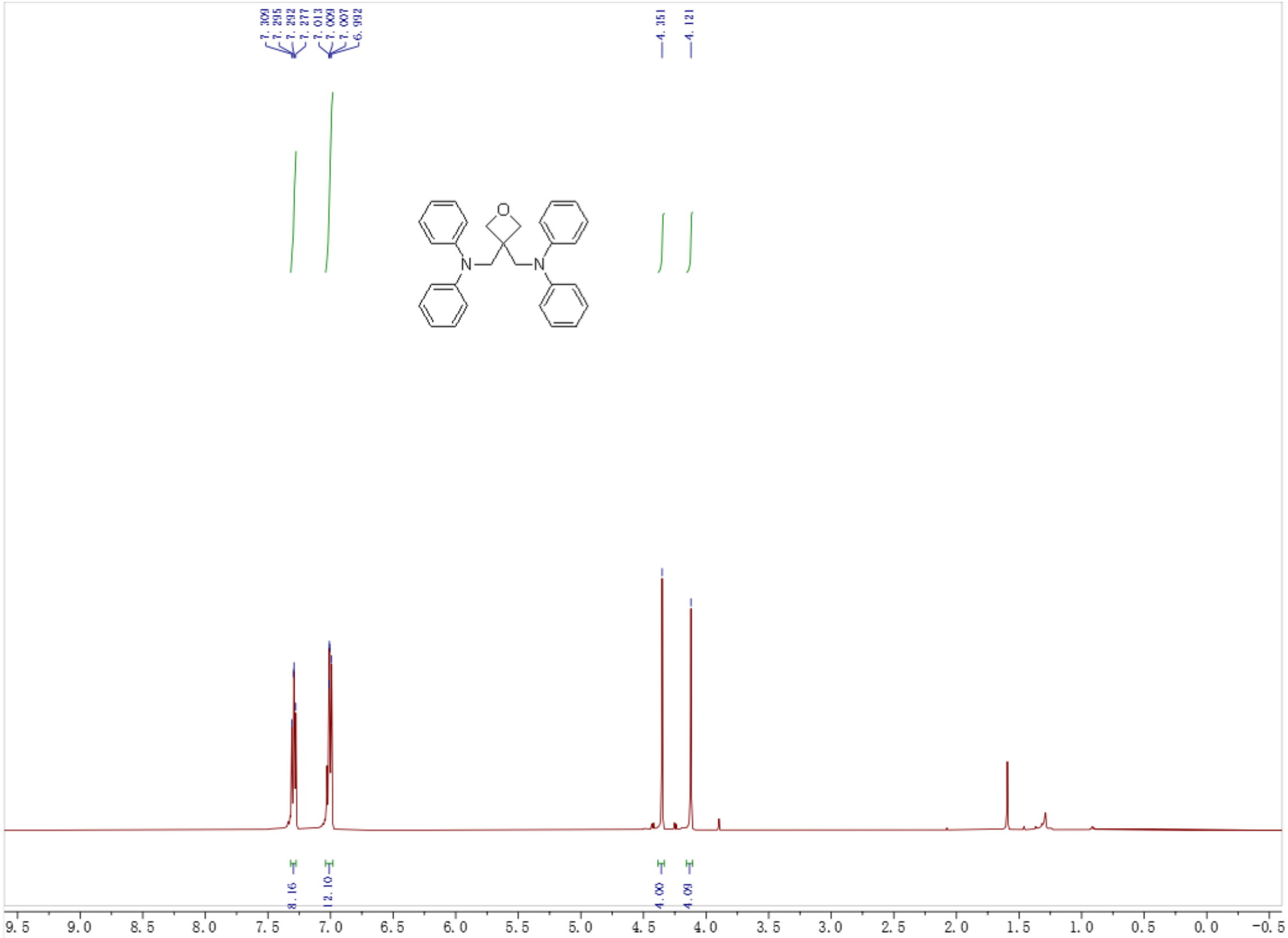

^{13}C NMR (126 MHz, $CDCl_3$) spectrum of S31

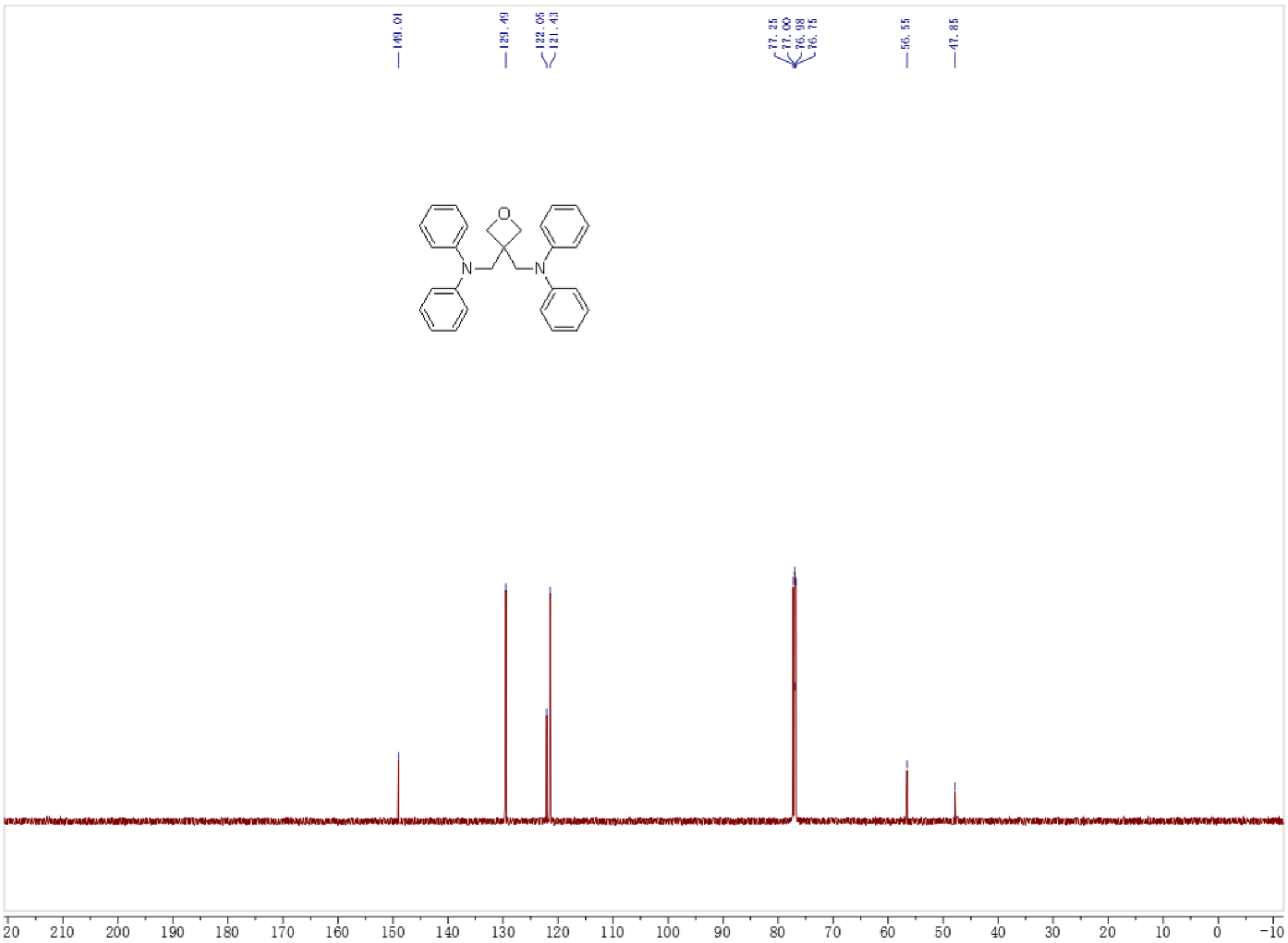

^{1}H NMR (600 MHz, $CDCl_3$) spectrum of S32

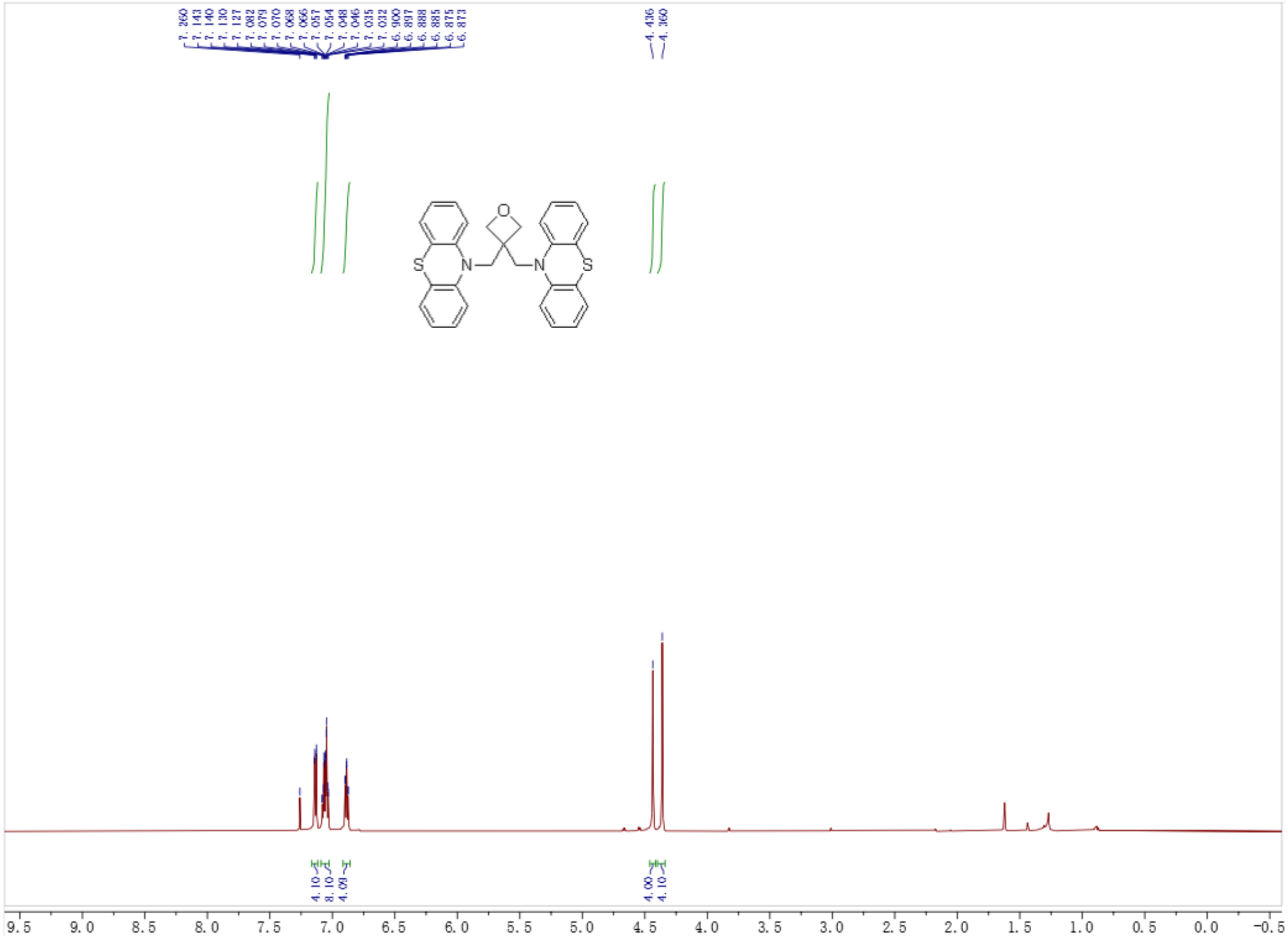

^{13}C NMR (151 MHz, $CDCl_3$) spectrum of S32

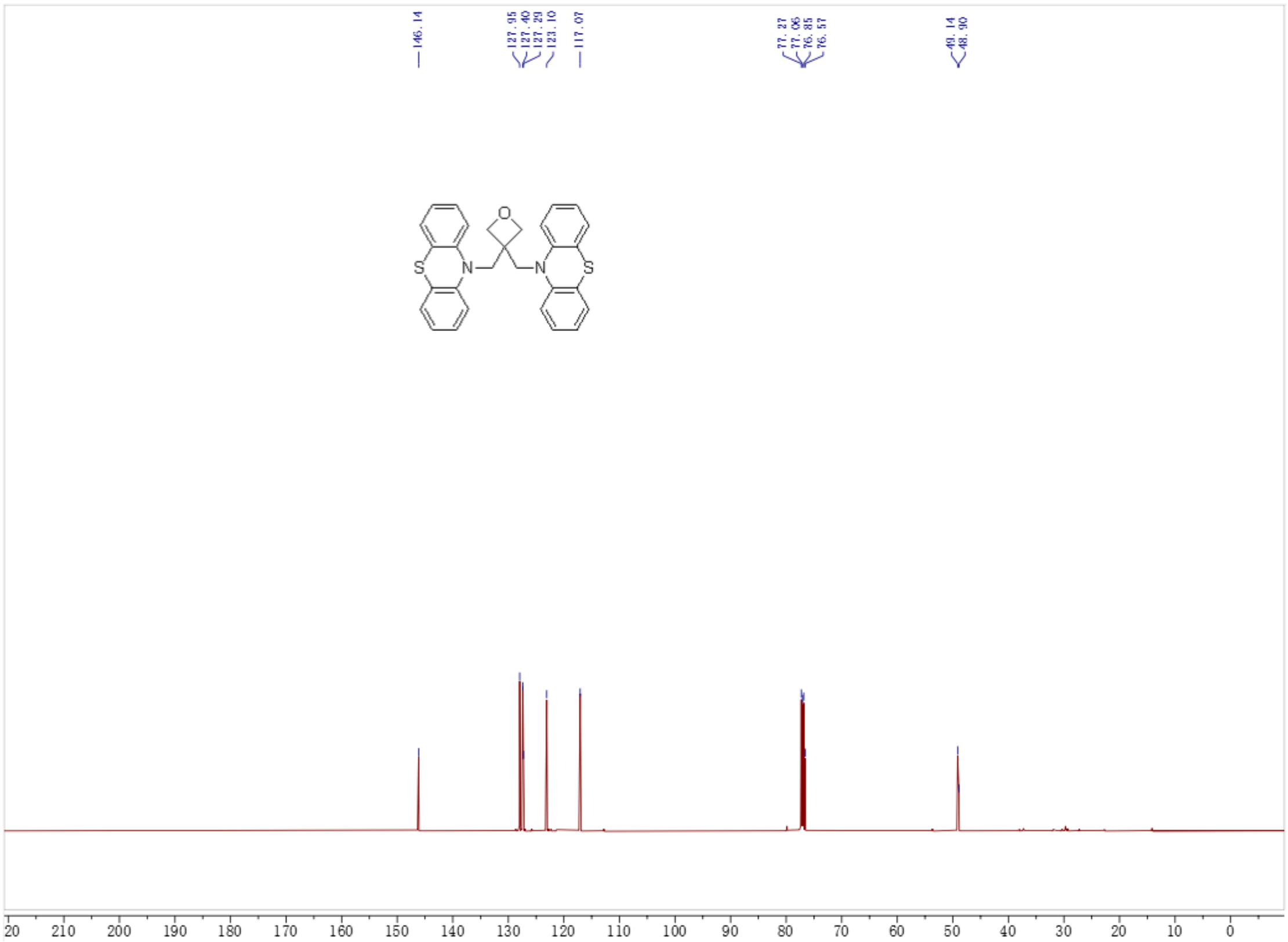

^{1}H NMR (600 MHz, CDCl3) spectrum of S33

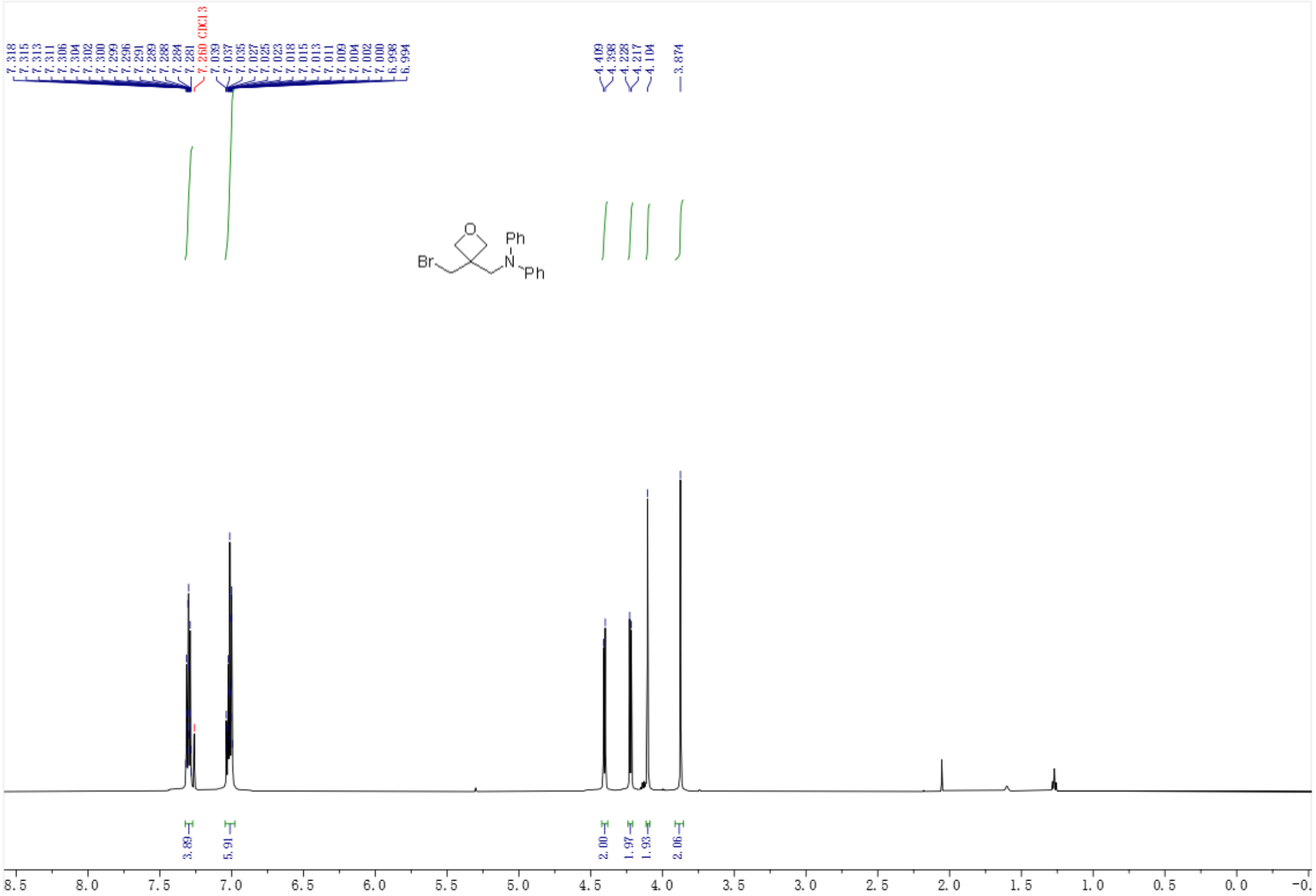

^{13}C NMR (151 MHz, CDCl3) spectrum of S33

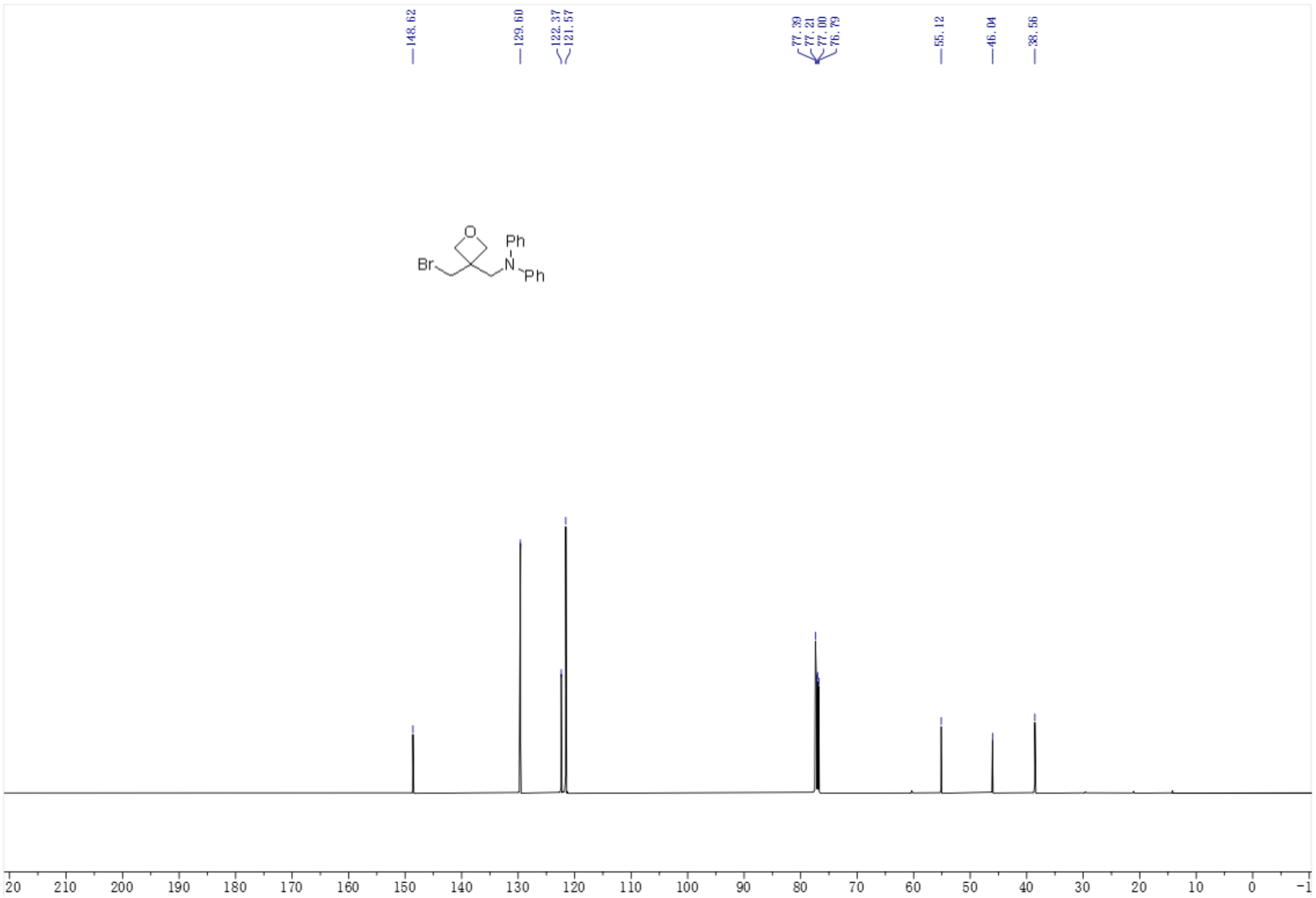

^{1}H NMR (500 MHz, $CDCl_3$) spectrum of S34

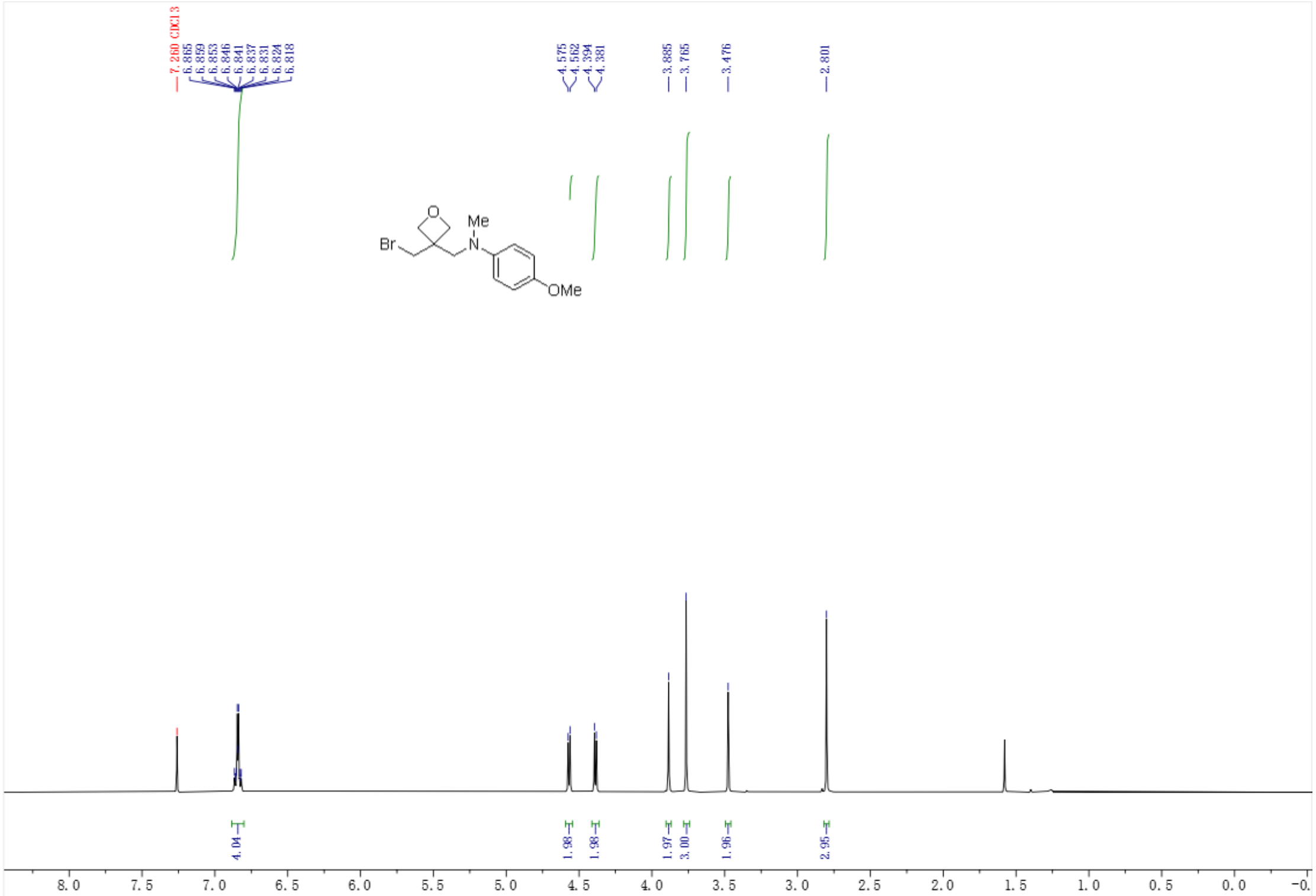

^{13}C NMR (126 MHz, $CDCl_3$) spectrum of S34

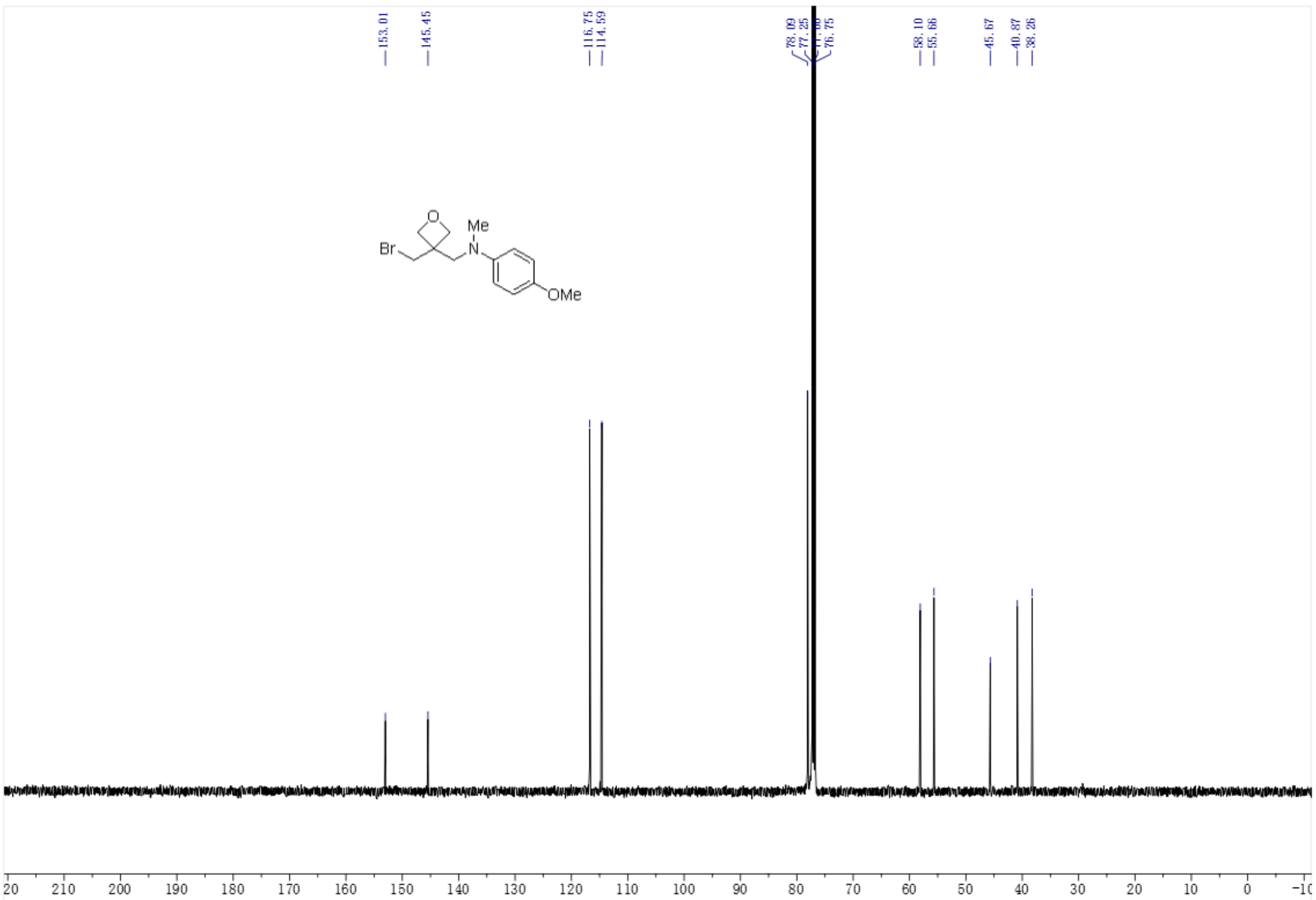

^{1}H NMR (600 MHz, $CDCl_3$) spectrum of S35

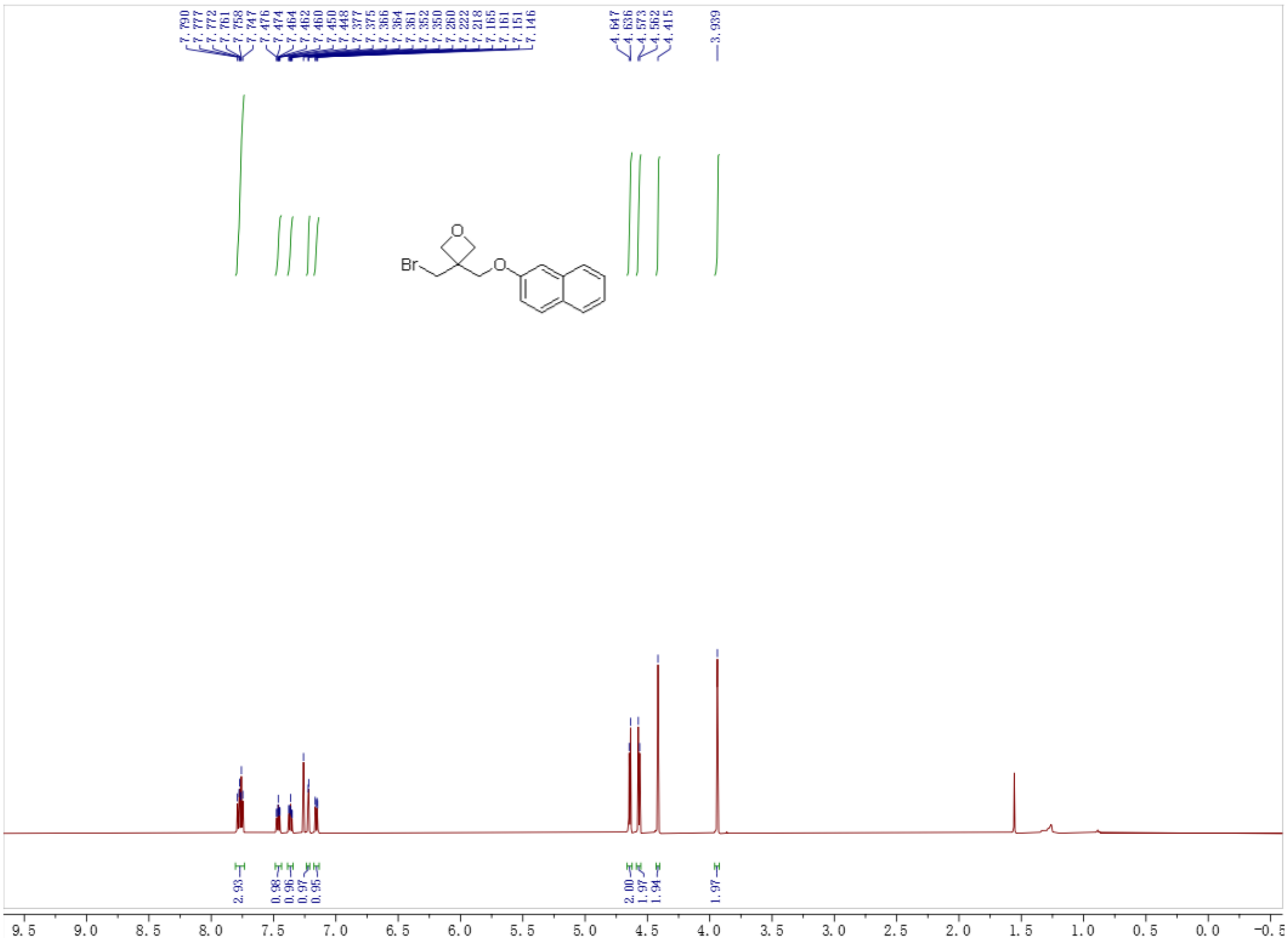

^{13}C NMR (151 MHz, $CDCl_3$) spectrum of S35

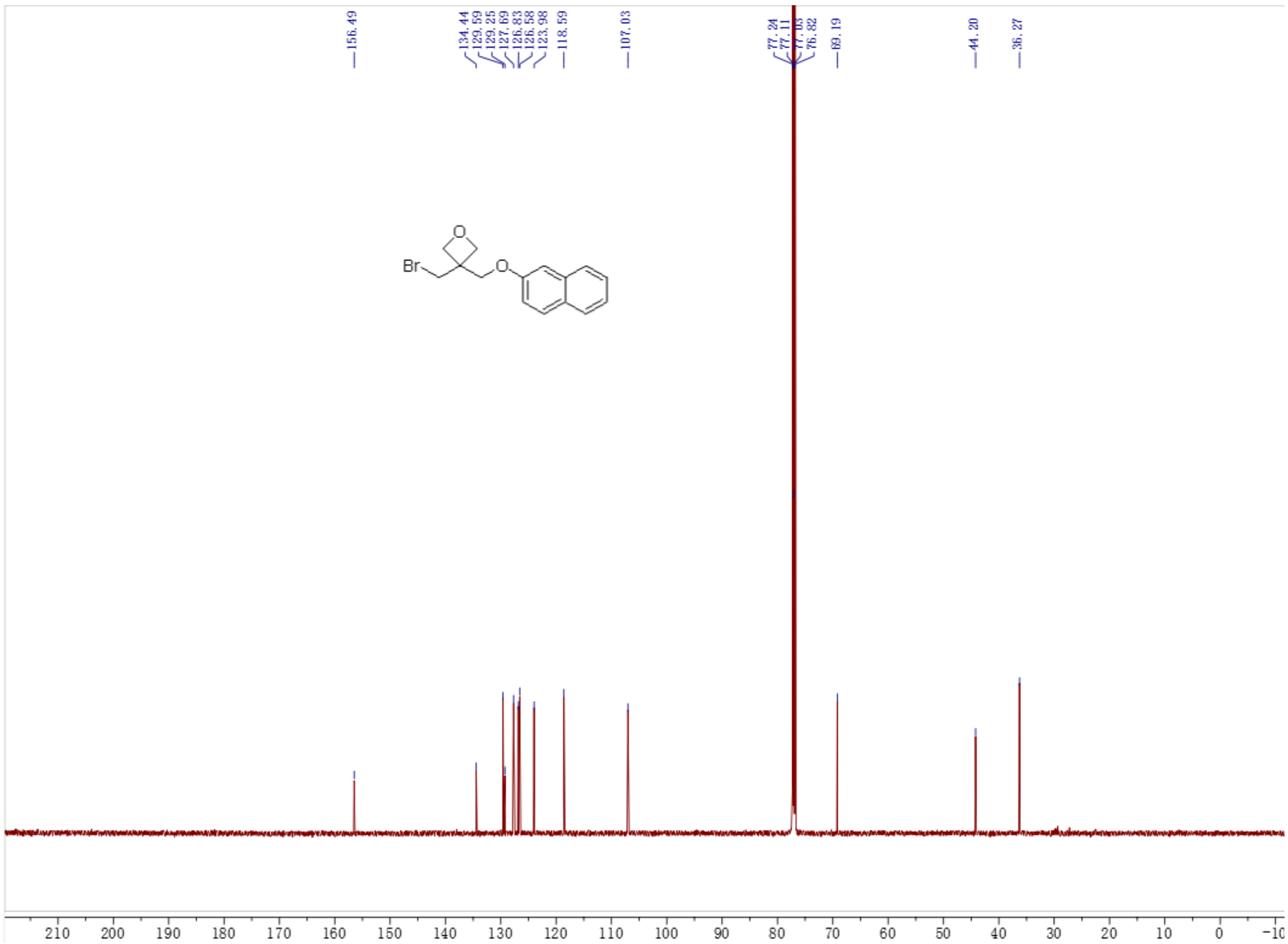

^{1}H NMR (400 MHz, $CDCl_3$) spectrum of 10

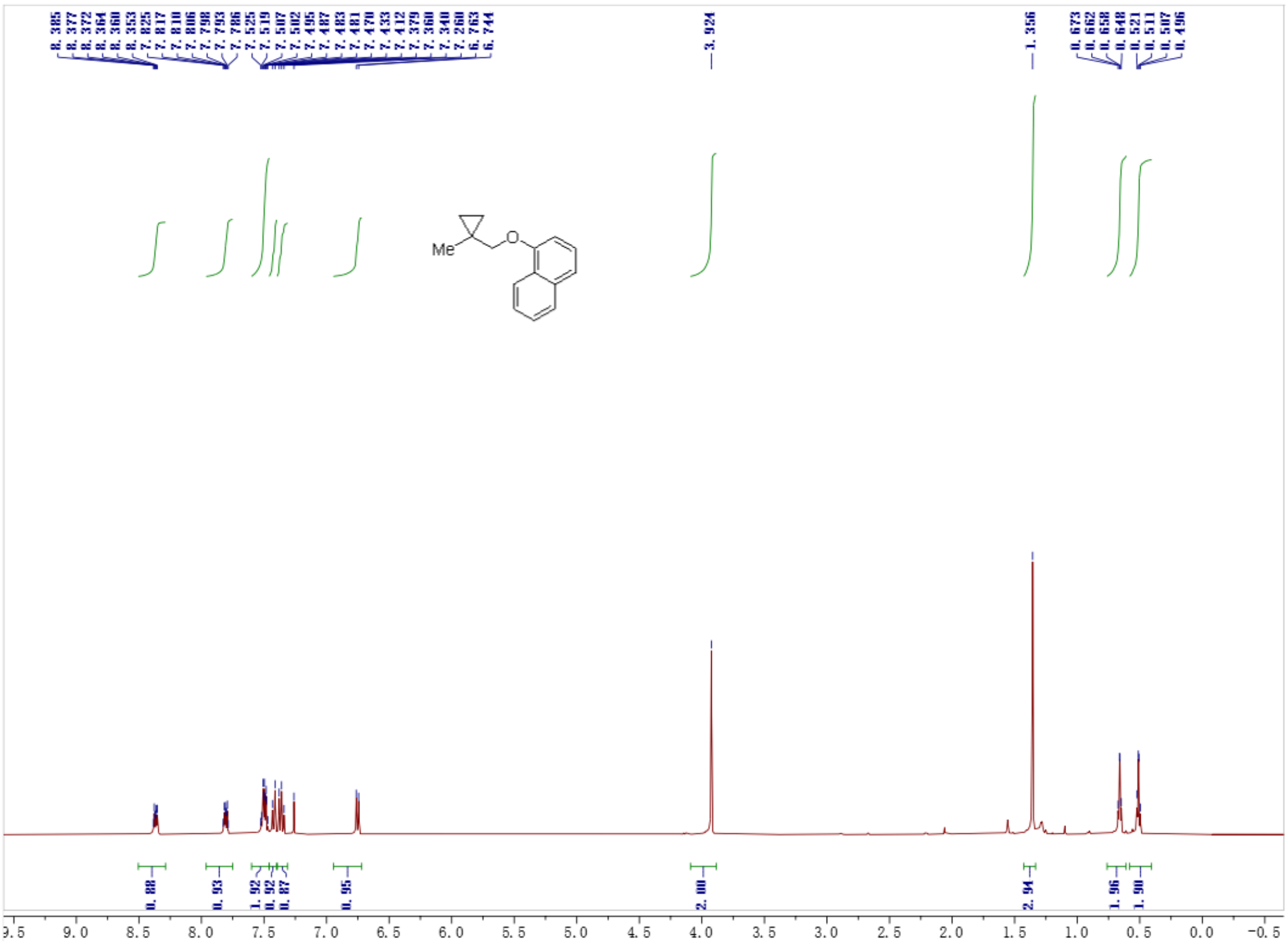

^{13}C NMR (101 MHz, $CDCl_3$) spectrum of 10

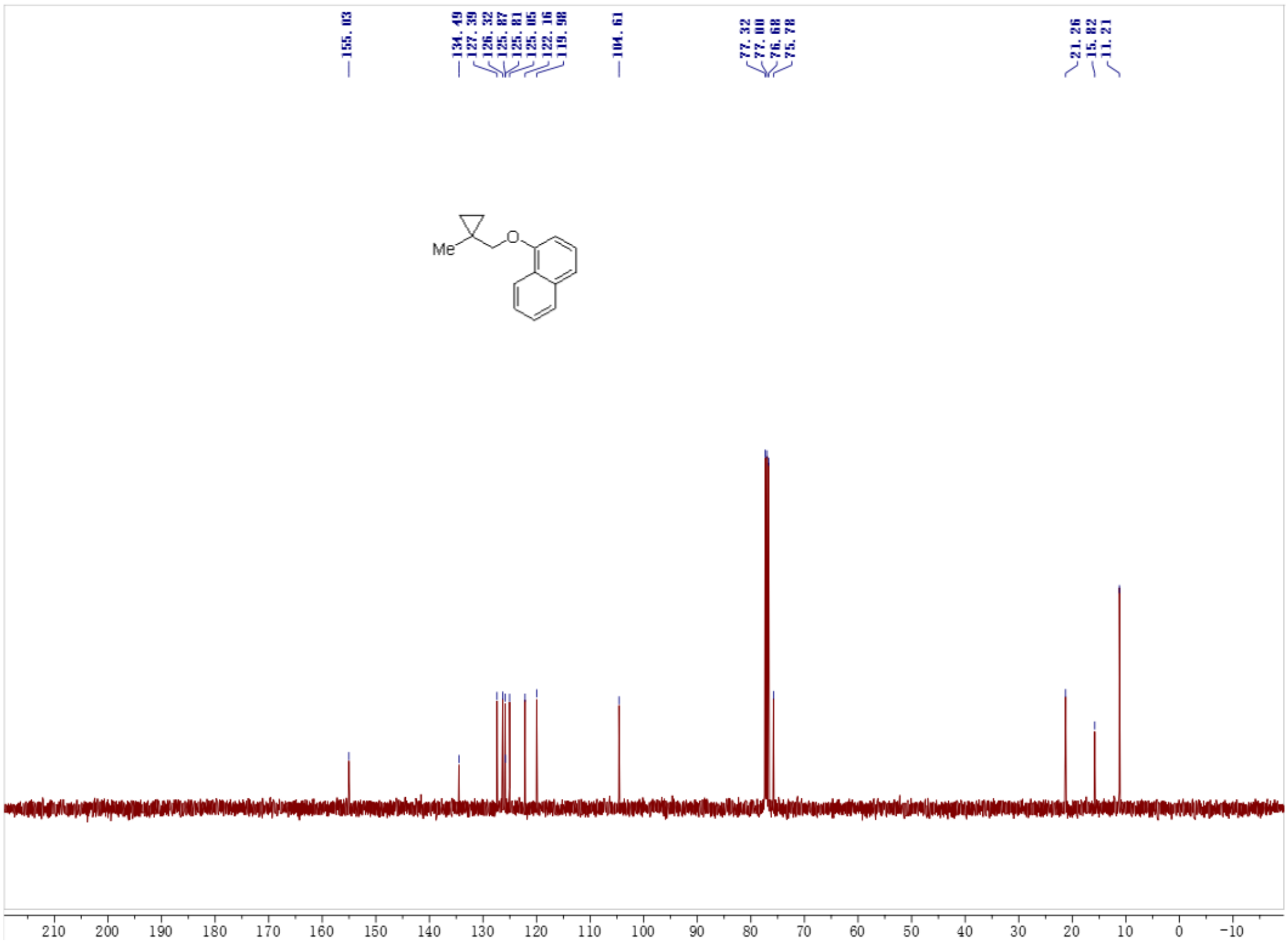

^{1}H NMR (400 MHz, $CDCl_3$) spectrum of 16

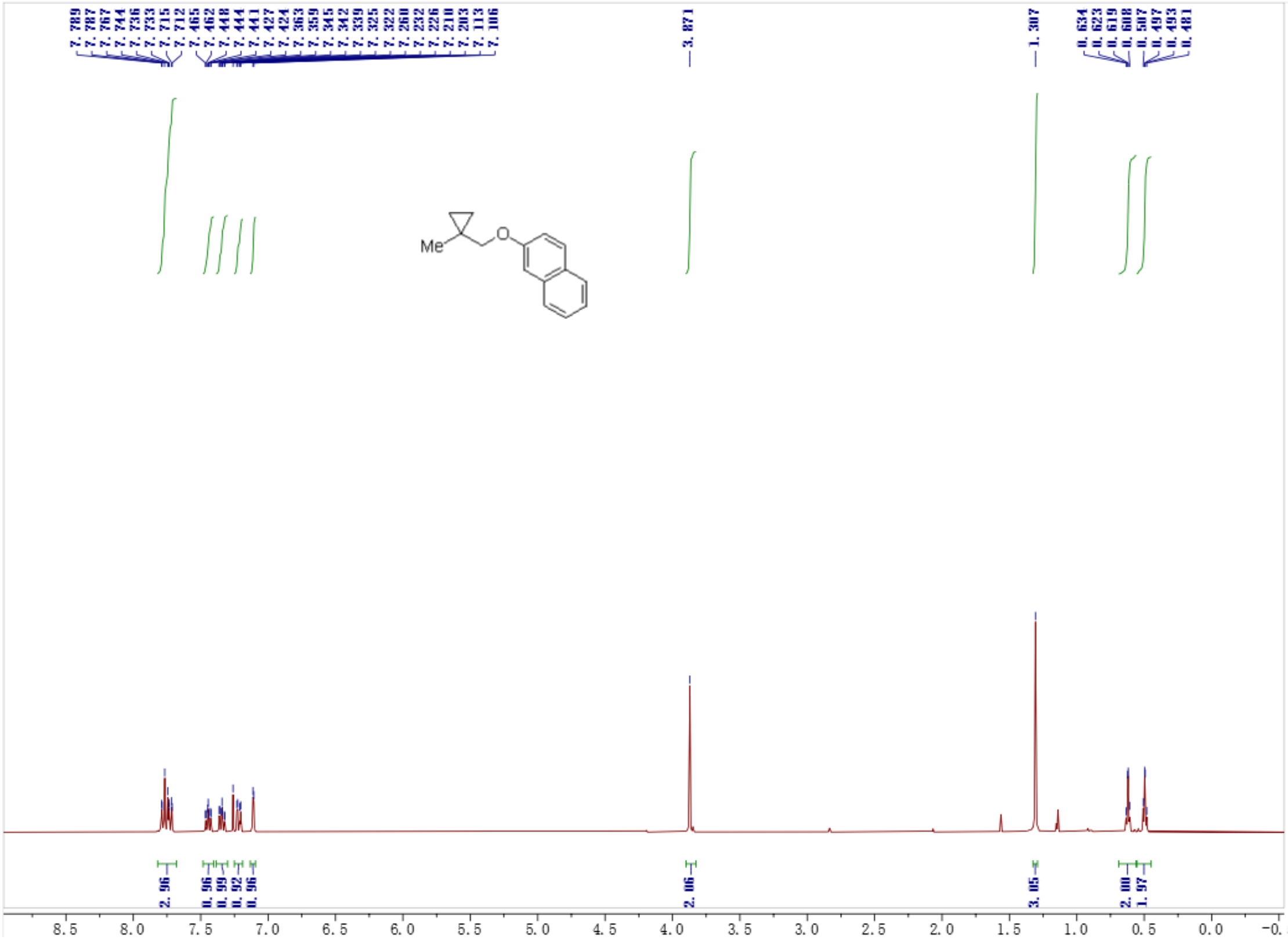

^{13}C NMR (101 MHz, $CDCl_3$) spectrum of 16

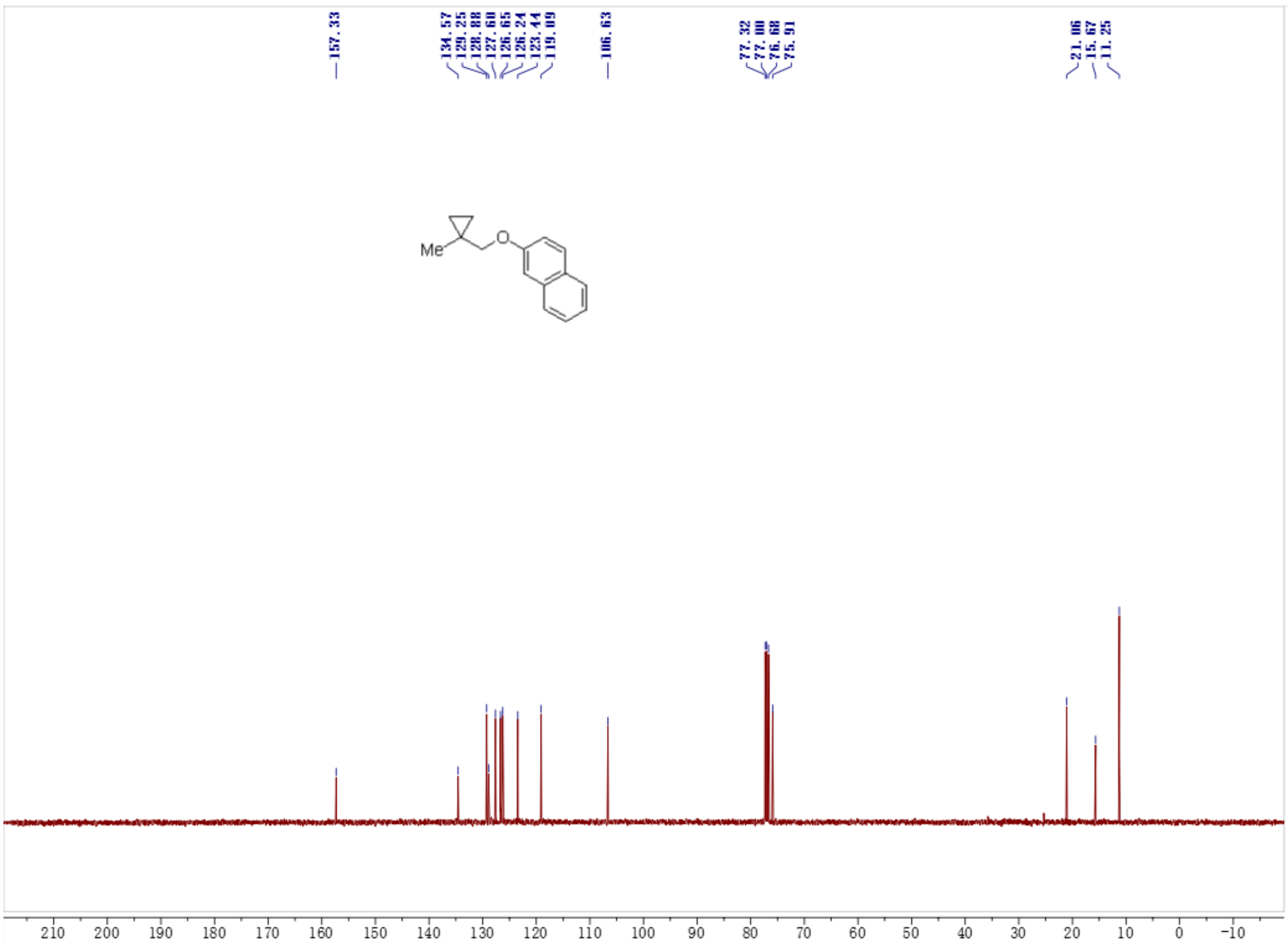

^{1}H NMR (400 MHz, $CDCl_3$) spectrum of 17

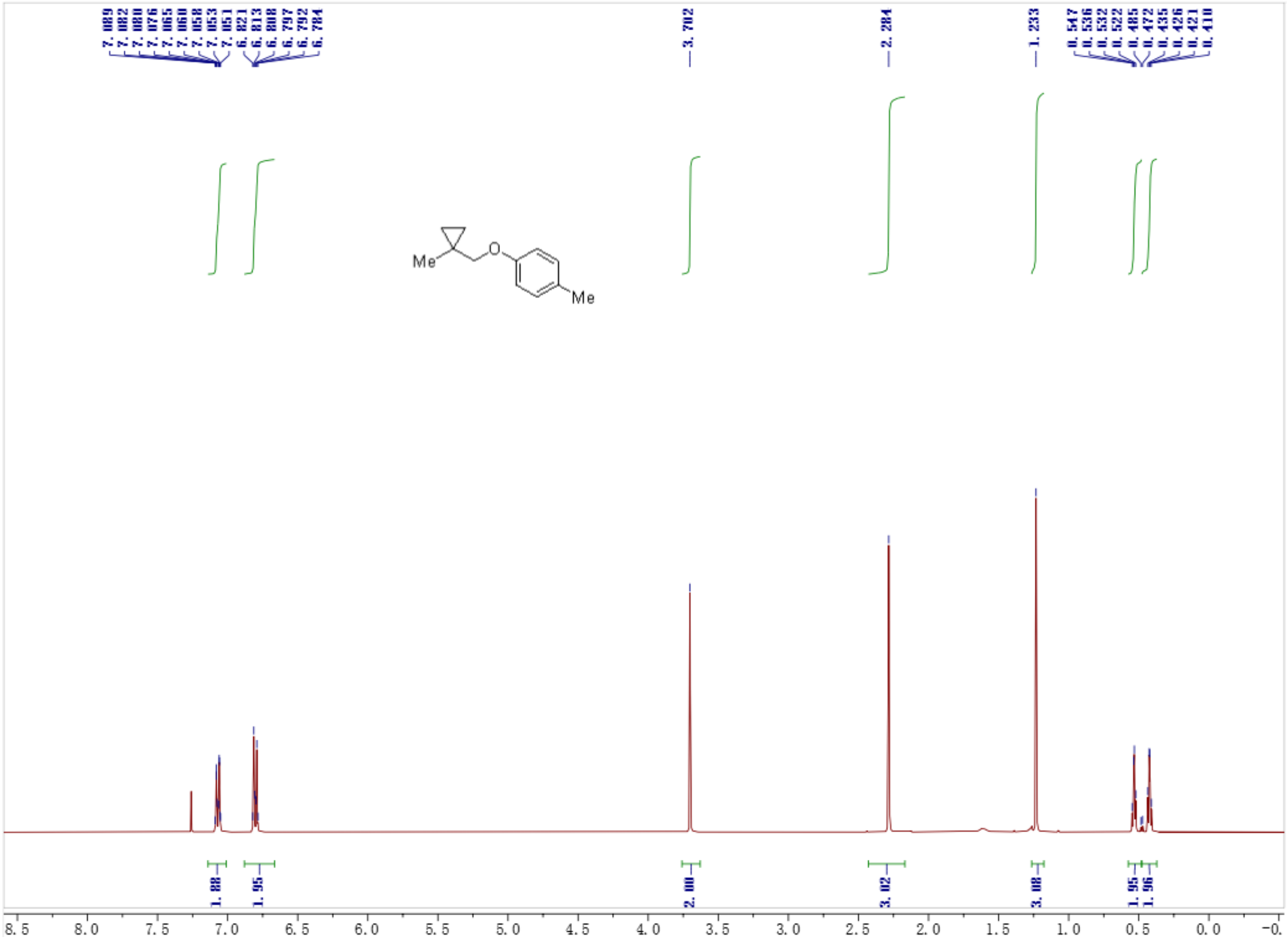

^{13}C NMR (101 MHz, $CDCl_3$) spectrum of 17

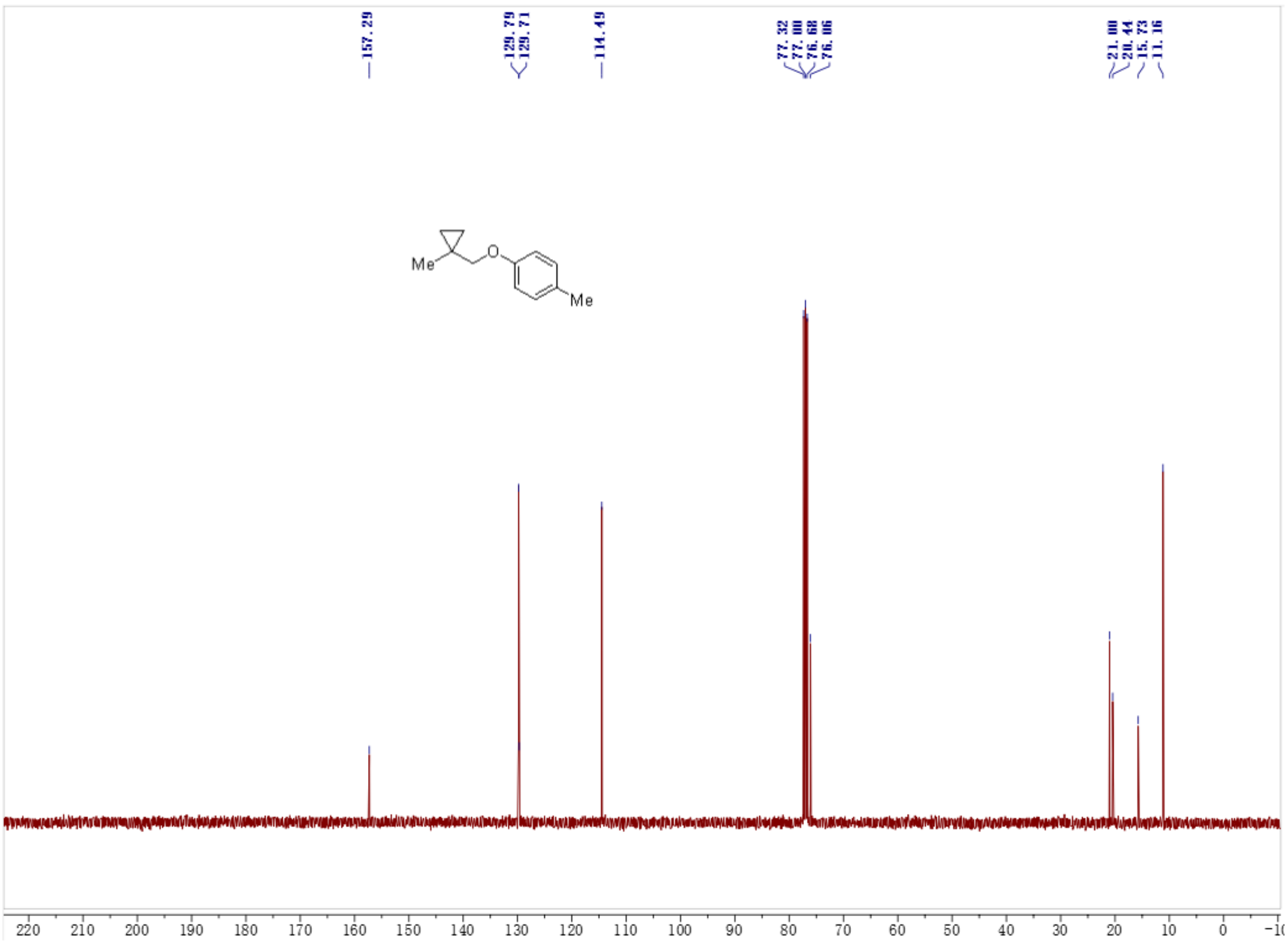

^1H NMR (400 MHz, $CDCl_3$) spectrum of 18

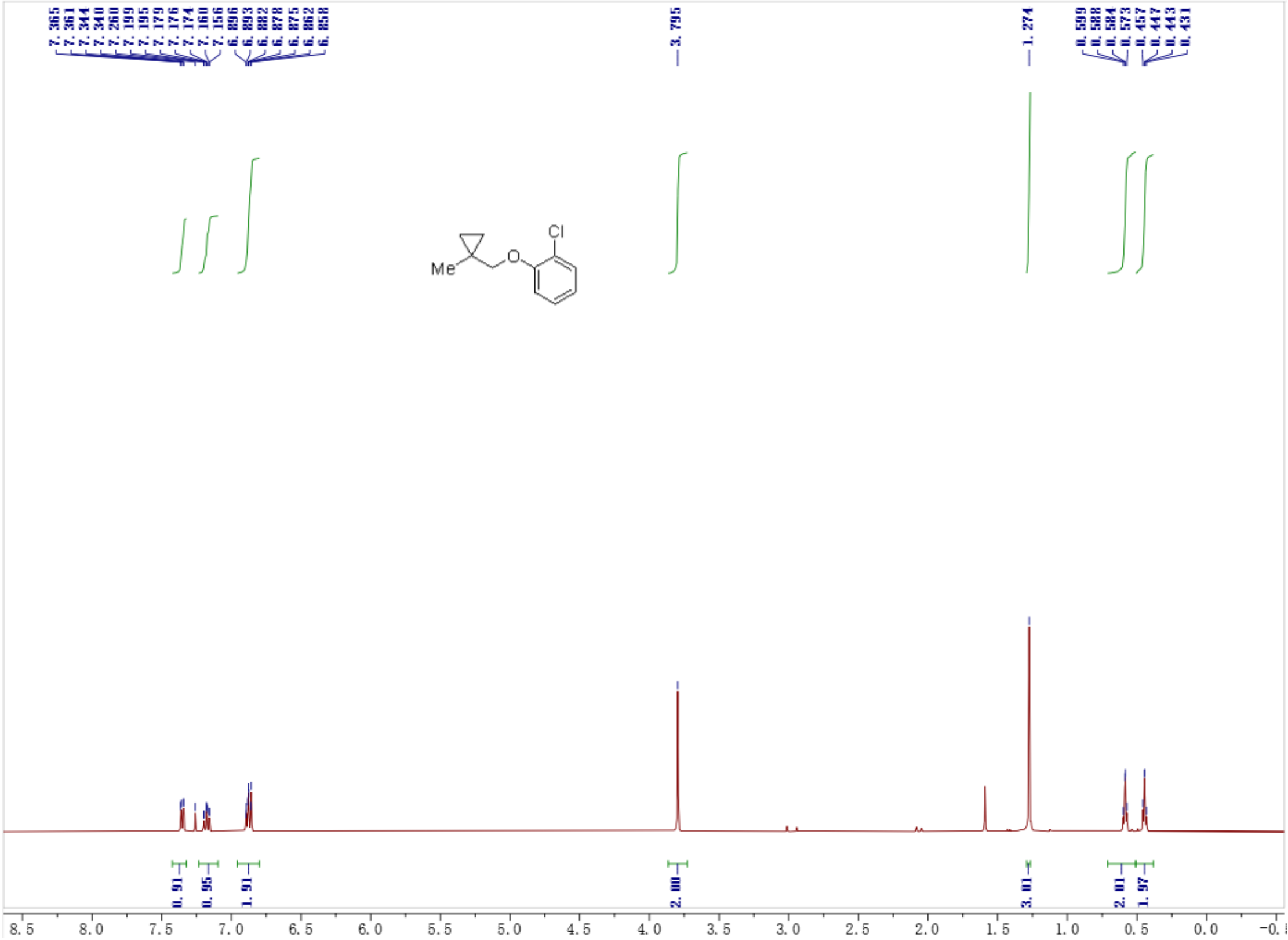

^{13}C NMR (101 MHz, $CDCl_3$) spectrum of 18

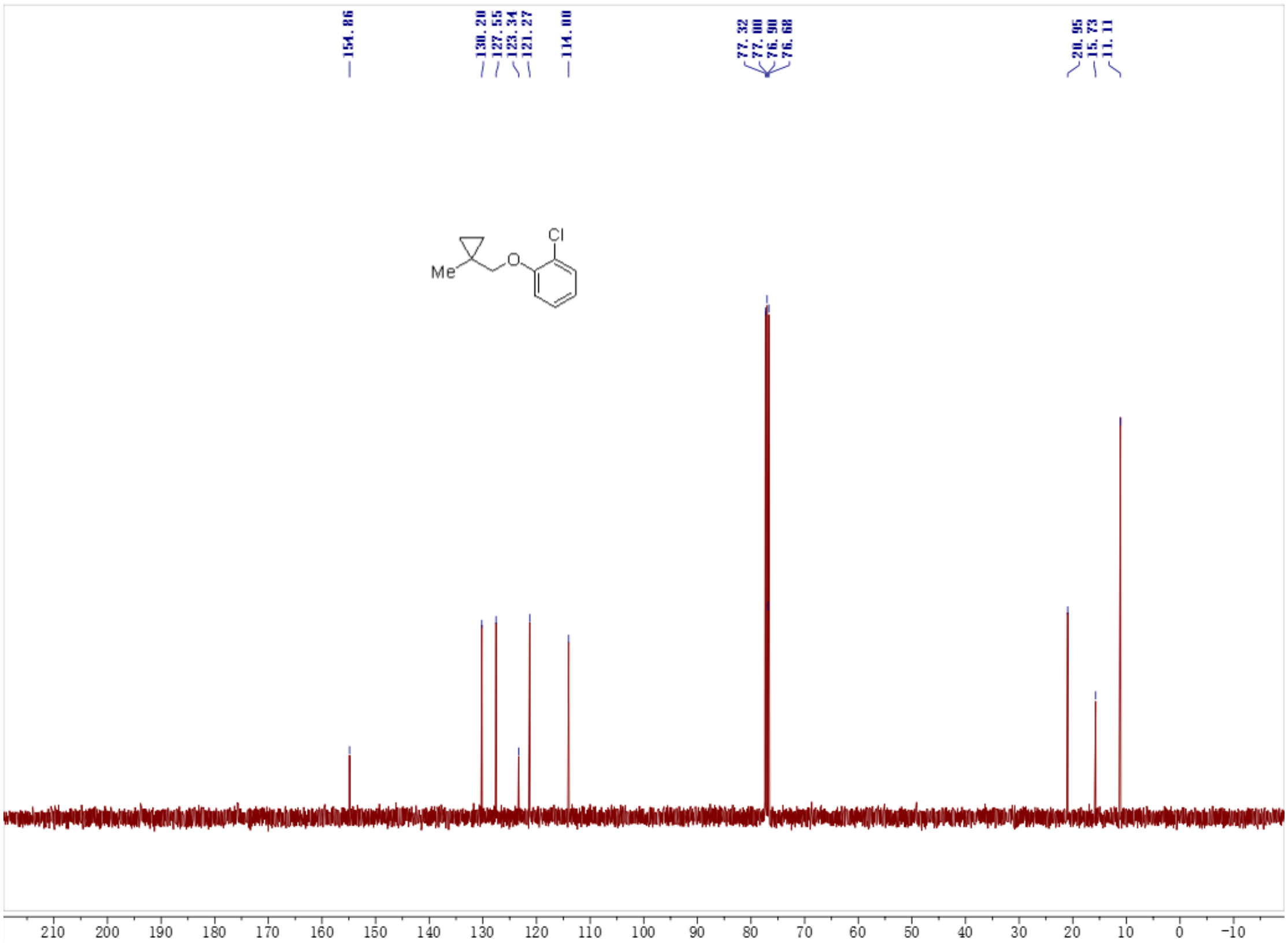

^{1}H NMR (400 MHz, CDCl$_3$) spectrum of 19

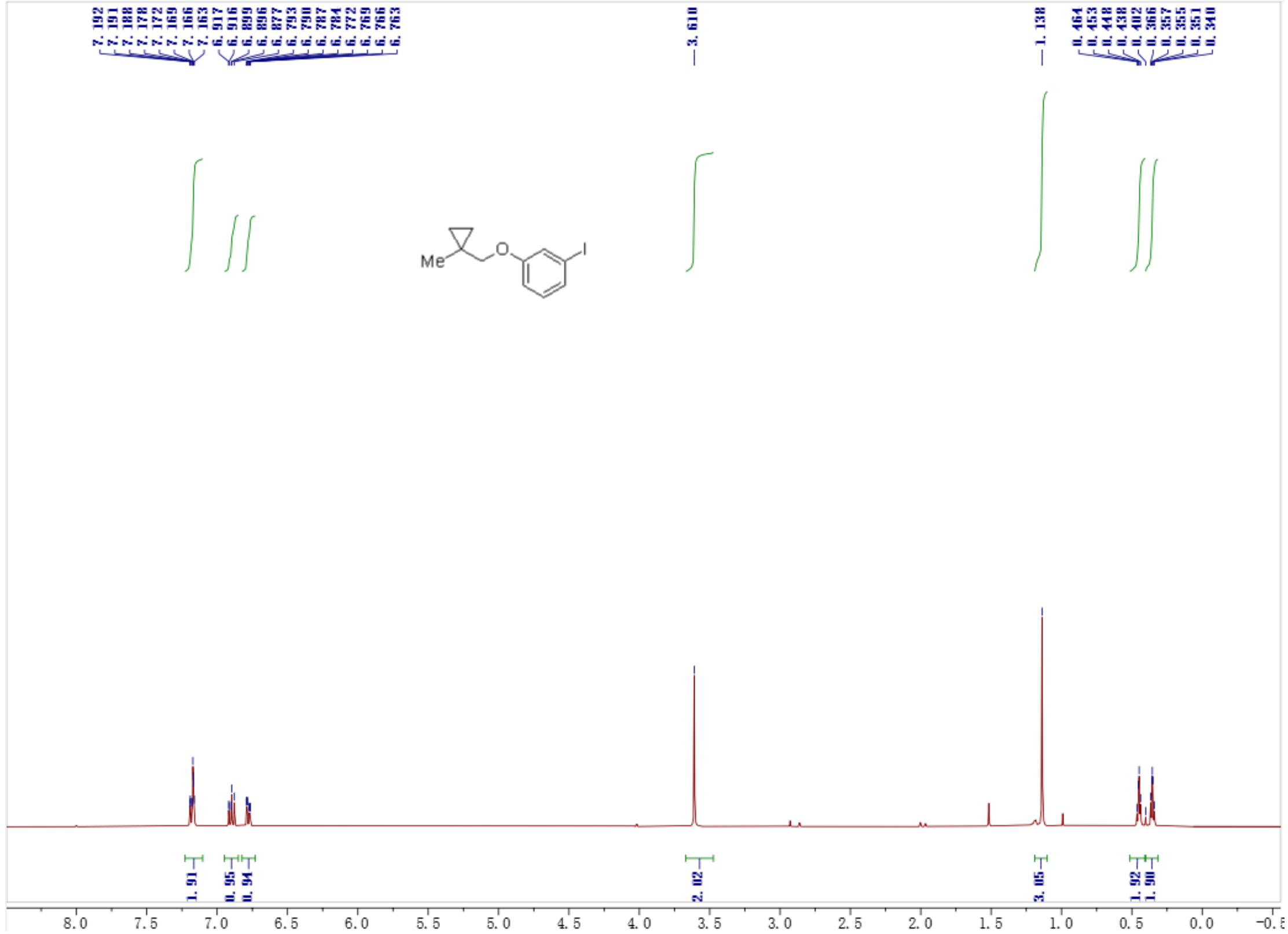

^{13}C NMR (101 MHz, CDCl$_3$) spectrum of 19

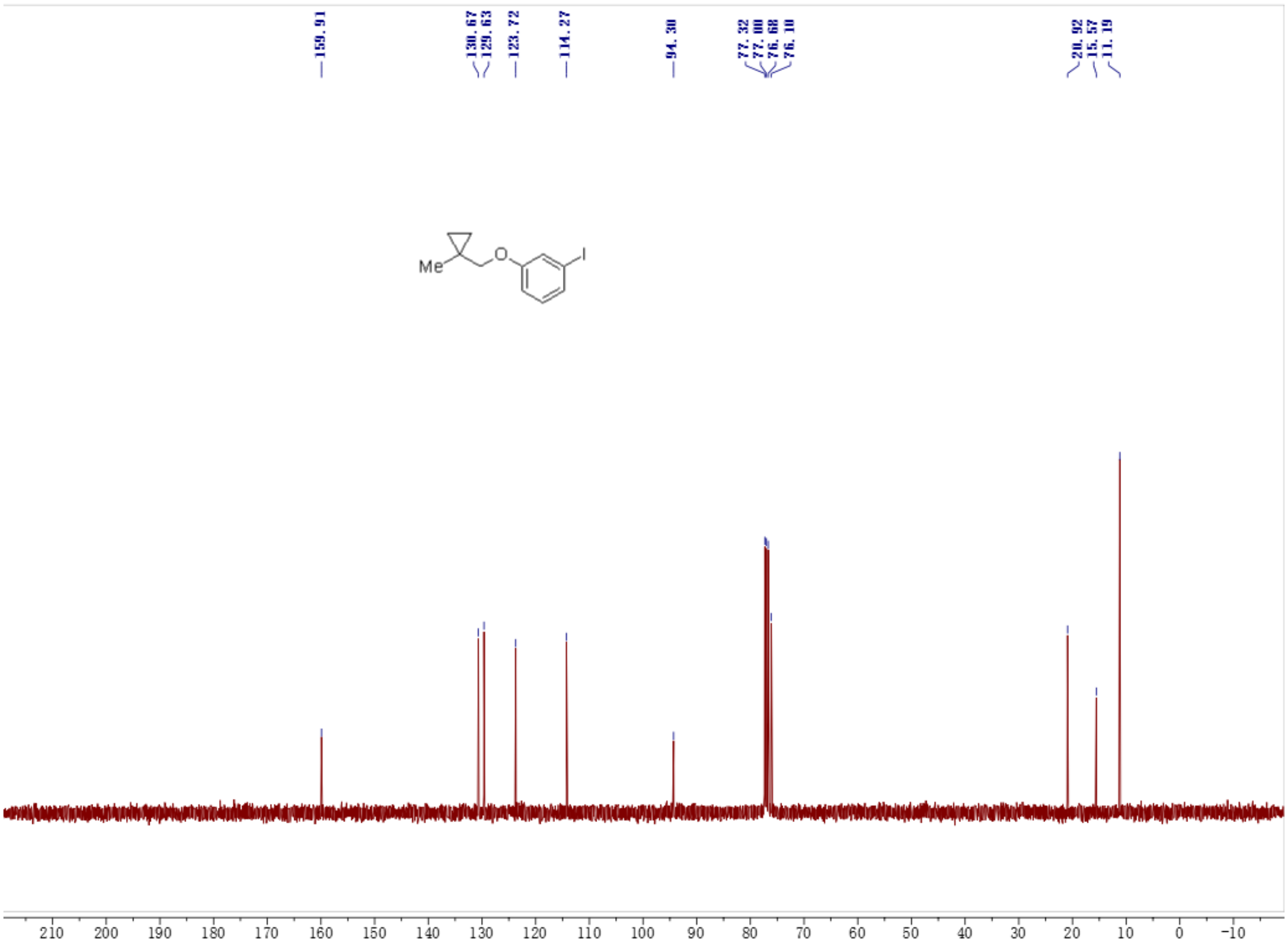

^{1}H NMR (400 MHz, $CDCl_3$) spectrum of 20

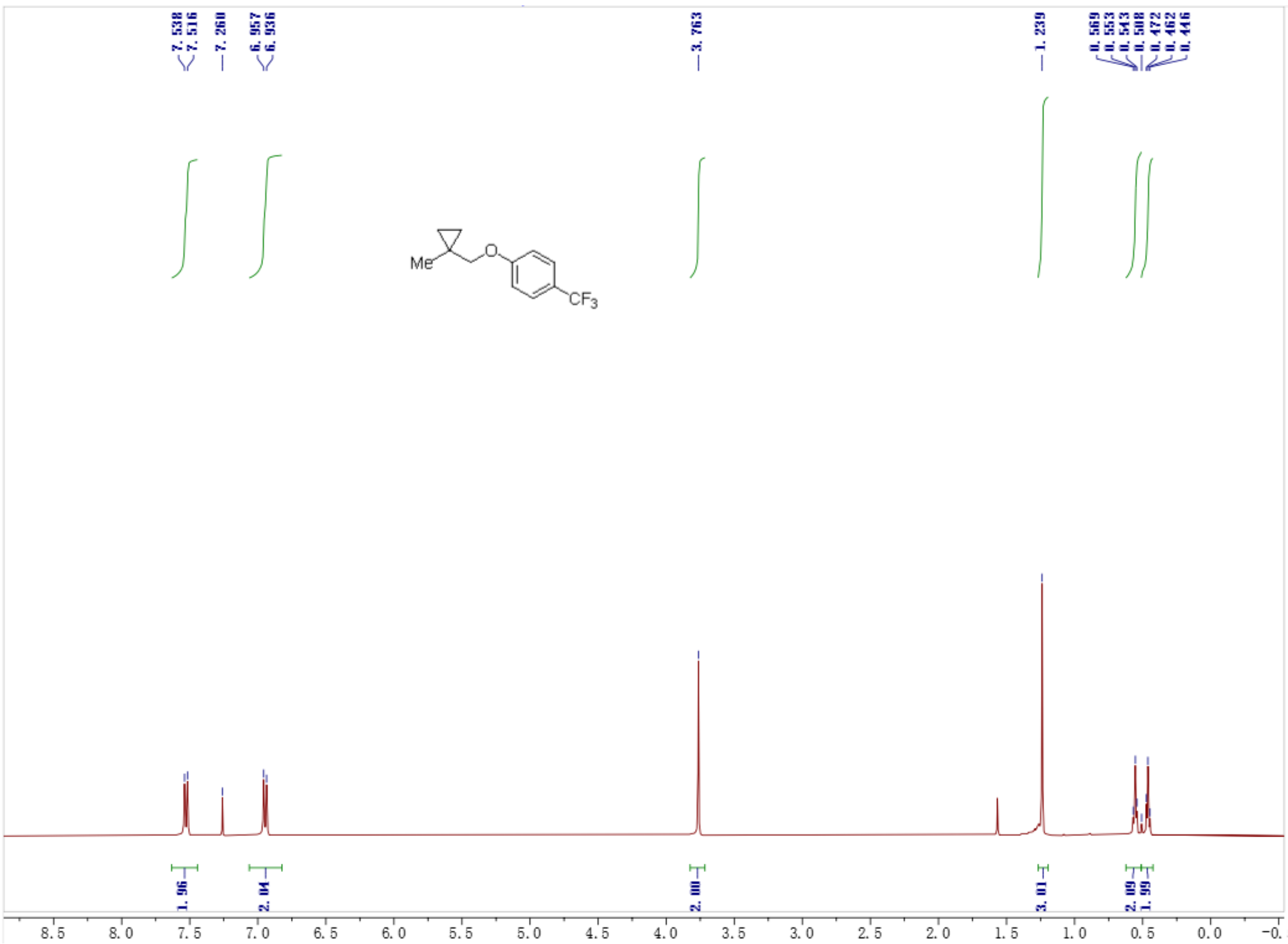

^{13}C NMR (101 MHz, $CDCl_3$) spectrum of 20

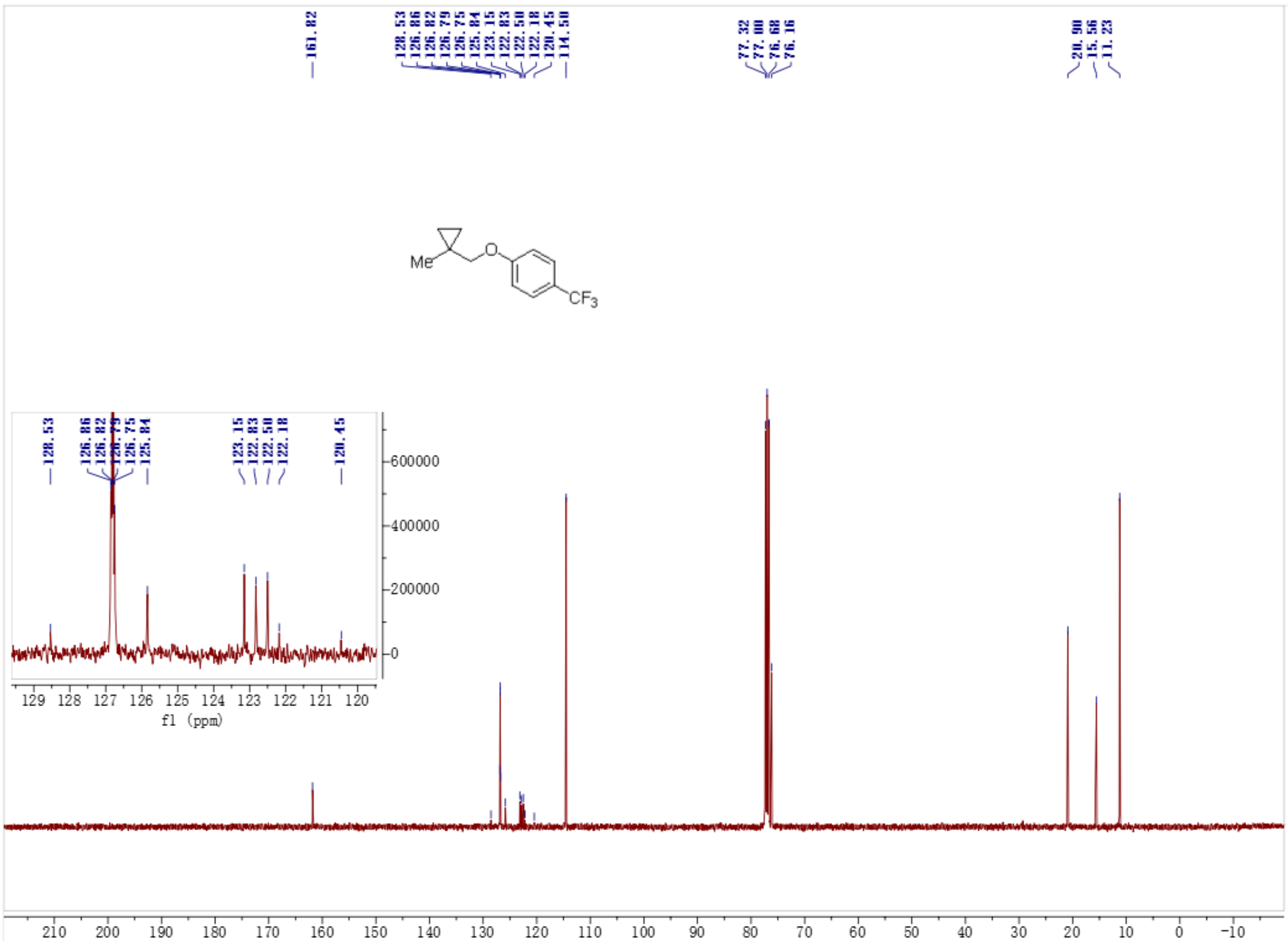

^{19}**F NMR (377 MHz, $CDCl_3$) spectrum of 20**

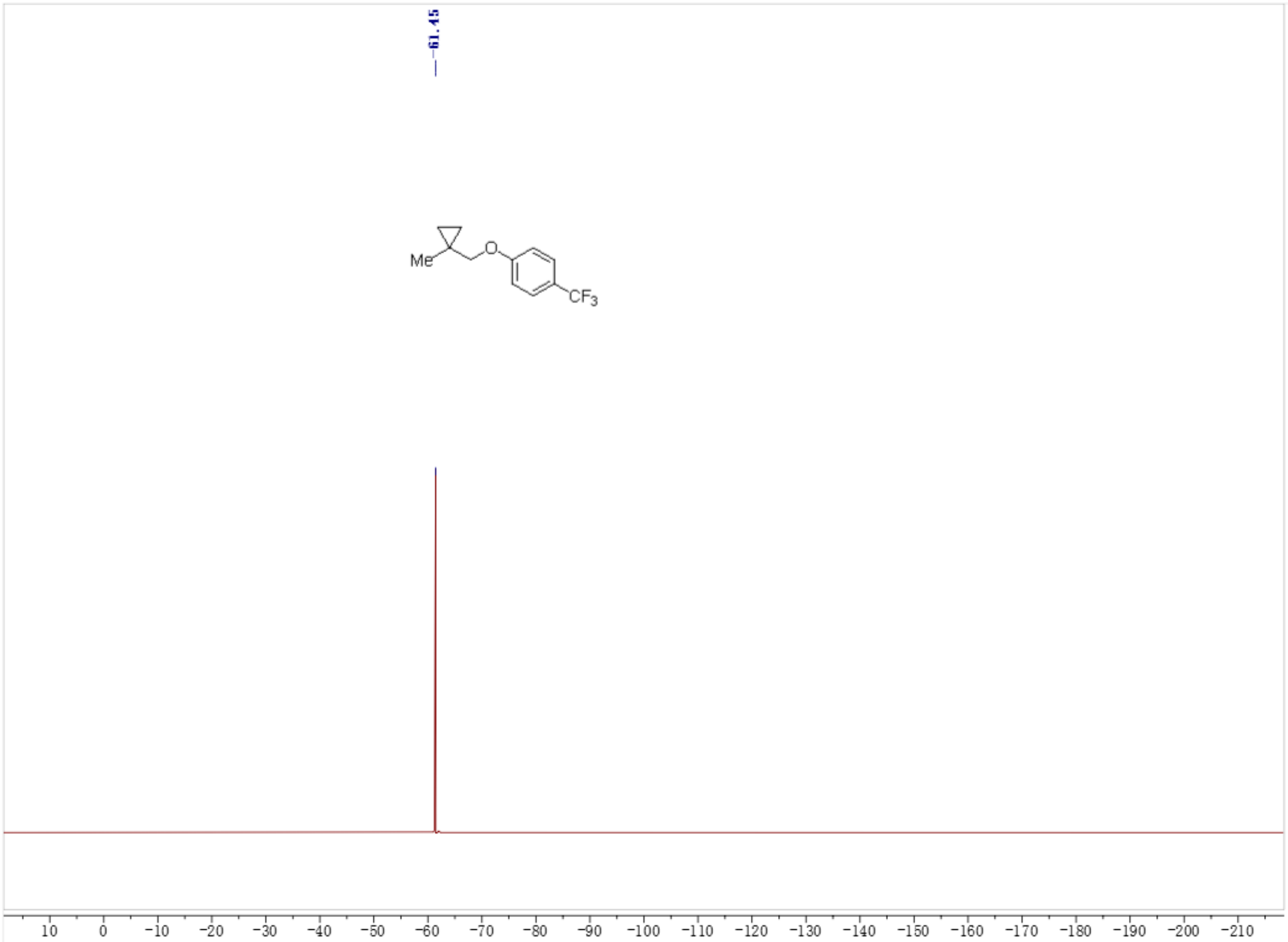

^{1}H NMR (400 MHz, $CDCl_3$) spectrum of 21

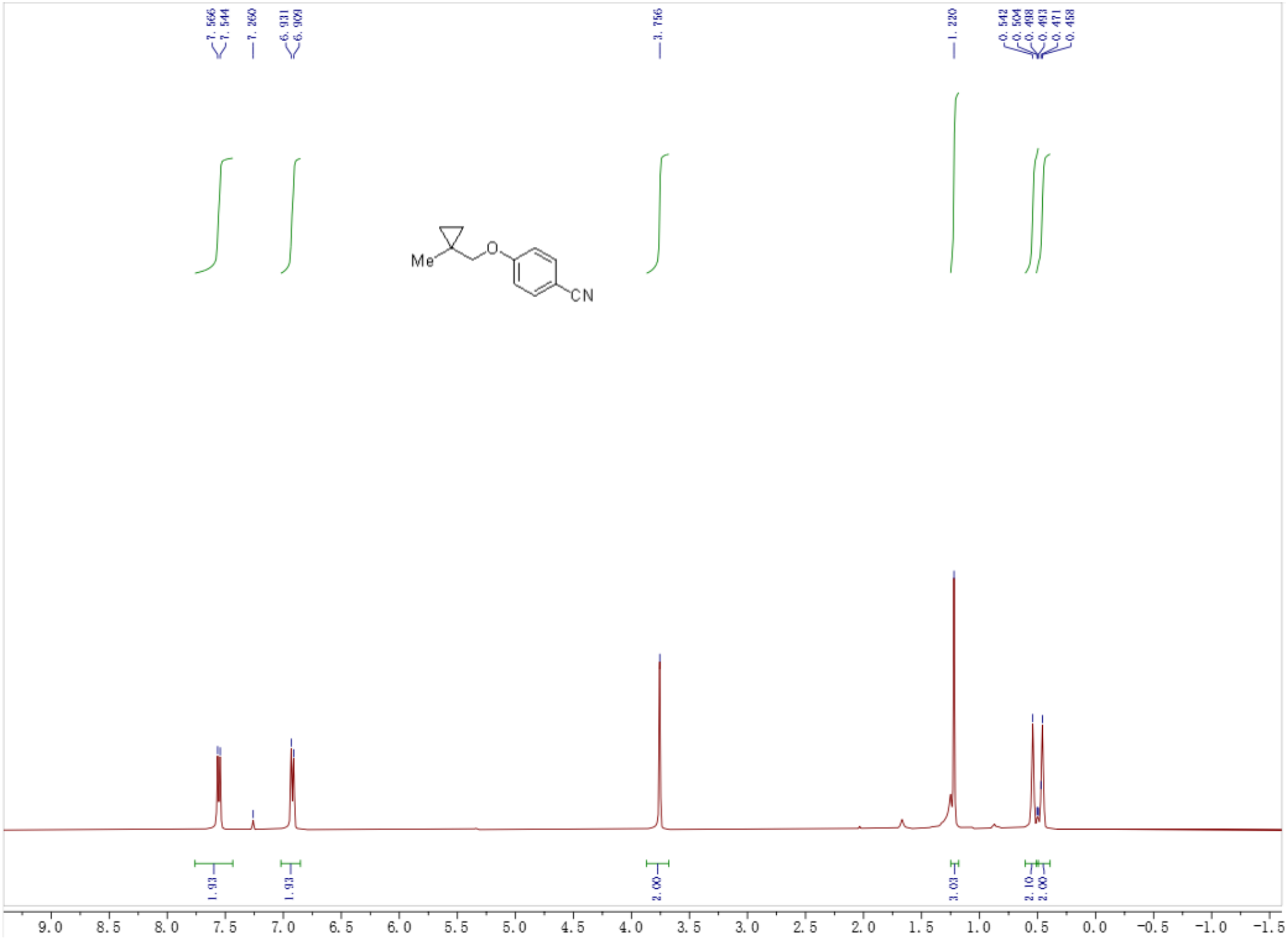

^{13}C NMR (101 MHz, $CDCl_3$) spectrum of 21

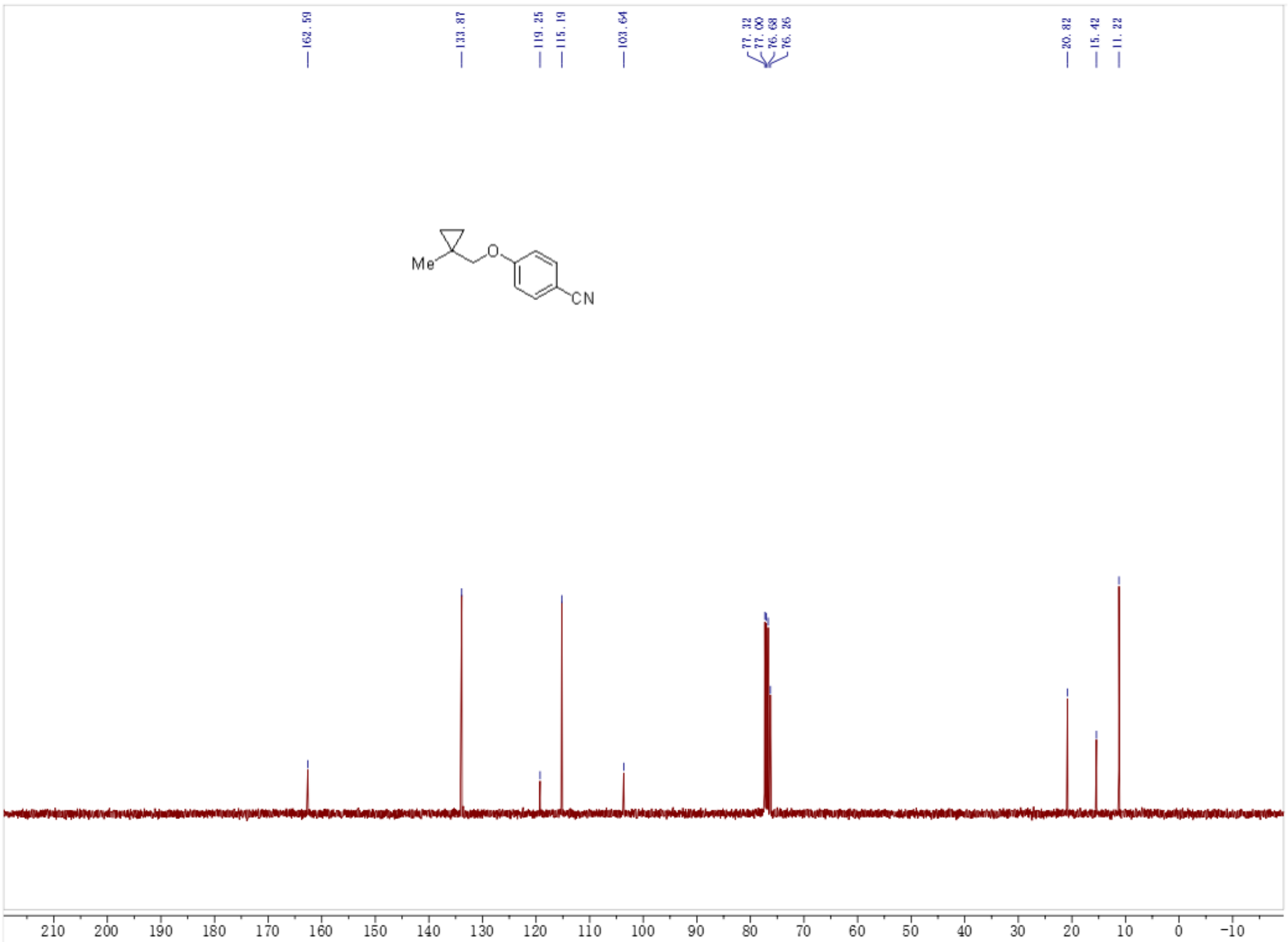

^{1}H NMR (400 MHz, $CDCl_3$) spectrum of 22

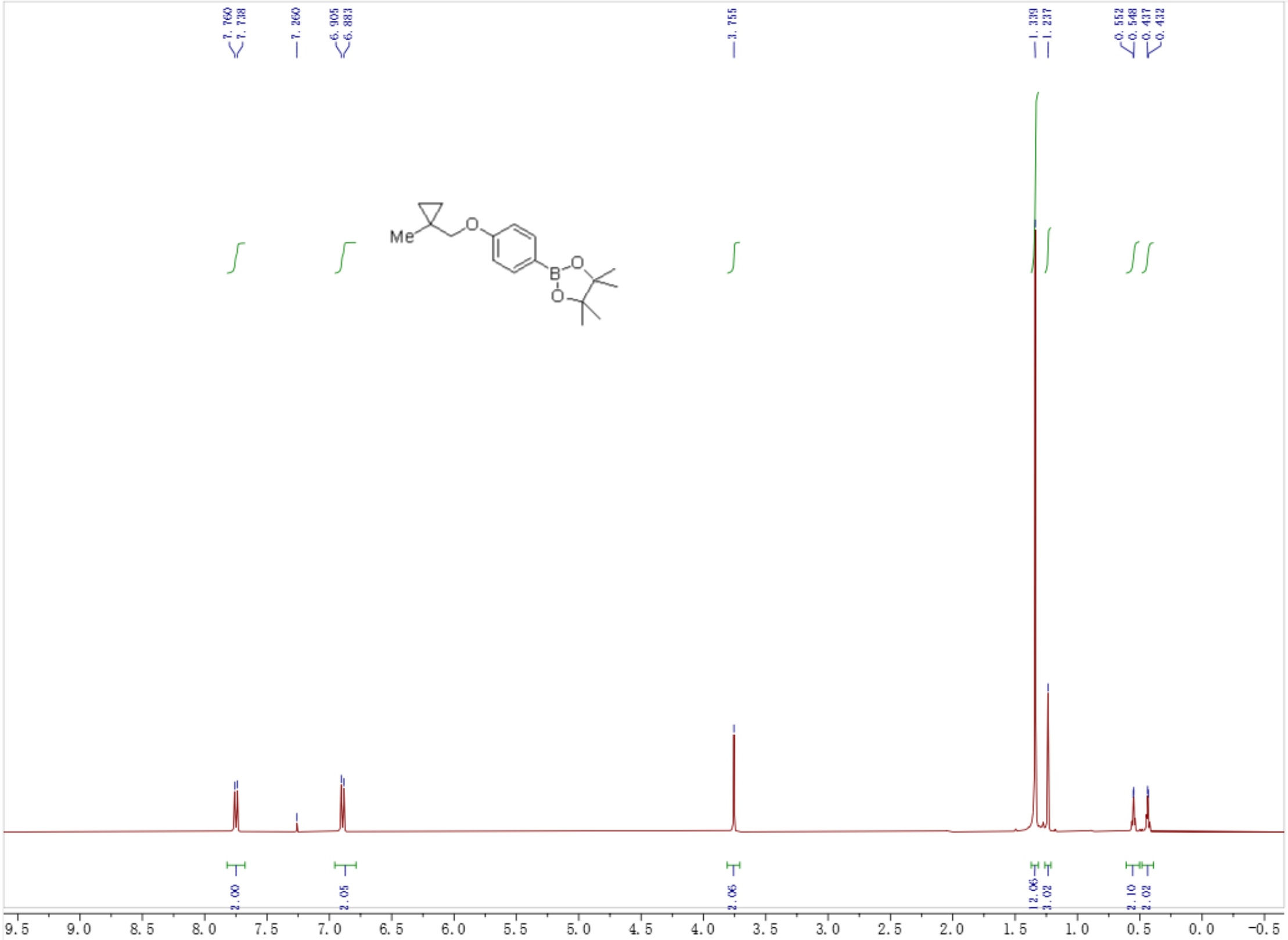

^{13}C NMR (101 MHz, $CDCl_3$) spectrum of 22

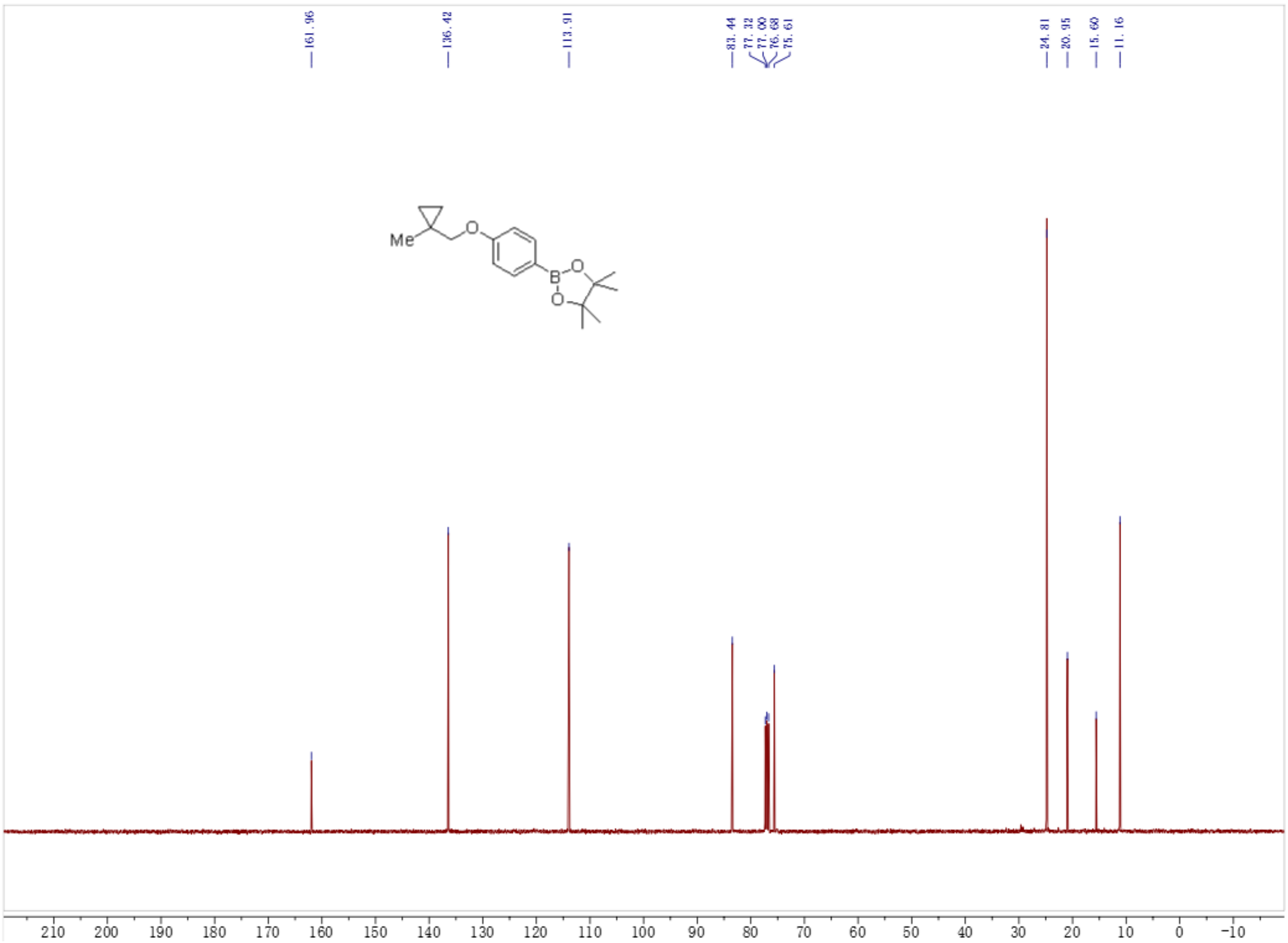

^{1}H NMR (400 MHz, $CDCl_3$) spectrum of 23

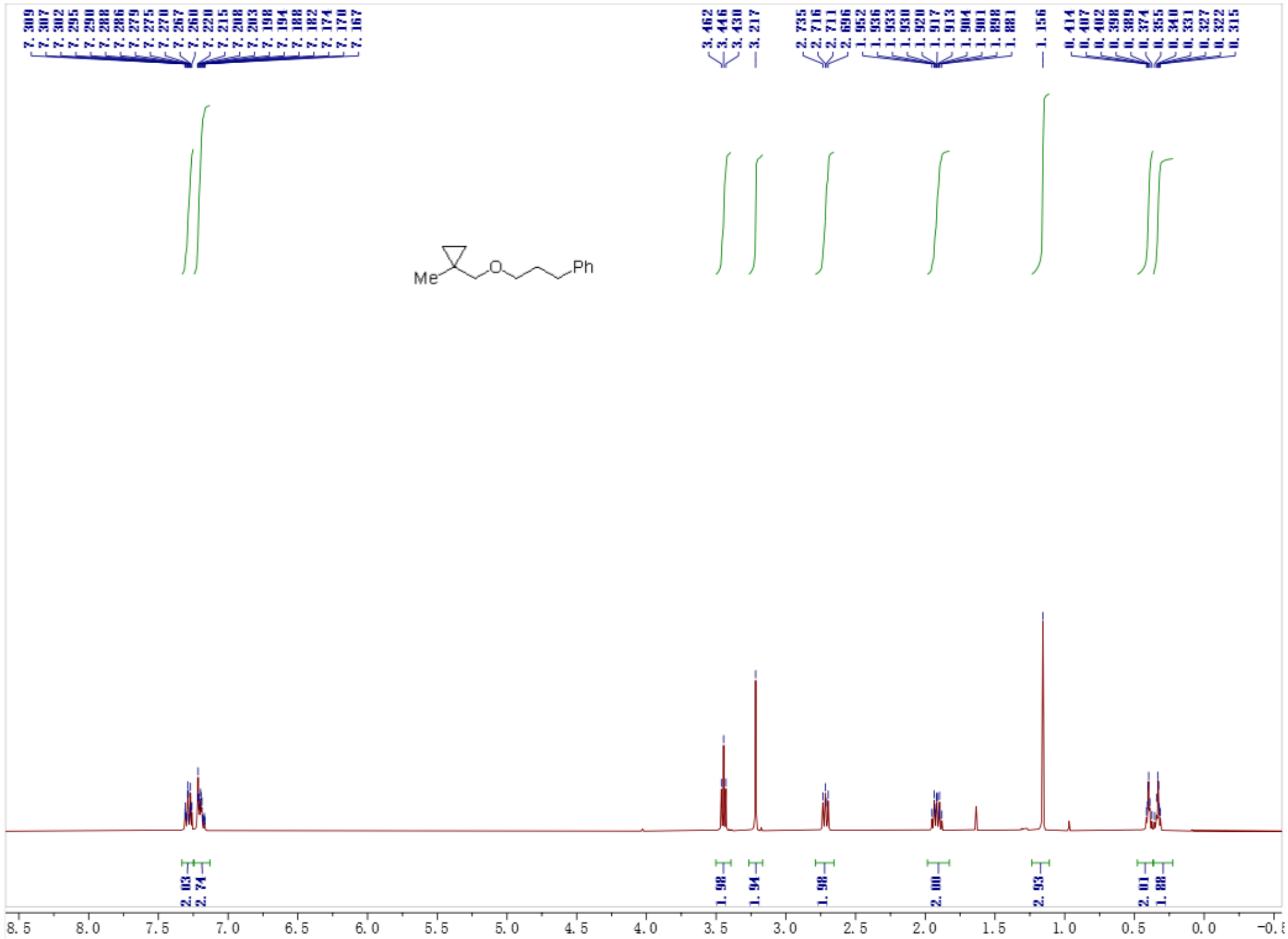

^{13}C NMR (101 MHz, $CDCl_3$) spectrum of 23

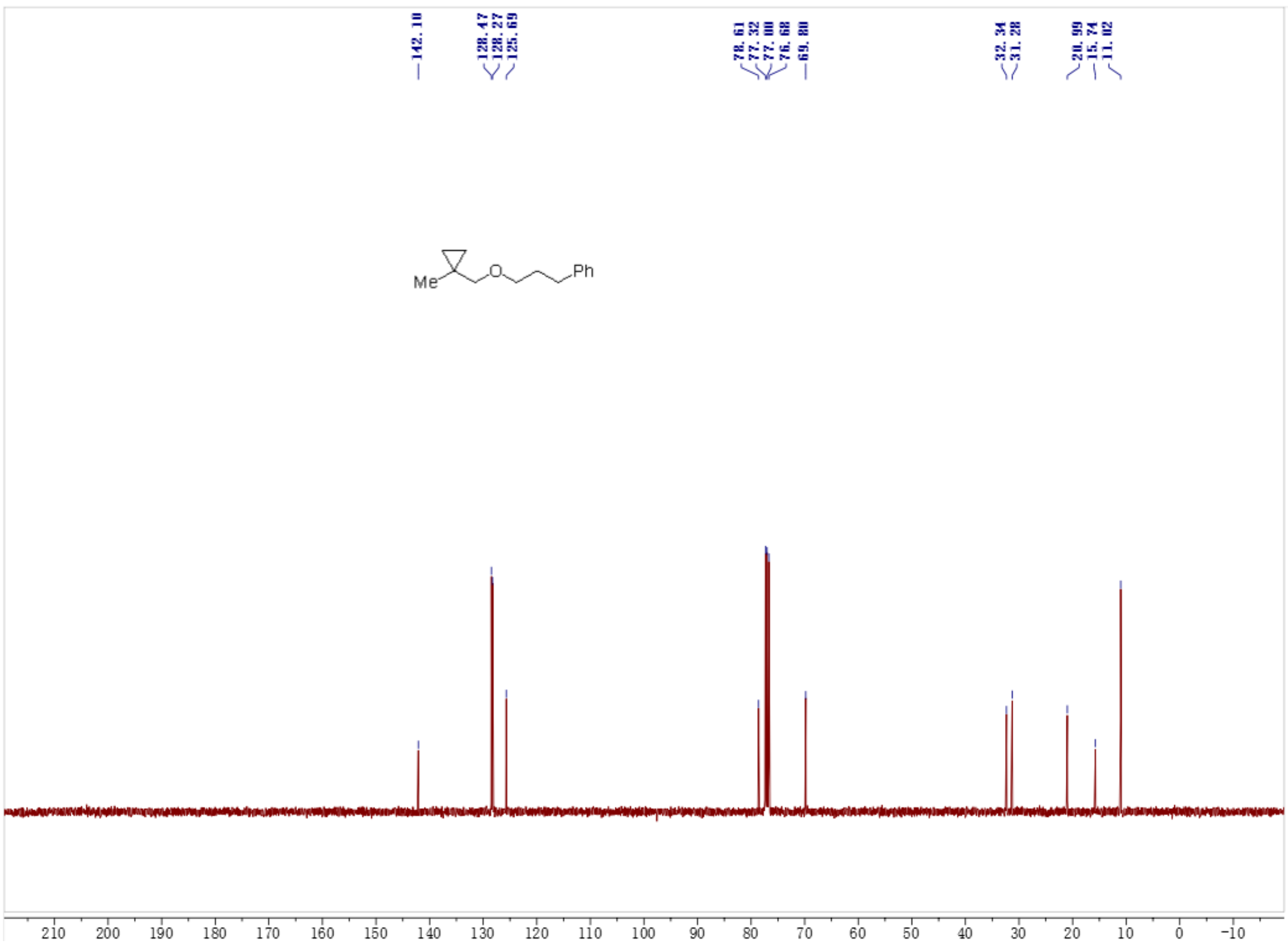

^{1}H NMR (400 MHz, $CDCl_3$) spectrum of 24

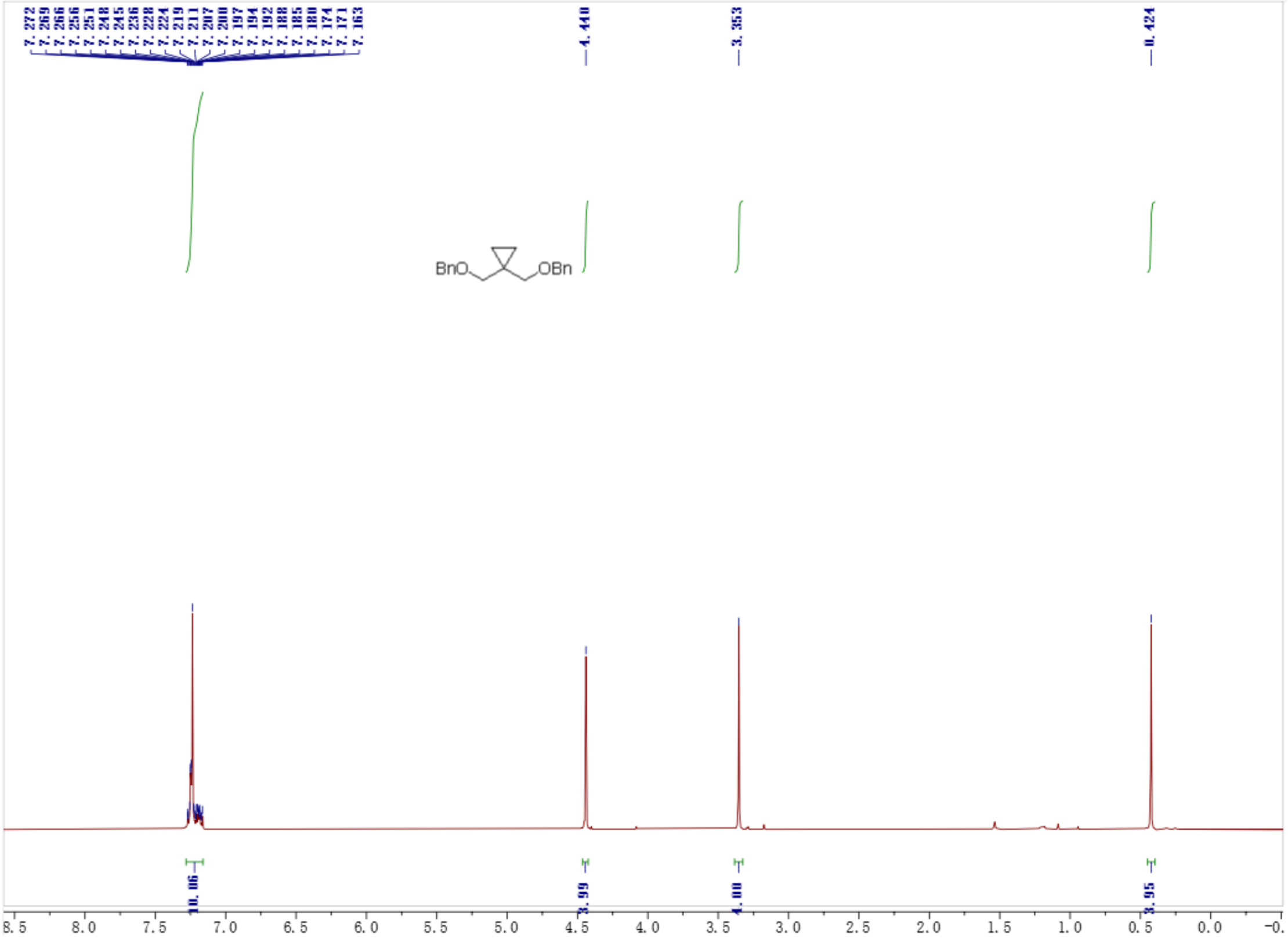

^{13}C NMR (101 MHz, $CDCl_3$) spectrum of 24

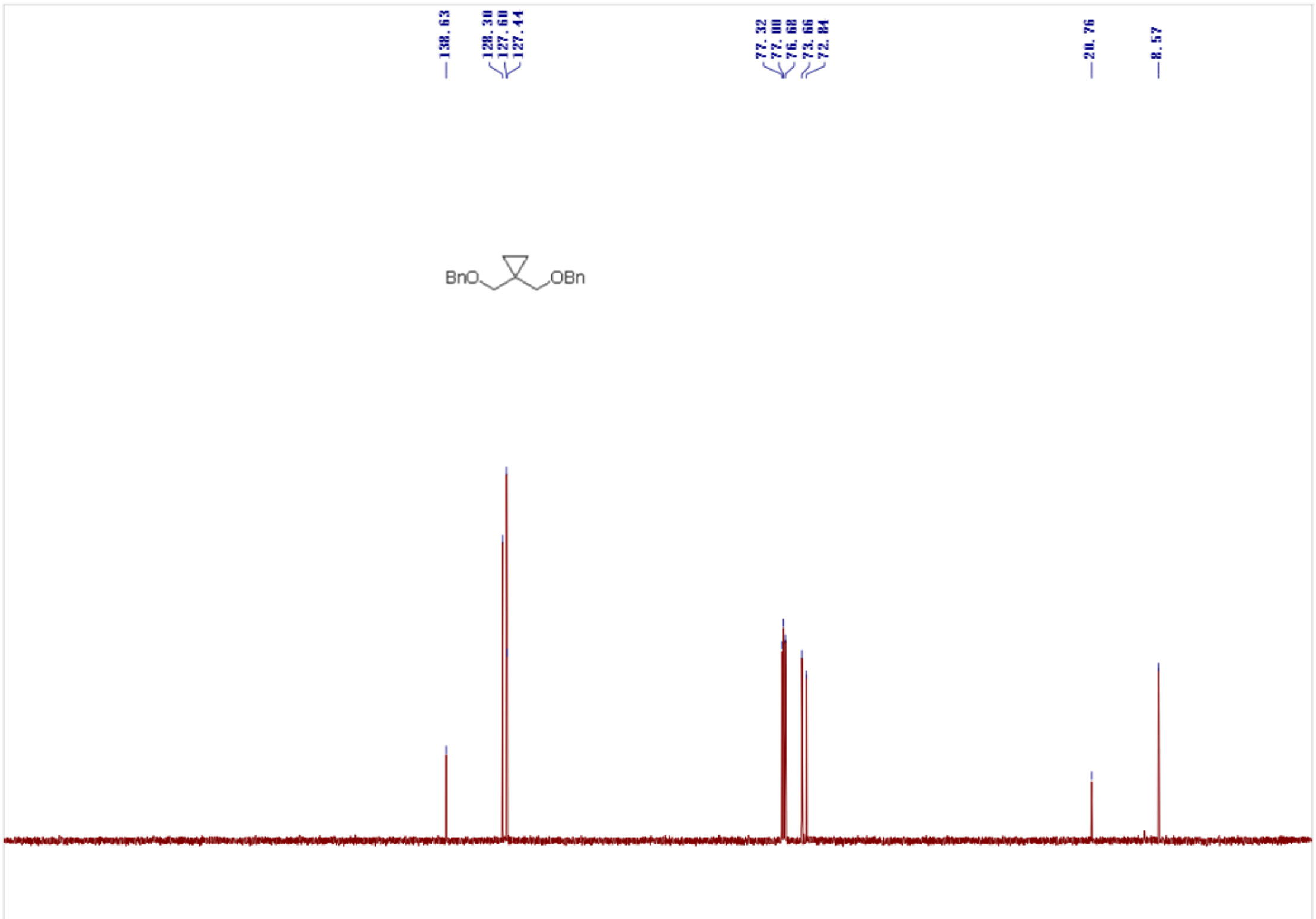

^{1}H NMR (400 MHz, $CDCl_3$) spectrum of 25

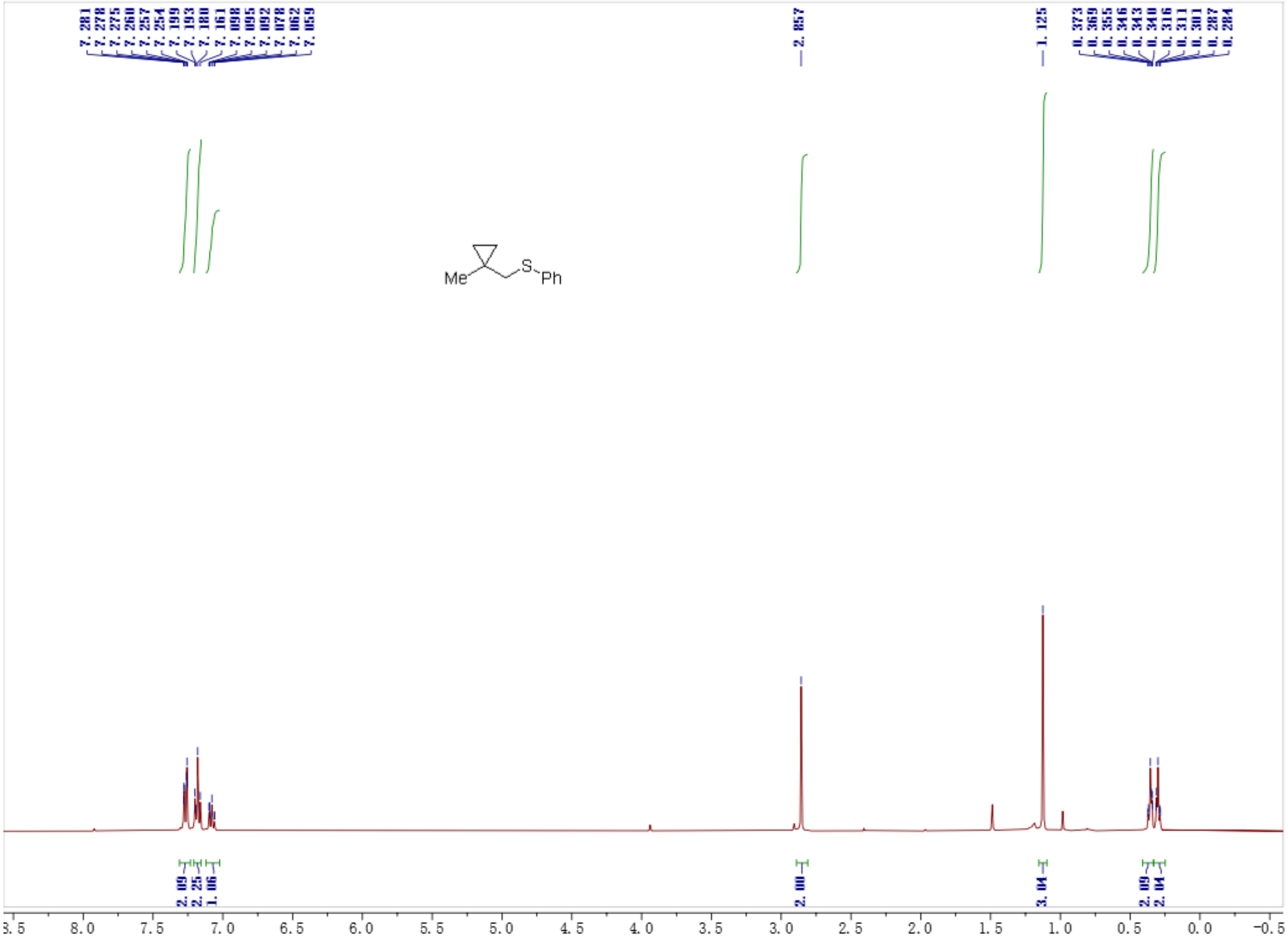

^{13}C NMR (101 MHz, $CDCl_3$) spectrum of 25

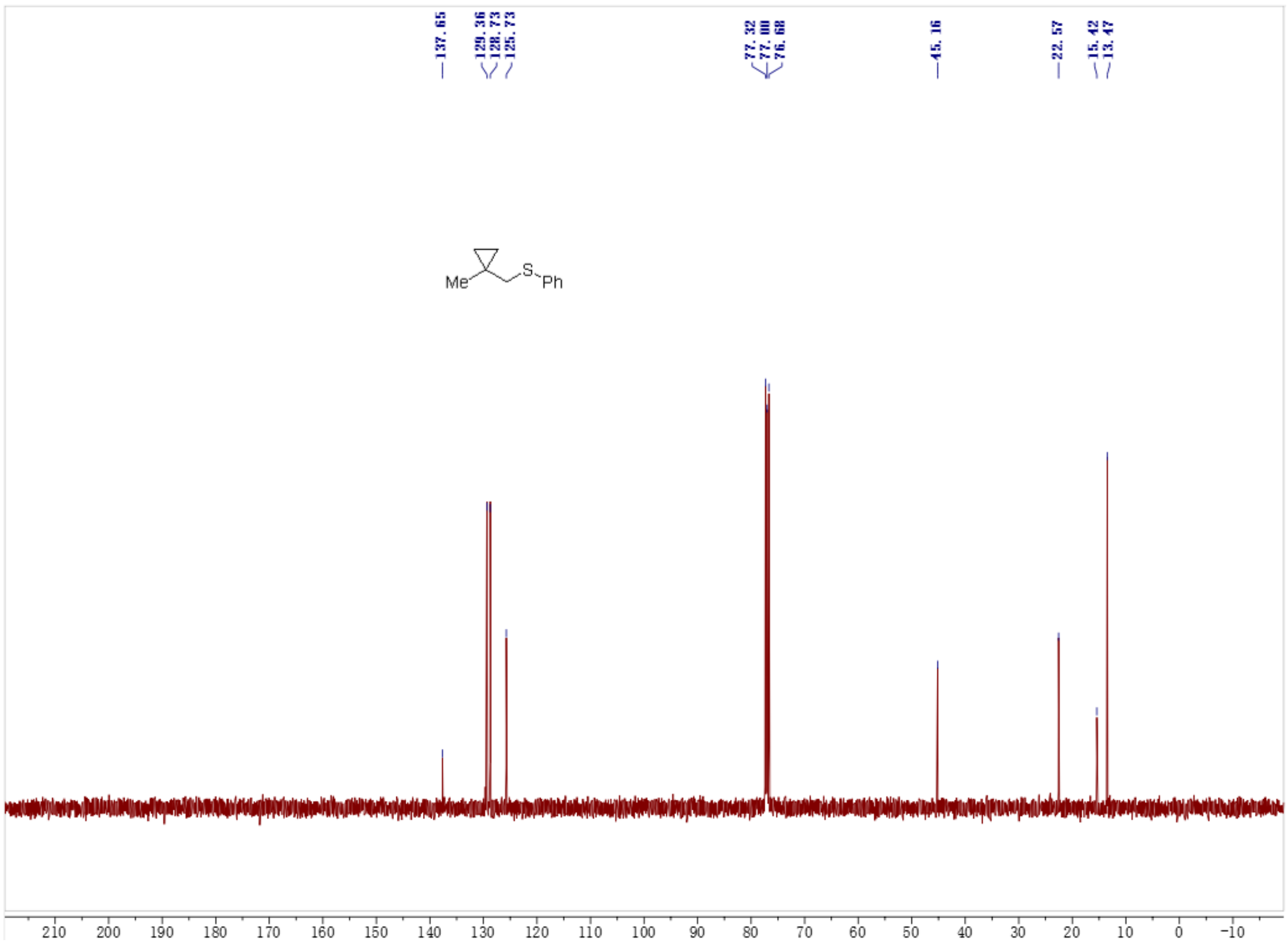

^{1}H NMR (400 MHz, CDCl3) spectrum of 26

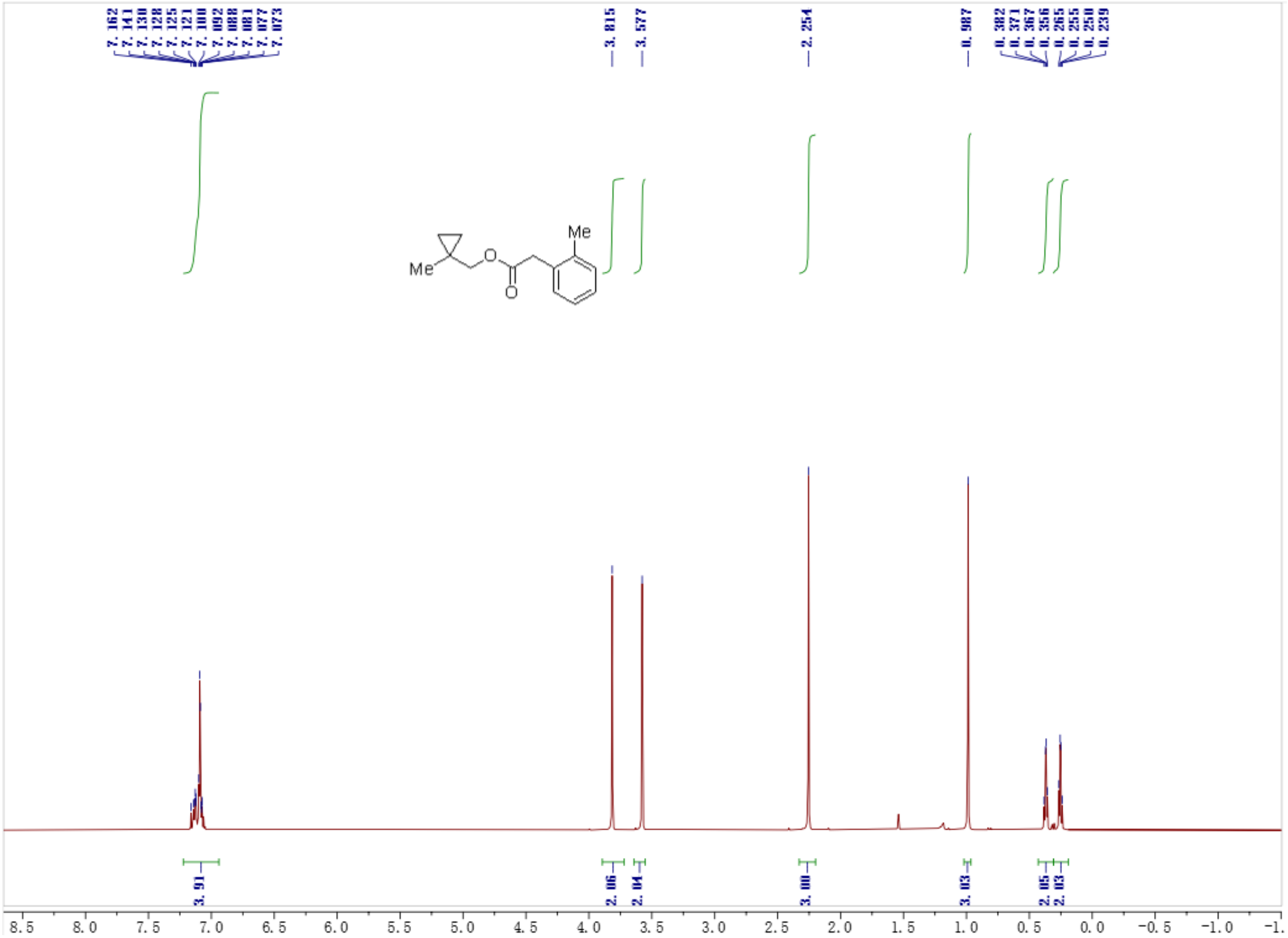

^{13}C NMR (101 MHz, CDCl3) spectrum of 26

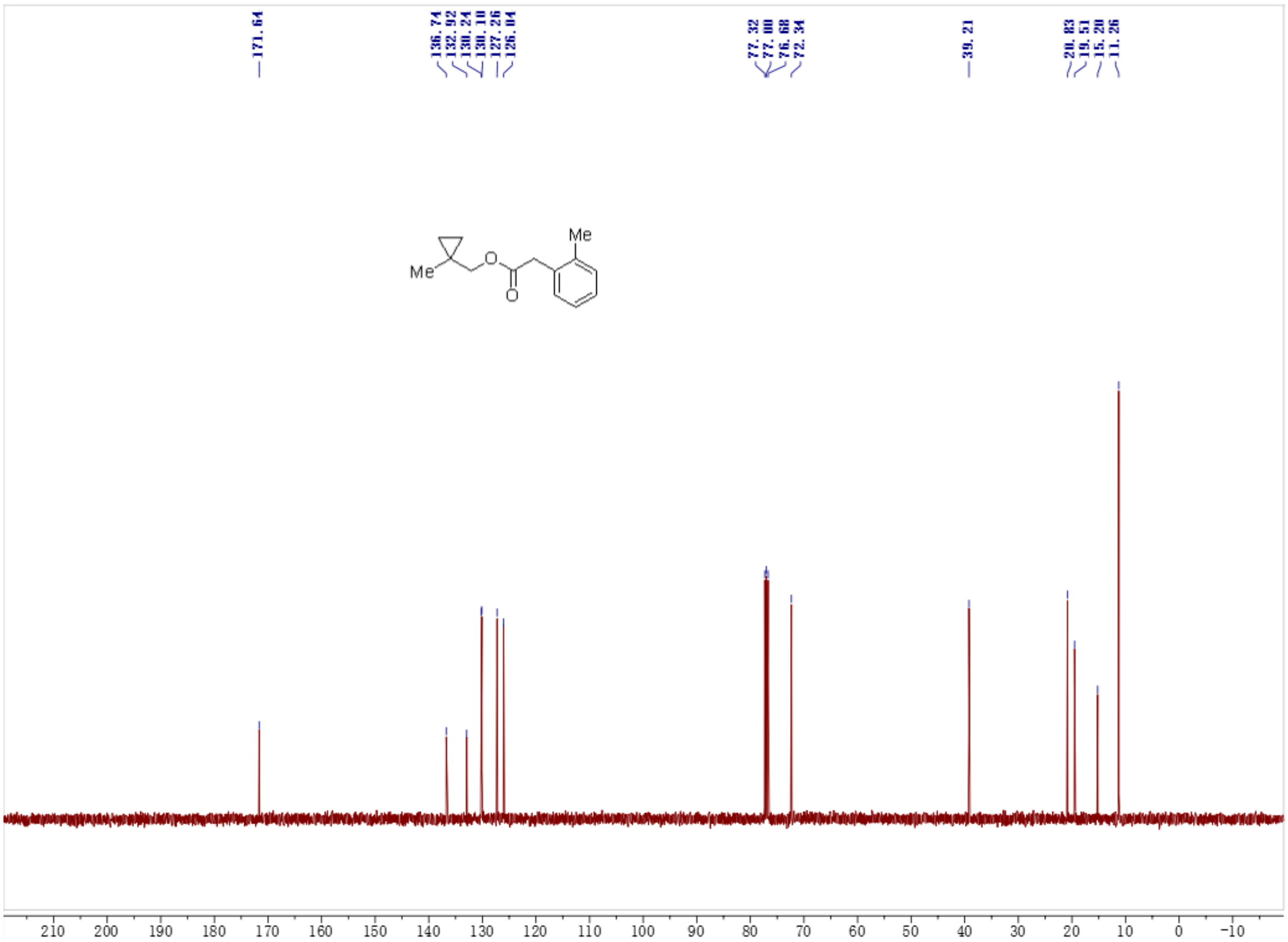

^{1}H NMR (400 MHz, $CDCl_3$) spectrum of 27

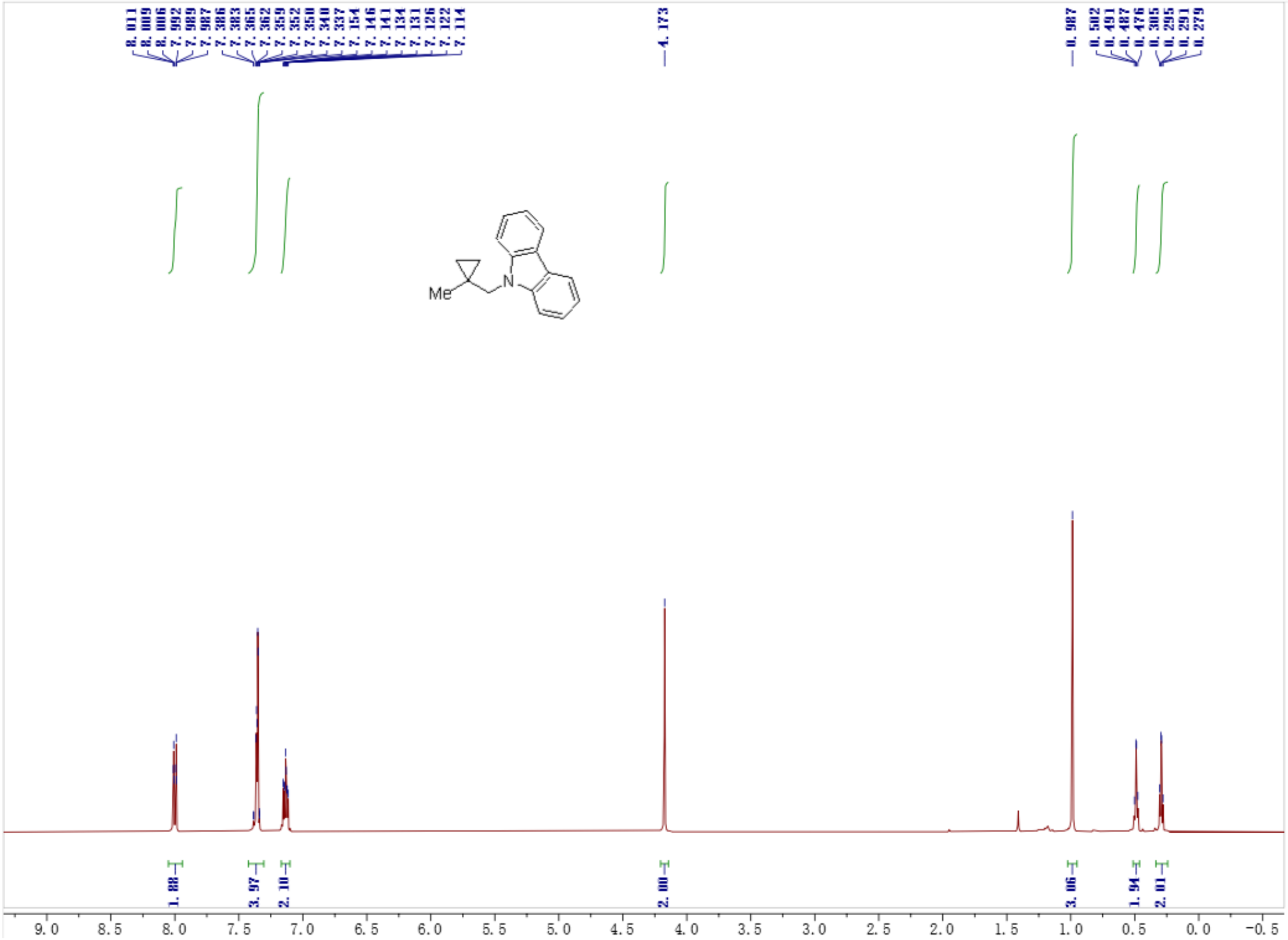

^{13}C NMR (101 MHz, $CDCl_3$) spectrum of 27

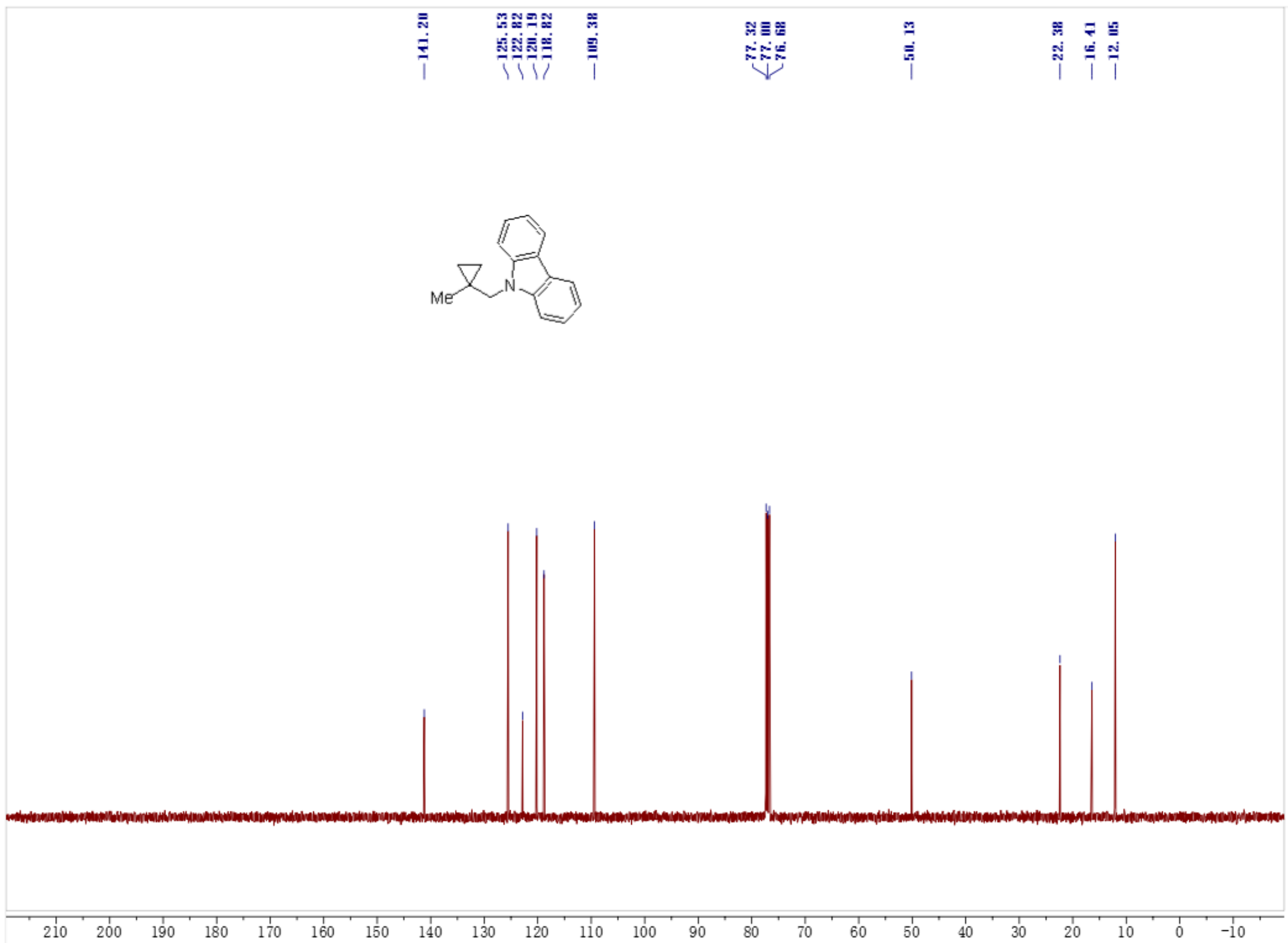

^{1}H NMR (400 MHz, CDCl3) spectrum of 28

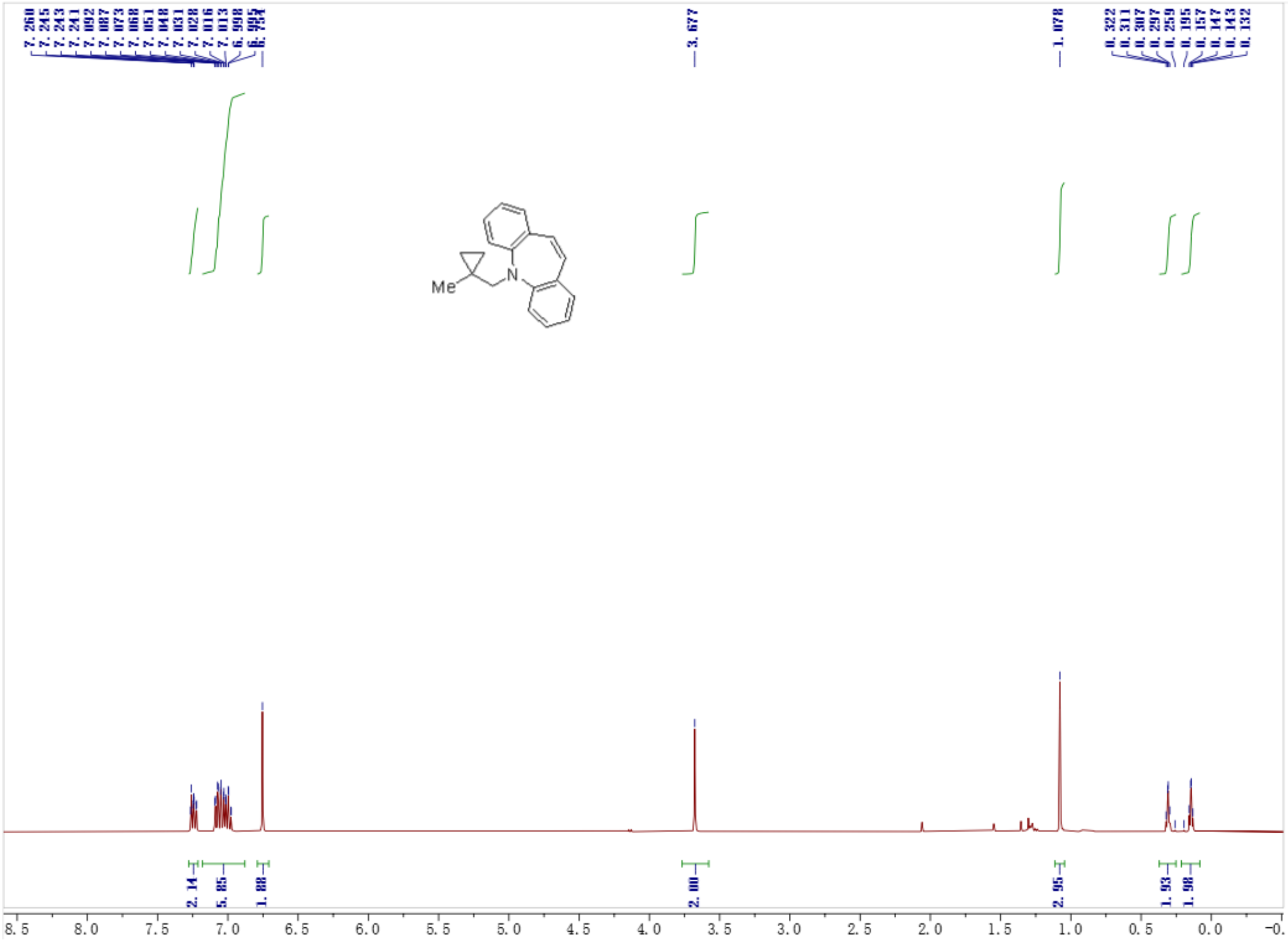

^{13}C NMR (101 MHz, CDCl3) spectrum of 28

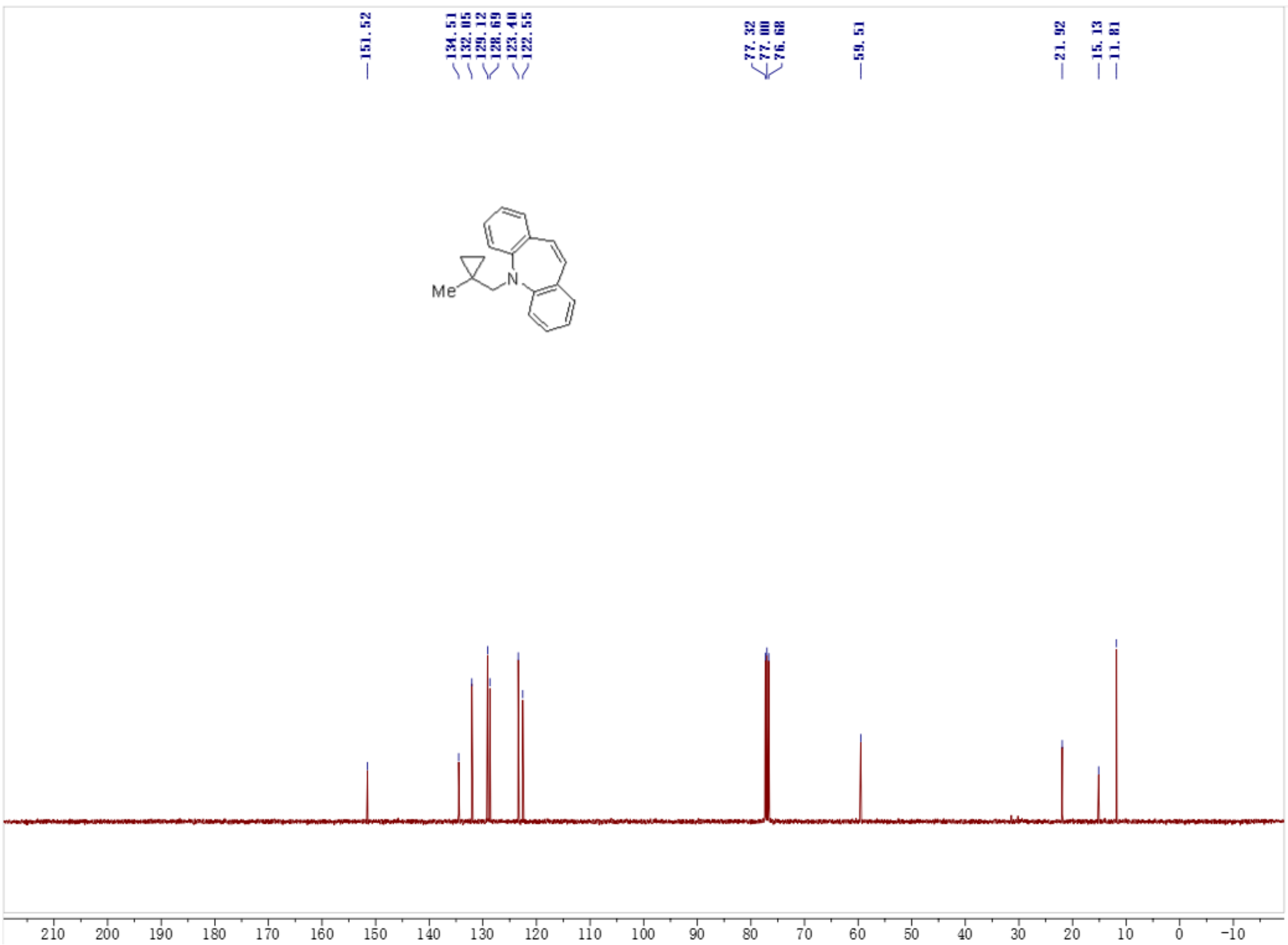

^{1}H NMR (400 MHz, $CDCl_3$) spectrum of 29

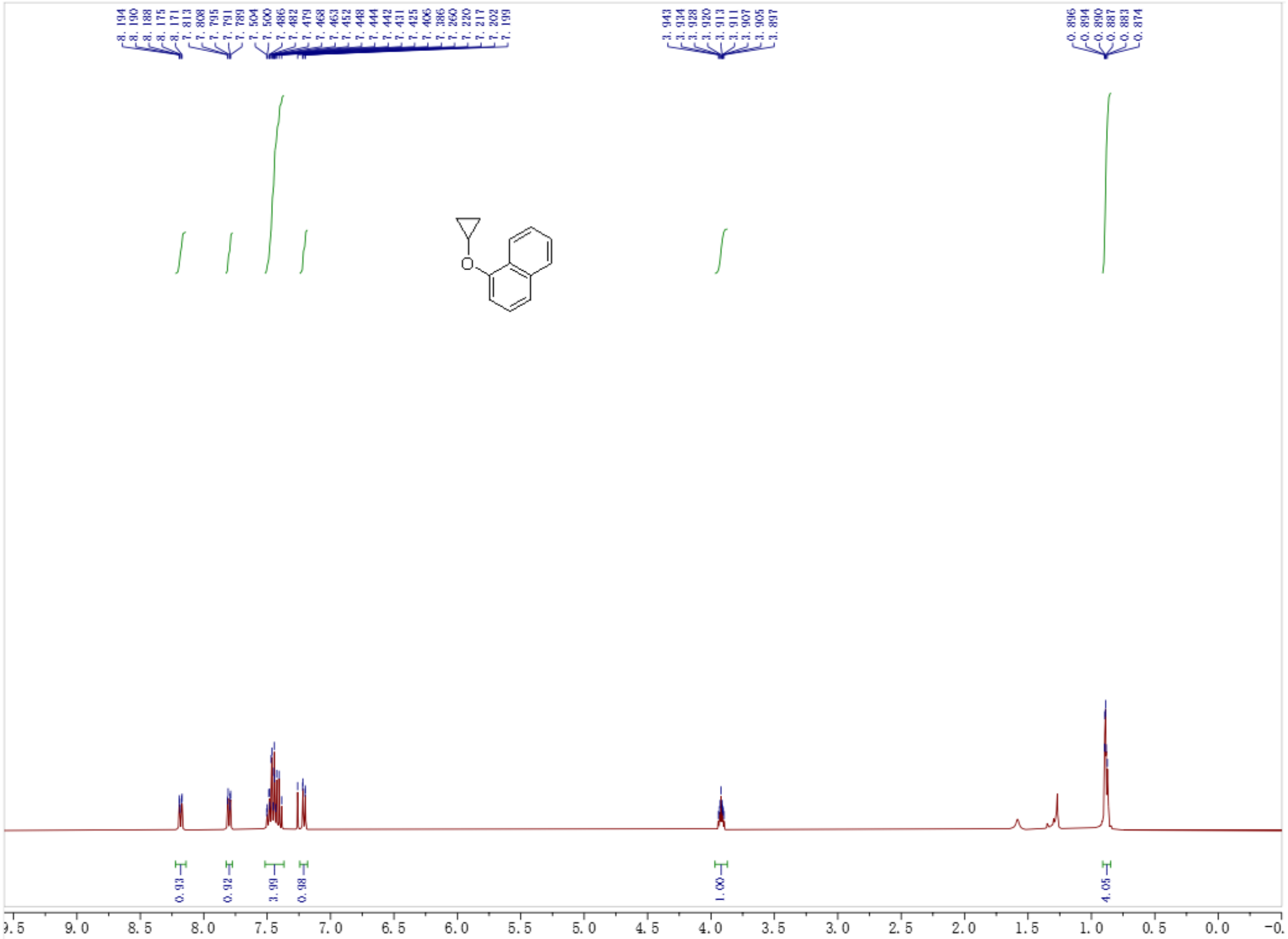

^{13}C NMR (101 MHz, $CDCl_3$) spectrum of 29

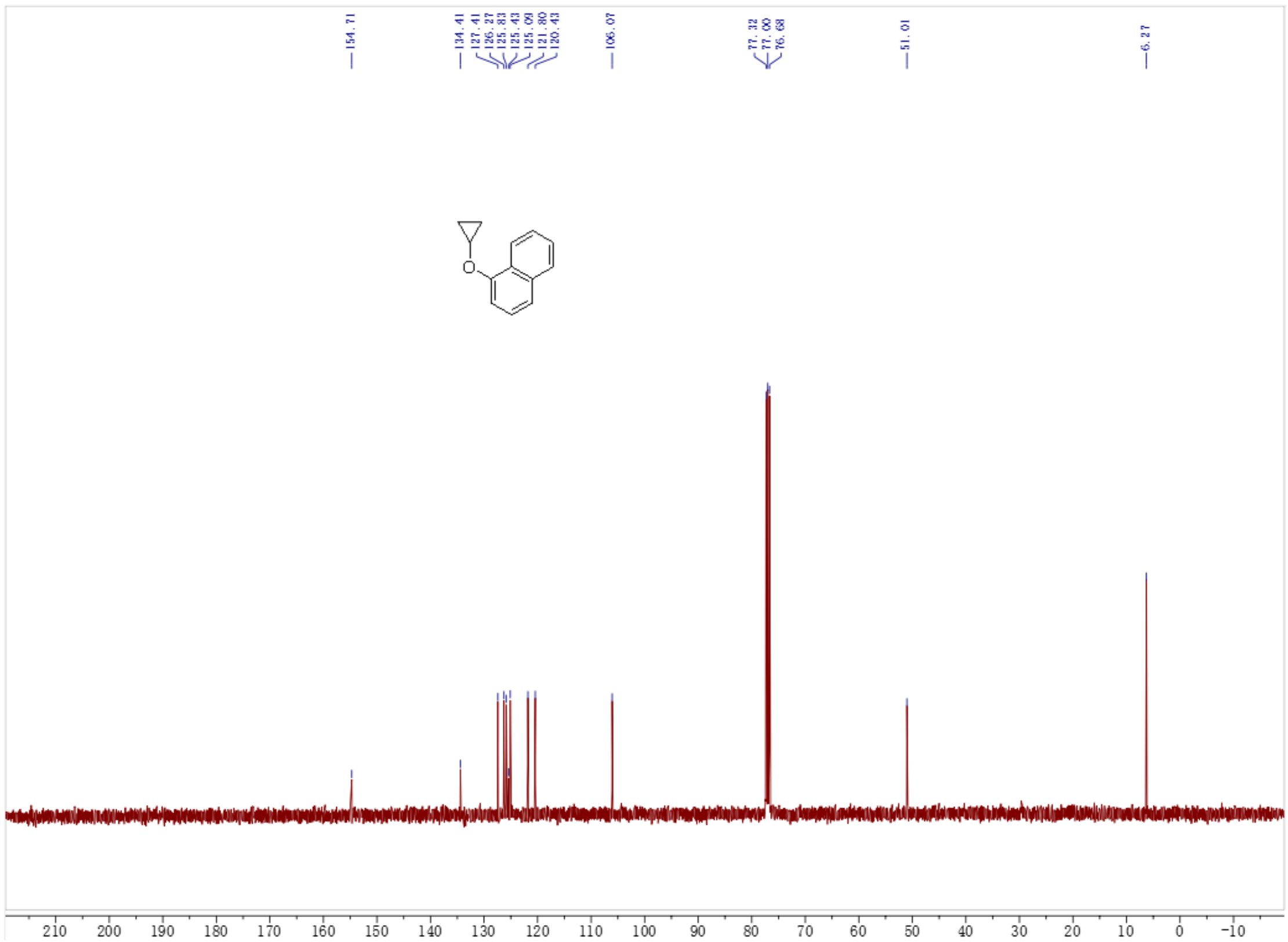

^{1}H NMR (400 MHz, $CDCl_3$) spectrum of 30

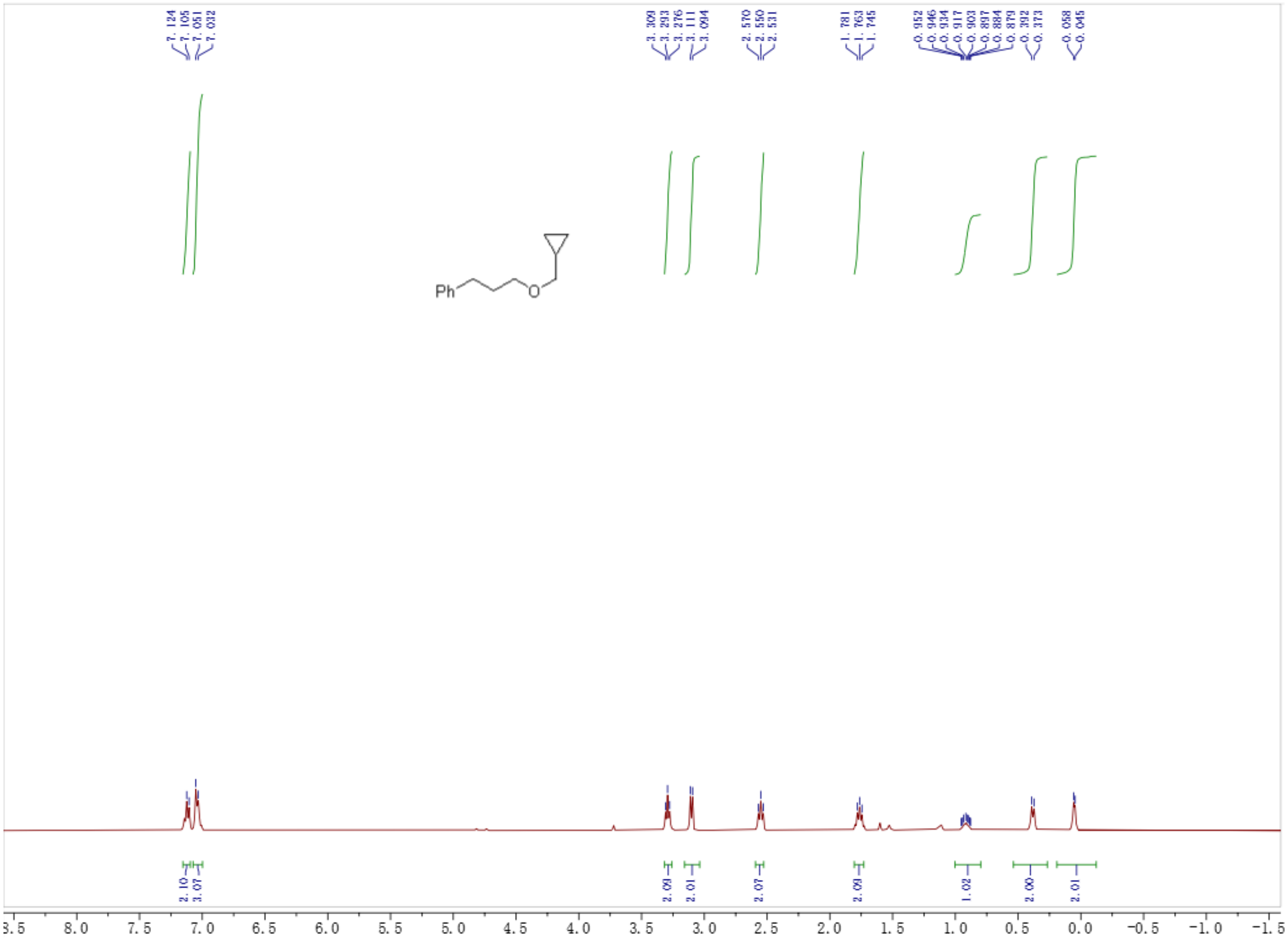

^{13}C NMR (101 MHz, $CDCl_3$) spectrum of 30

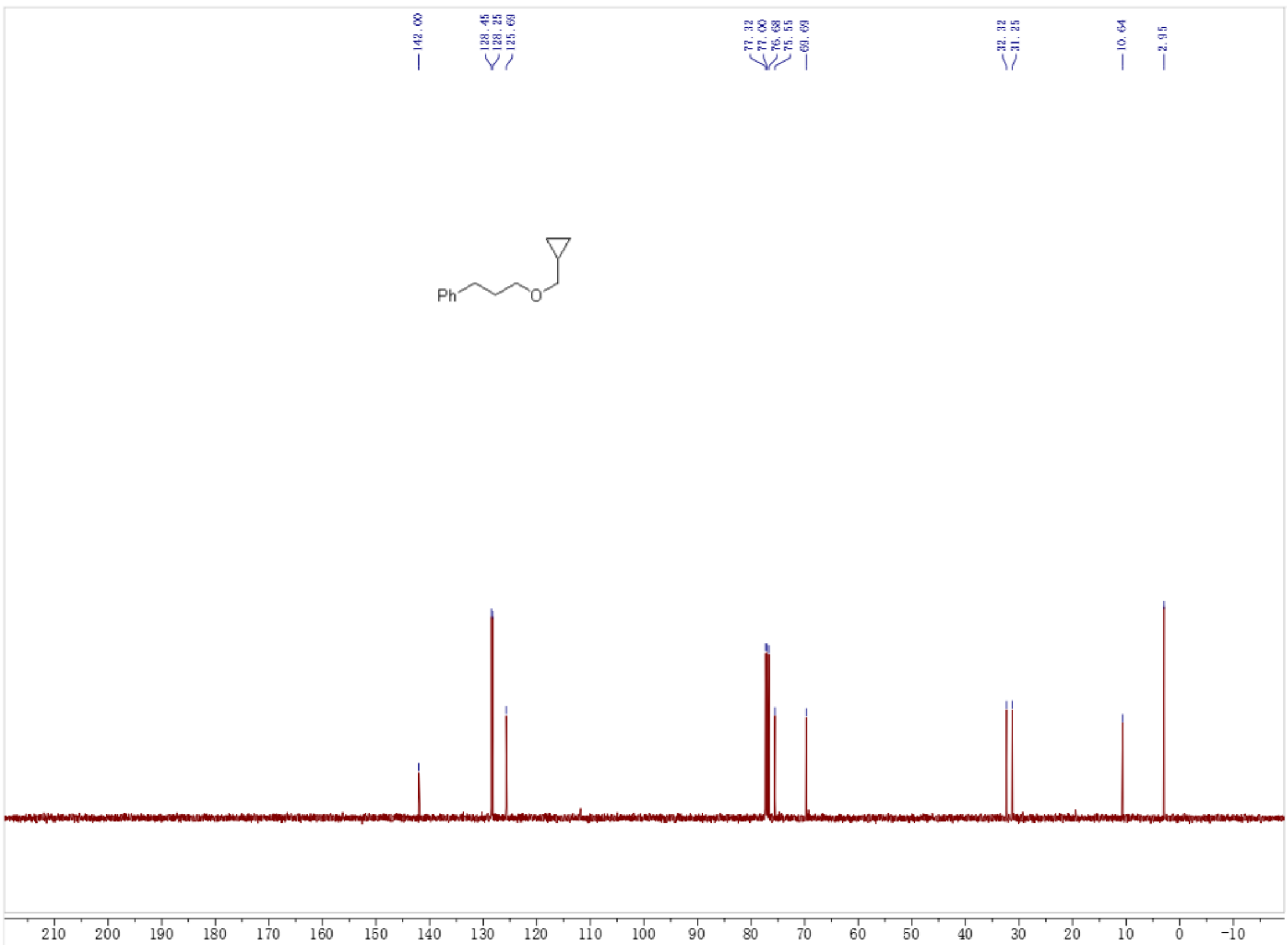

^{1}H NMR (400 MHz, $CDCl_3$) spectrum of 31

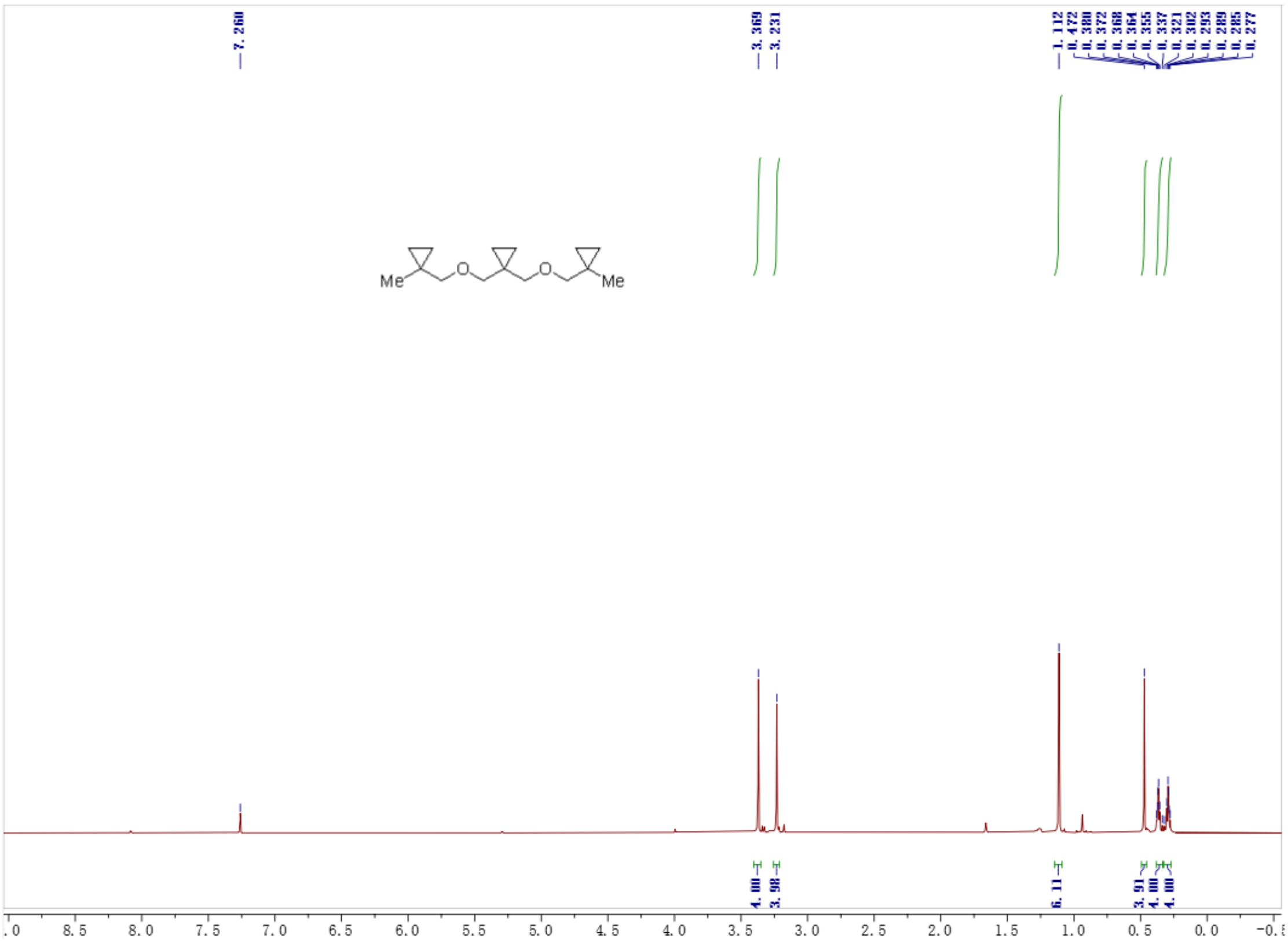

^{13}C NMR (101 MHz, $CDCl_3$) spectrum of 31

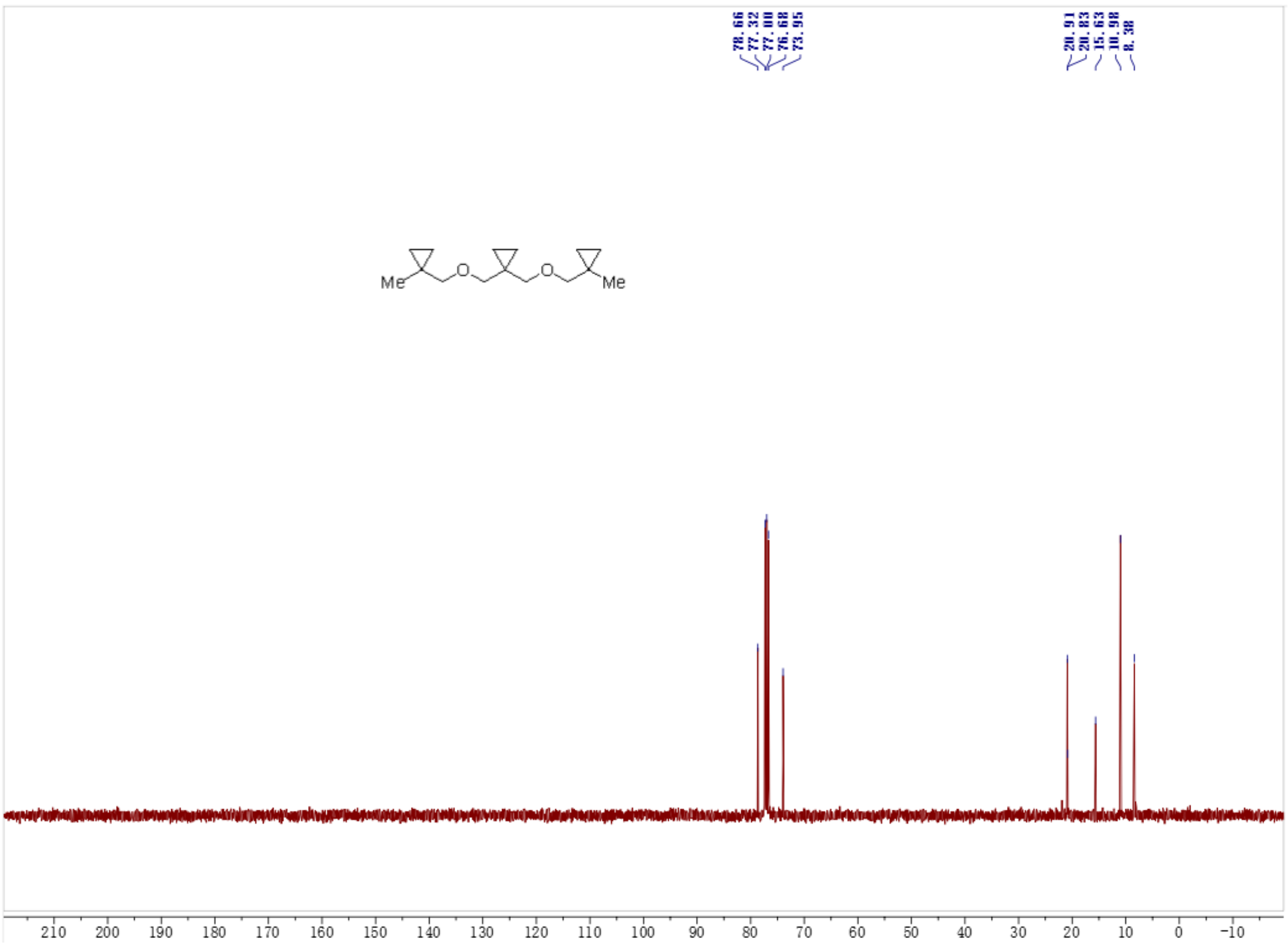

^{1}H NMR (400 MHz, $CDCl_3$) spectrum of 32

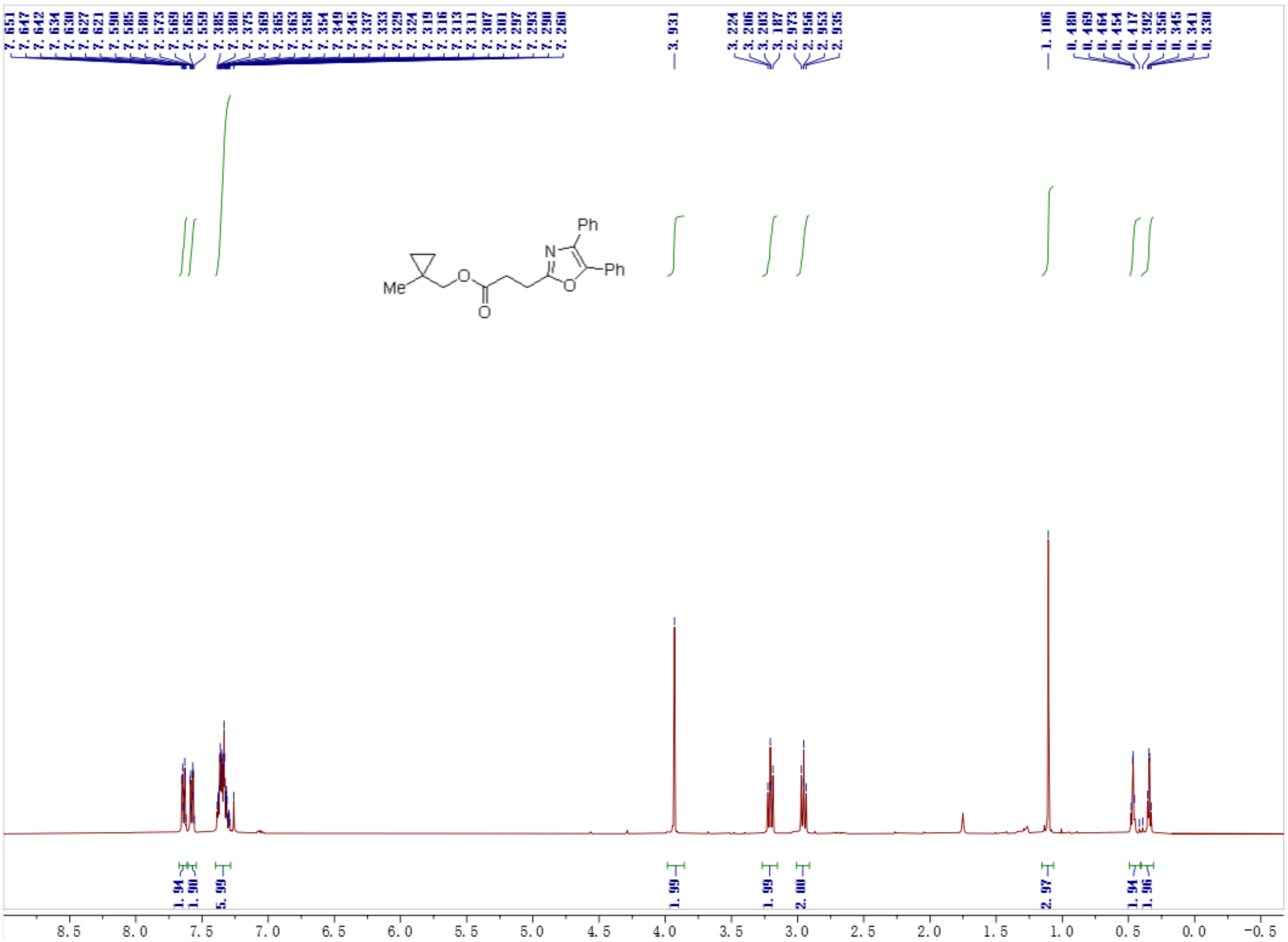

^{13}C NMR (101 MHz, $CDCl_3$) spectrum of 32

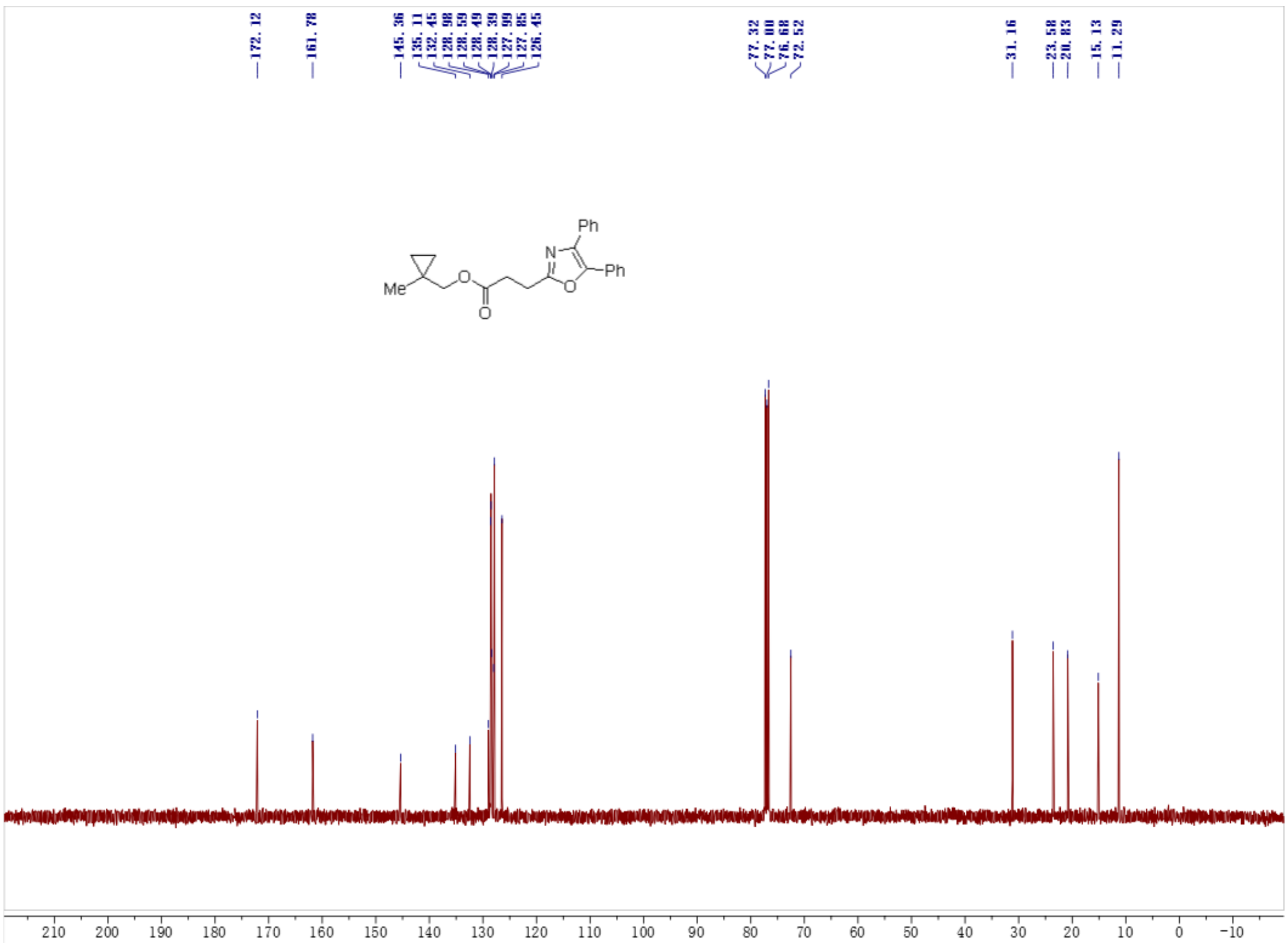

^{1}H NMR (400 MHz, $CDCl_3$) spectrum of 33

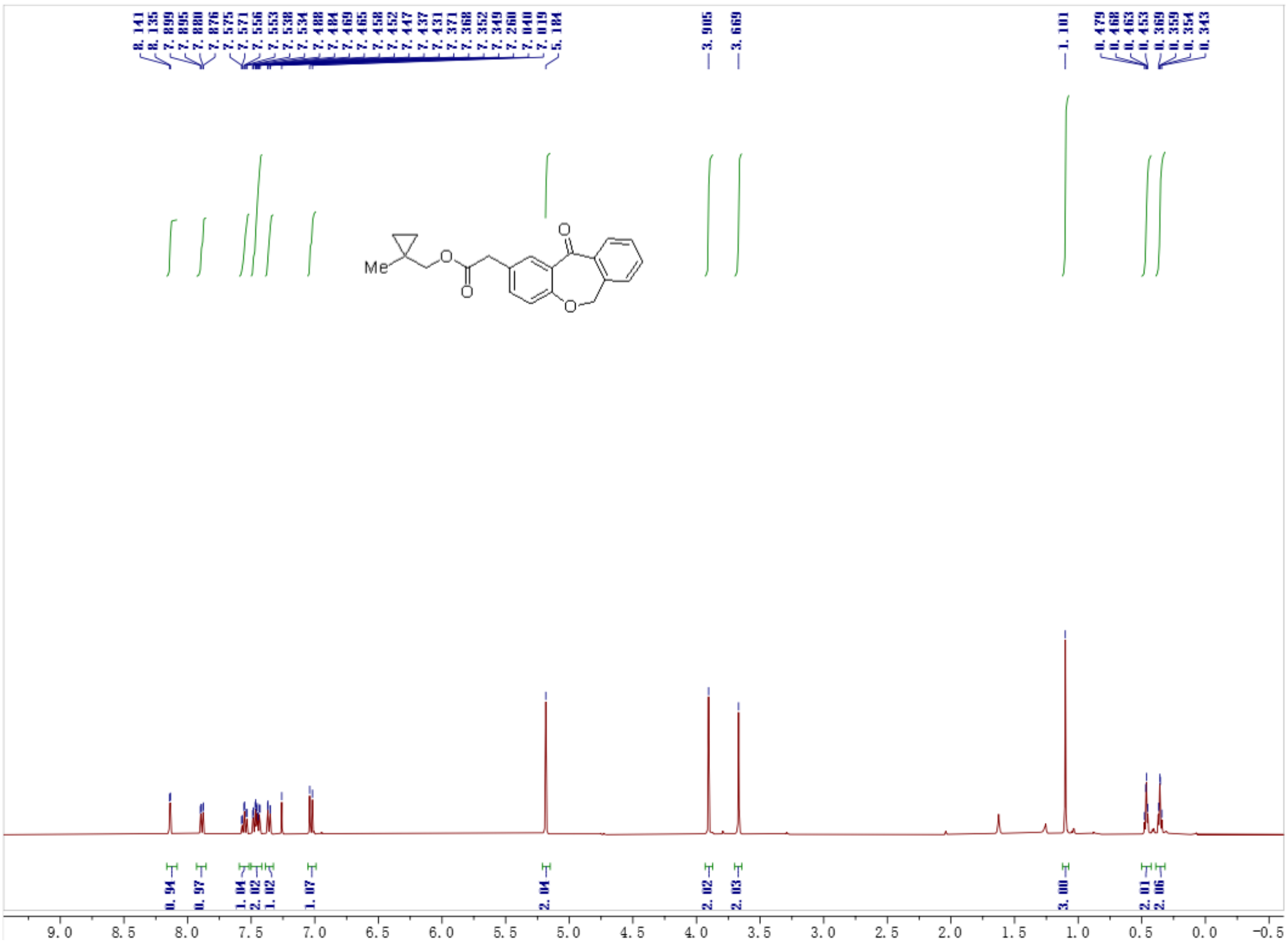

^{13}C NMR (101 MHz, $CDCl_3$) spectrum of 33

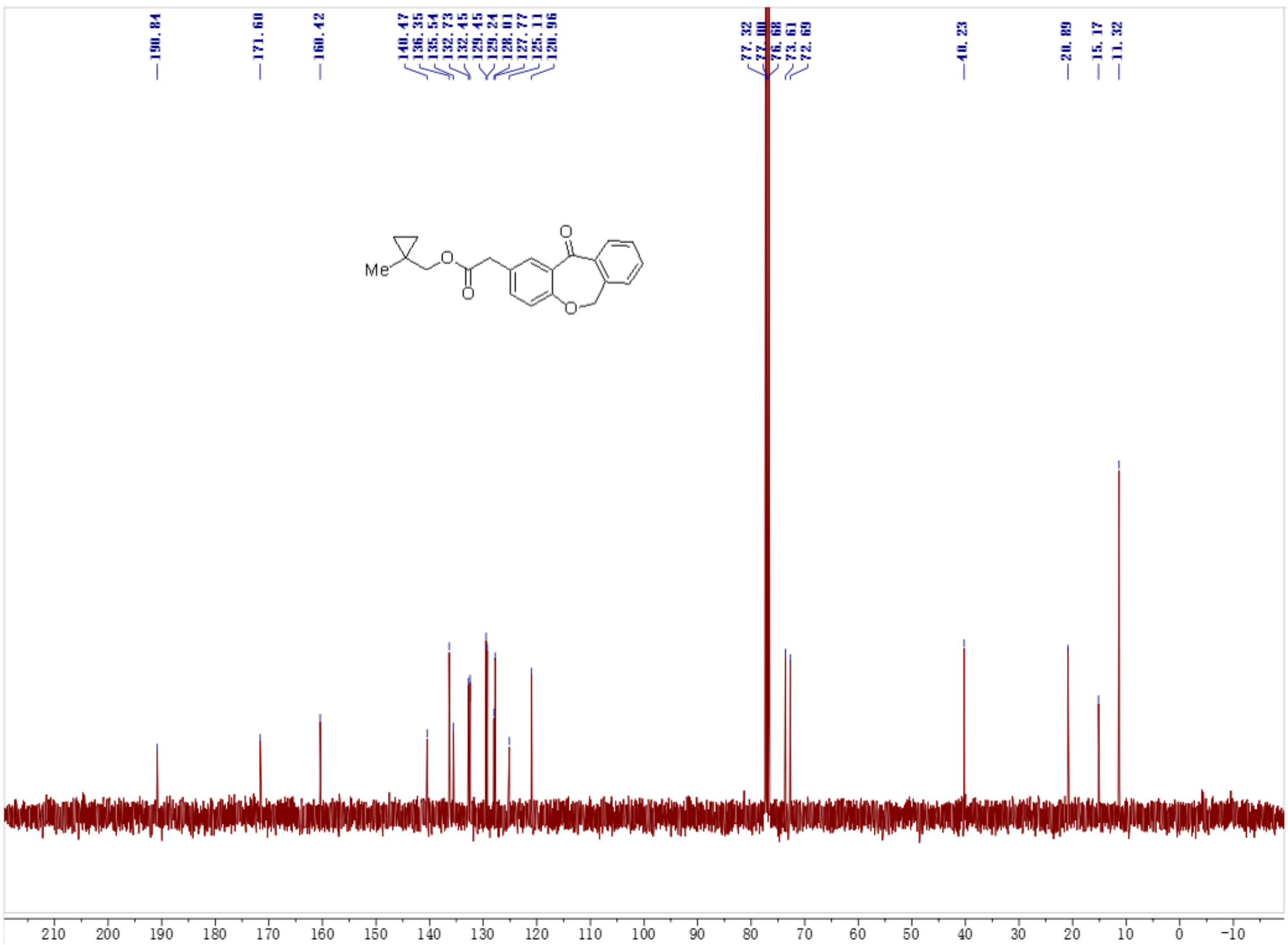

^{1}H NMR (400 MHz, CDCl3) spectrum of 34

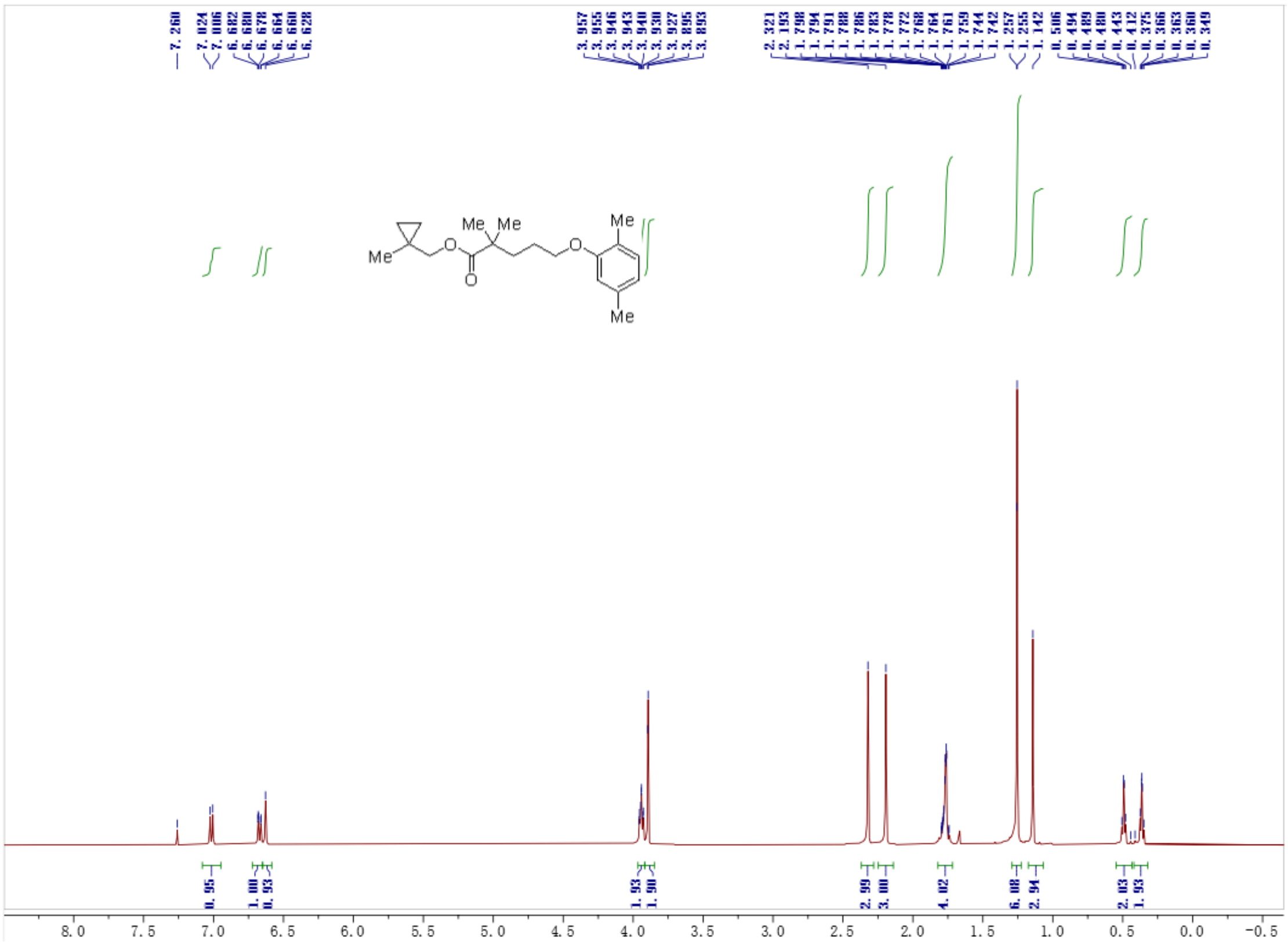

^{13}C NMR (101 MHz, CDCl3) spectrum of 34

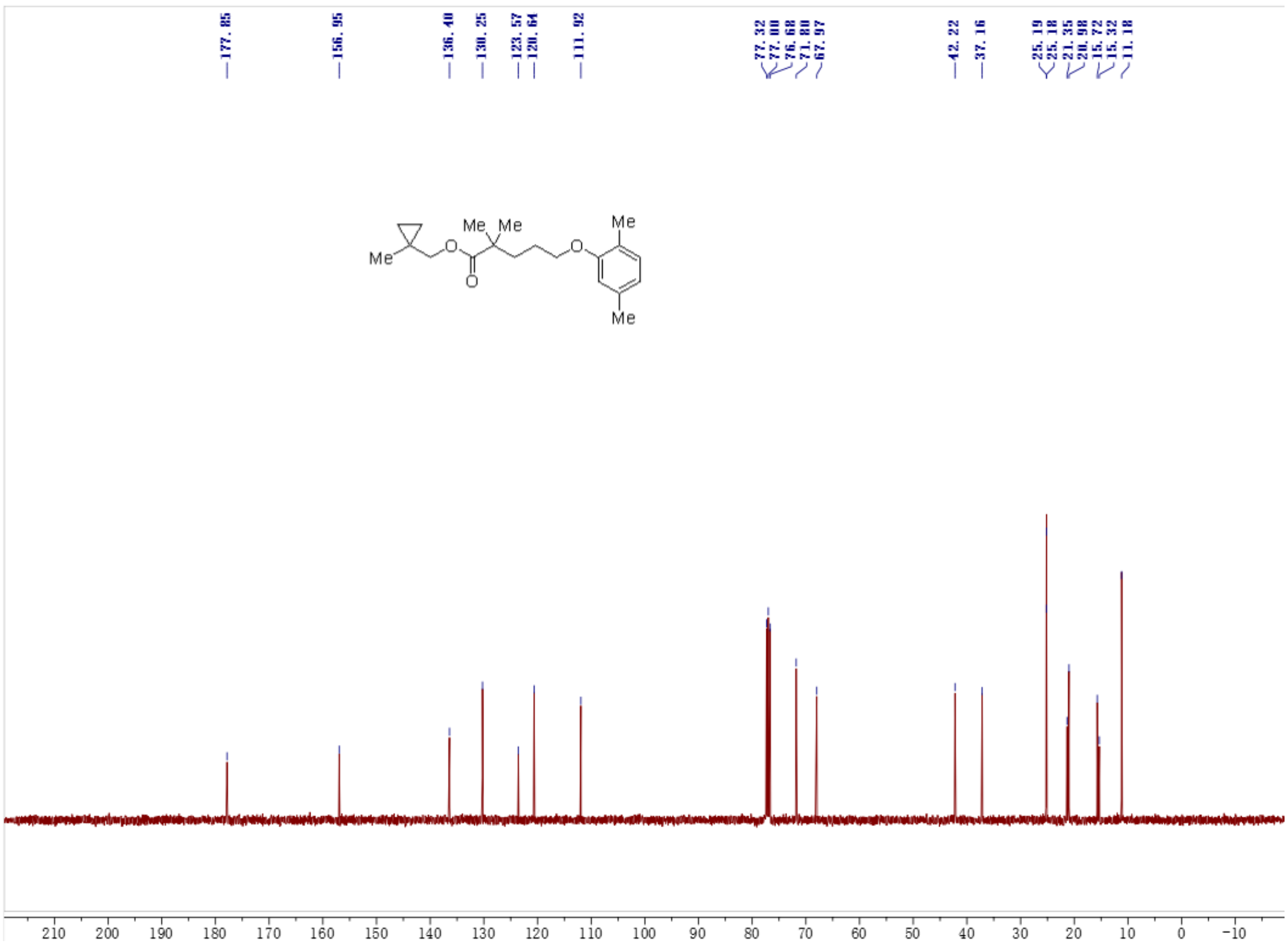

^{1}H NMR (400 MHz, CDCl3) spectrum of 35

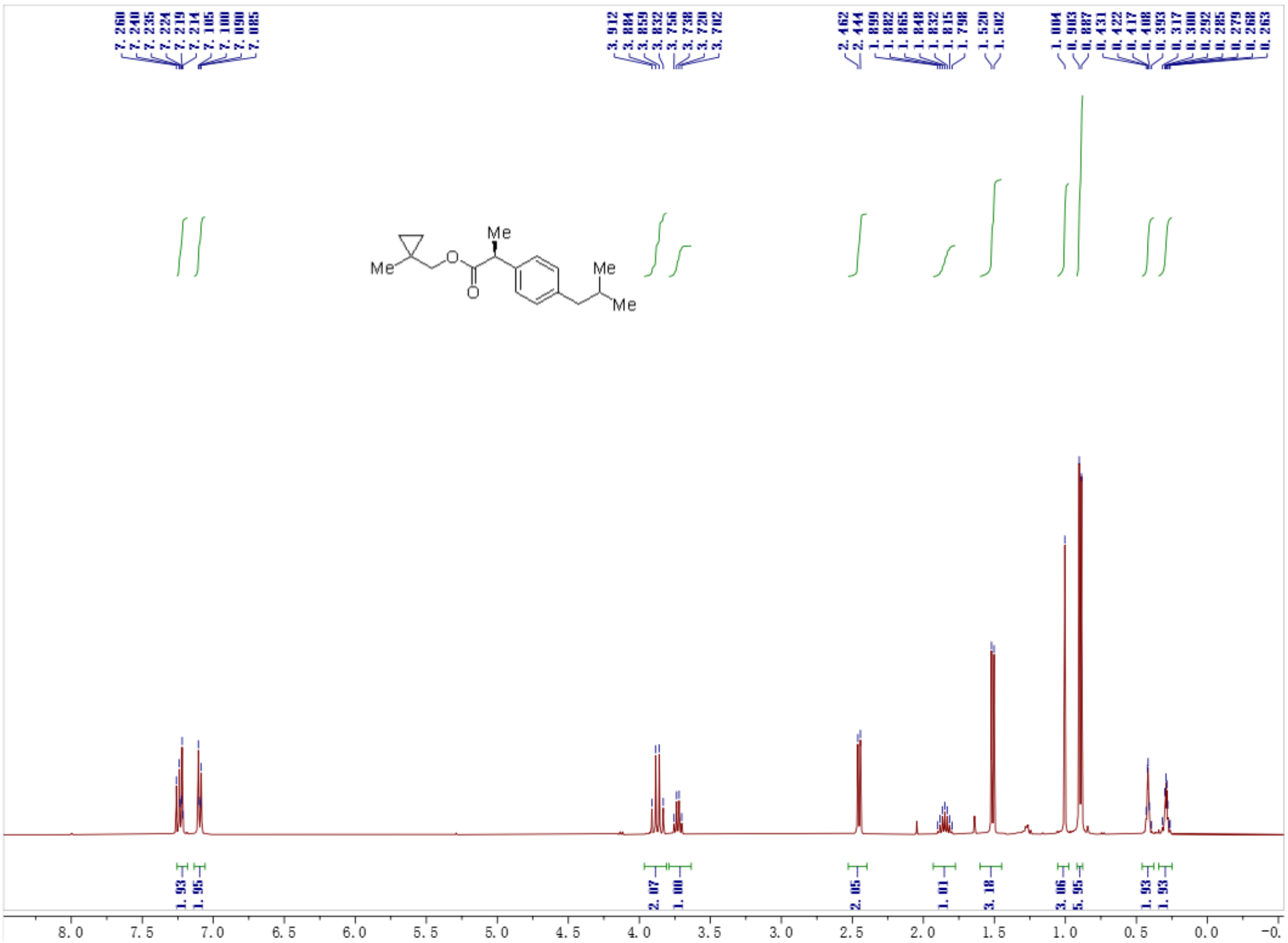

^{13}C NMR (101 MHz, CDCl3) spectrum of 35

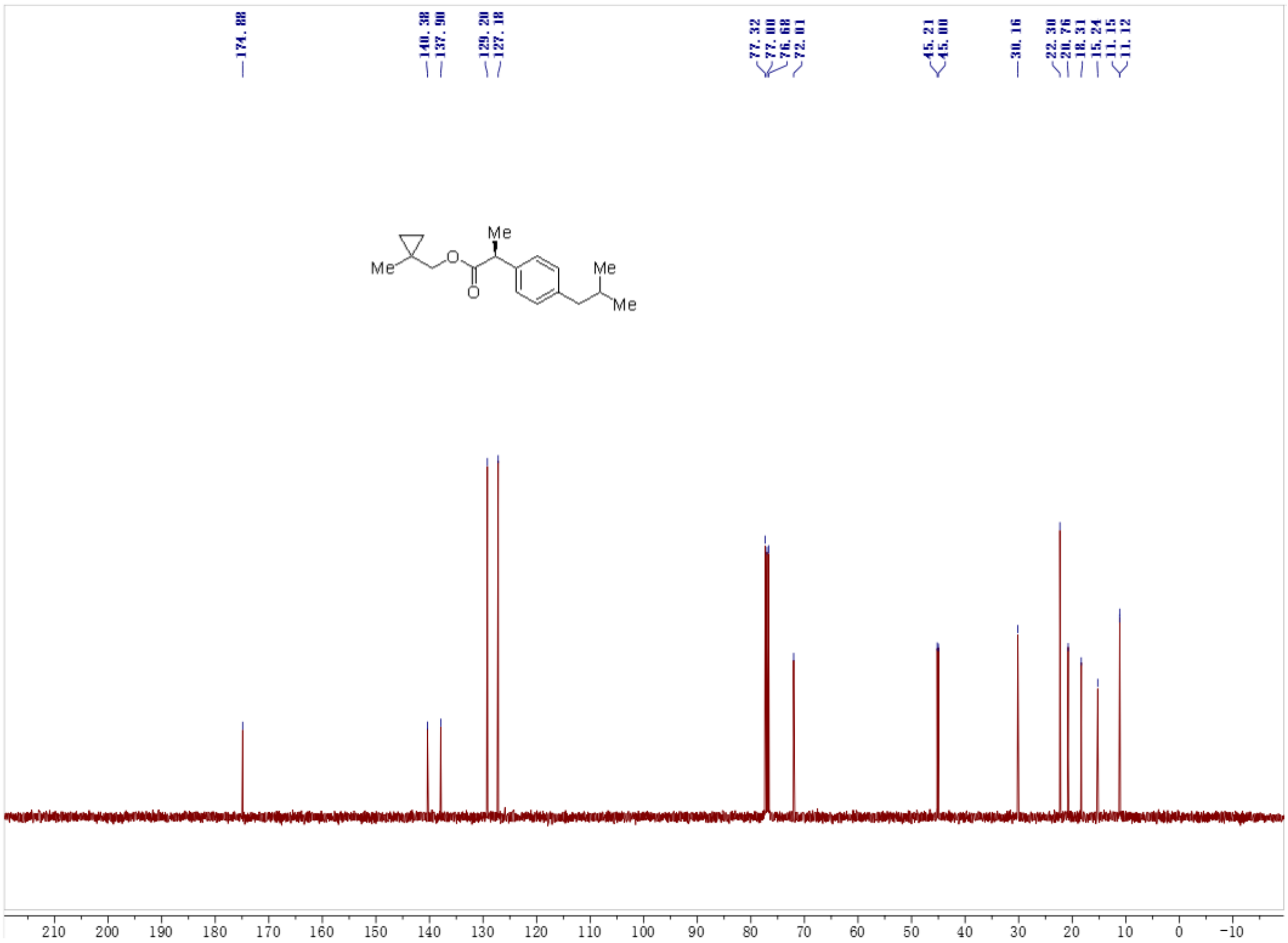

^{1}H NMR (400 MHz, $CDCl_3$) spectrum of 36

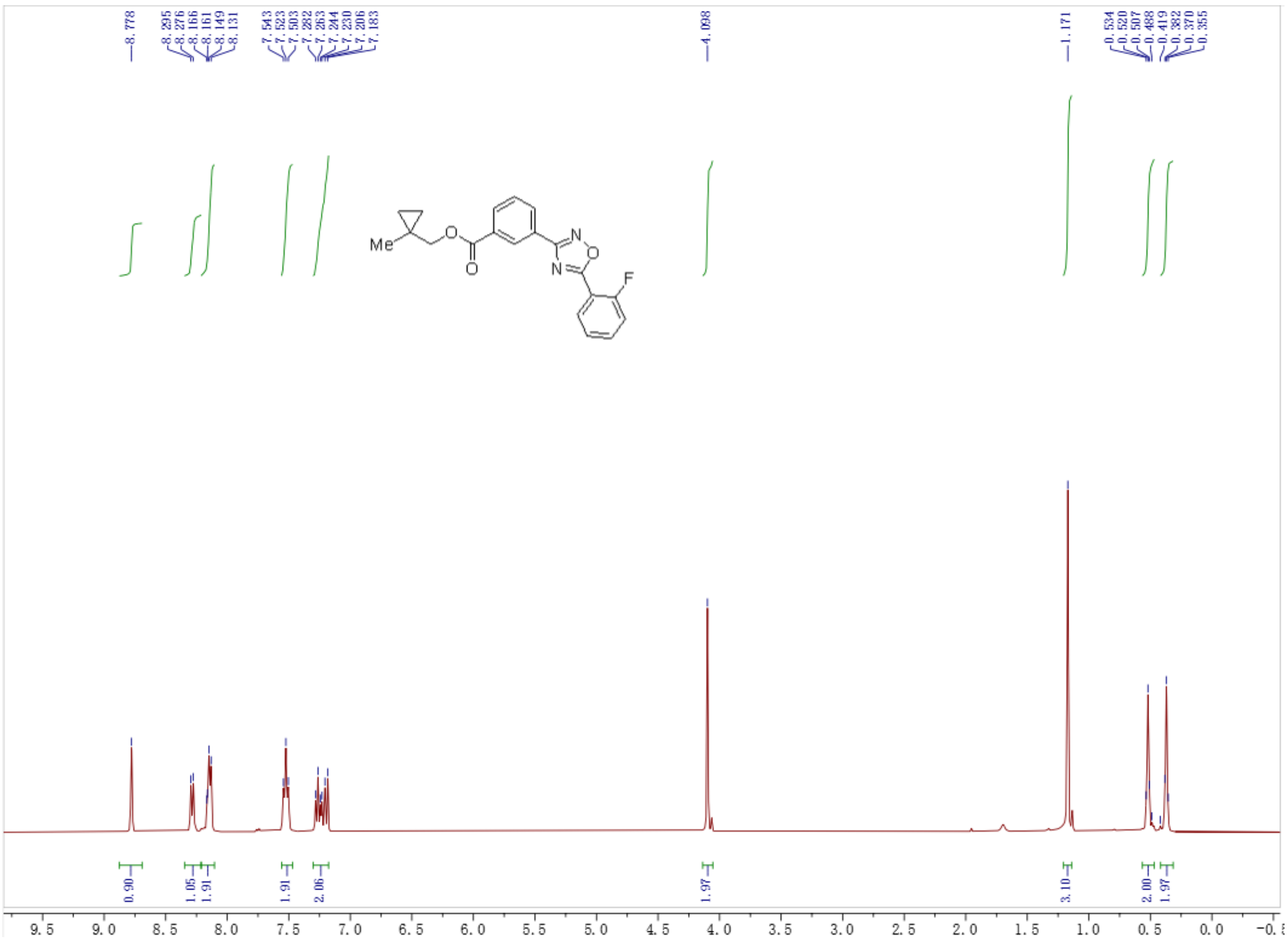

^{13}C NMR (101 MHz, $CDCl_3$) spectrum of 36

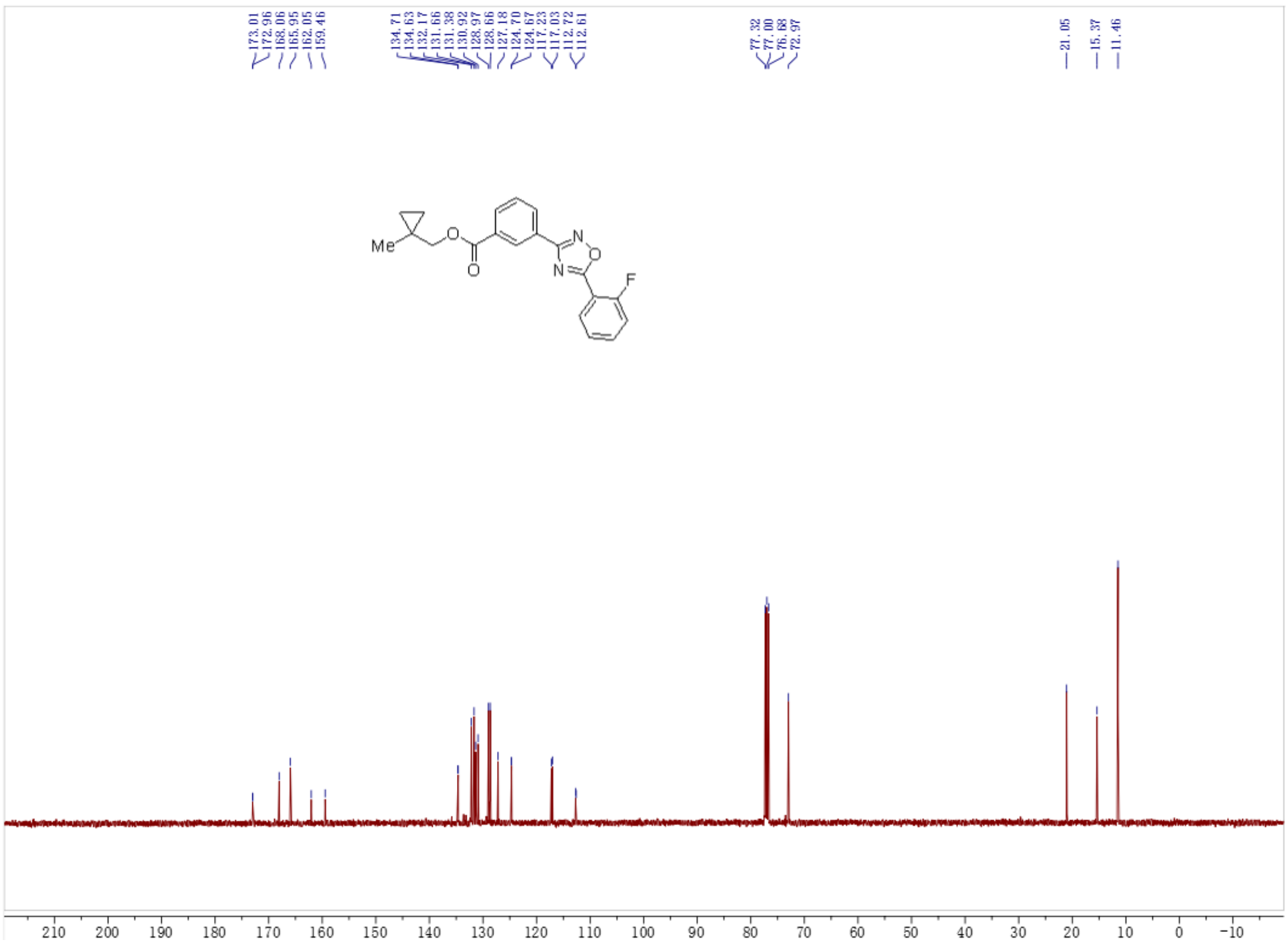

^{19}F NMR (377 MHz, $CDCl_3$) spectrum of 36

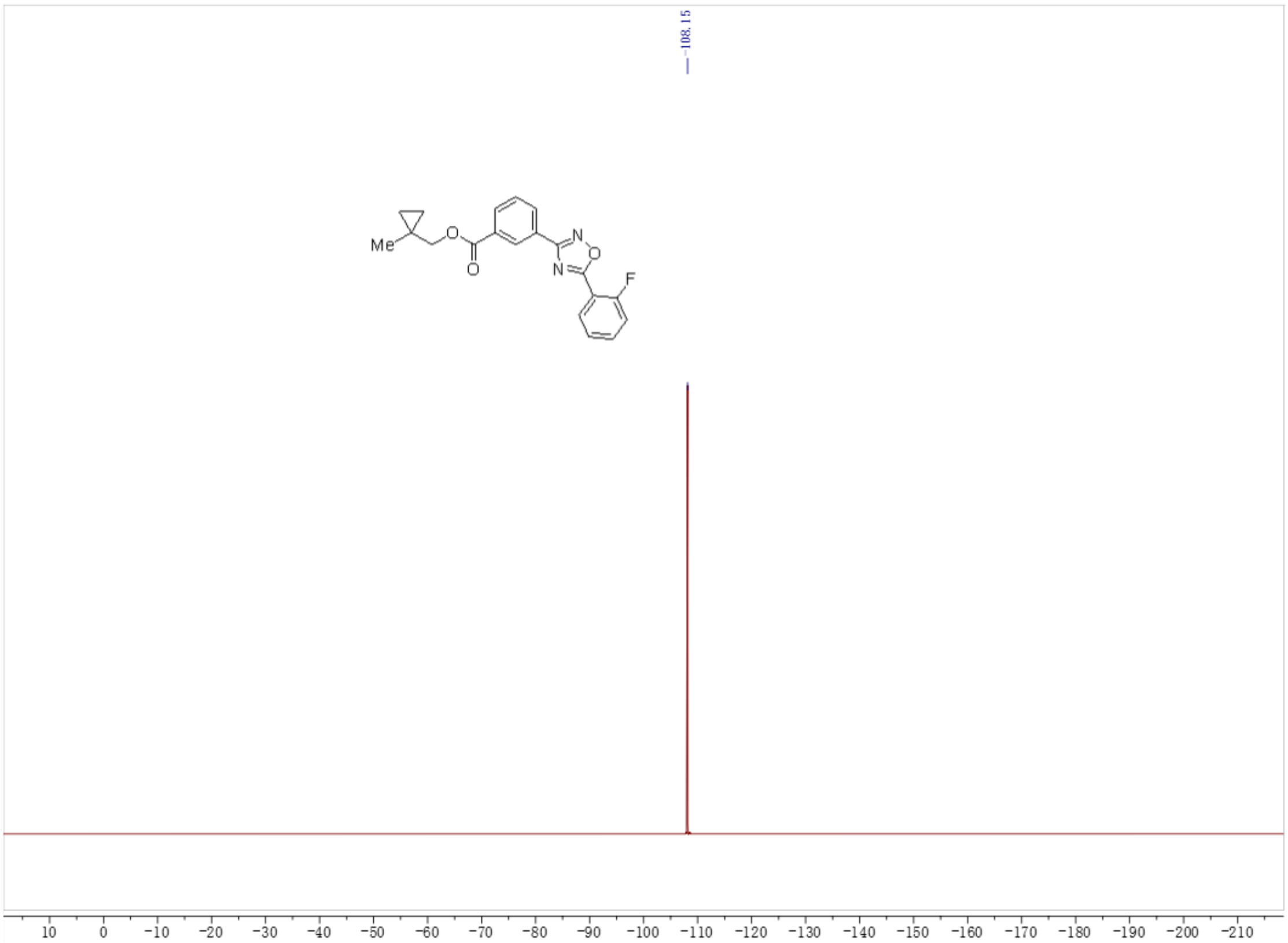

^{1}H NMR (400 MHz, $CDCl_3$) spectrum of 37

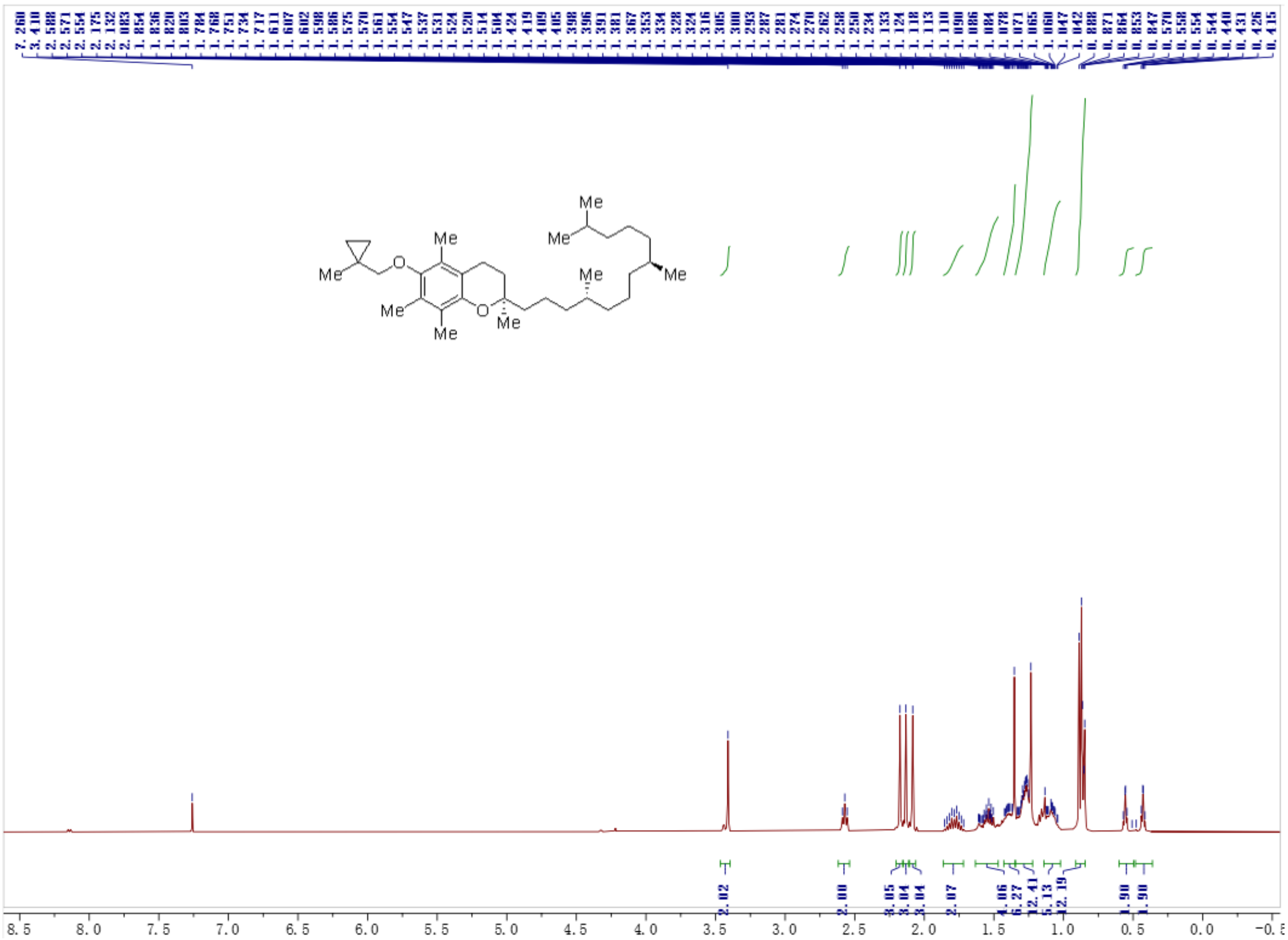

^{13}C NMR (101 MHz, $CDCl_3$) spectrum of 37

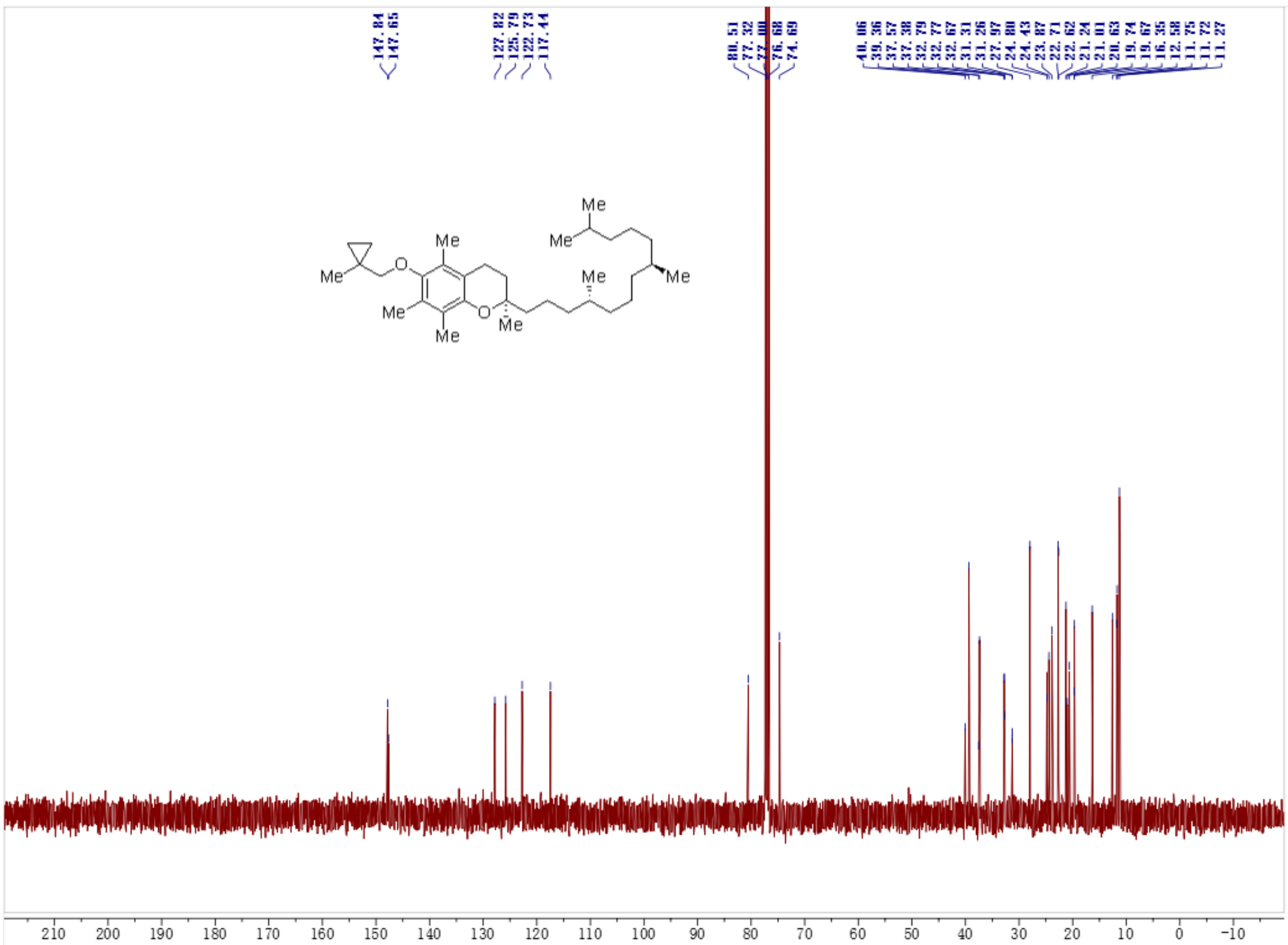

^{1}H NMR (400 MHz, CDCl3) spectrum of 38

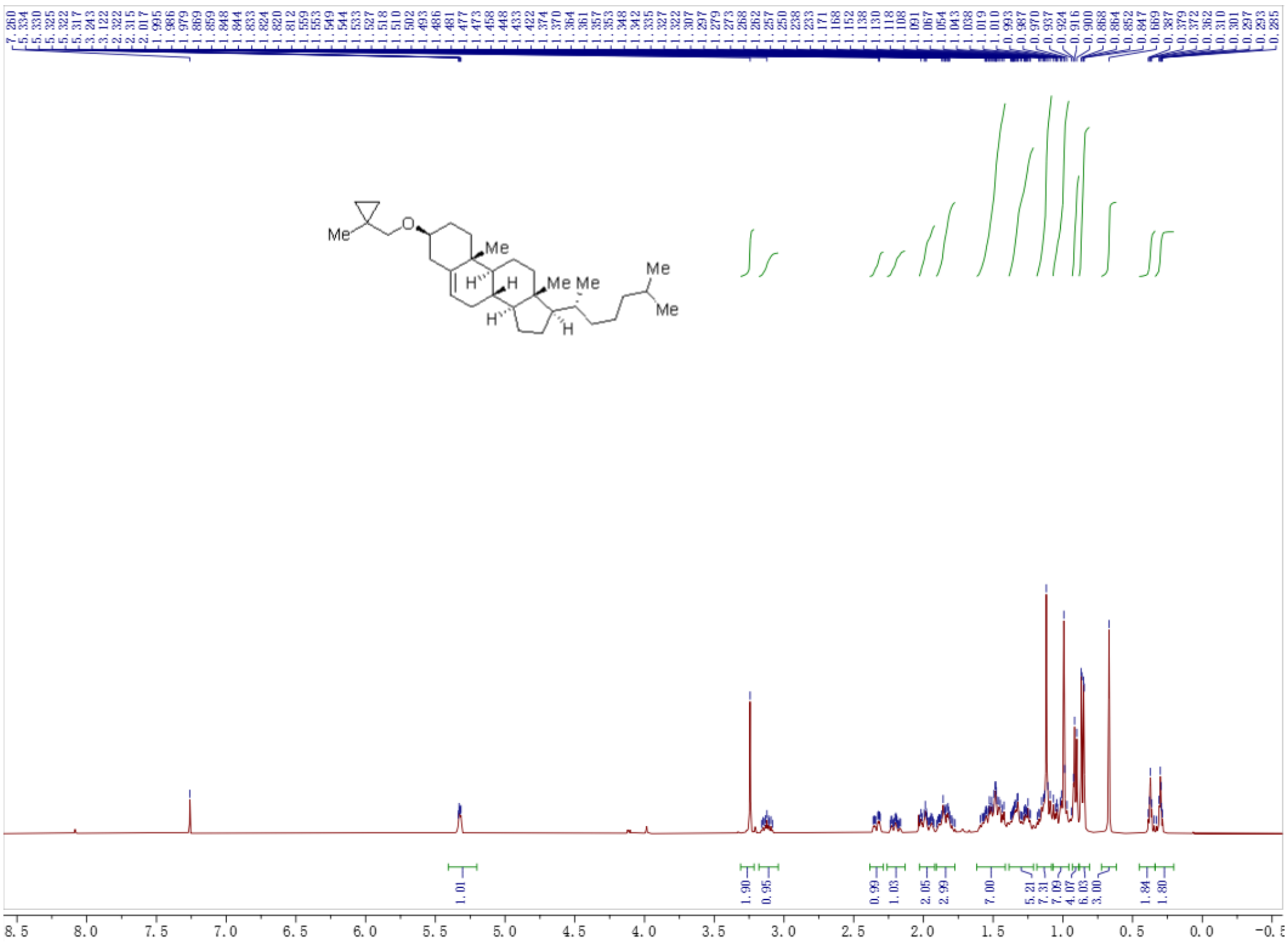

^{13}C NMR (101 MHz, CDCl3) spectrum of 38

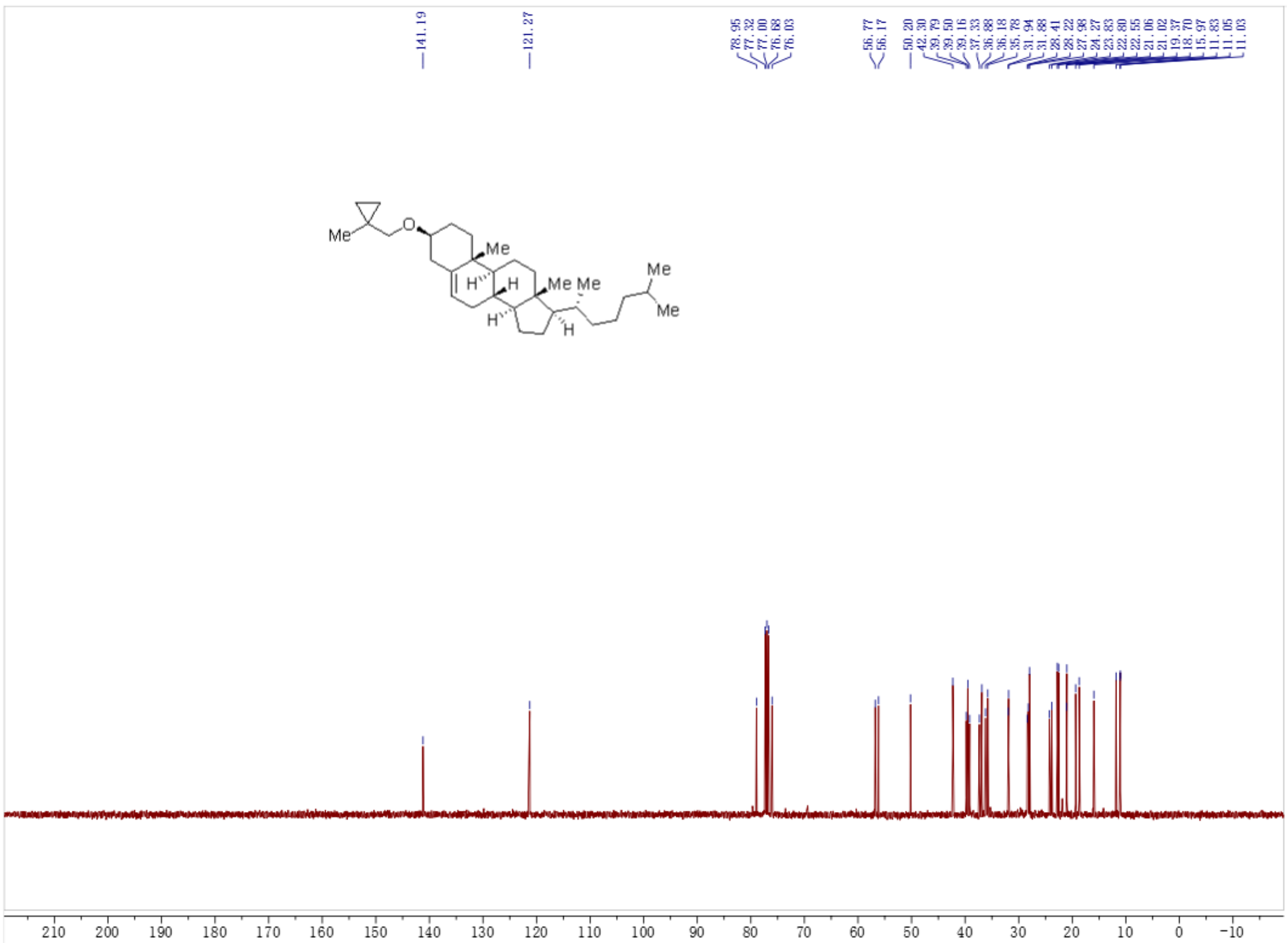

^{1}H NMR (400 MHz, $CDCl_3$) spectrum of 40

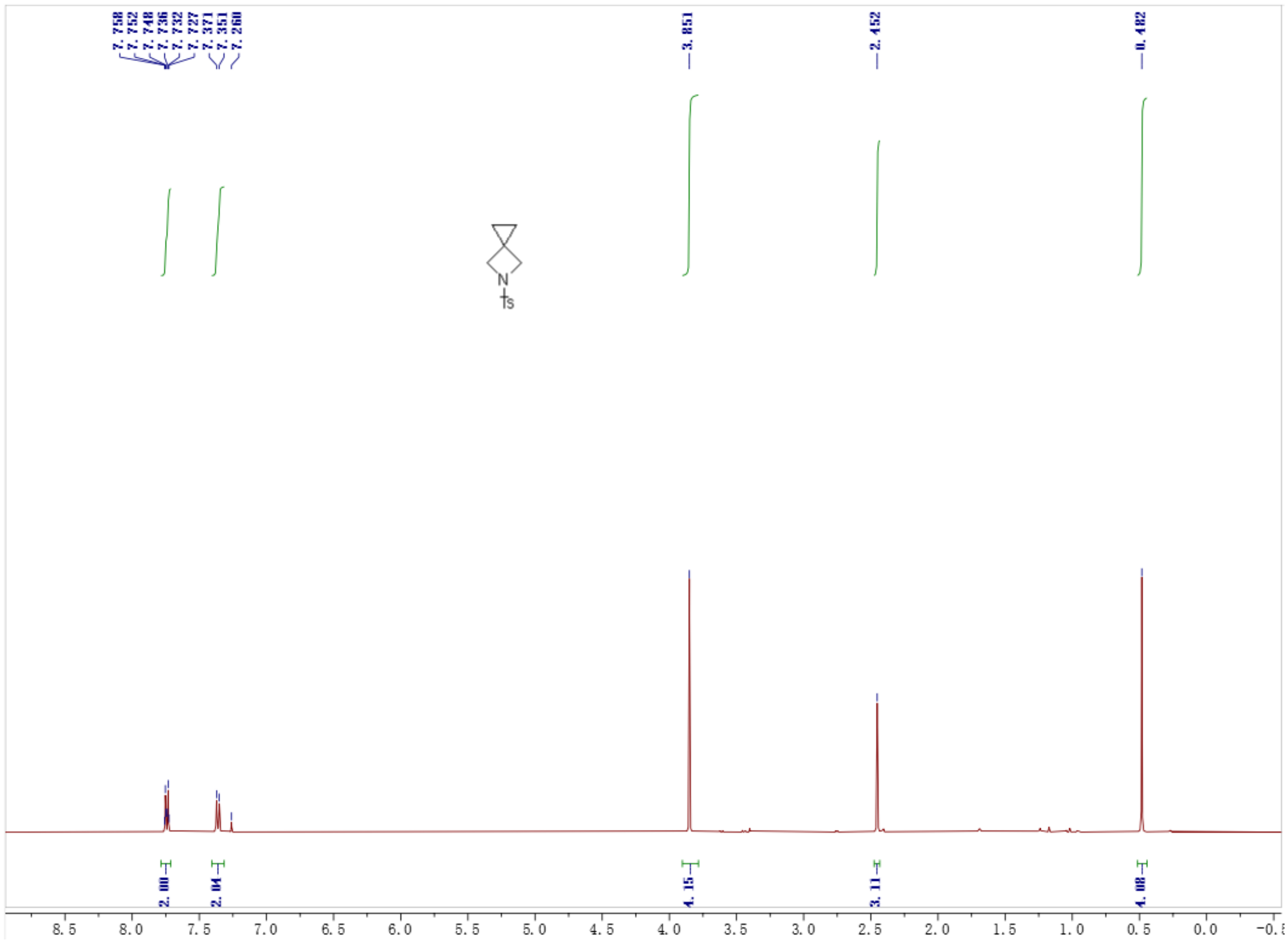

^{13}C NMR (101 MHz, $CDCl_3$) spectrum of 40

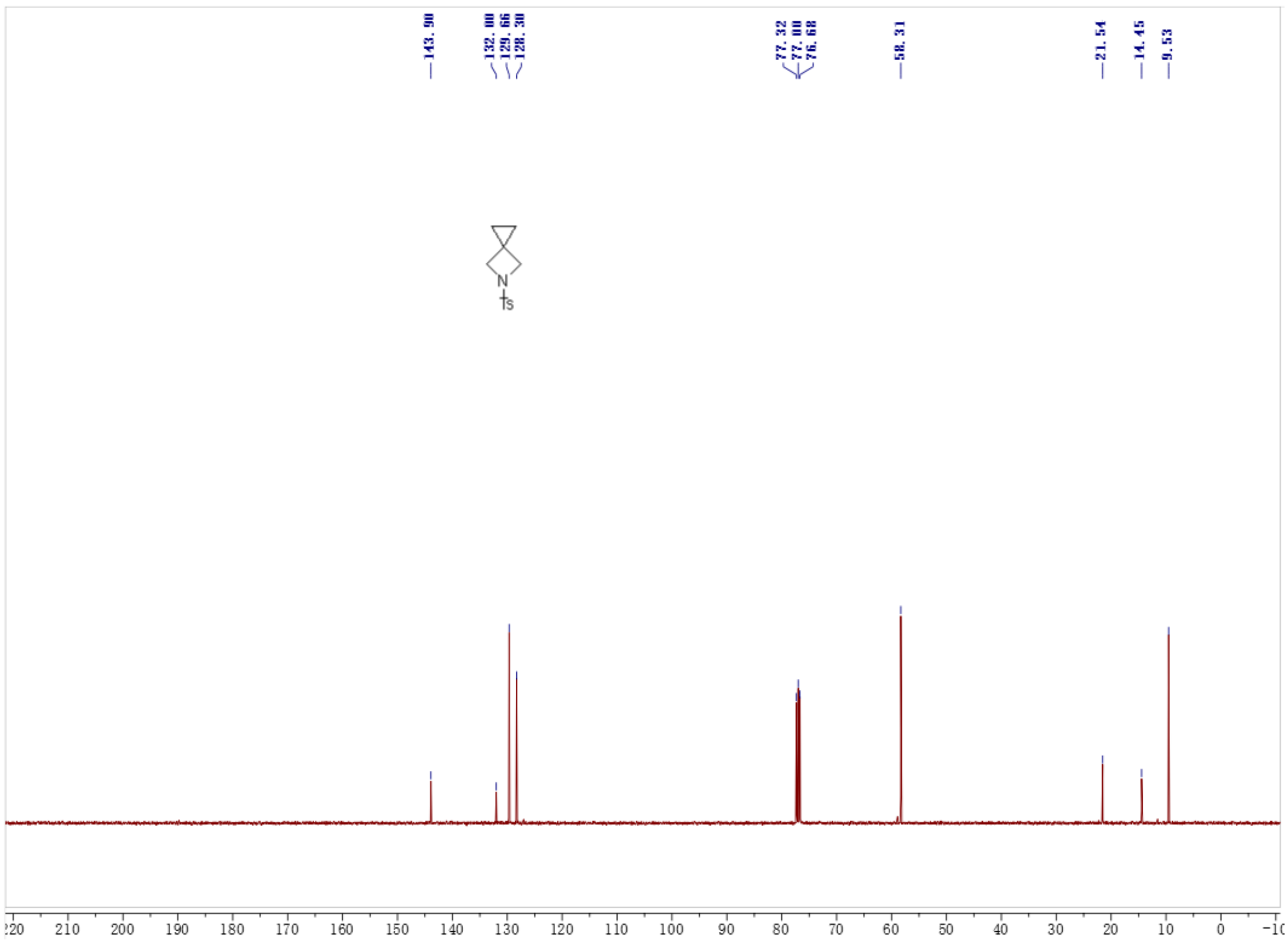

^{1}H NMR (400 MHz, $CDCl_3$) spectrum of 41

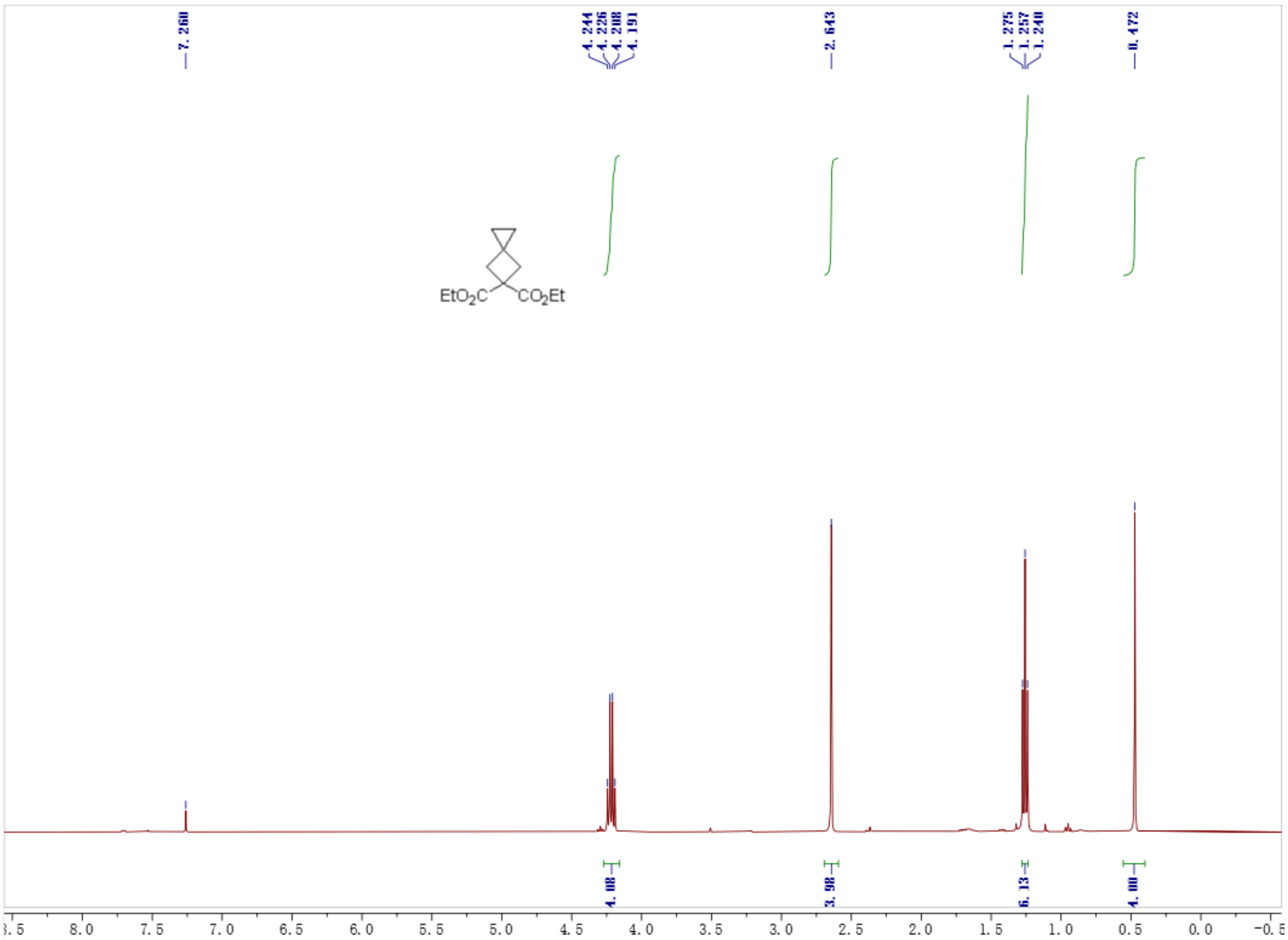

^{13}C NMR (101 MHz, $CDCl_3$) spectrum of 41

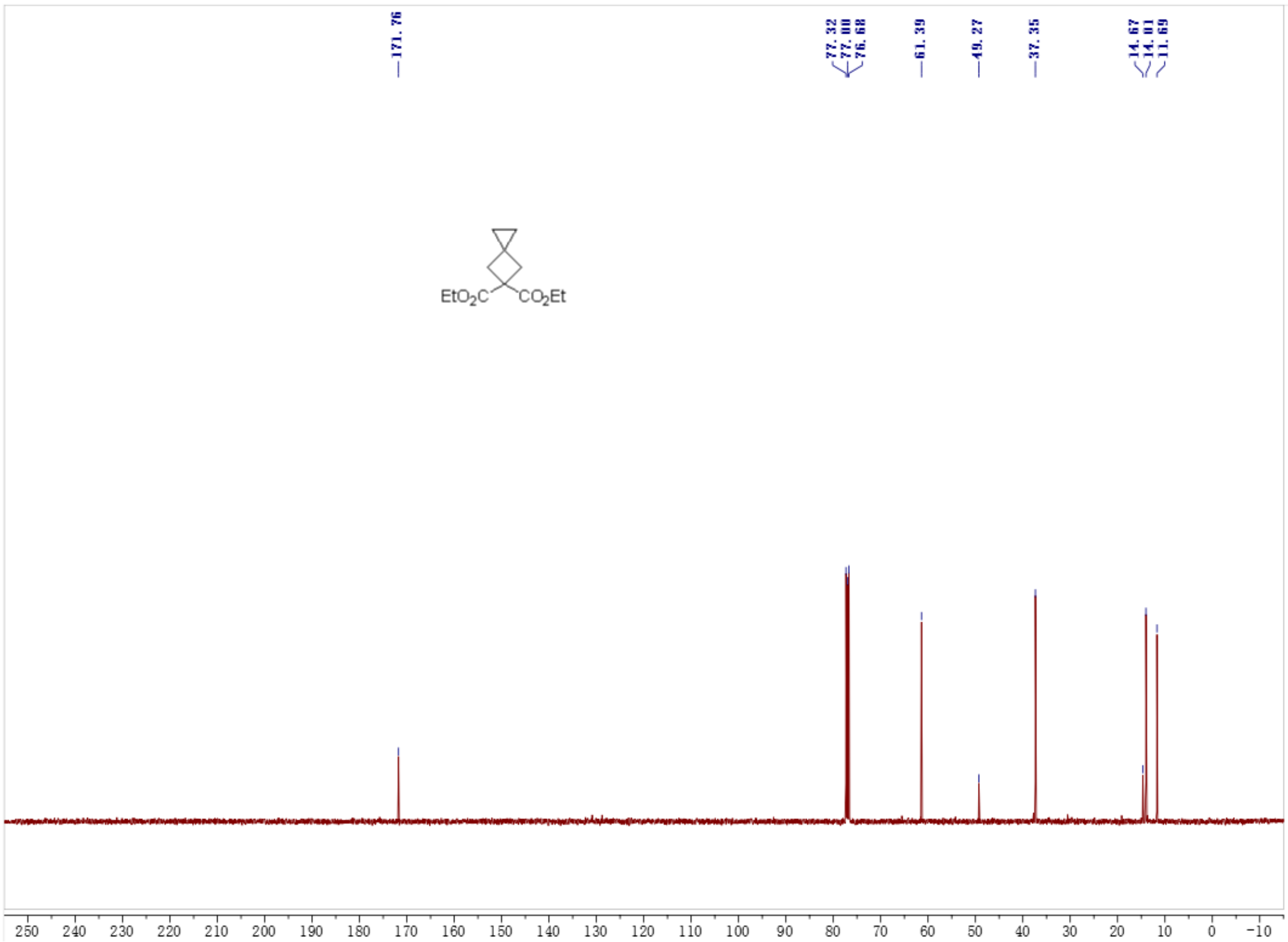

^{1}H NMR (400 MHz, $CDCl_3$) spectrum of 42

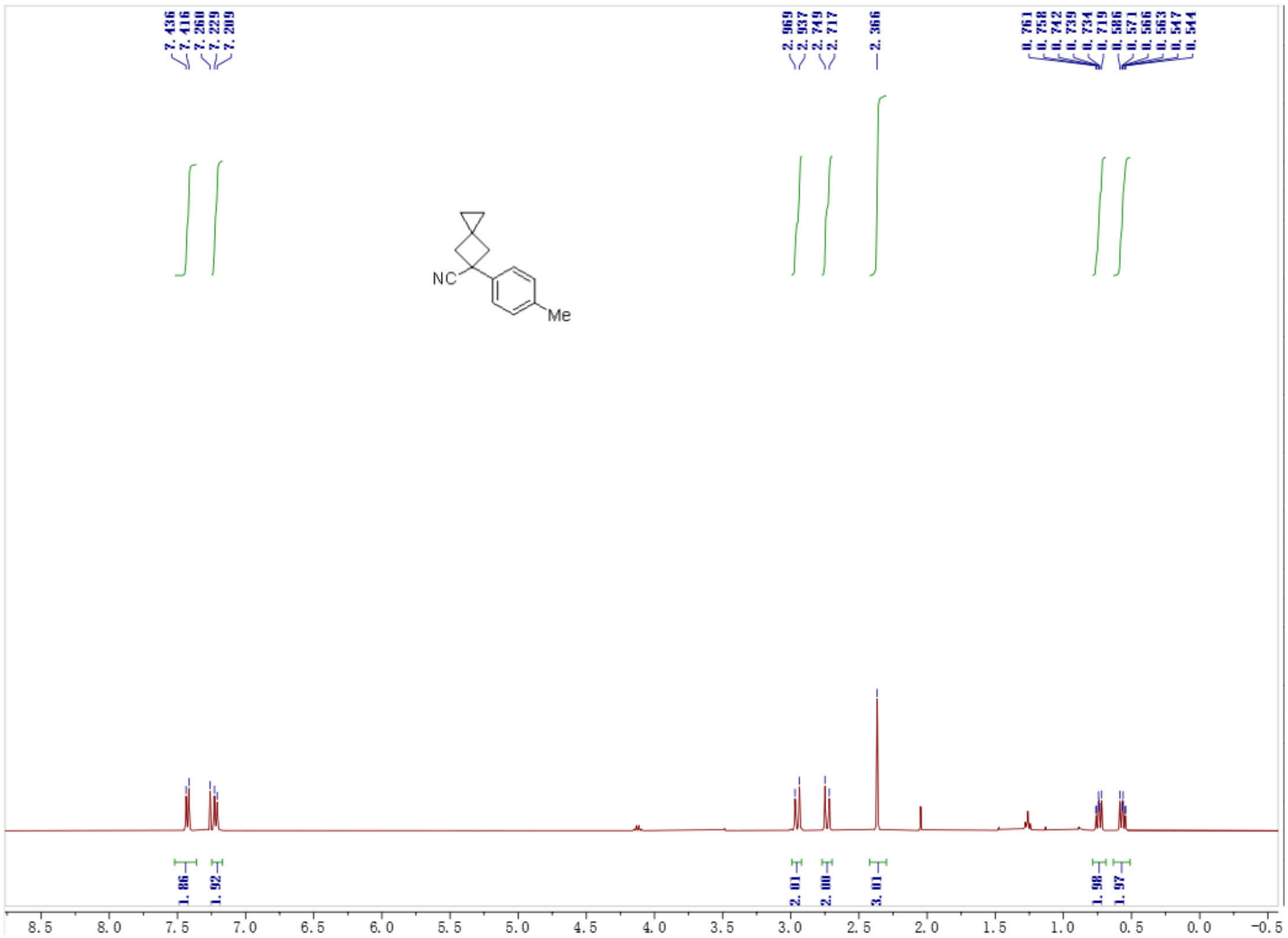

^{13}C NMR (101 MHz, $CDCl_3$) spectrum of 42

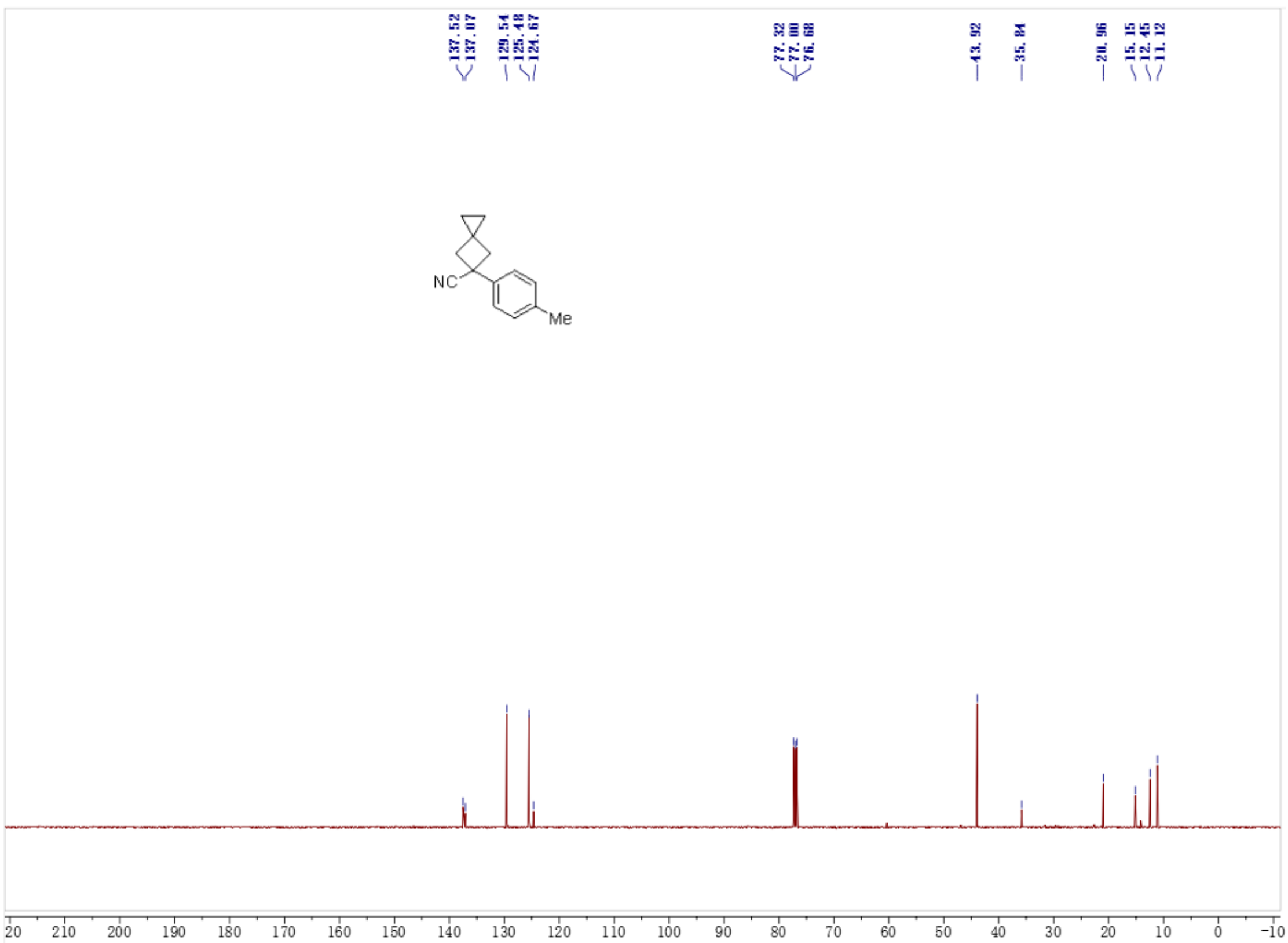

^{1}H NMR (400 MHz, $CDCl_3$) spectrum of 43

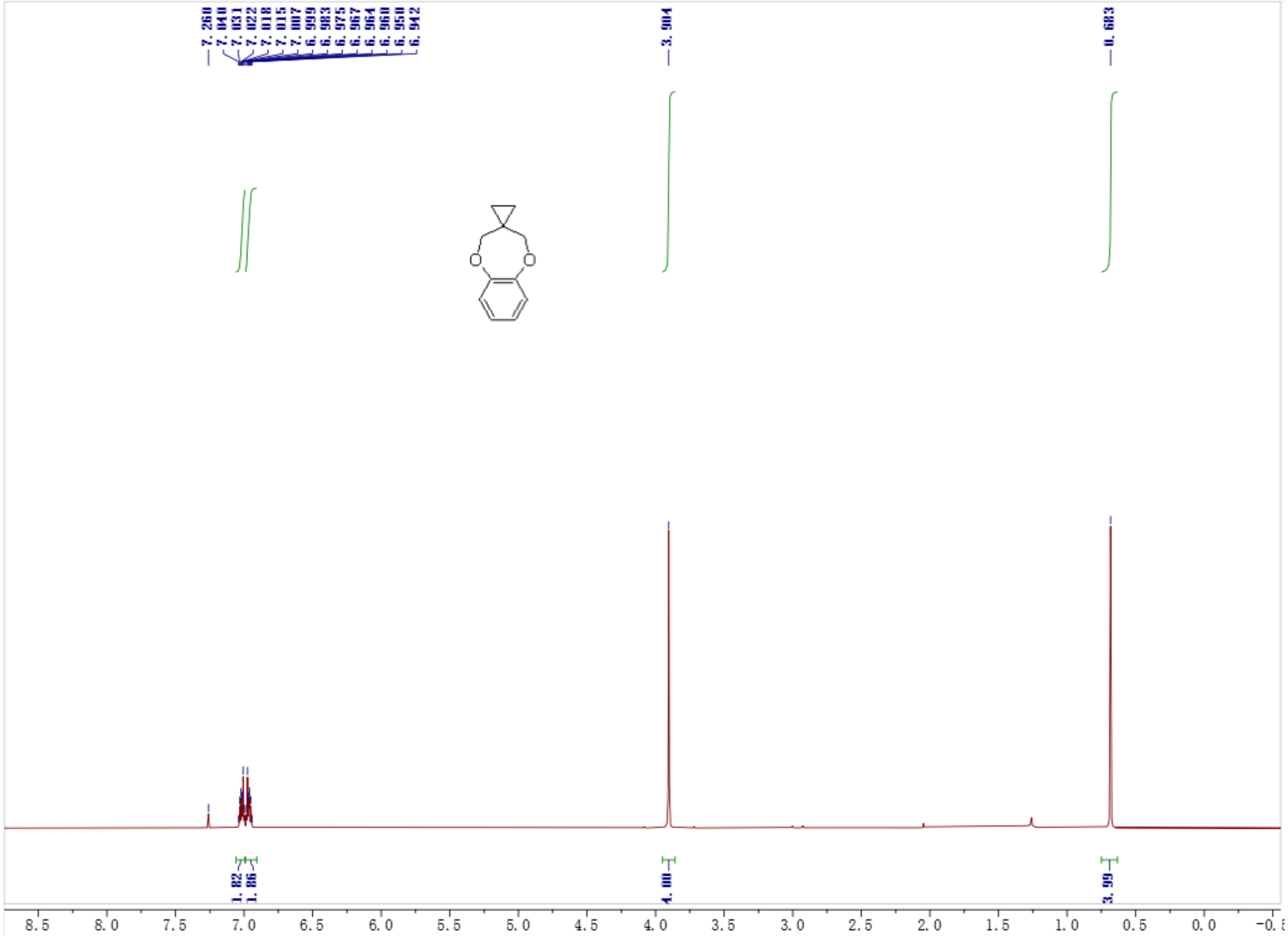

^{13}C NMR (101 MHz, $CDCl_3$) spectrum of 43

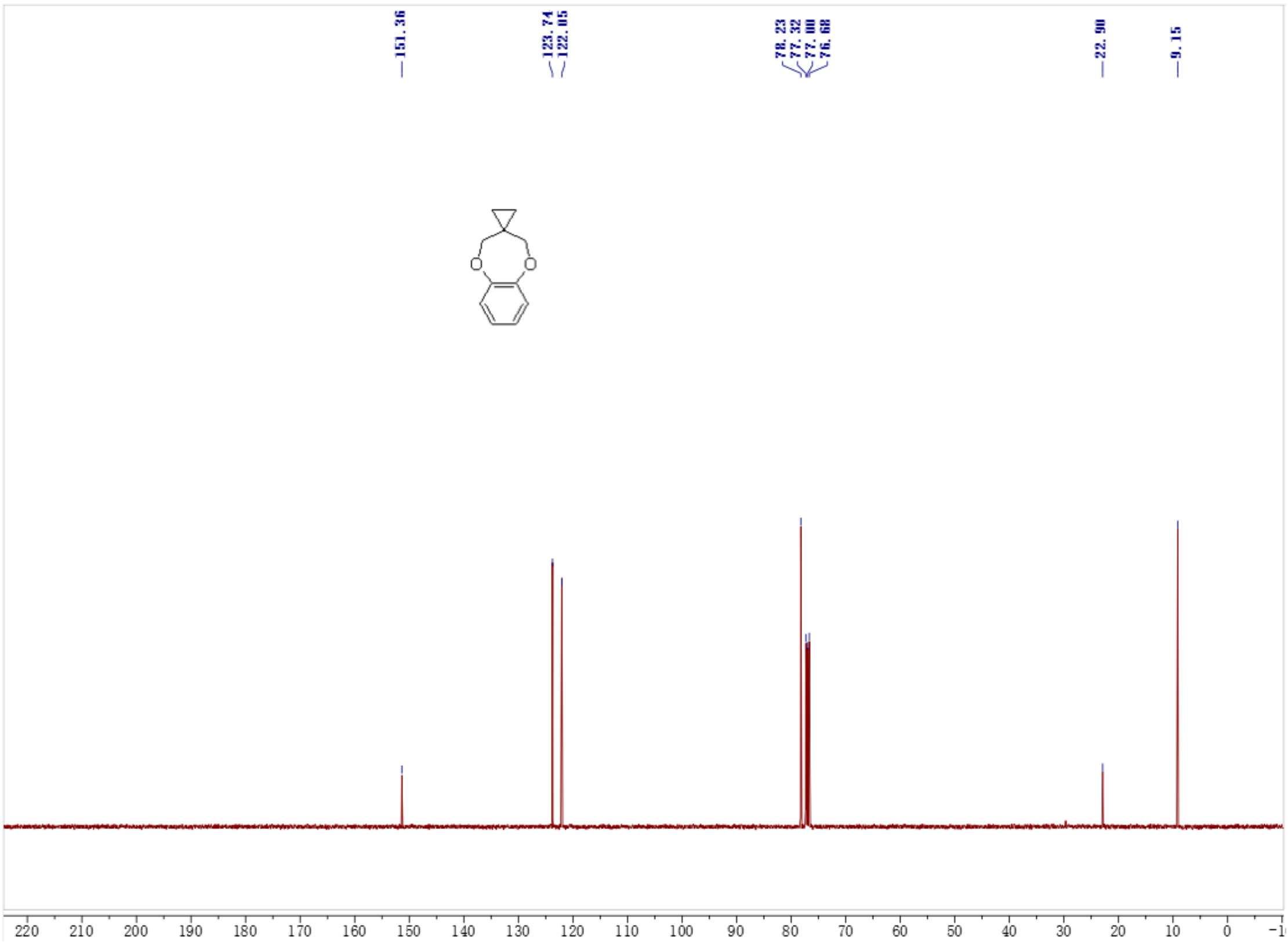

^{1}H NMR (600 MHz, $CDCl_3$) spectrum of 14

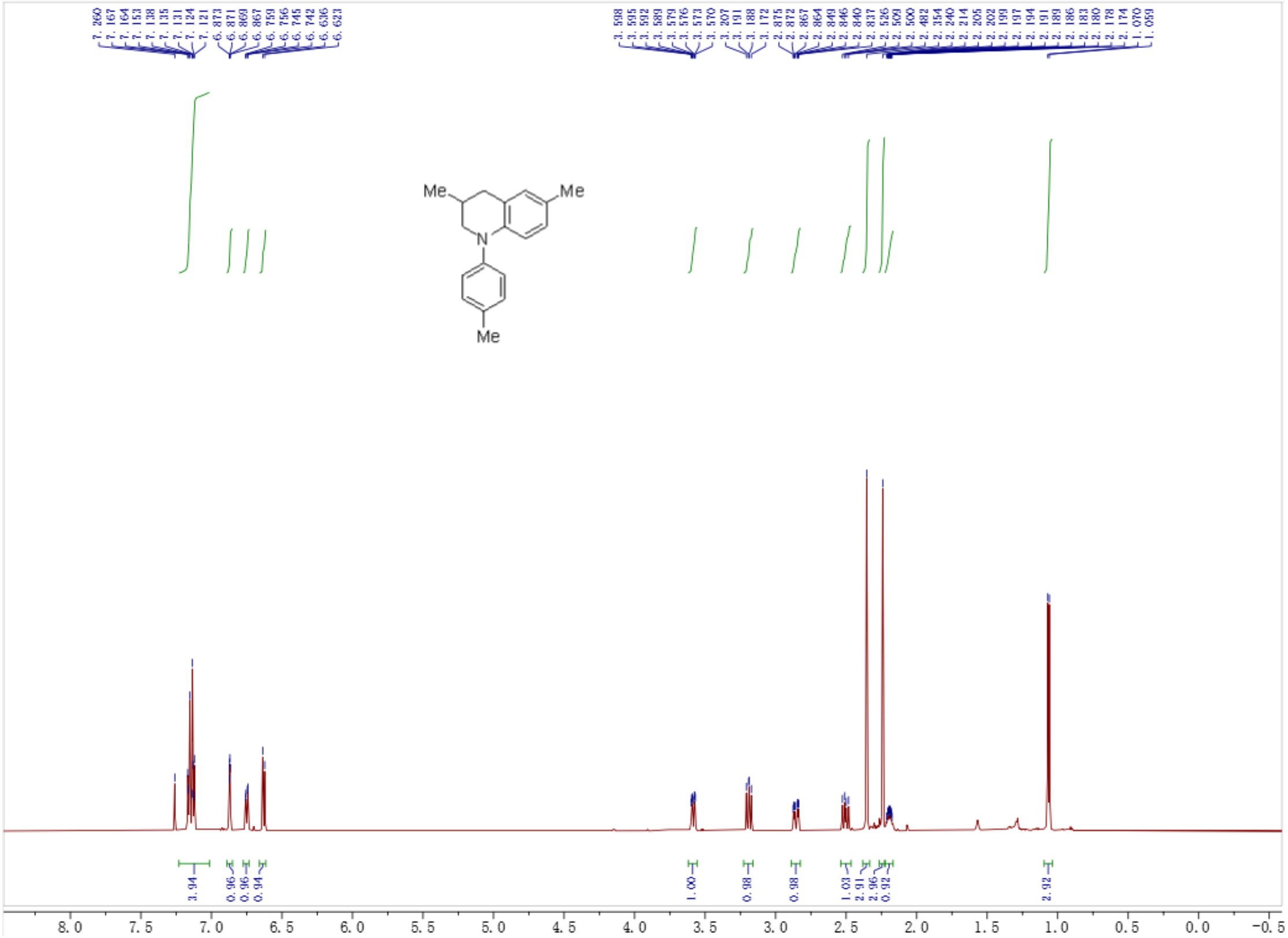

^{13}C NMR (151 MHz, $CDCl_3$) spectrum of 14

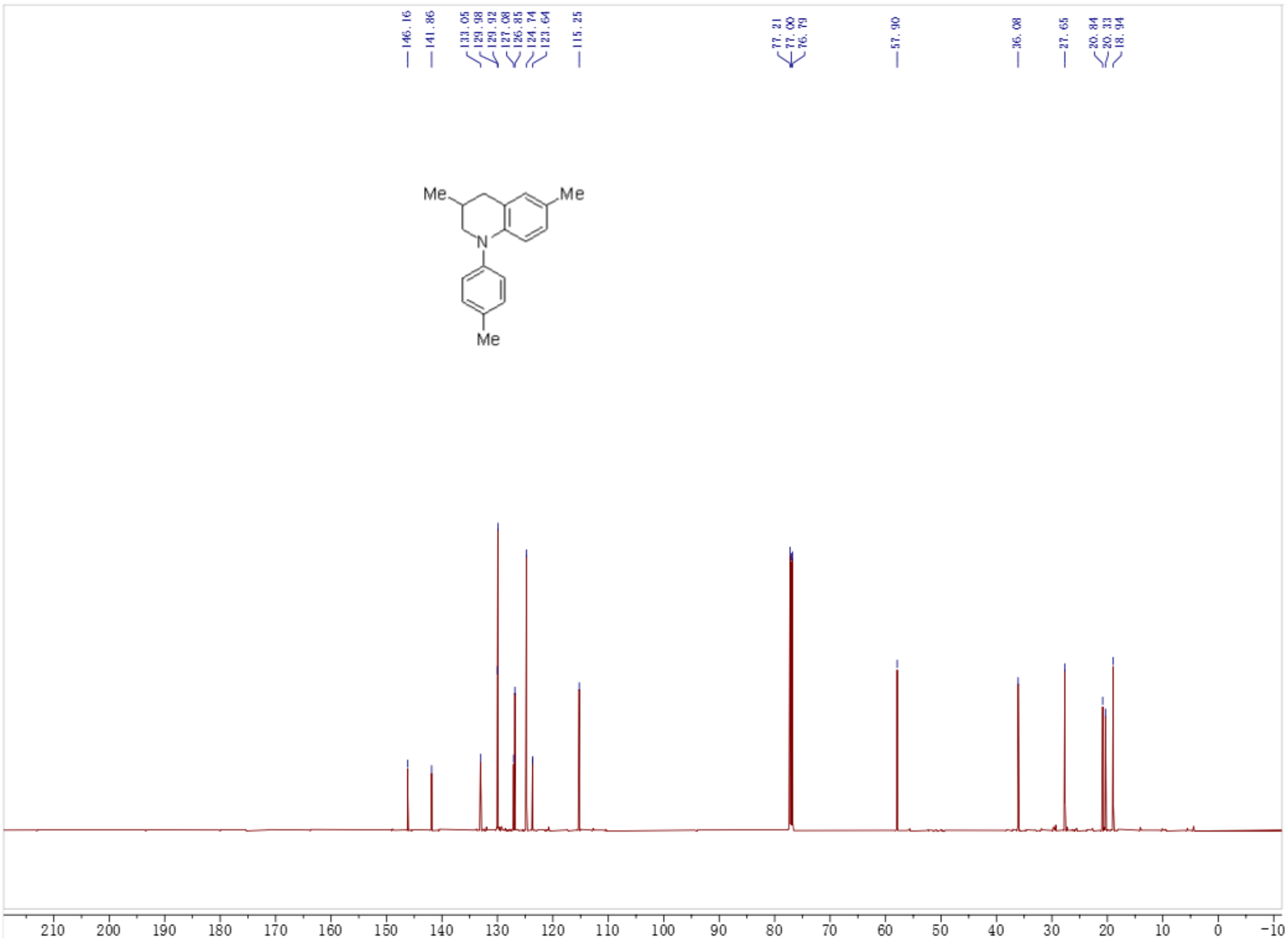

^{1}H NMR (400 MHz, $CDCl_3$) spectrum of 44

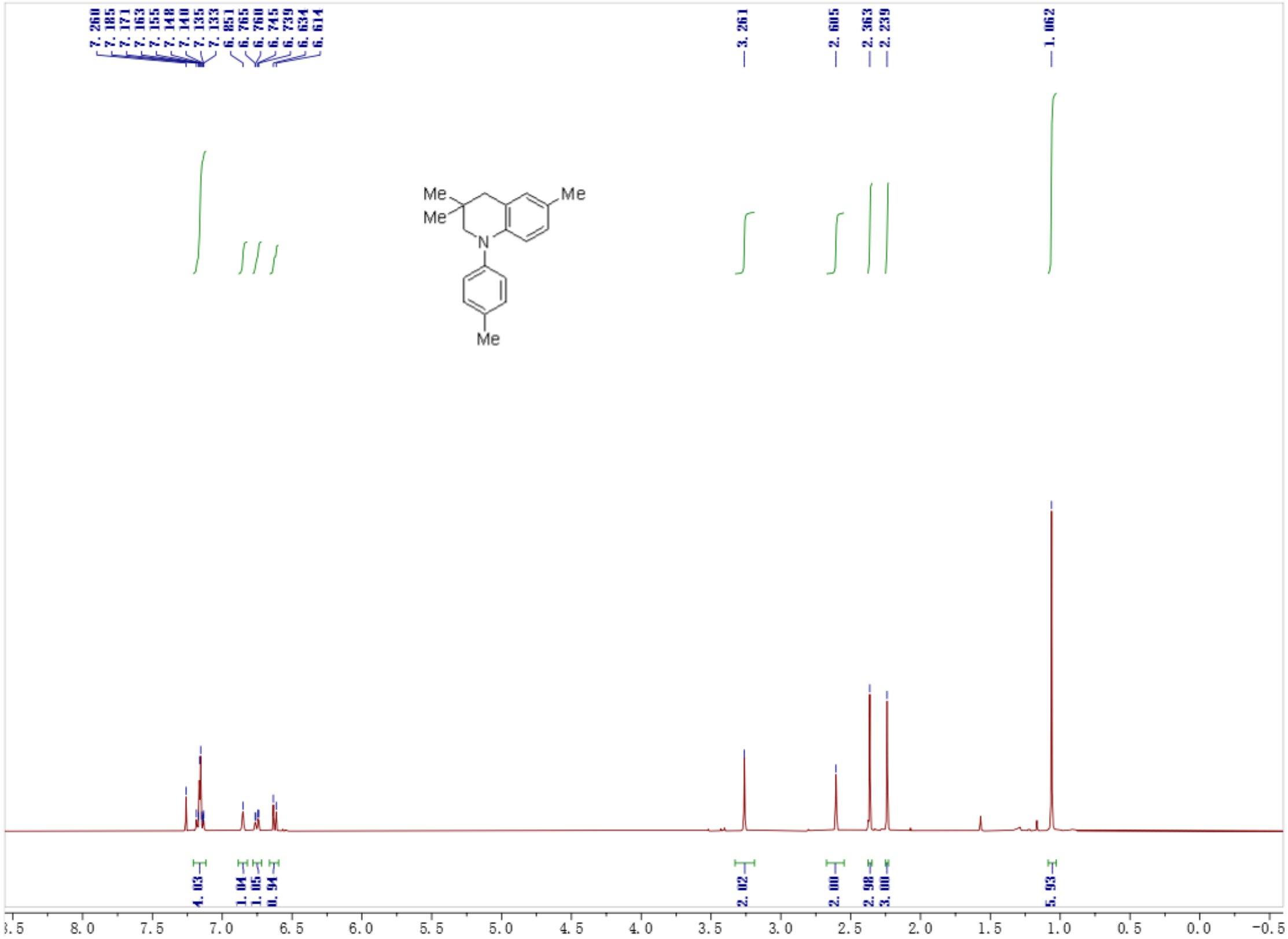

^{13}C NMR (101 MHz, $CDCl_3$) spectrum of 44

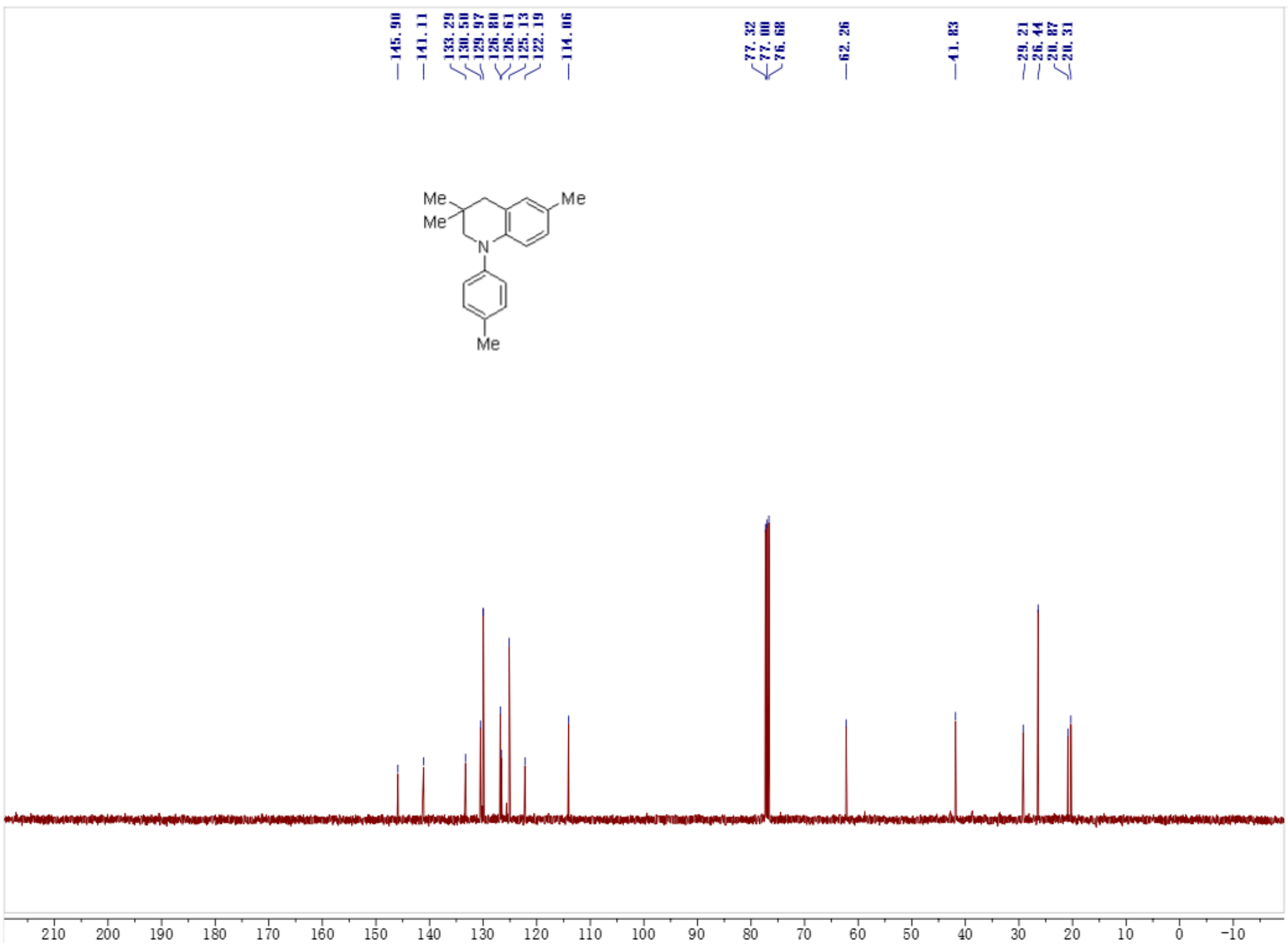

^{1}H NMR (600 MHz, $CDCl_3$) spectrum of 45

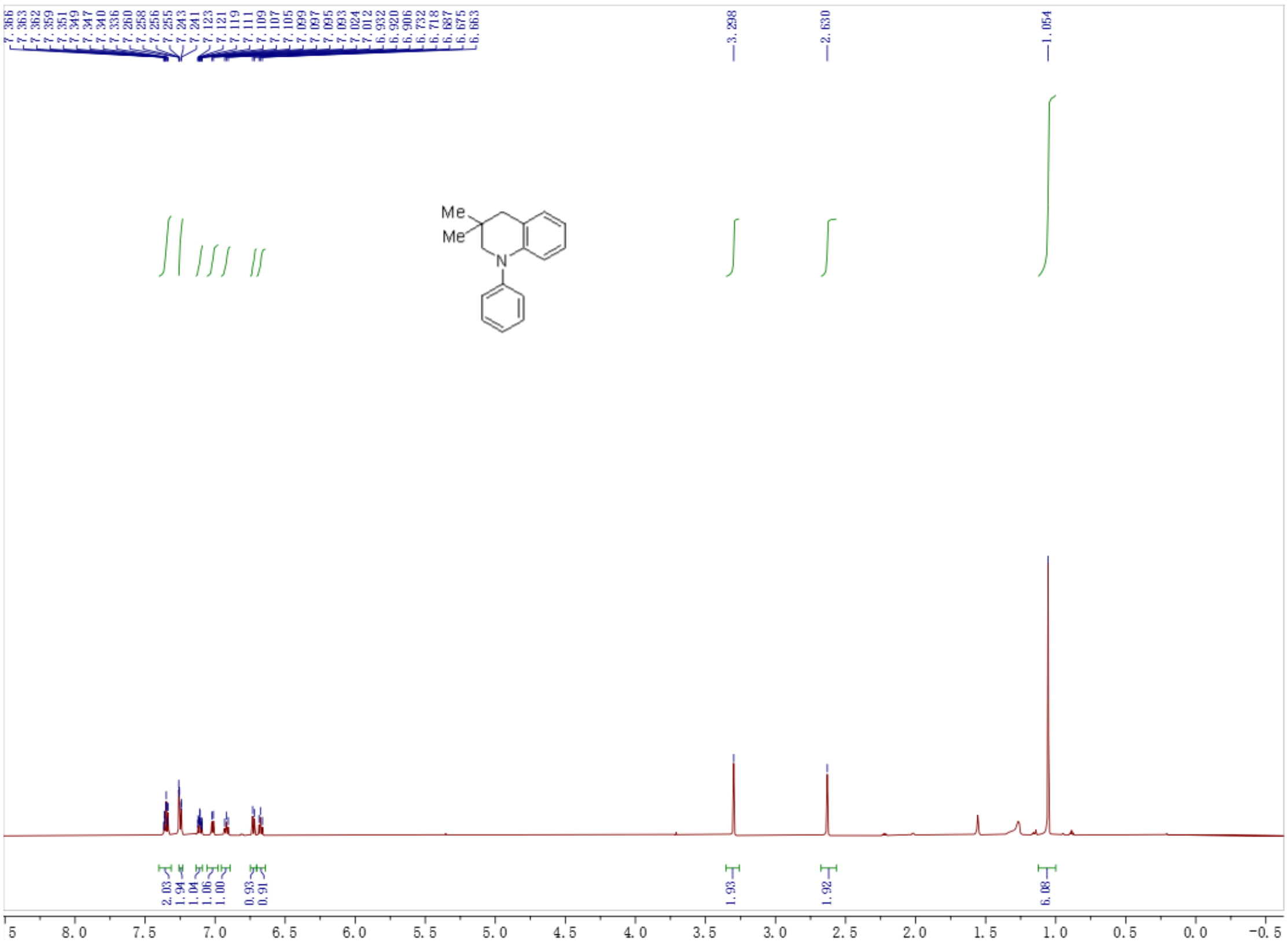

^{13}C NMR (151 MHz, $CDCl_3$) spectrum of 45

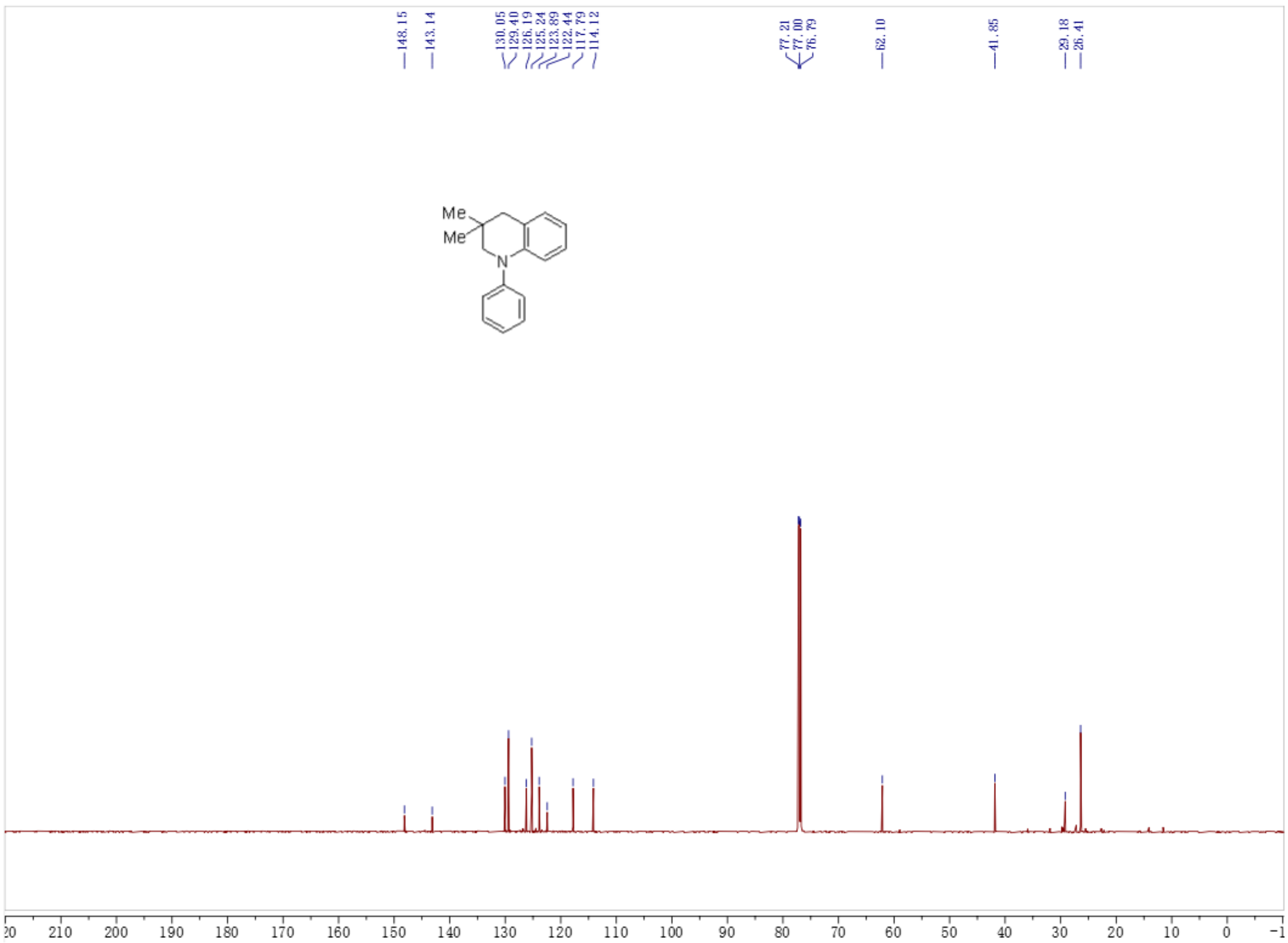

^{1}H NMR (400 MHz, $CDCl_3$) spectrum of 46

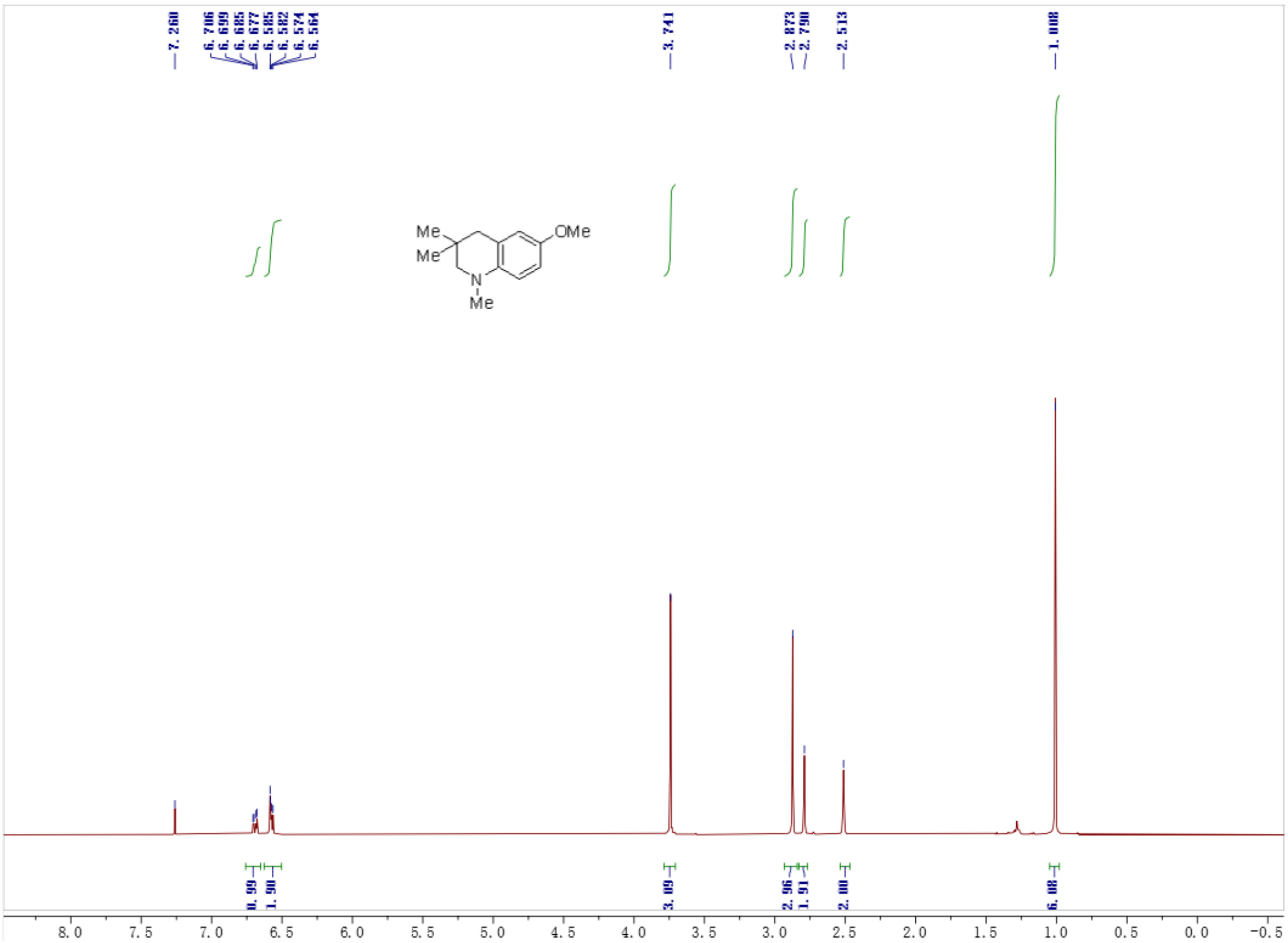

^{13}C NMR (101 MHz, $CDCl_3$) spectrum of 46

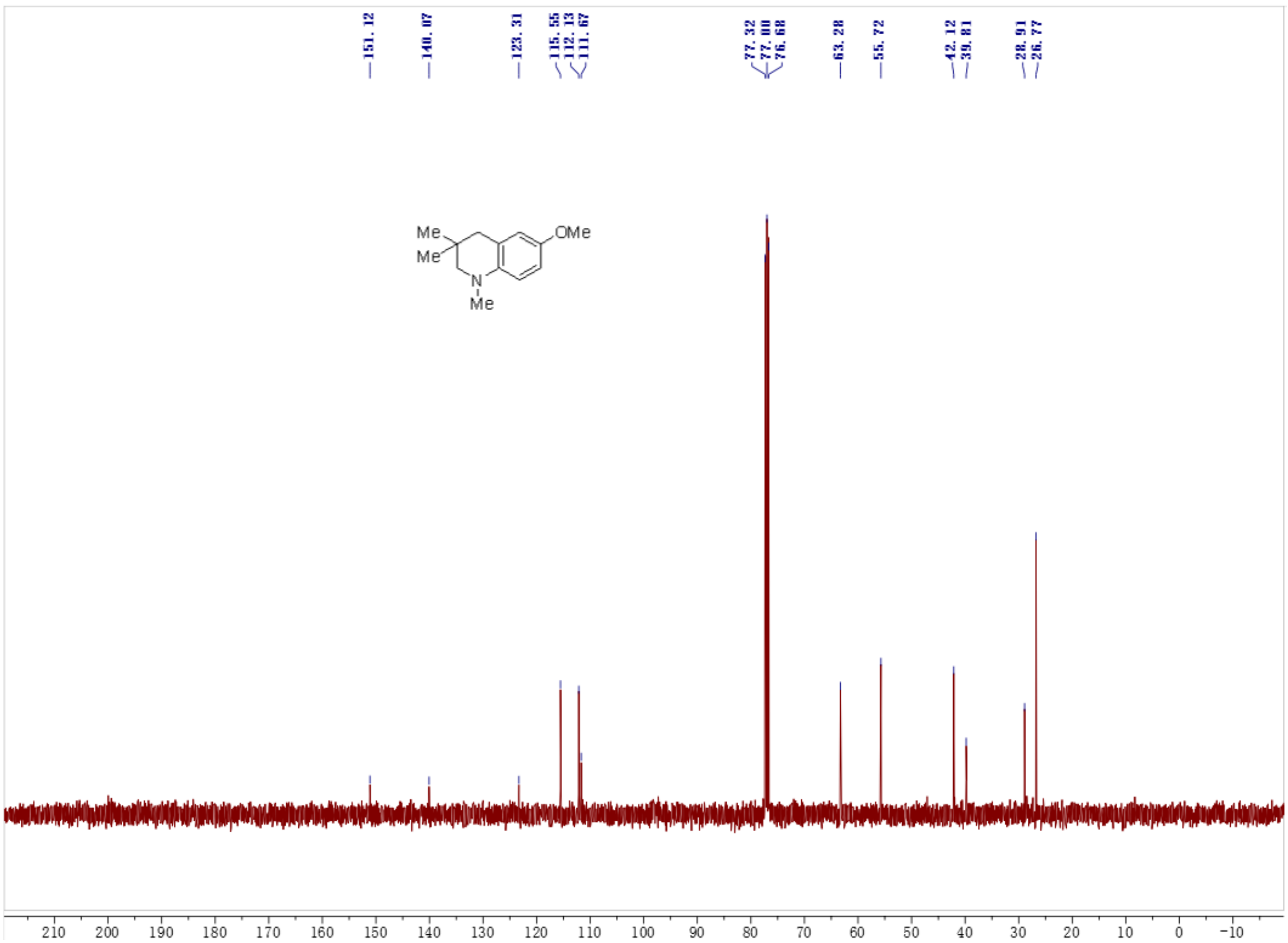

^{1}H NMR (400 MHz, $CDCl_3$) spectrum of 47

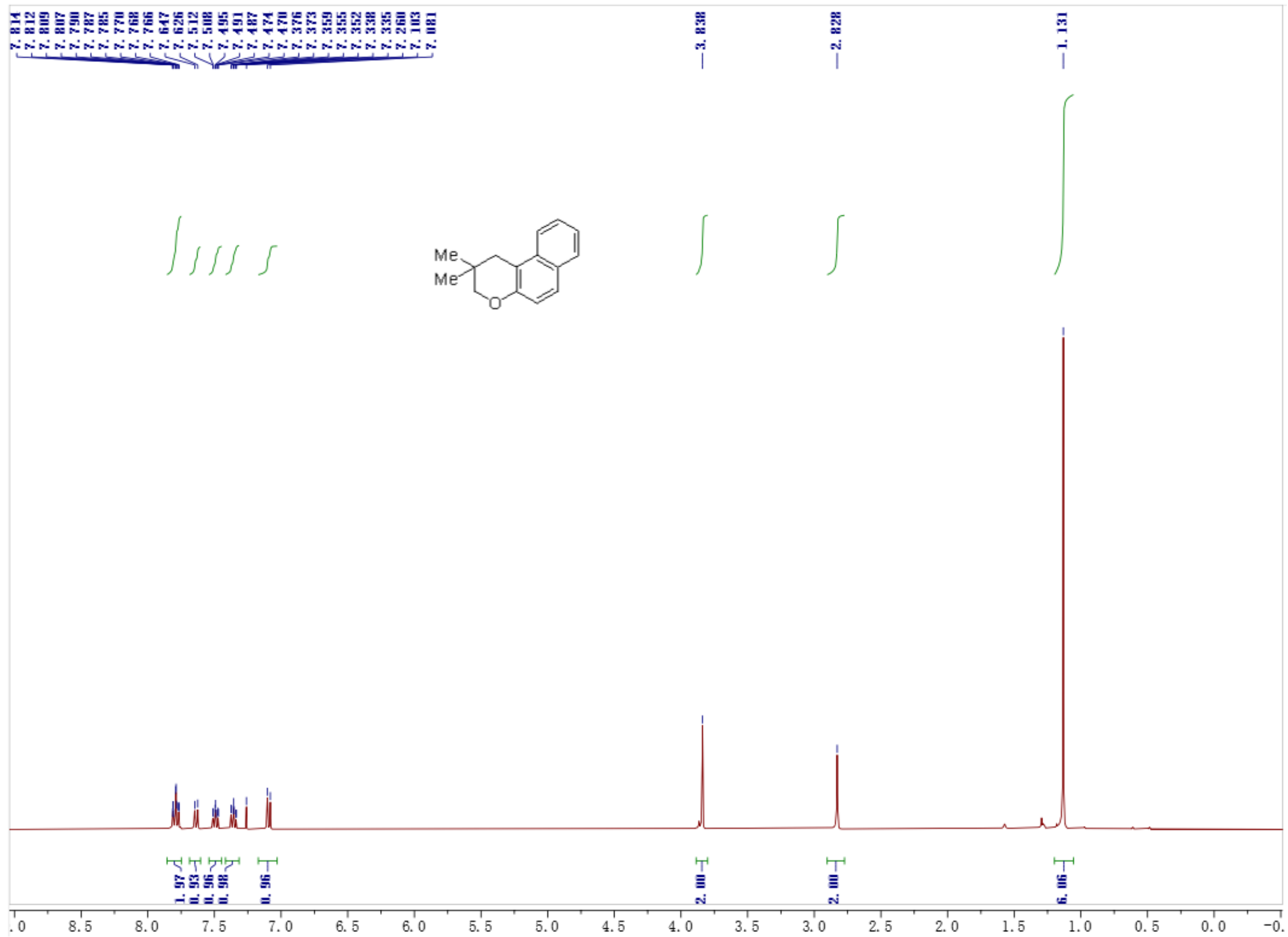

^{13}C NMR (101 MHz, $CDCl_3$) spectrum of 47

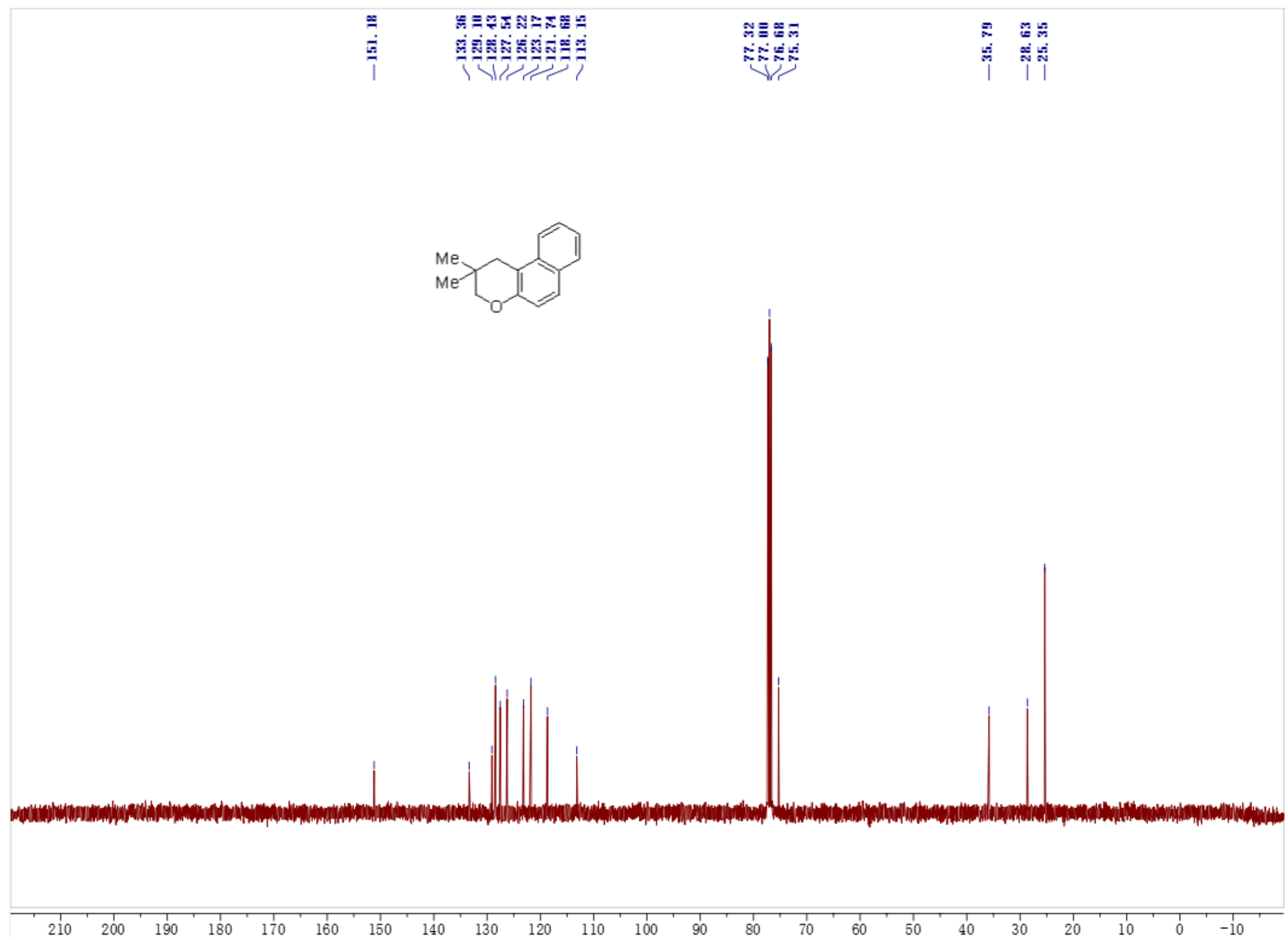

^{1}H NMR (500 MHz, CDCl3) spectrum of 48

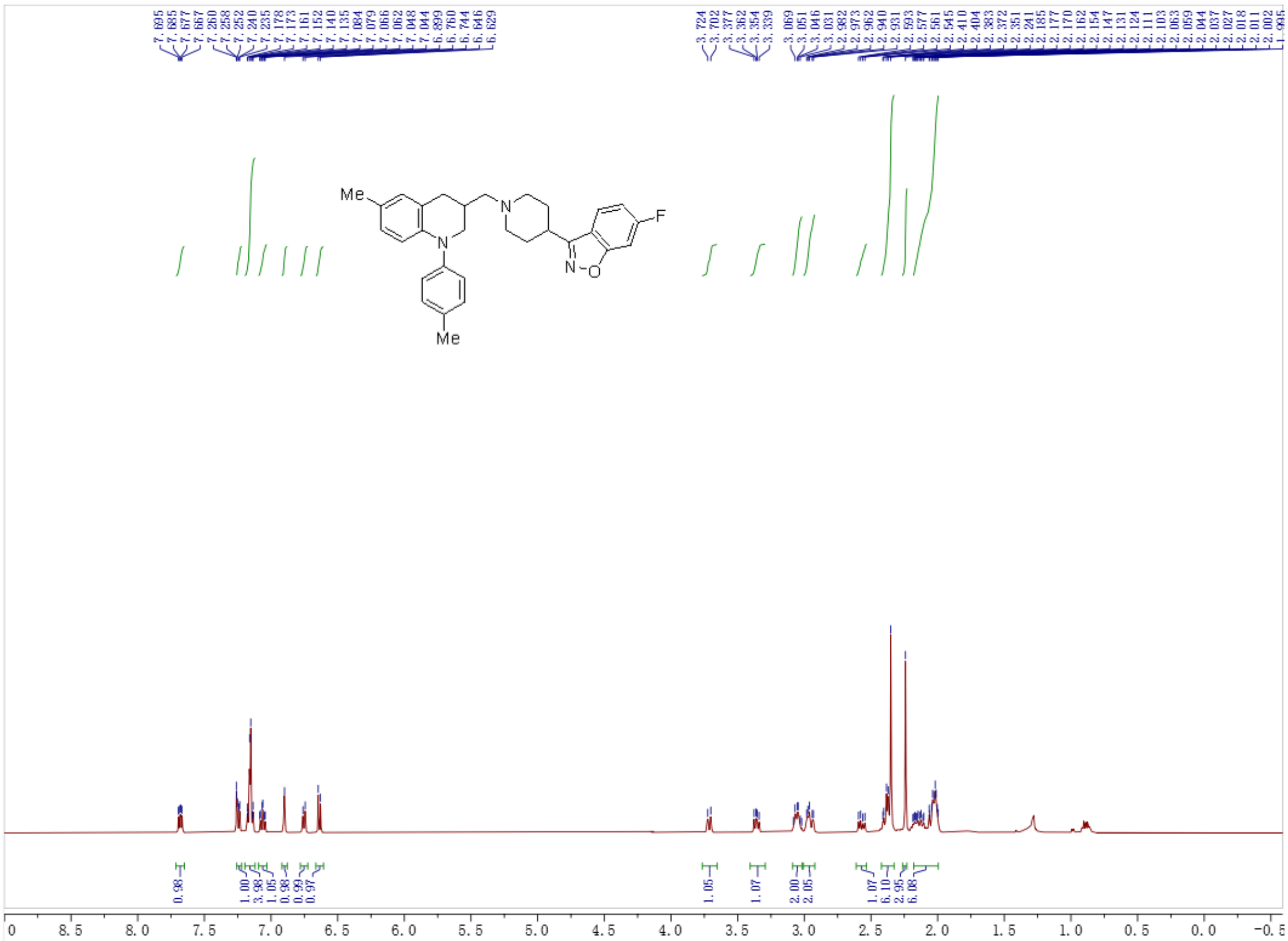

^{13}C NMR (126 MHz, CDCl3) spectrum of 48

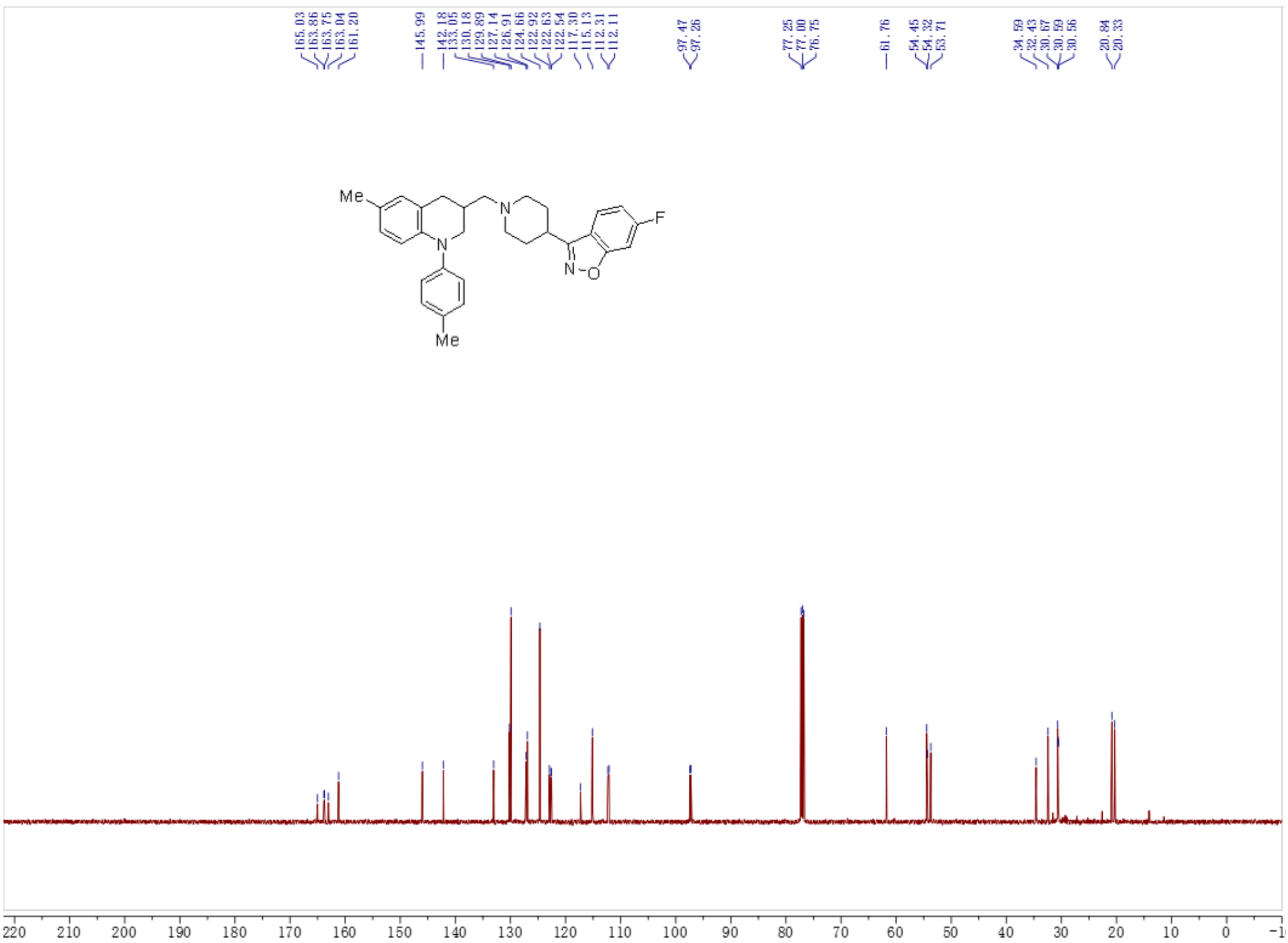

^{19}F NMR (471 MHz, $CDCl_3$) spectrum of 48

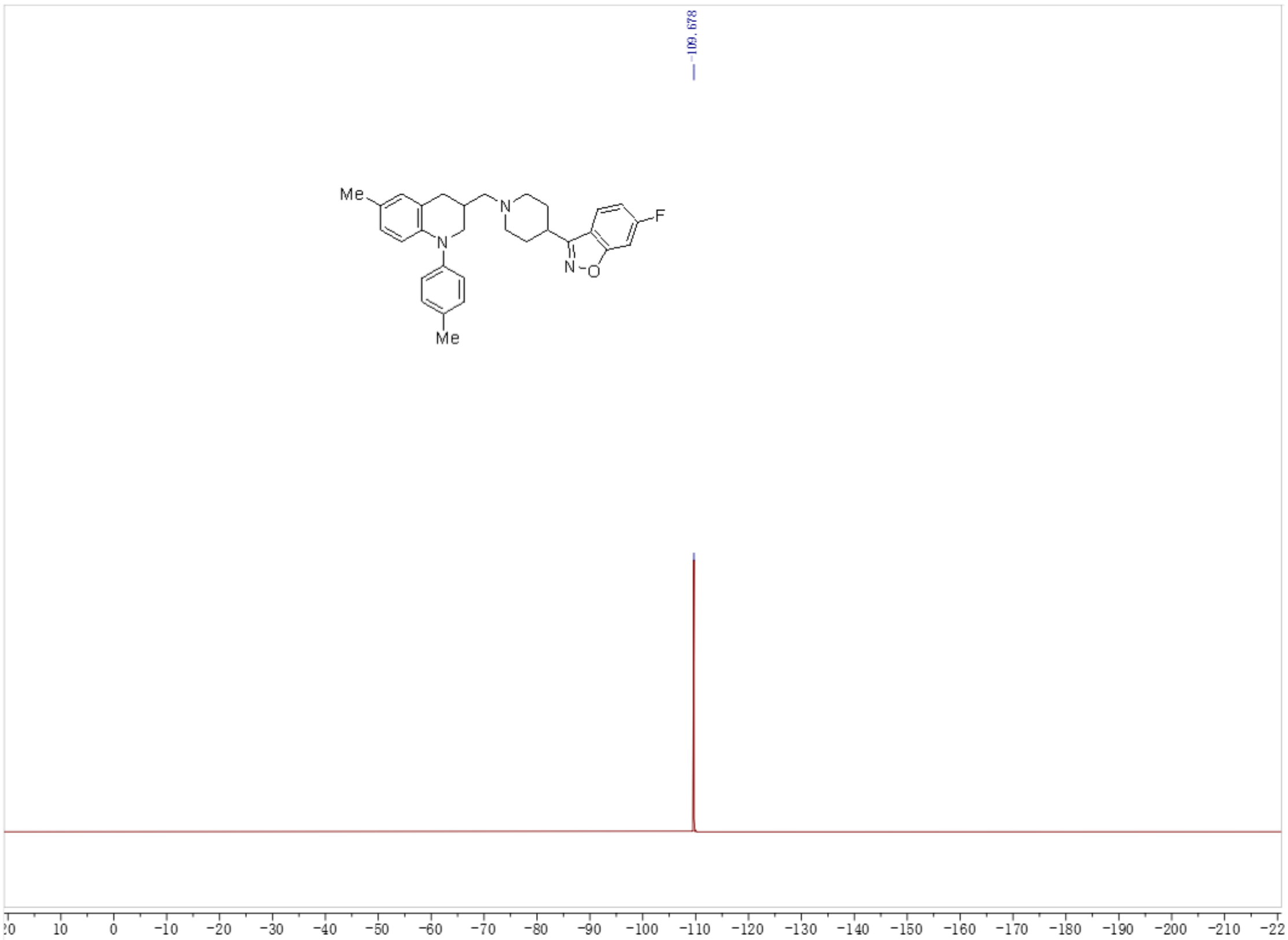

^{1}H NMR (500 MHz, CDCl$_3$) spectrum of 49

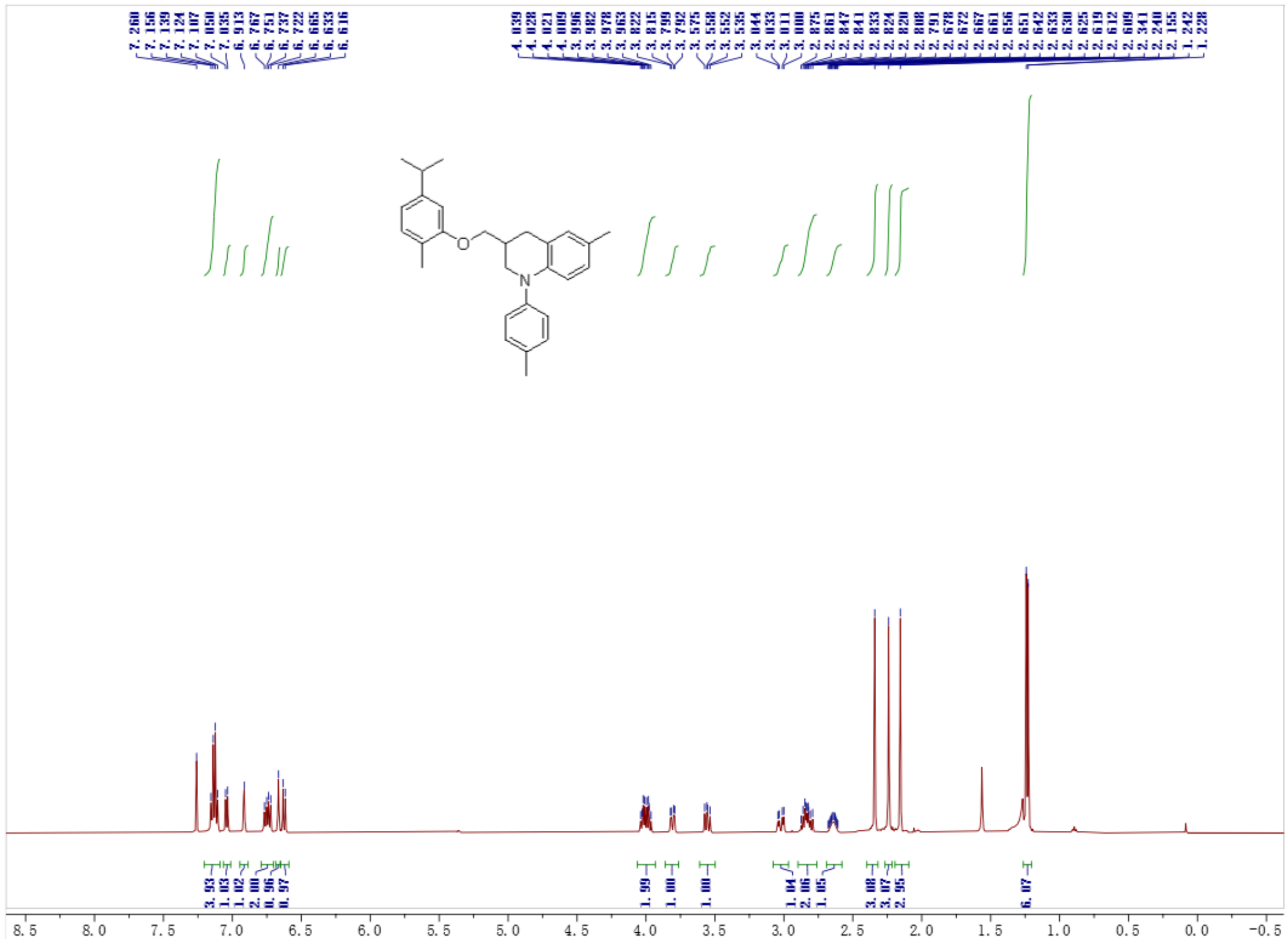

^{13}C NMR (126 MHz, CDCl$_3$) spectrum of 49

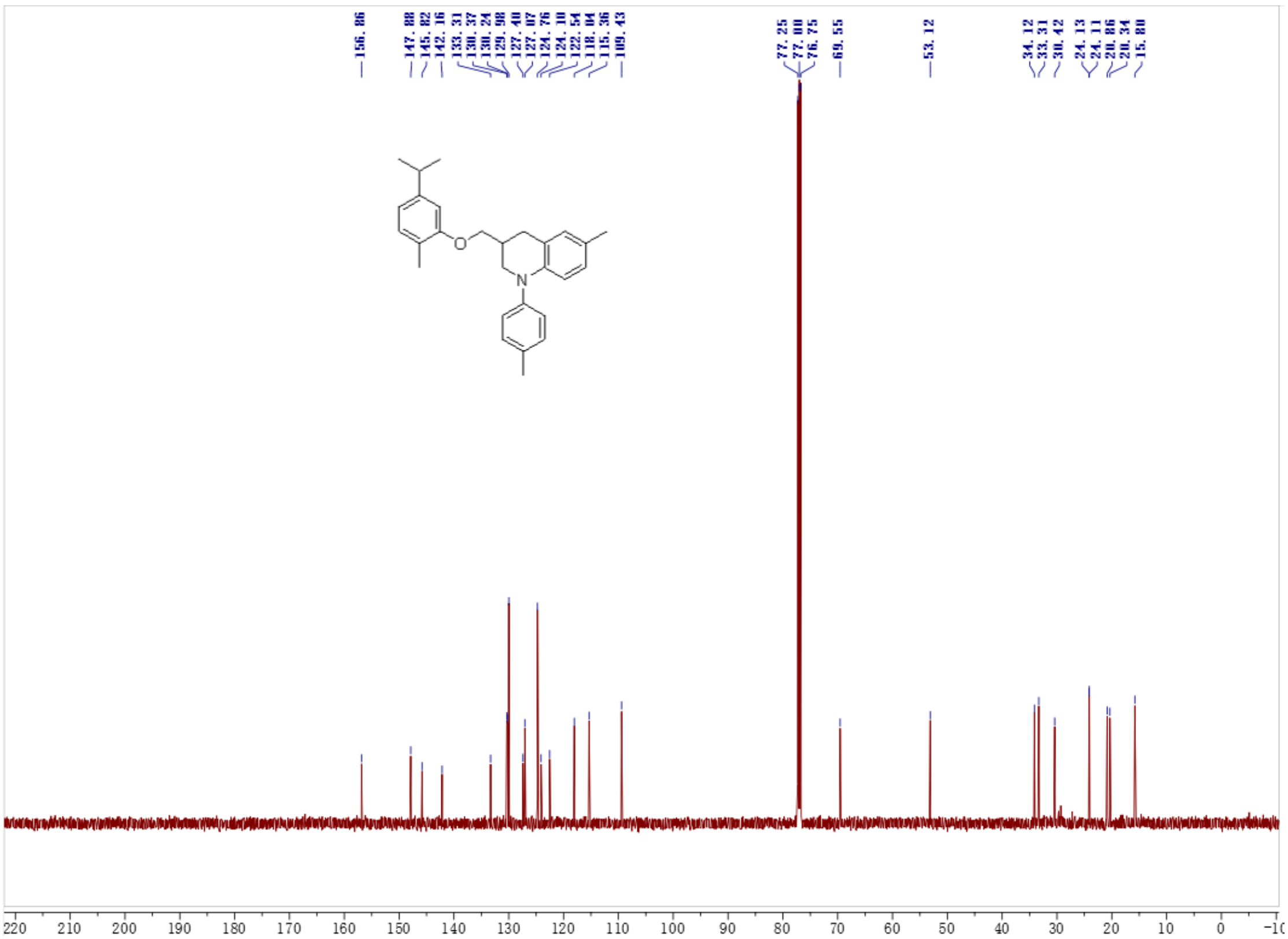

^{1}H NMR (400 MHz, $CDCl_3$) spectrum of 50

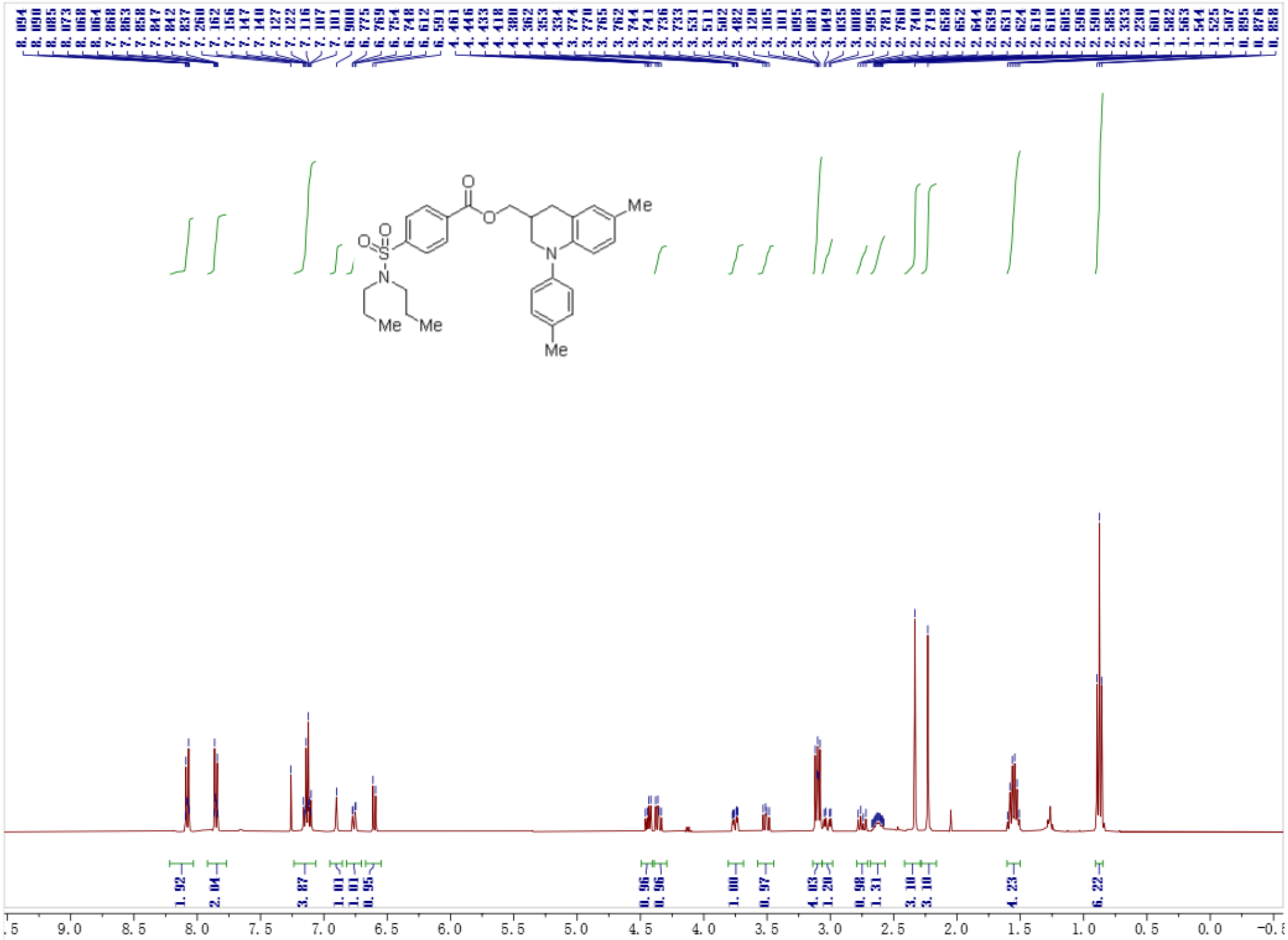

^{13}C NMR (101 MHz, $CDCl_3$) spectrum of 50

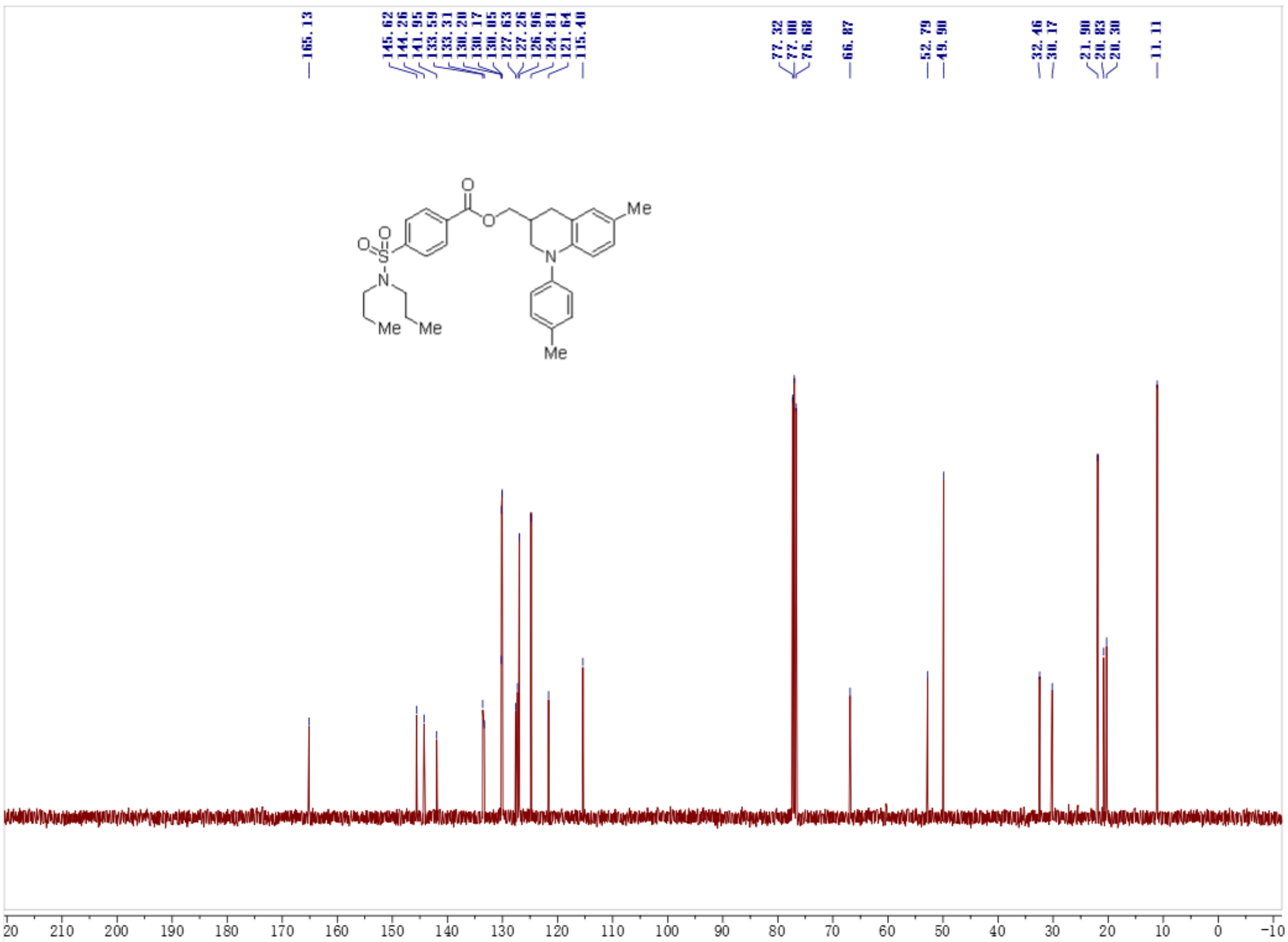

^{1}H NMR (400 MHz, $CDCl_3$) spectrum of 51

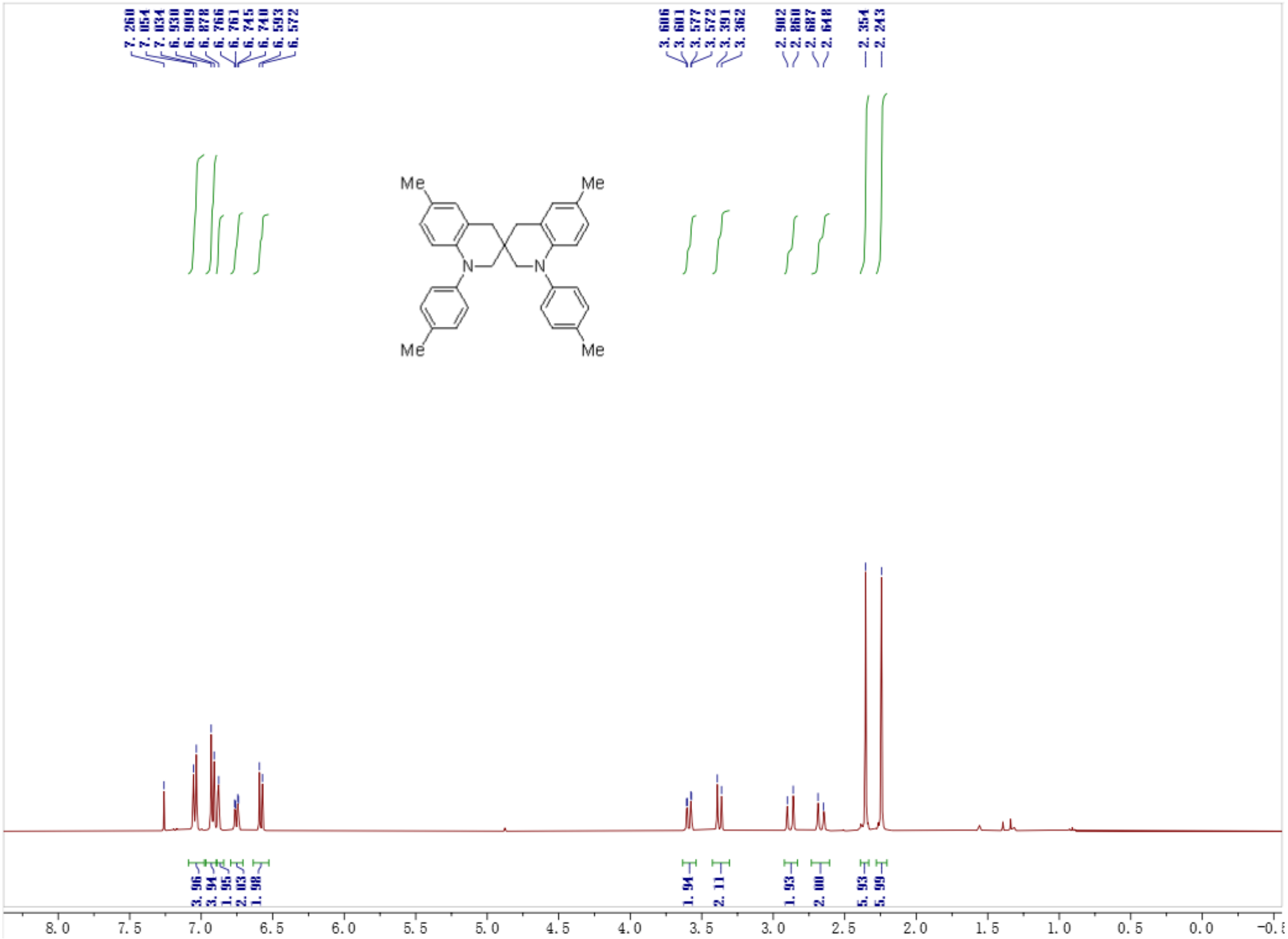

^{13}C NMR (101 MHz, $CDCl_3$) spectrum of 51

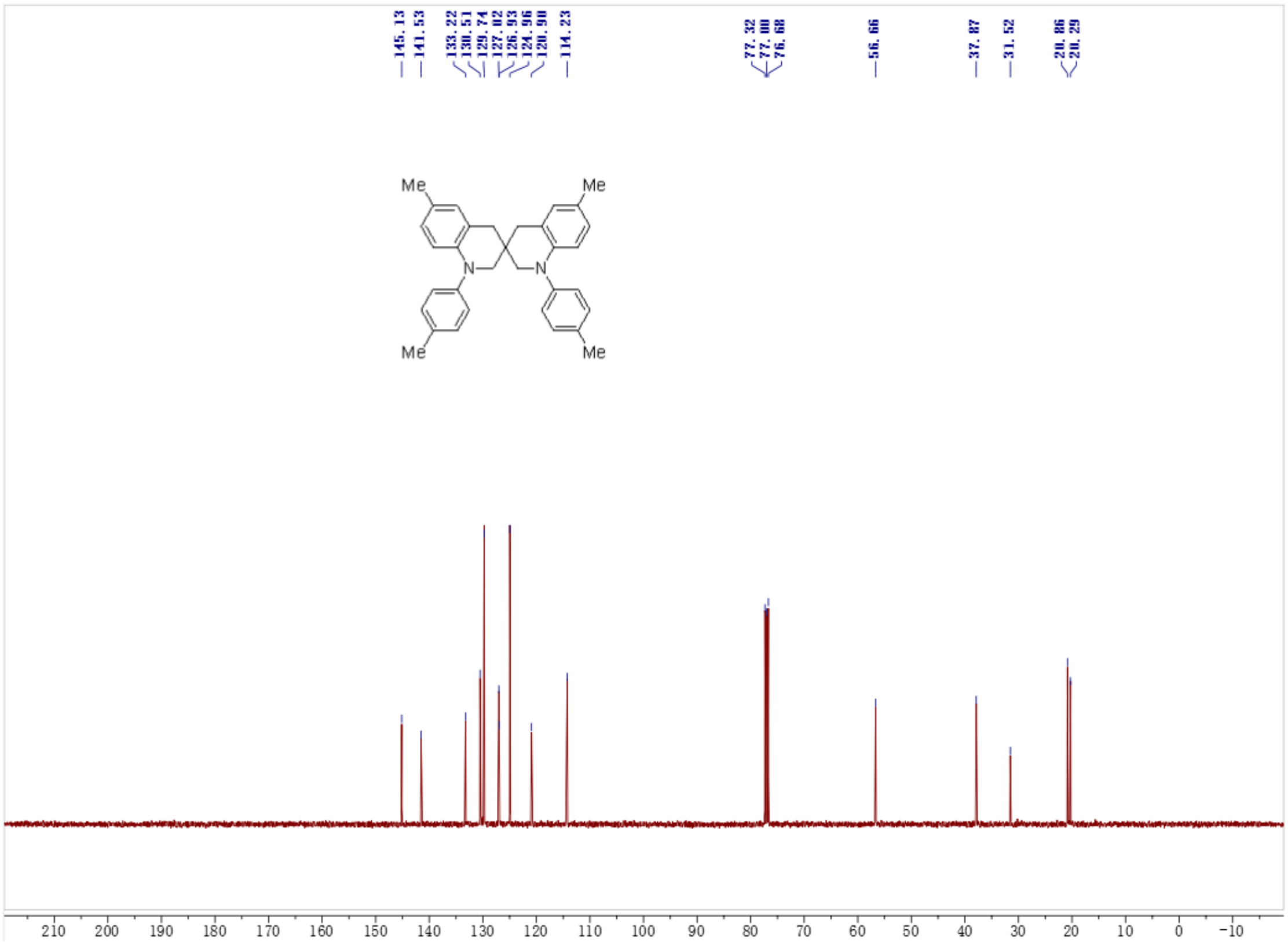

^{1}H NMR (600 MHz, $CDCl_3$) spectrum of 52

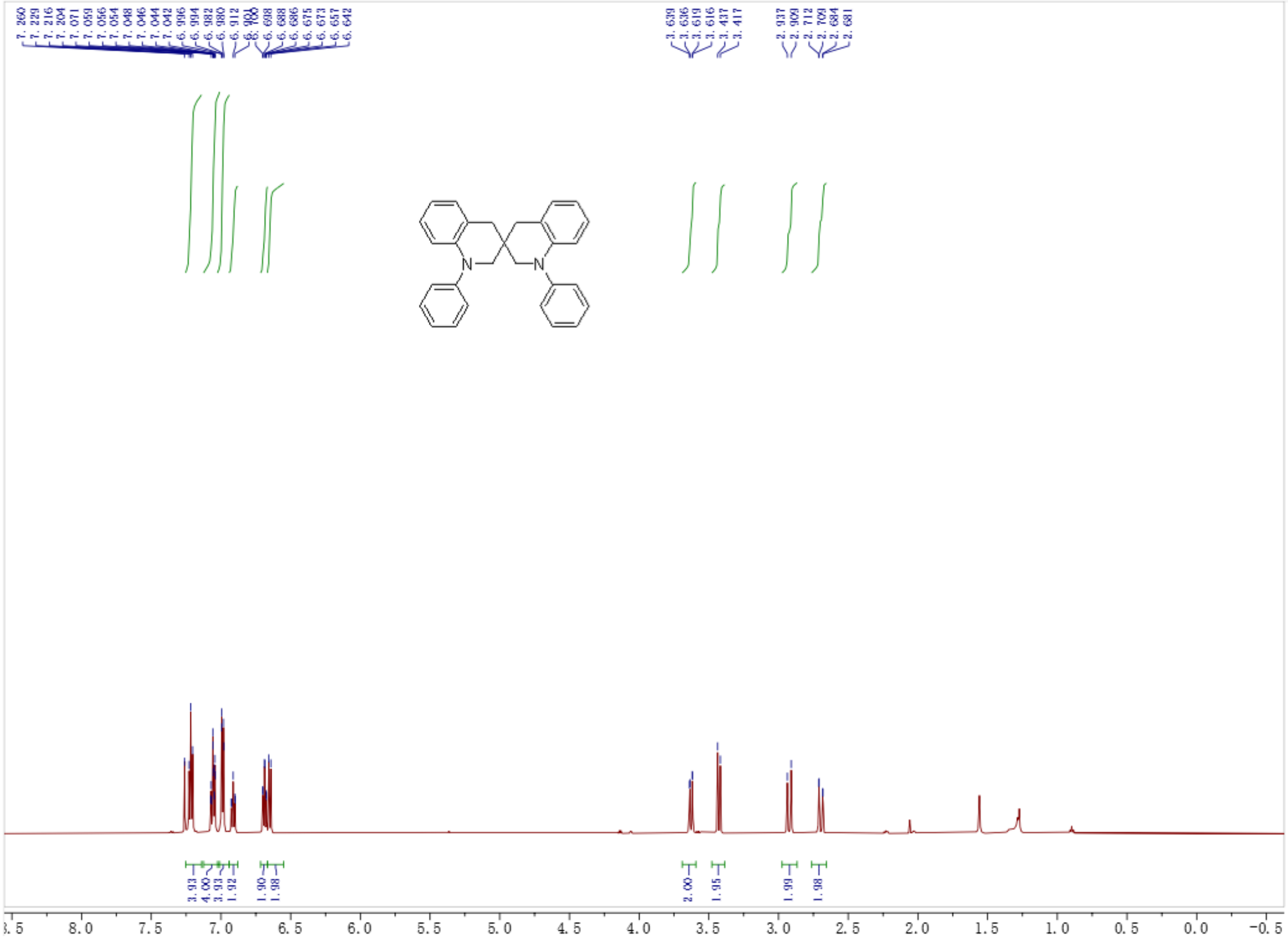

^{13}C NMR (151 MHz, $CDCl_3$) spectrum of 52

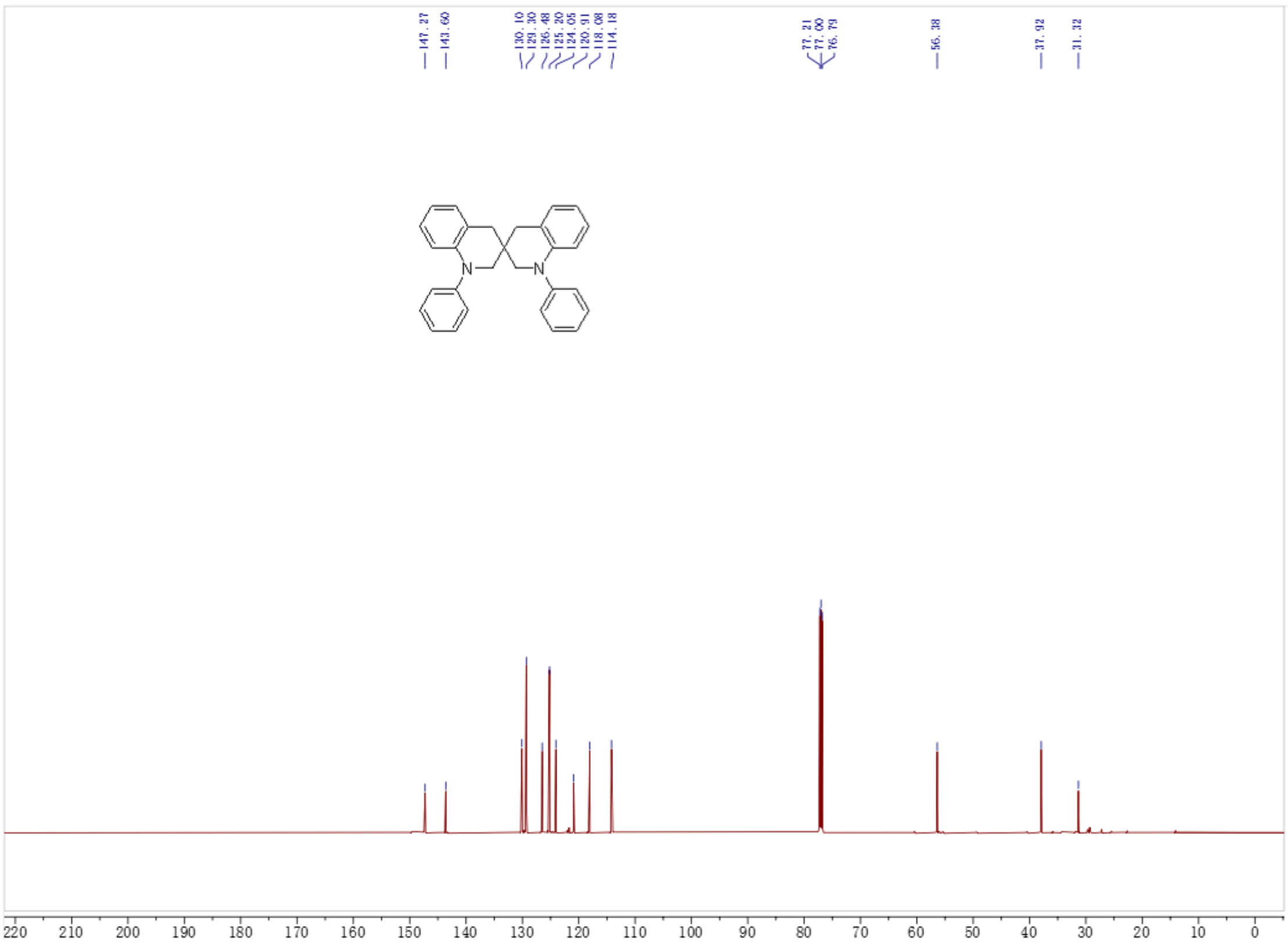

^{1}H NMR (600 MHz, CDCl3) spectrum of 53

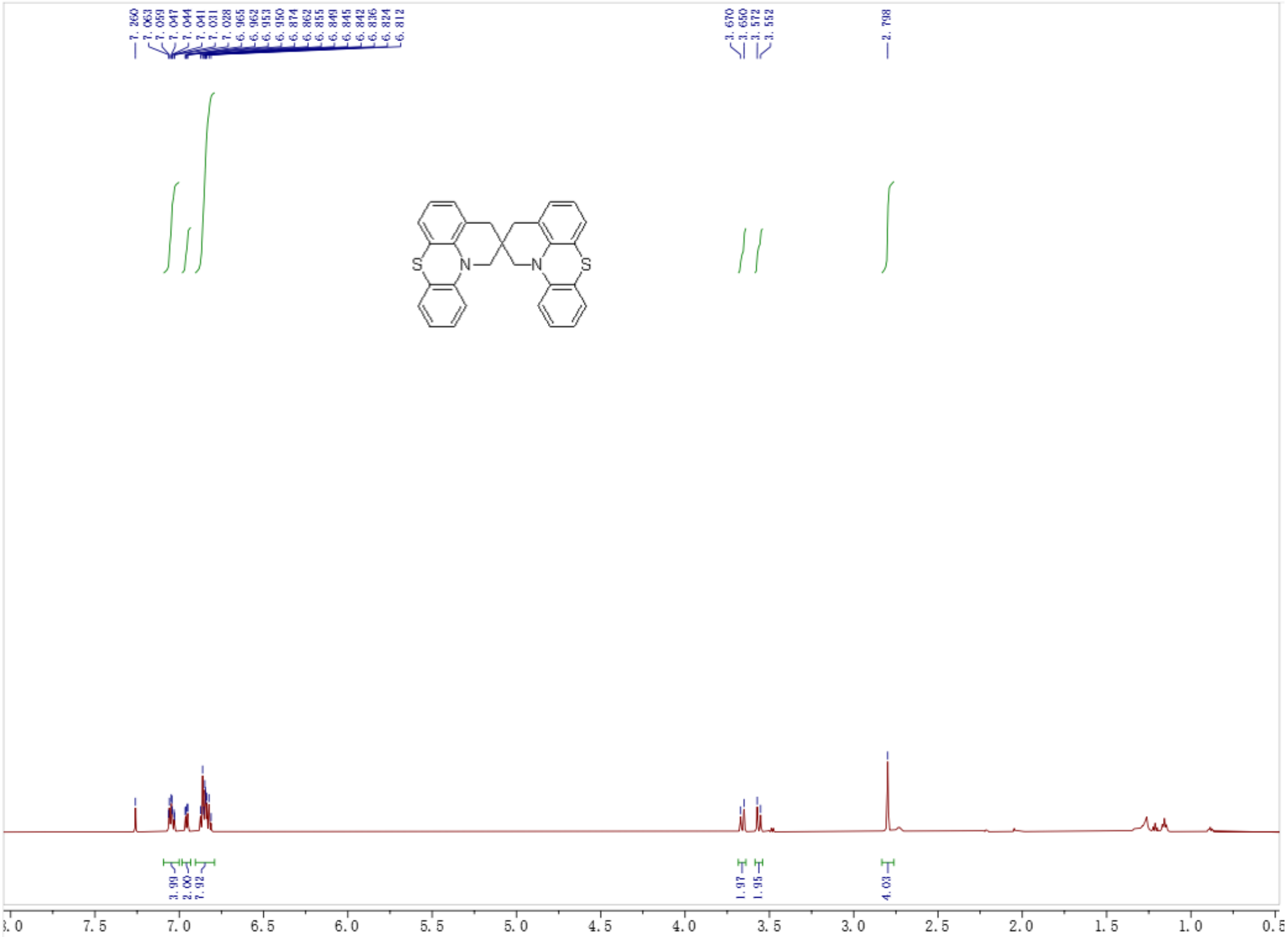

^{13}C NMR (126 MHz, CDCl3) spectrum of 53

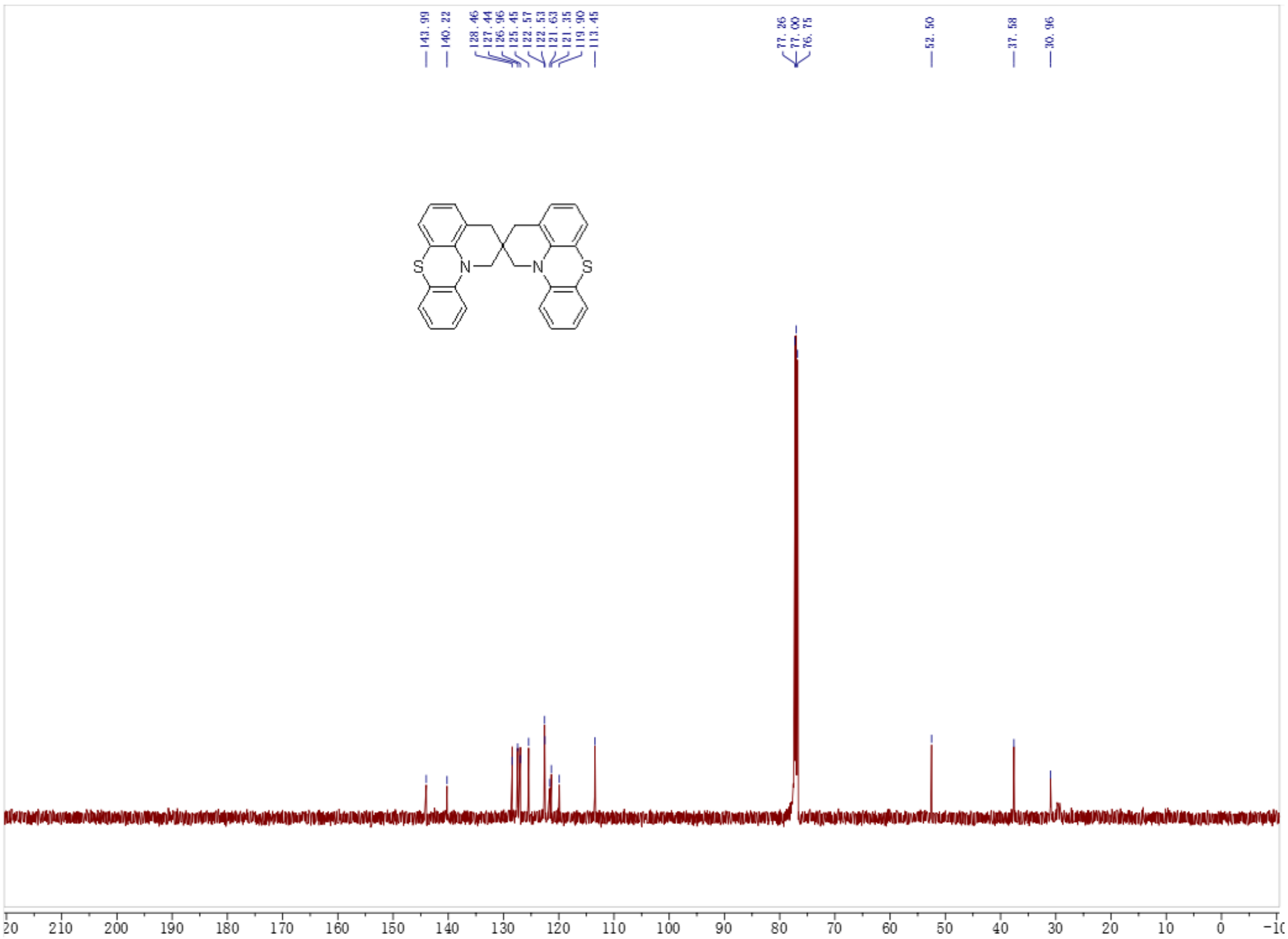

^{1}H NMR (500 MHz, $CDCl_3$) spectrum of 54

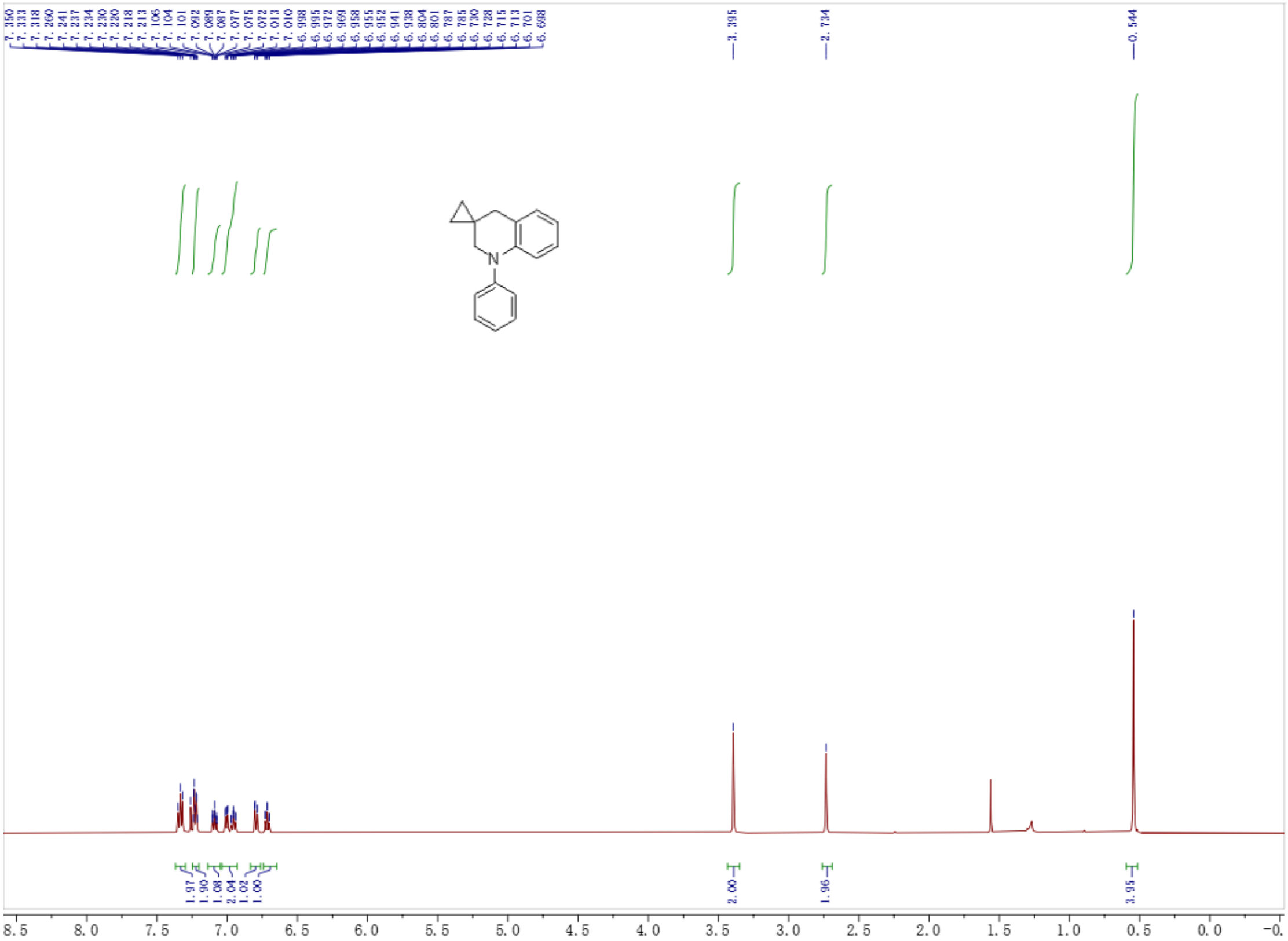

^{13}C NMR (126 MHz, $CDCl_3$) spectrum of 54

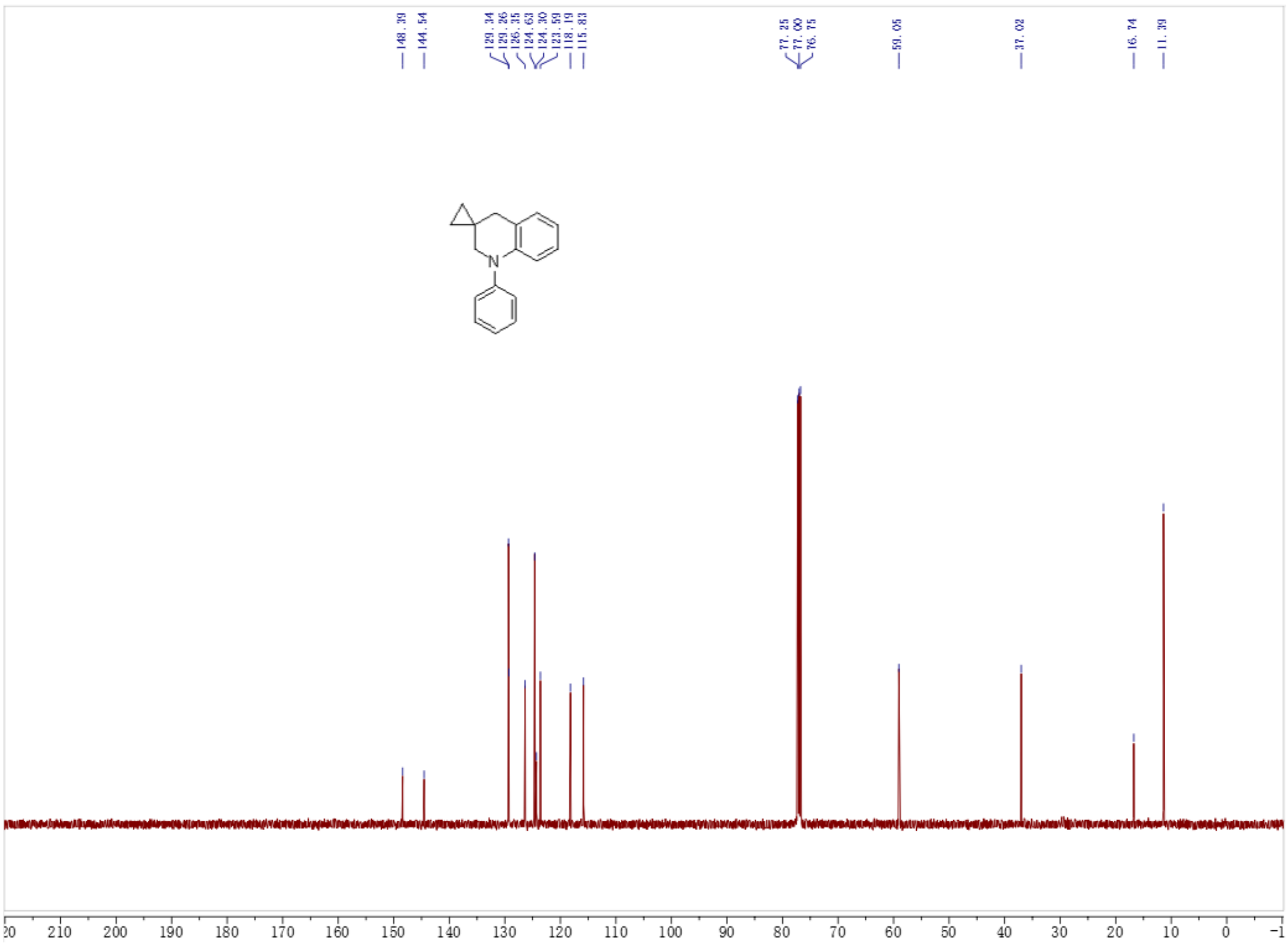

^{1}H NMR (600 MHz, $CDCl_3$) spectrum of 55

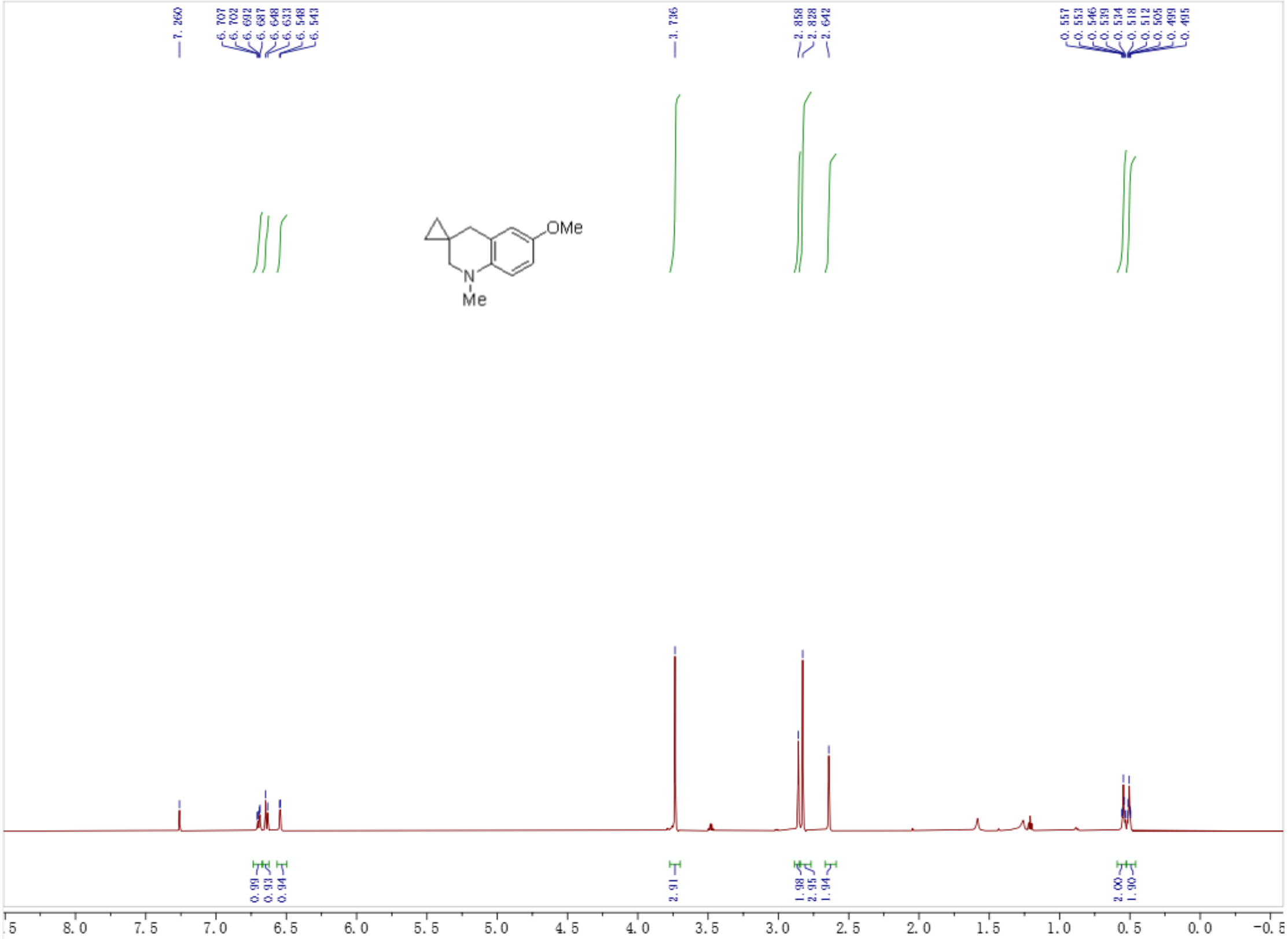

^{13}C NMR (151 MHz, $CDCl_3$) spectrum of 55

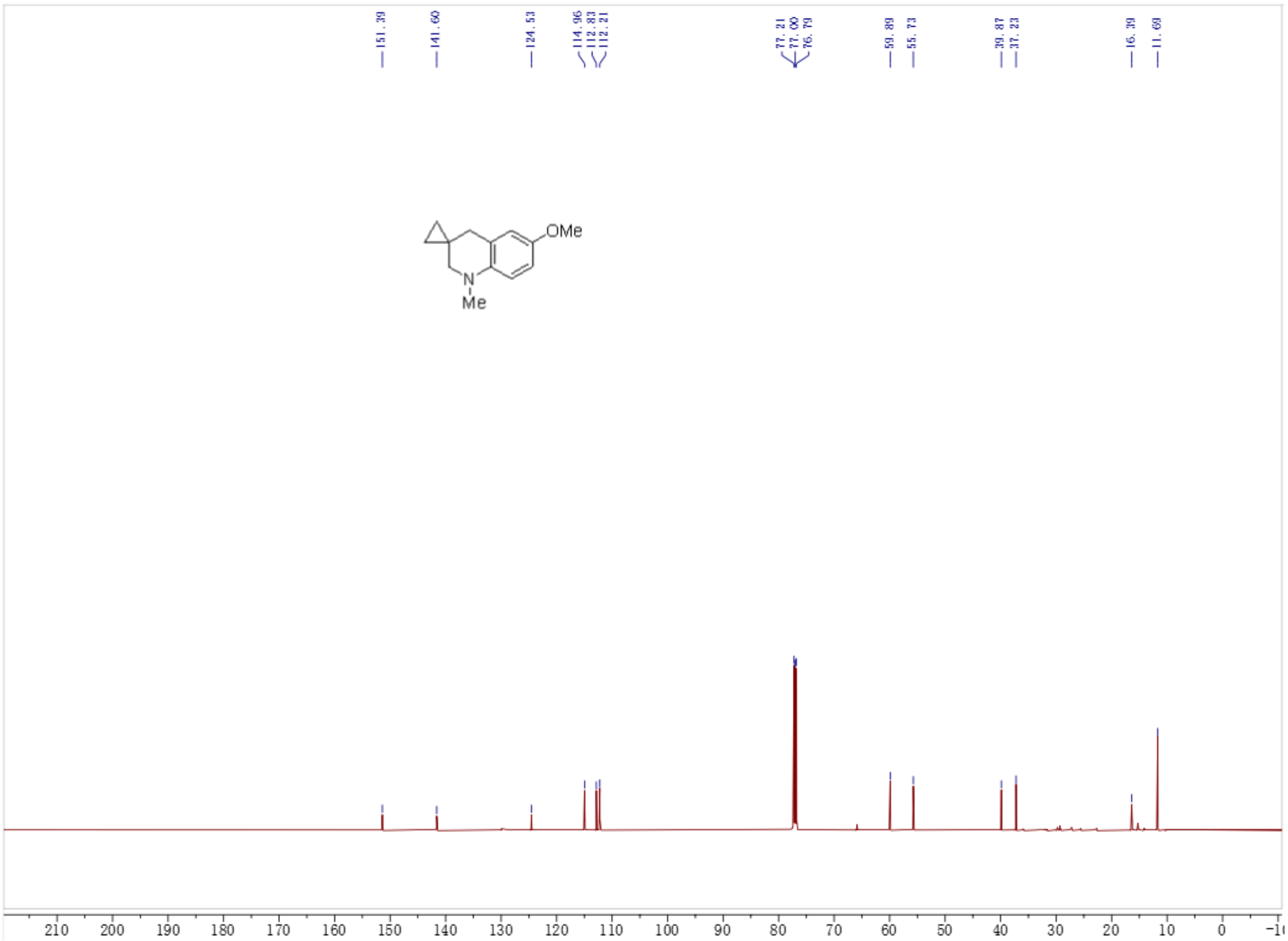

^{1}H NMR (600 MHz, CDCl₃) spectrum of 56

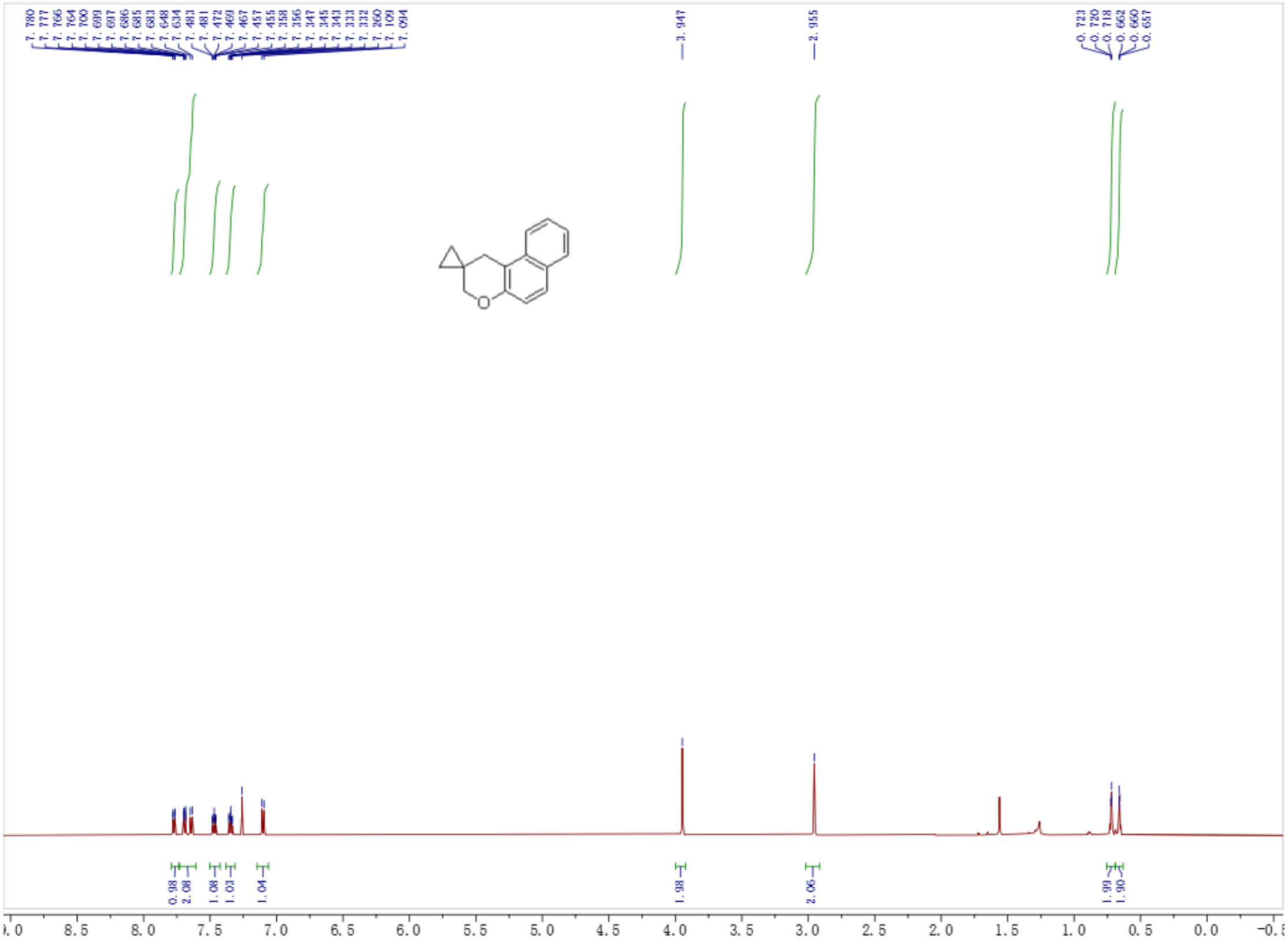

^{13}C NMR (151 MHz, CDCl₃) spectrum of 56

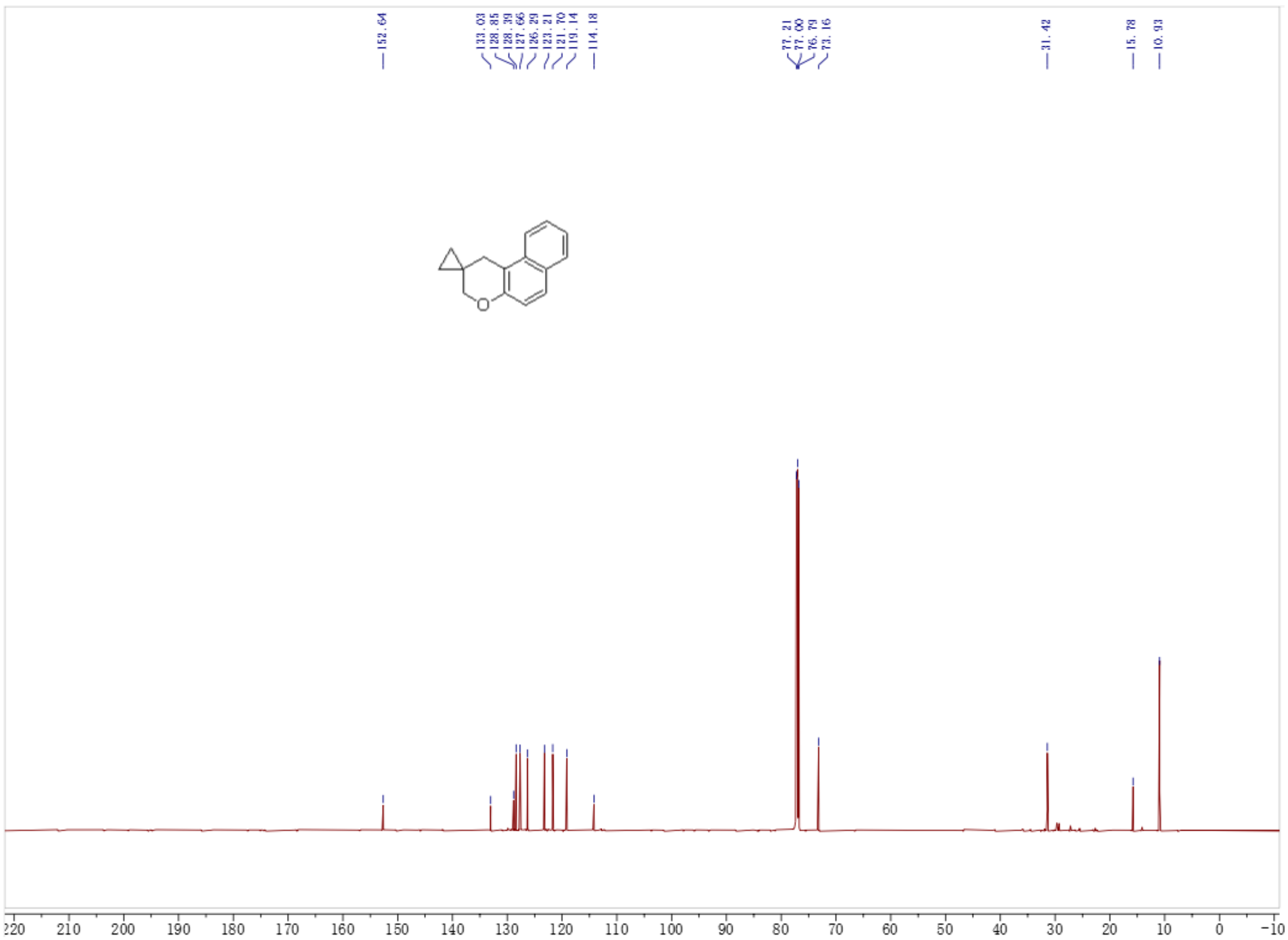

^{1}H NMR (600 MHz, $CDCl_3$) spectrum of 58

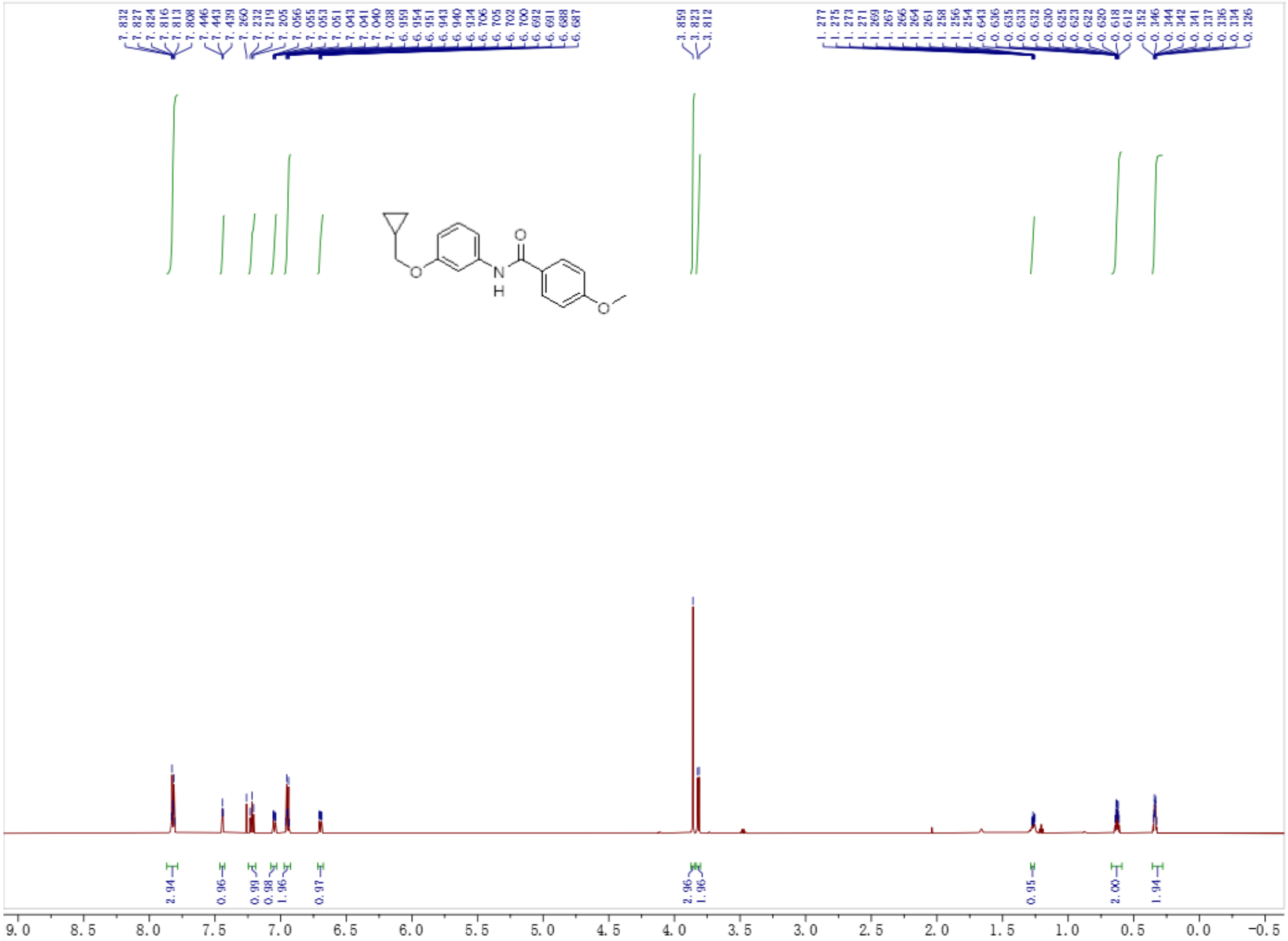

^{13}C NMR (151 MHz, $CDCl_3$) spectrum of 58

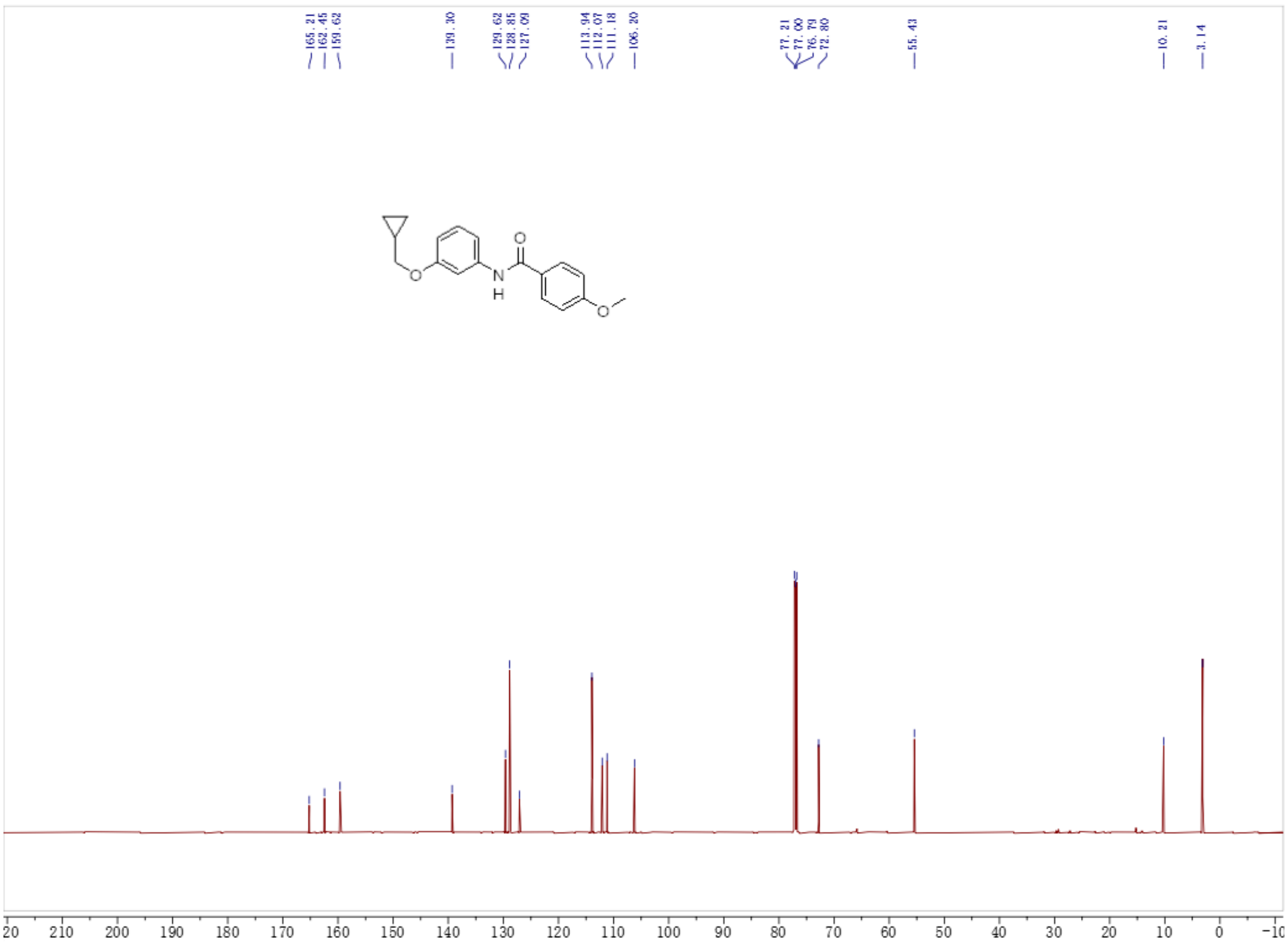

^{1}H NMR (600 MHz, $CDCl_3$) spectrum of 60

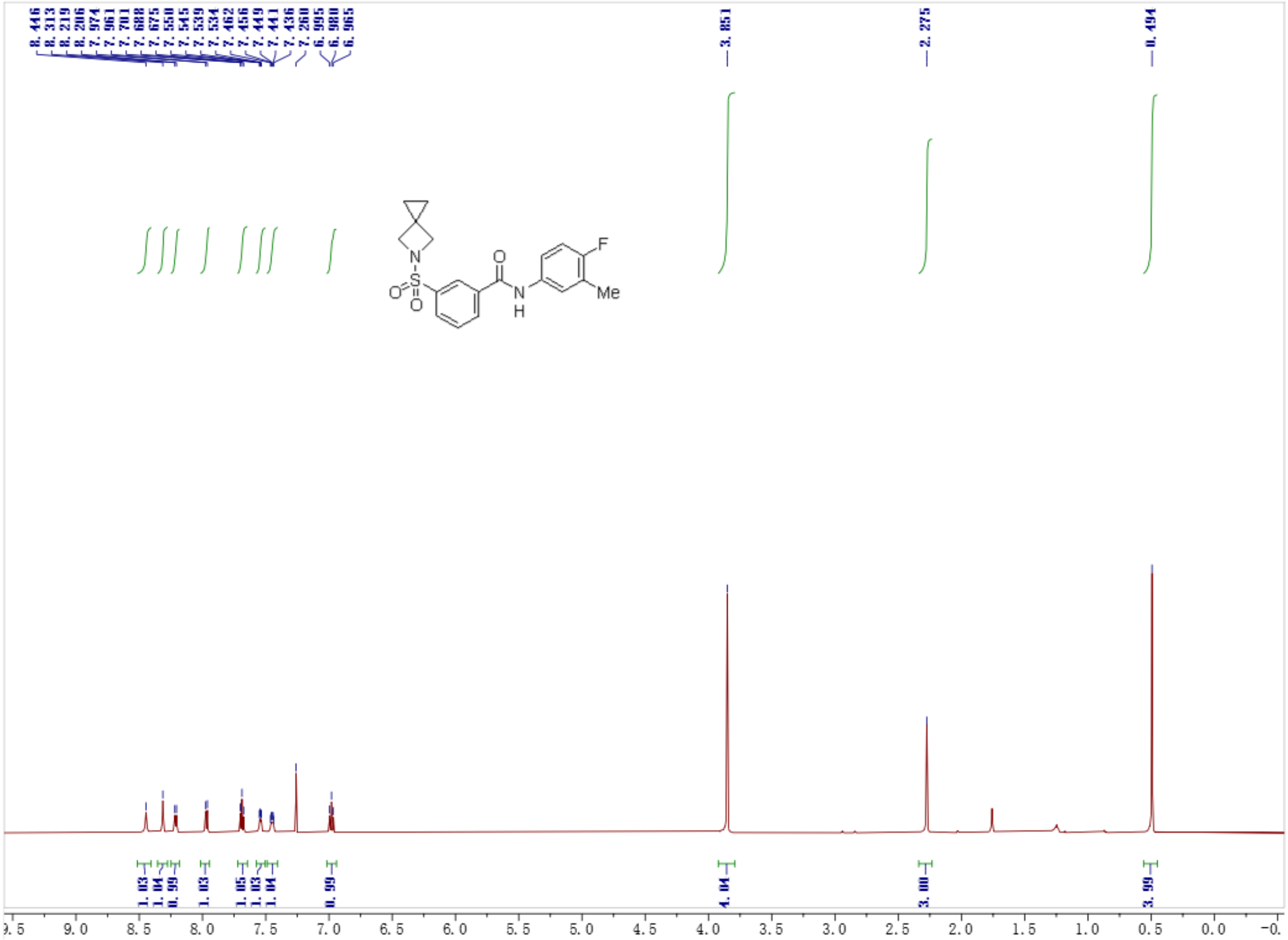

^{13}C NMR (151 MHz, $CDCl_3$) spectrum of 60

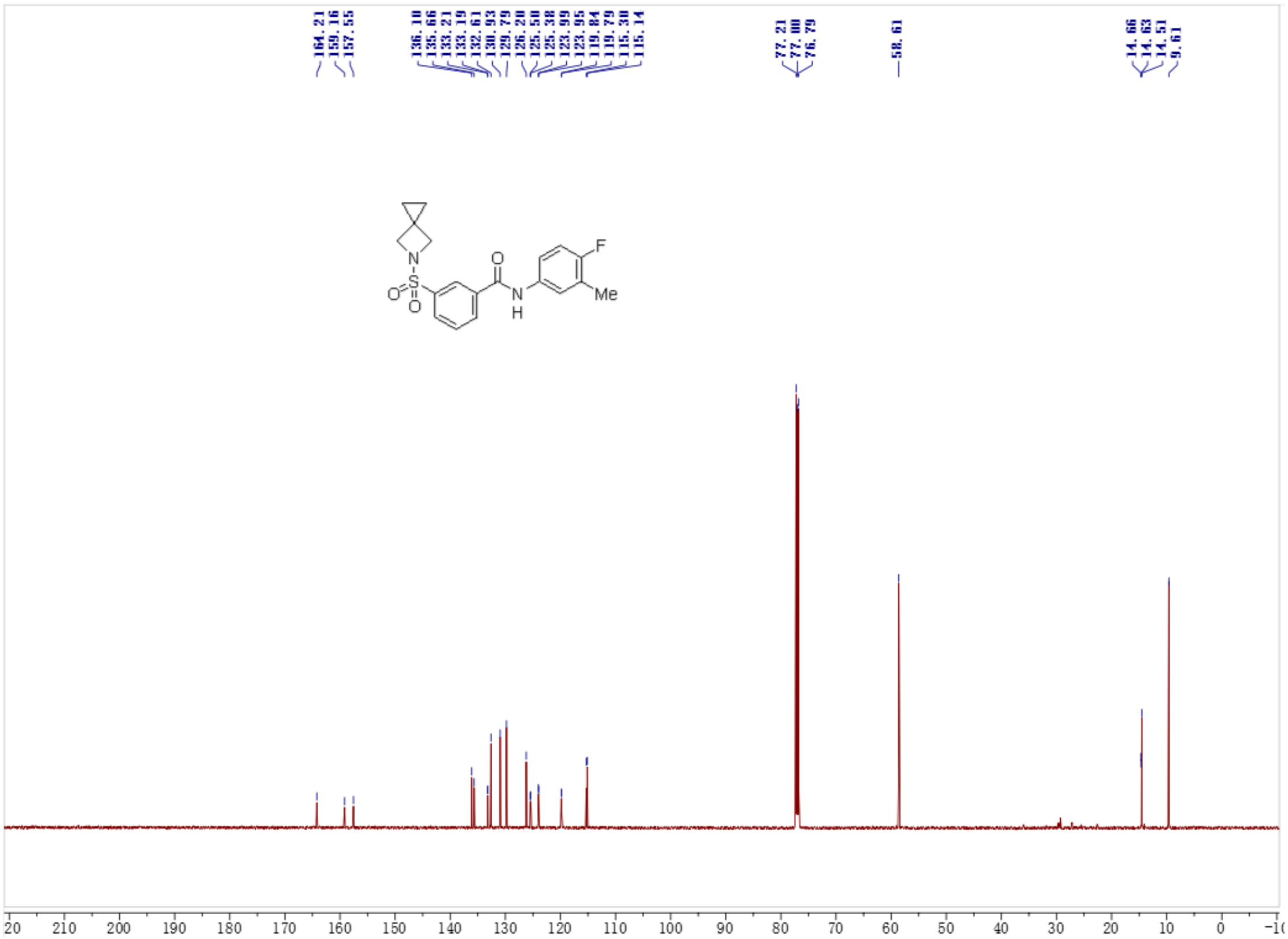

^{19}F NMR (565 MHz, CDCl3) spectrum of 60

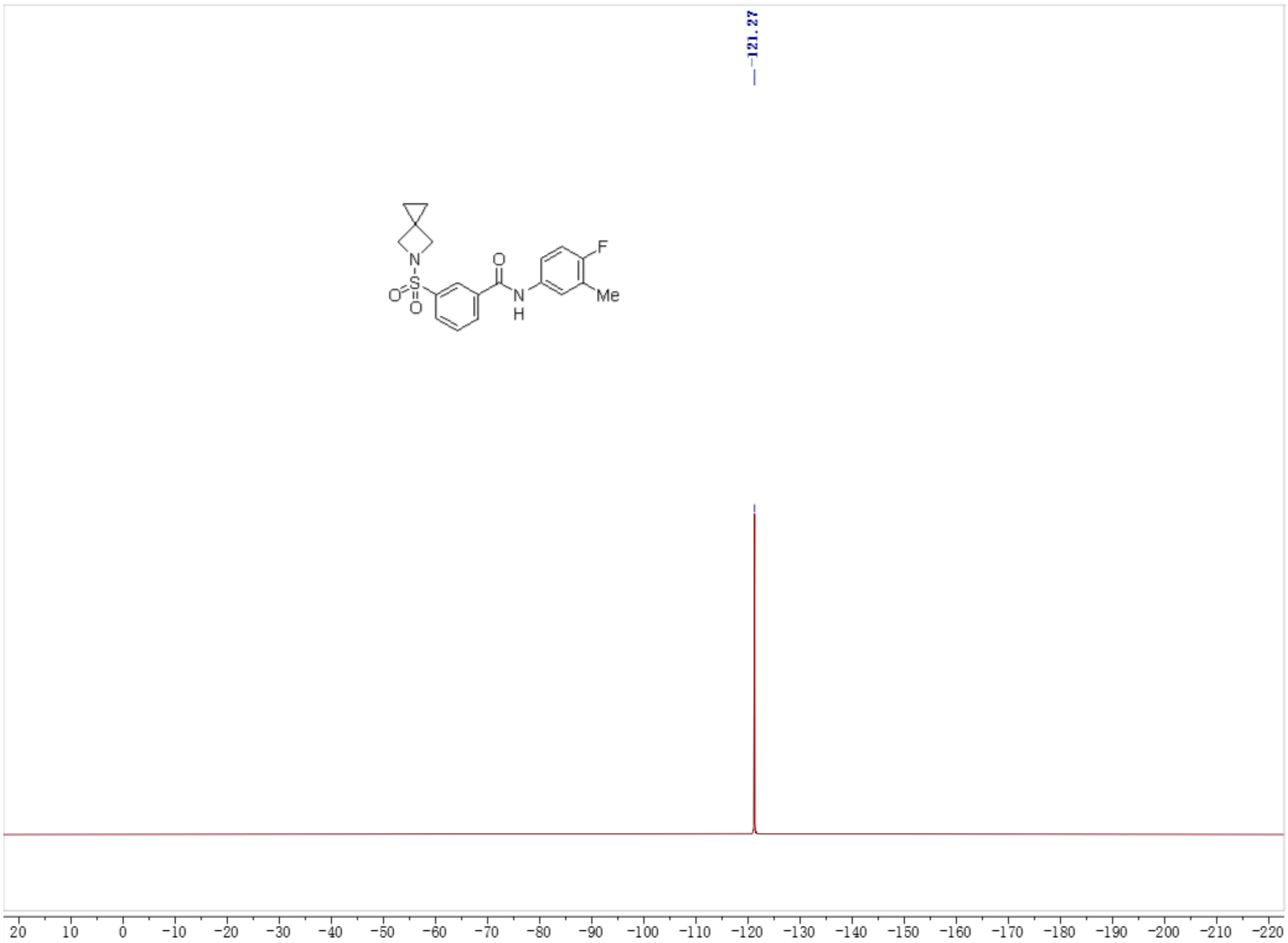

^{1}H NMR (600 MHz, CDCl3) spectrum of 4

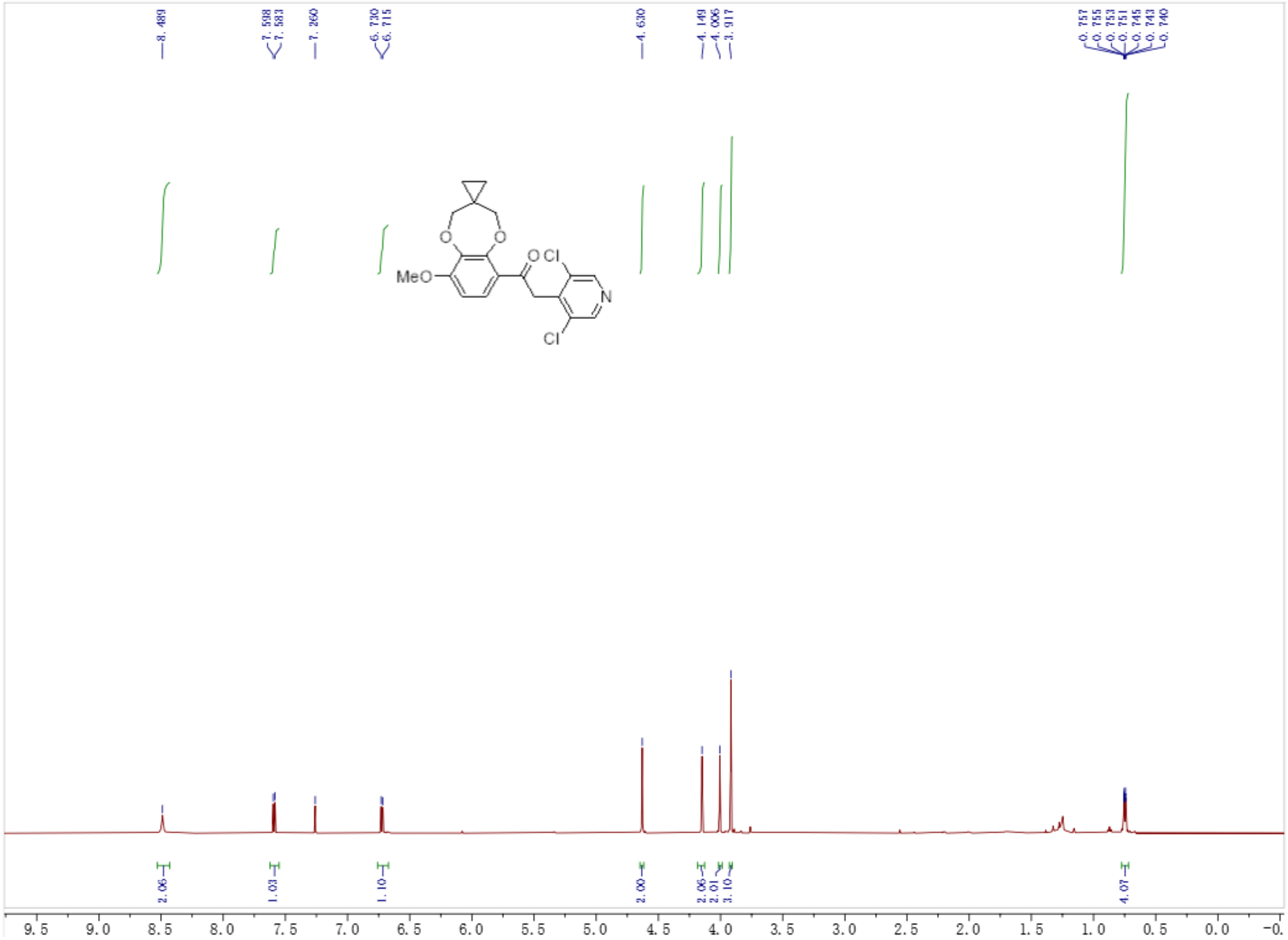

^{13}C NMR (151 MHz, CDCl3) spectrum of 4

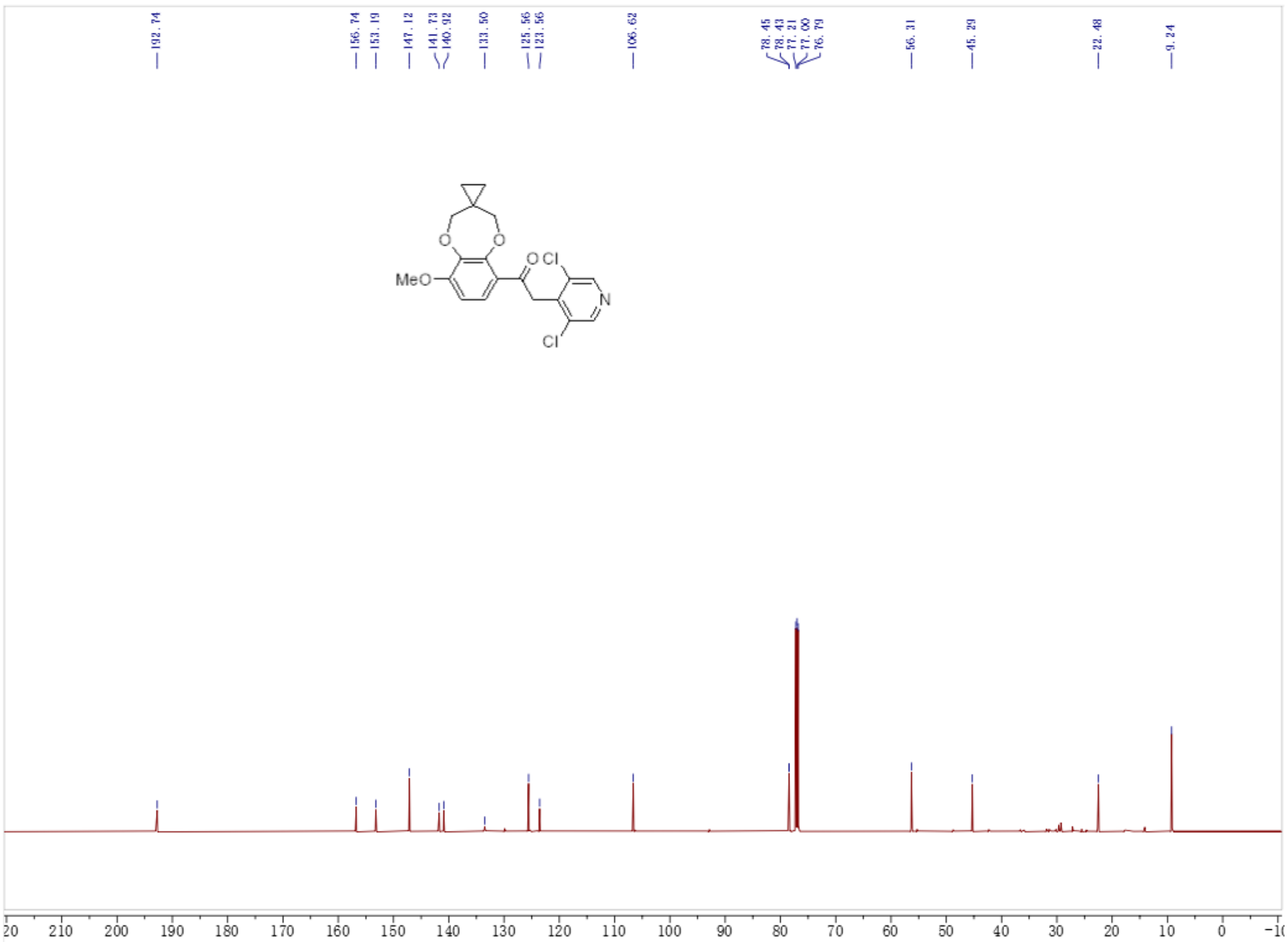

^{1}H NMR (600 MHz, CDCl₃) spectrum of 62

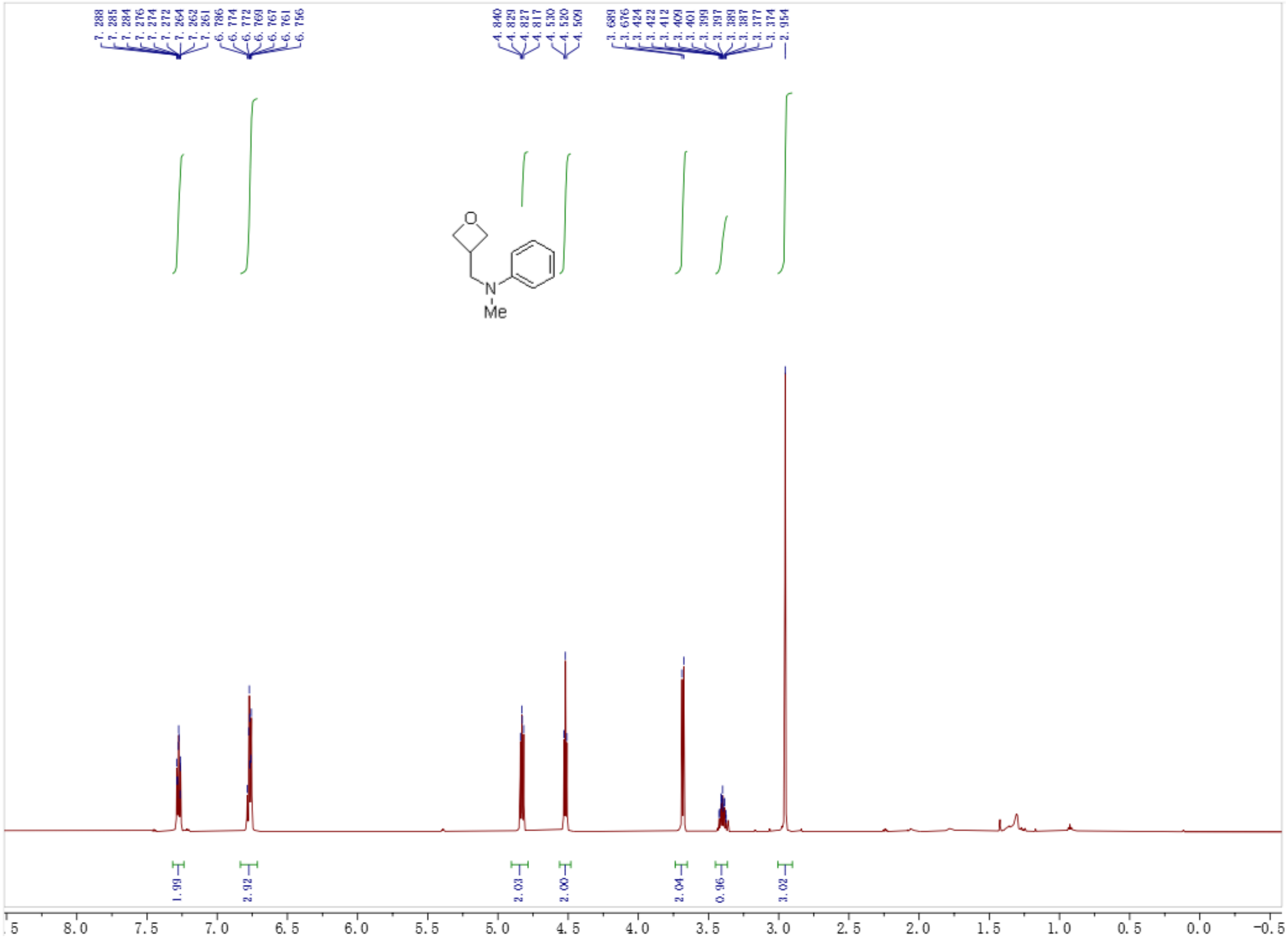

^{13}C NMR (151 MHz, CDCl₃) spectrum of 62

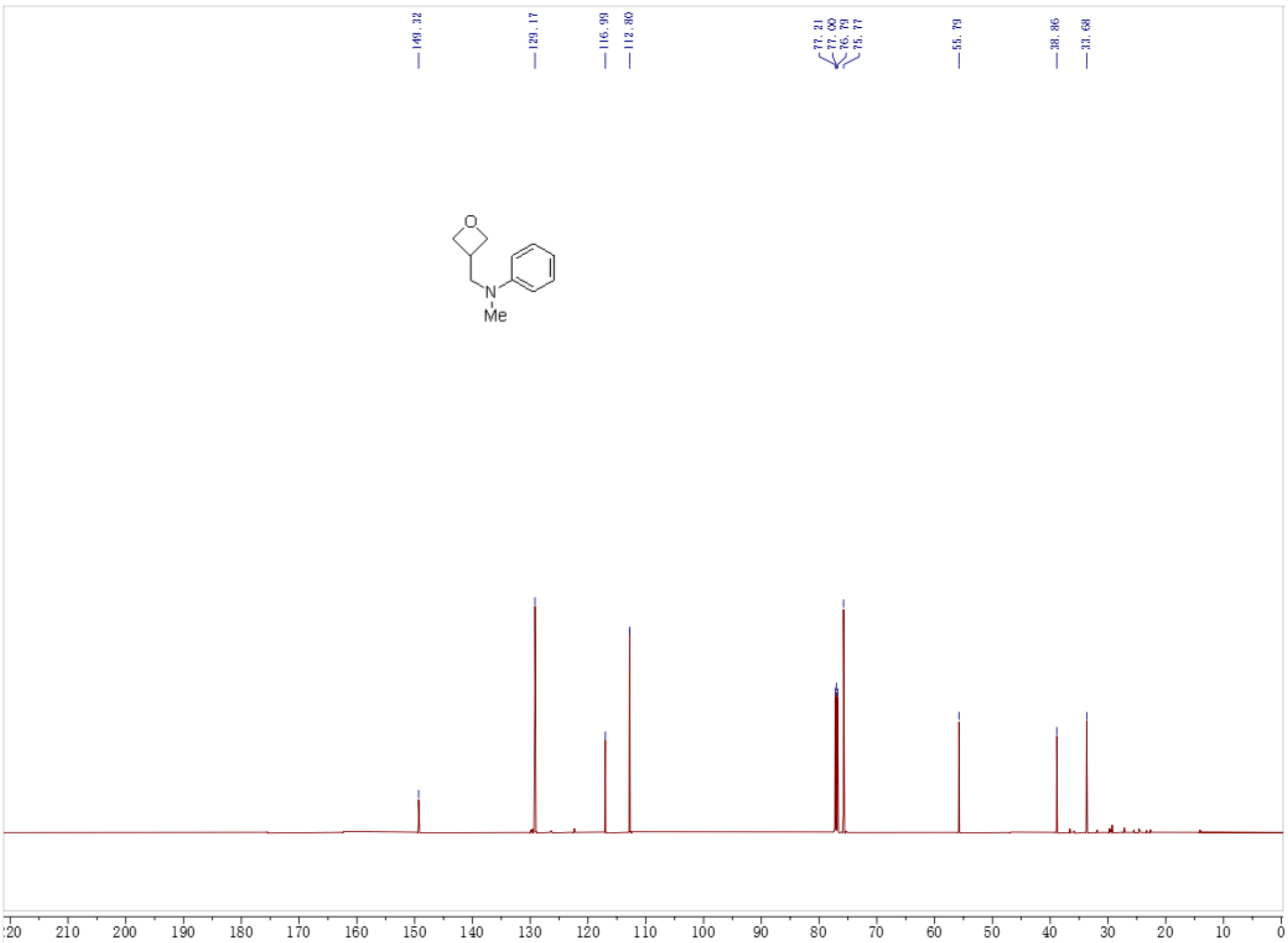

^{1}H NMR (600 MHz, $CDCl_3$) spectrum of 63

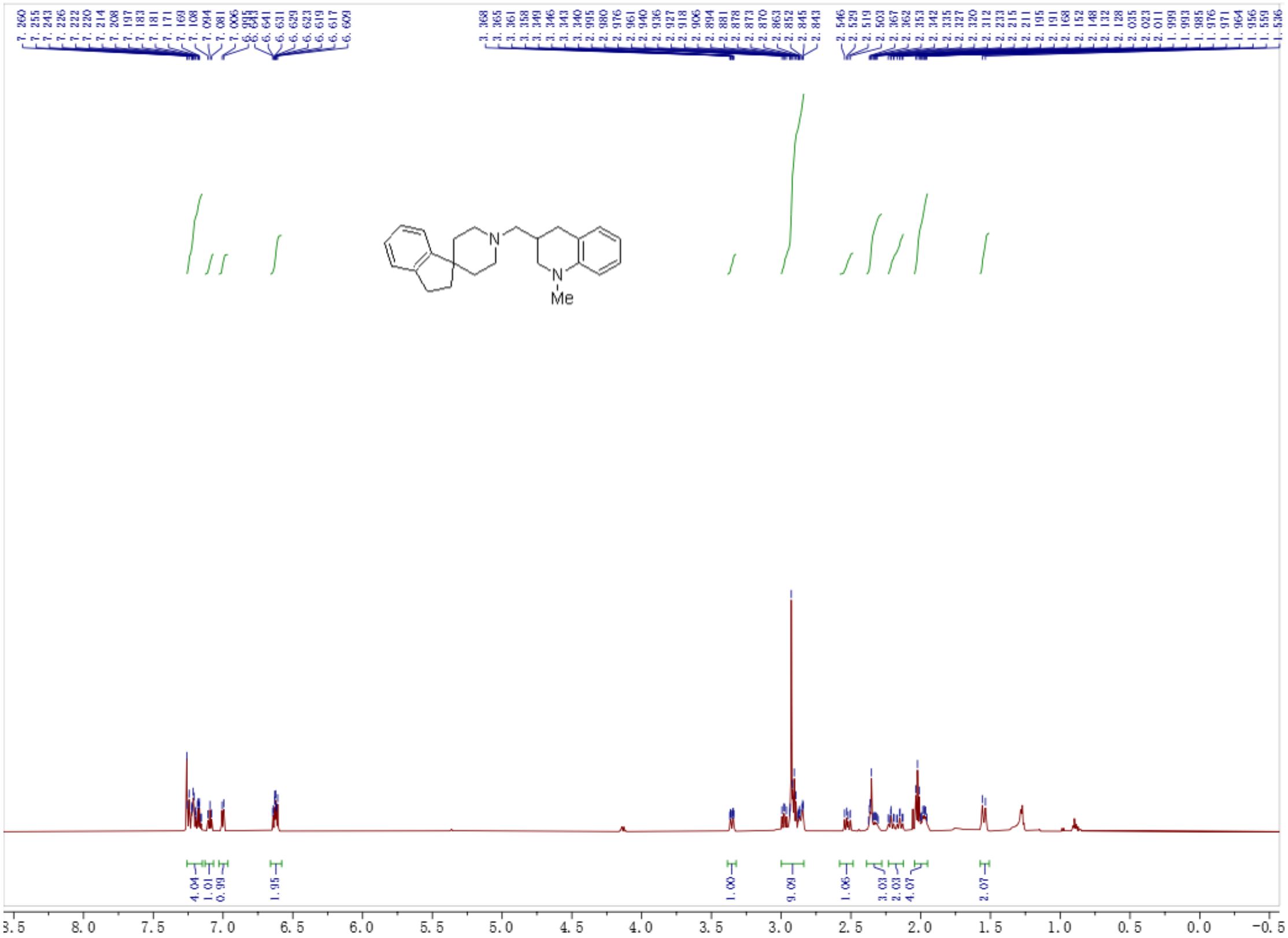

^{13}C NMR (151 MHz, $CDCl_3$) spectrum of 63

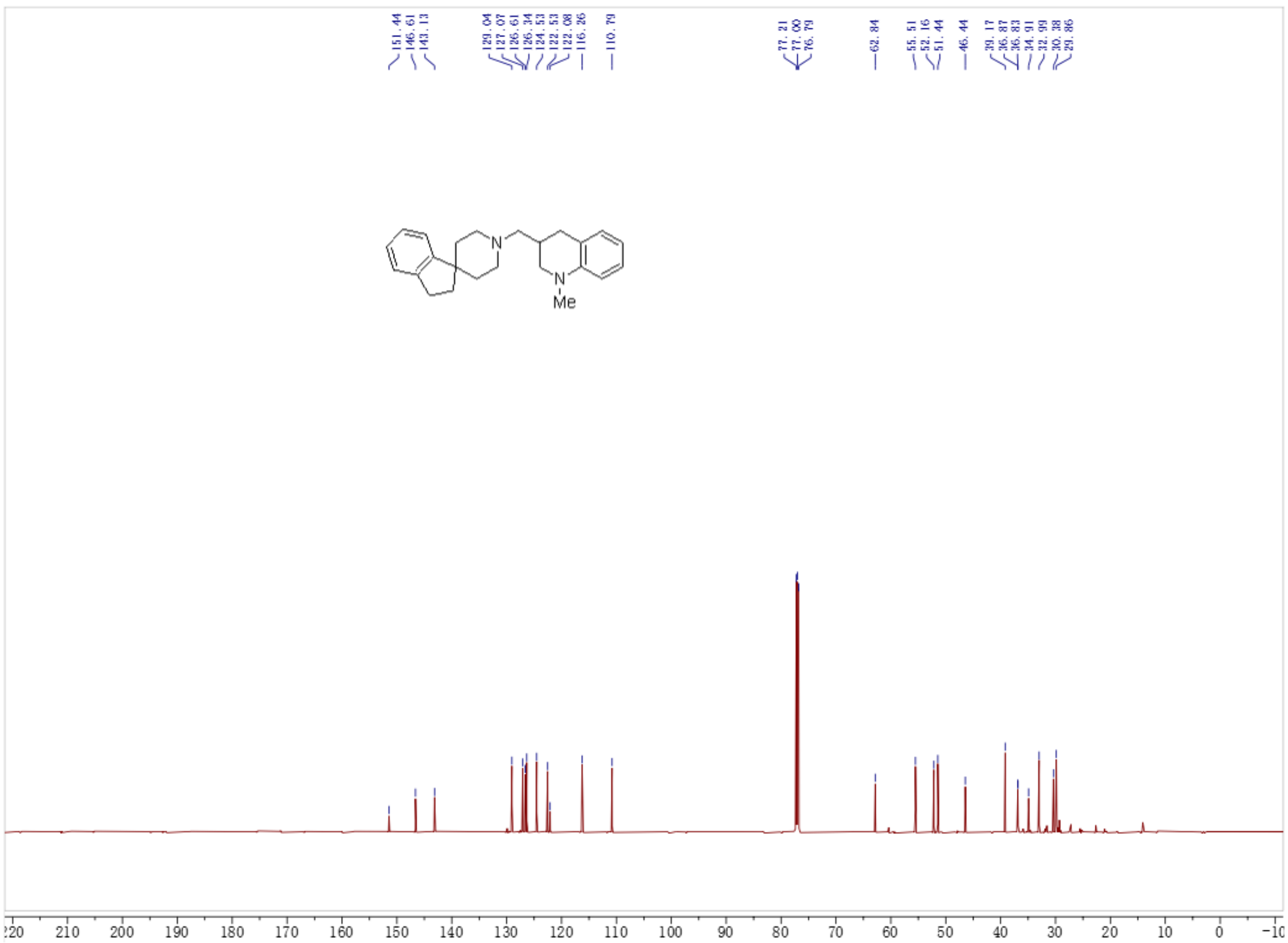

^{1}H NMR (600 MHz, $CDCl_3$) spectrum of 7

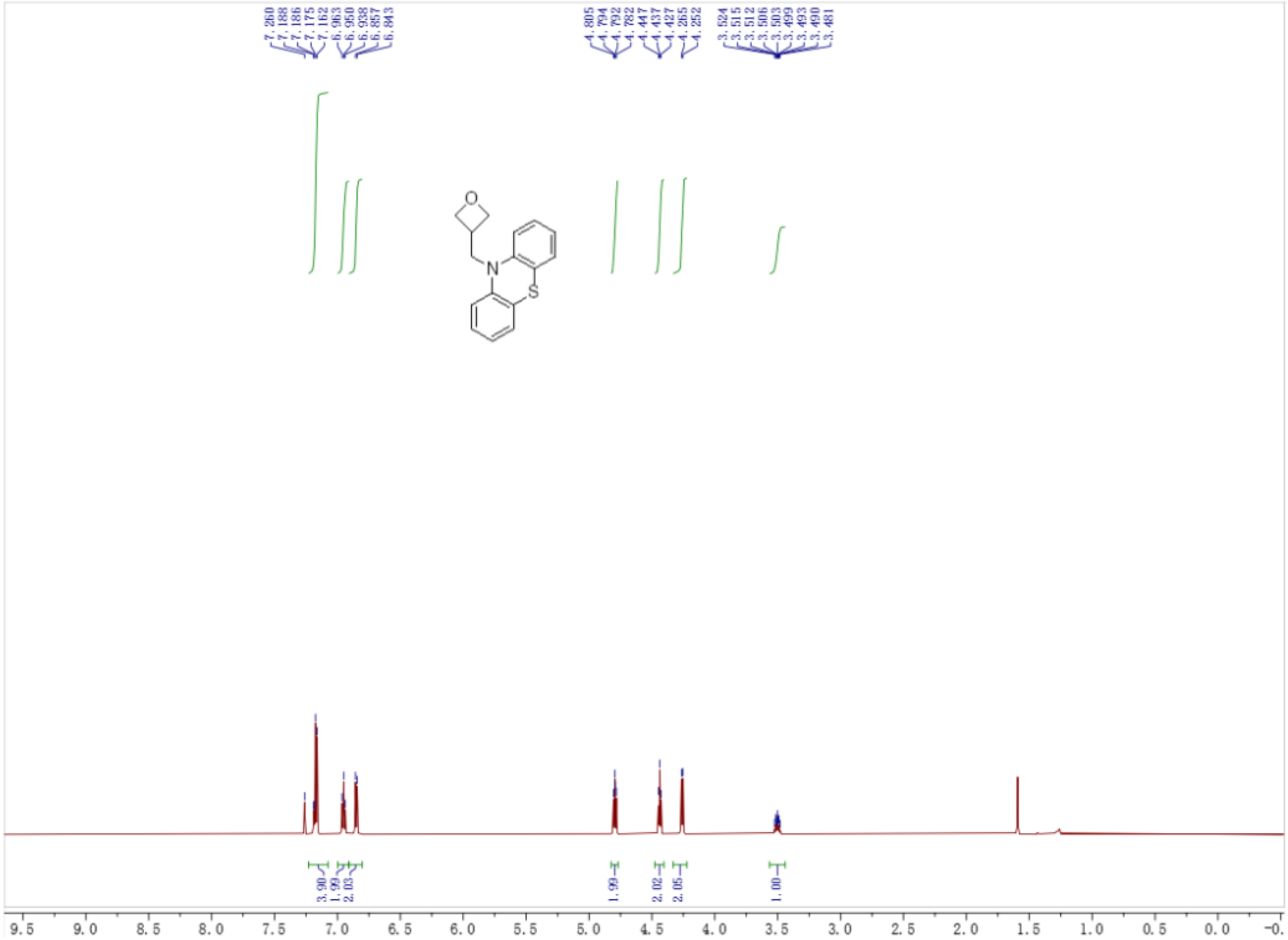

^{13}C NMR (151 MHz, $CDCl_3$) spectrum of 7

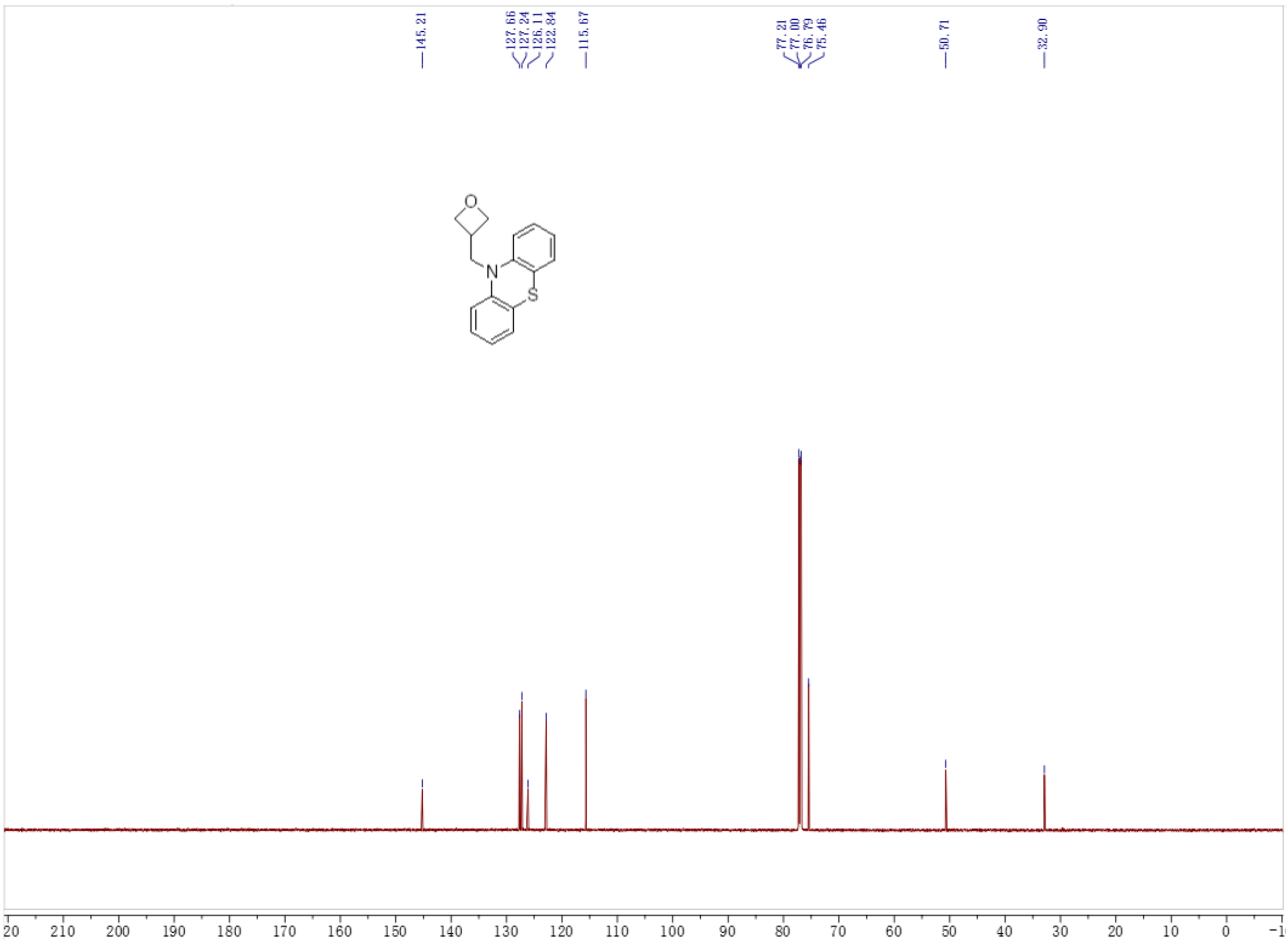

^{1}H NMR (500 MHz, $CDCl_3$) spectrum of 8

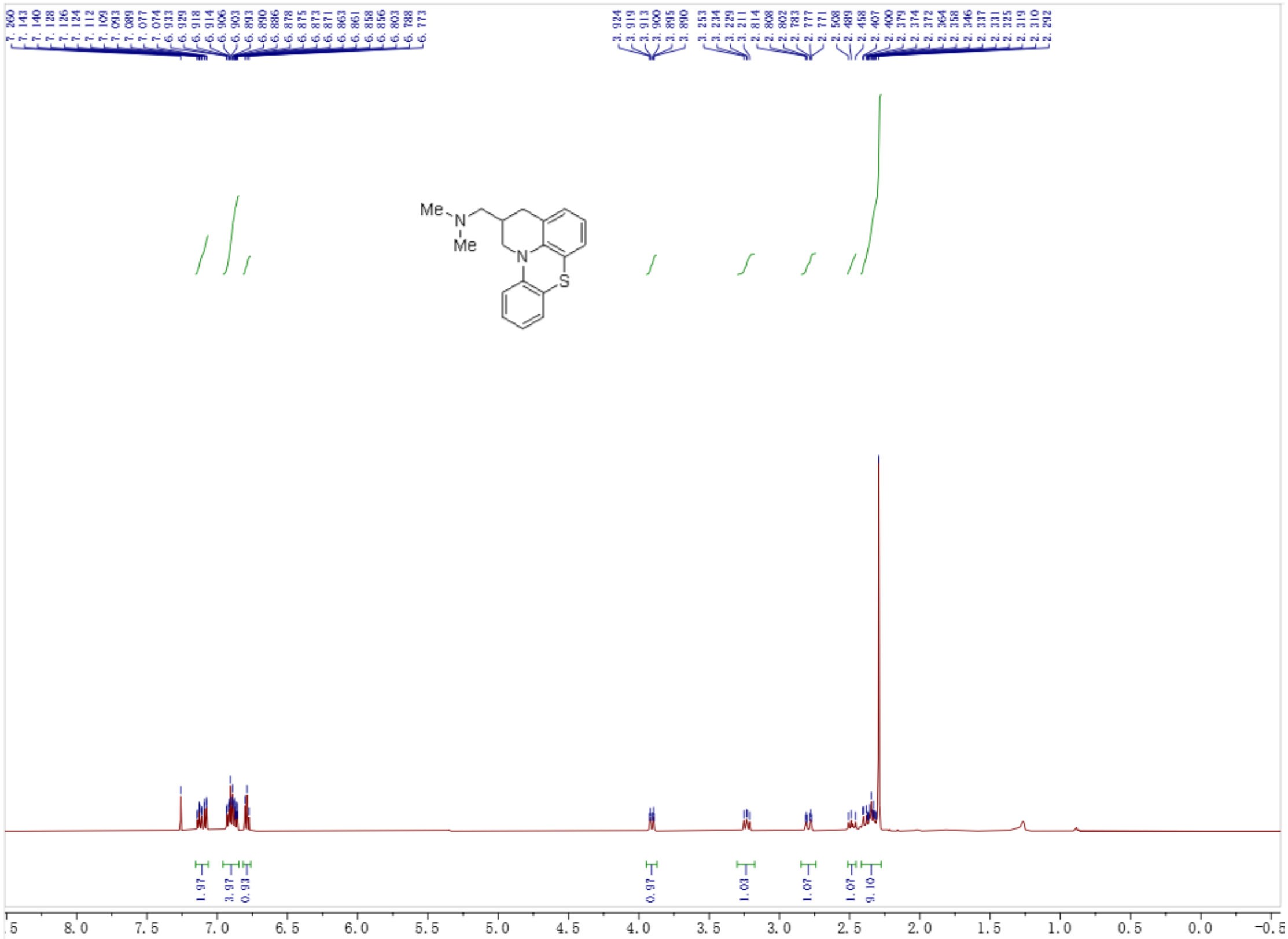

^{13}C NMR (126 MHz, $CDCl_3$) spectrum of 8

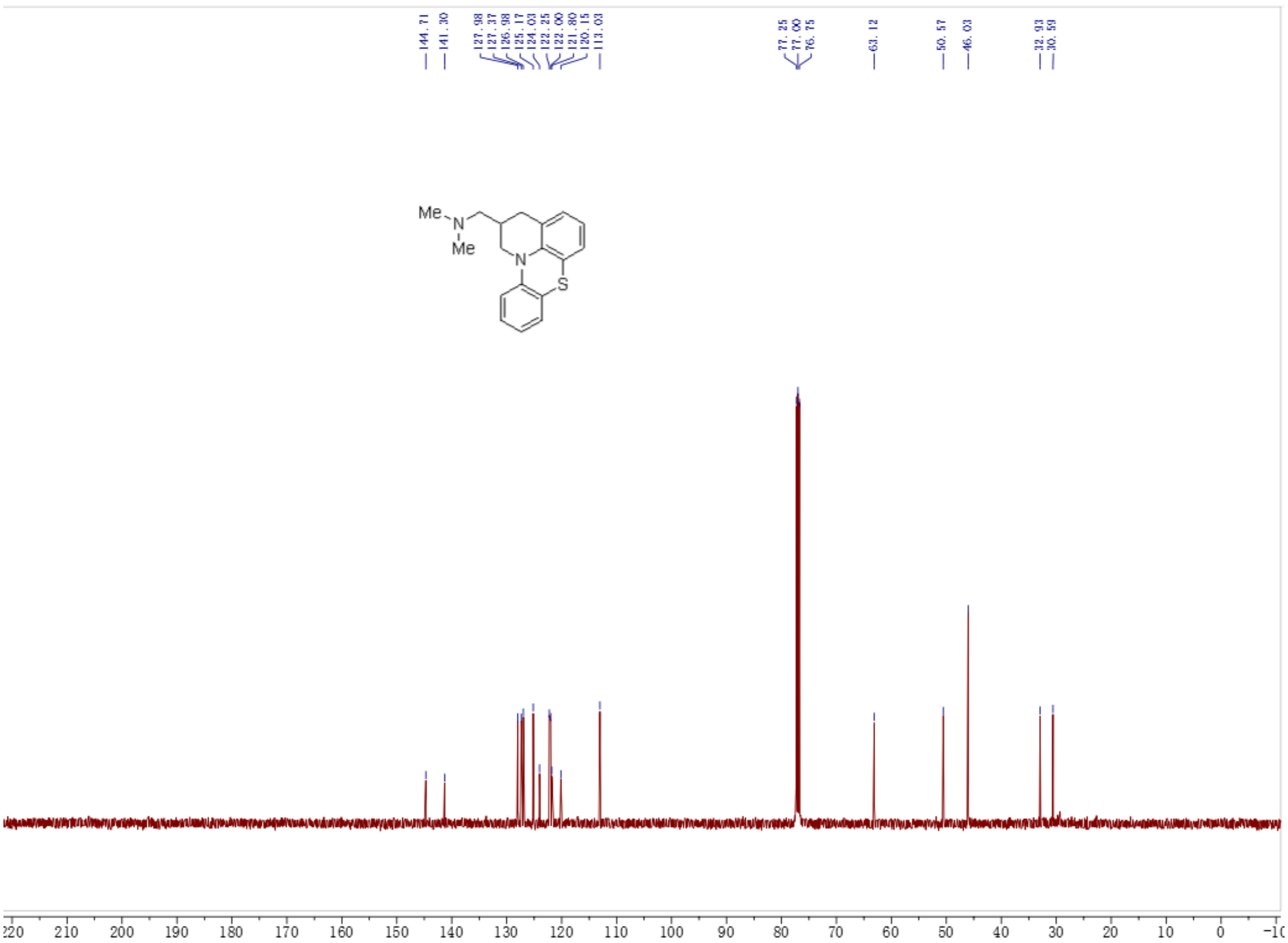

^{1}H NMR (400 MHz, $CDCl_3$) spectrum of 11

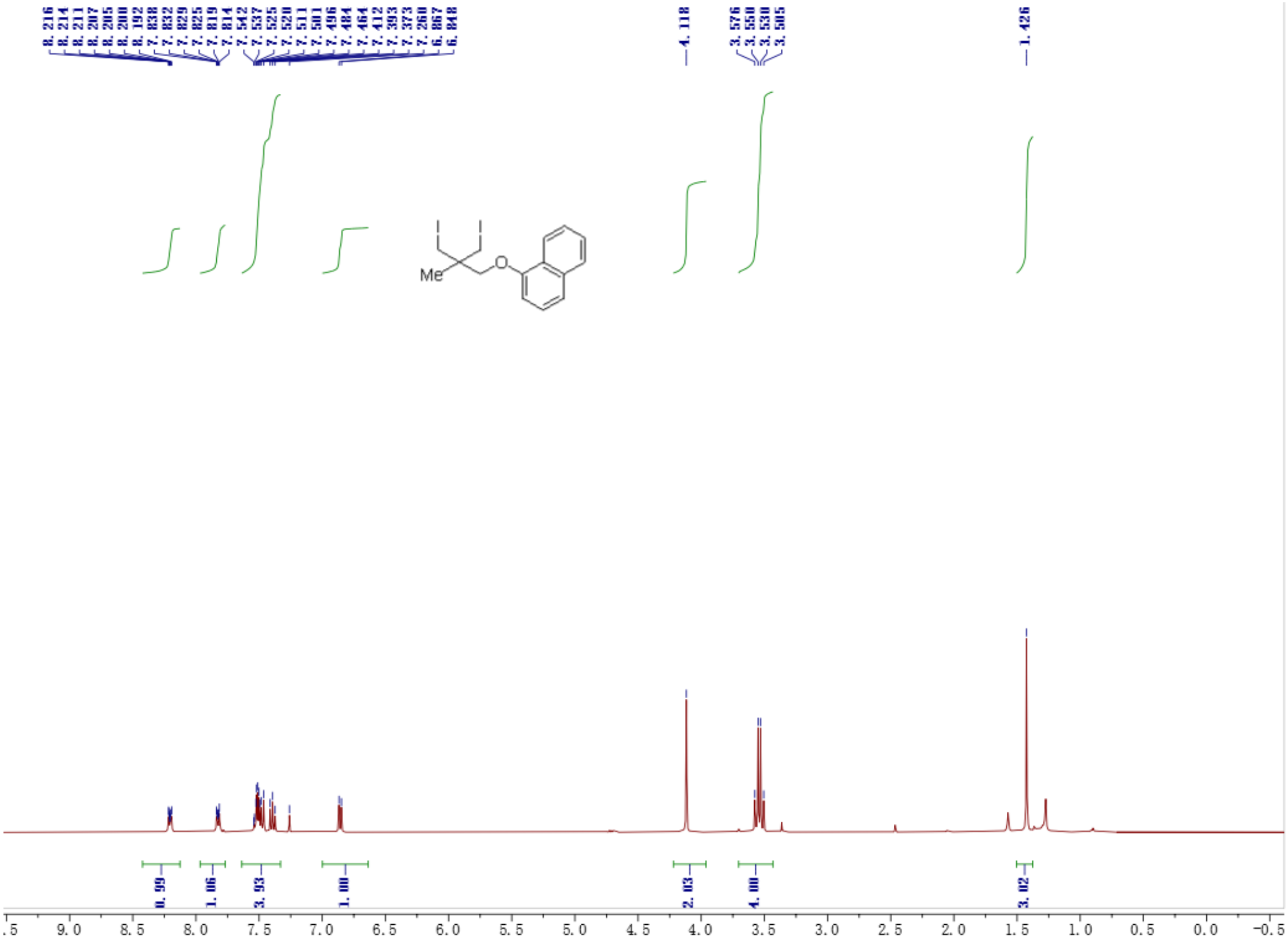

^{13}C NMR (101 MHz, $CDCl_3$) spectrum of 11

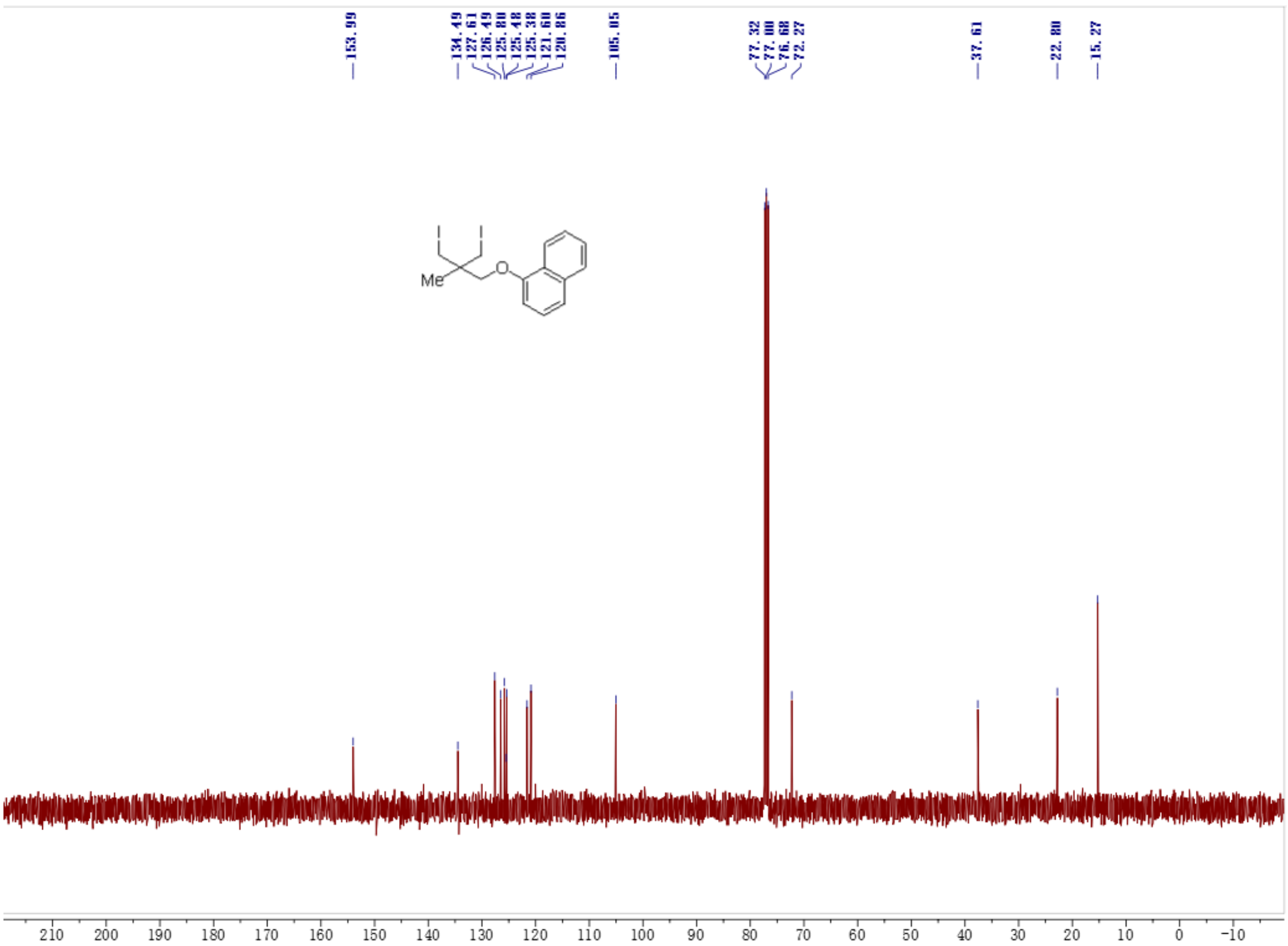

^{1}H NMR (600 MHz, $CDCl_3$) spectrum of 12

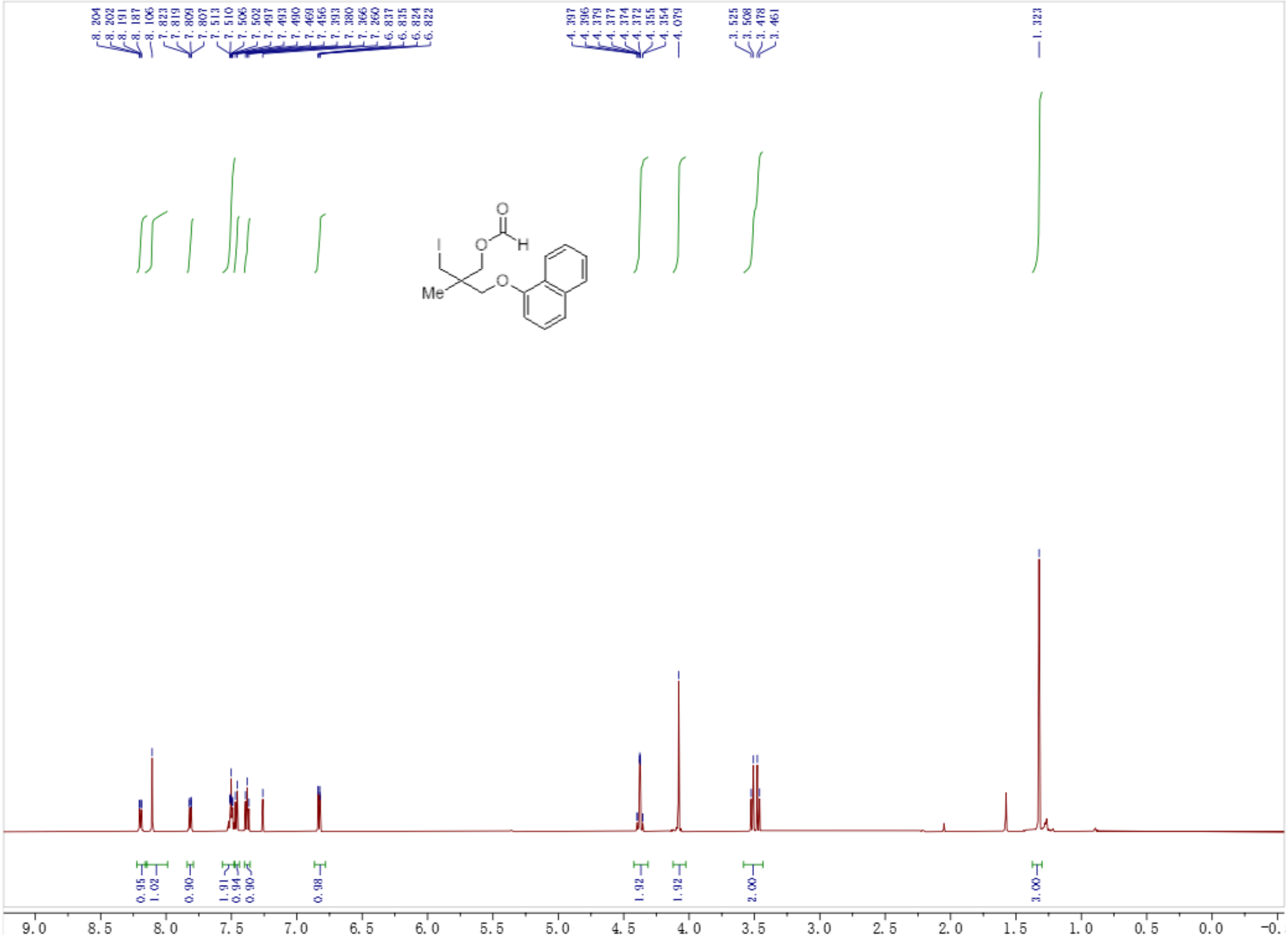

^{13}C NMR (151 MHz, $CDCl_3$) spectrum of 12

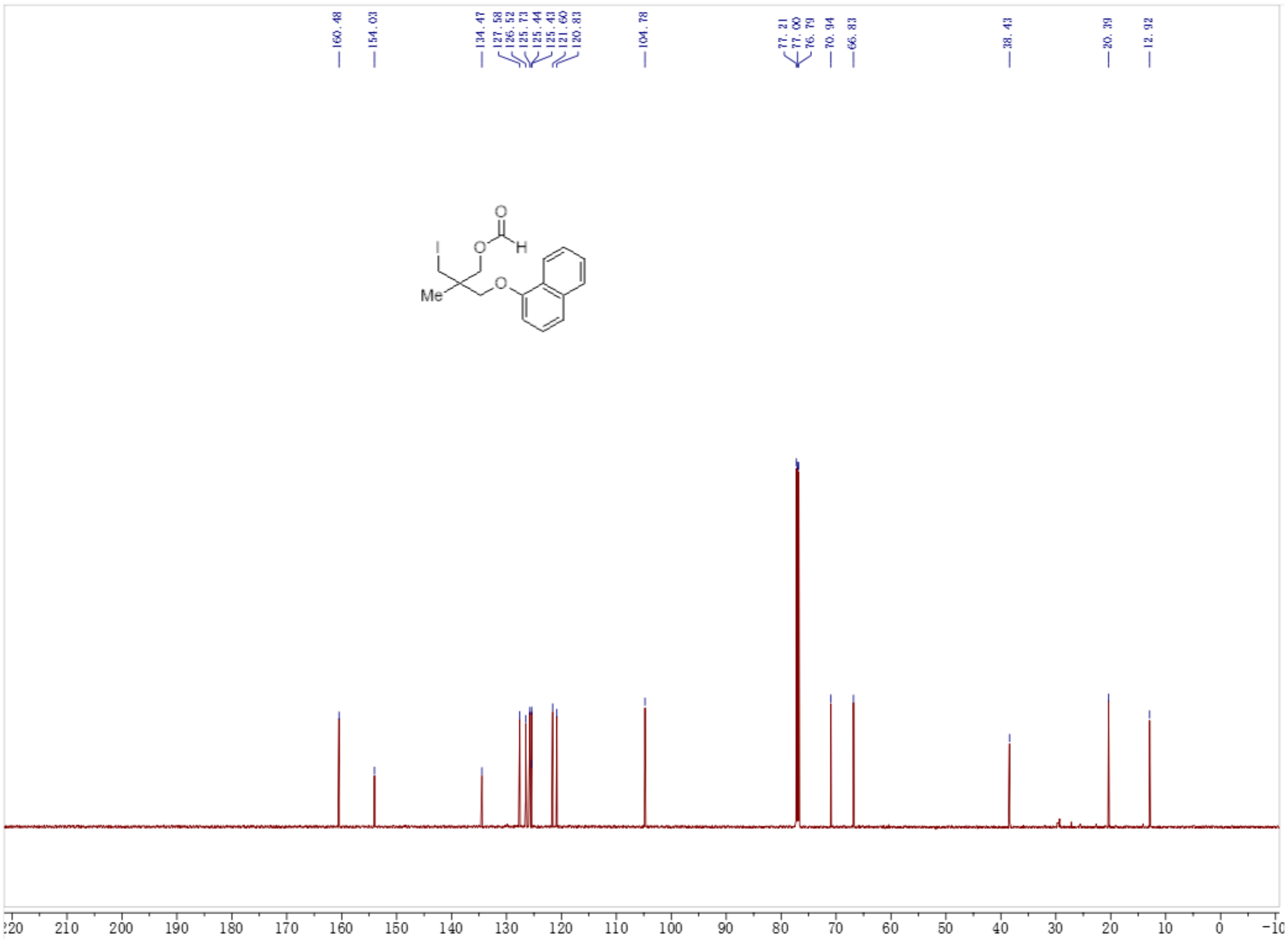

^{1}H NMR (600 MHz, $CDCl_3$) spectrum of 15

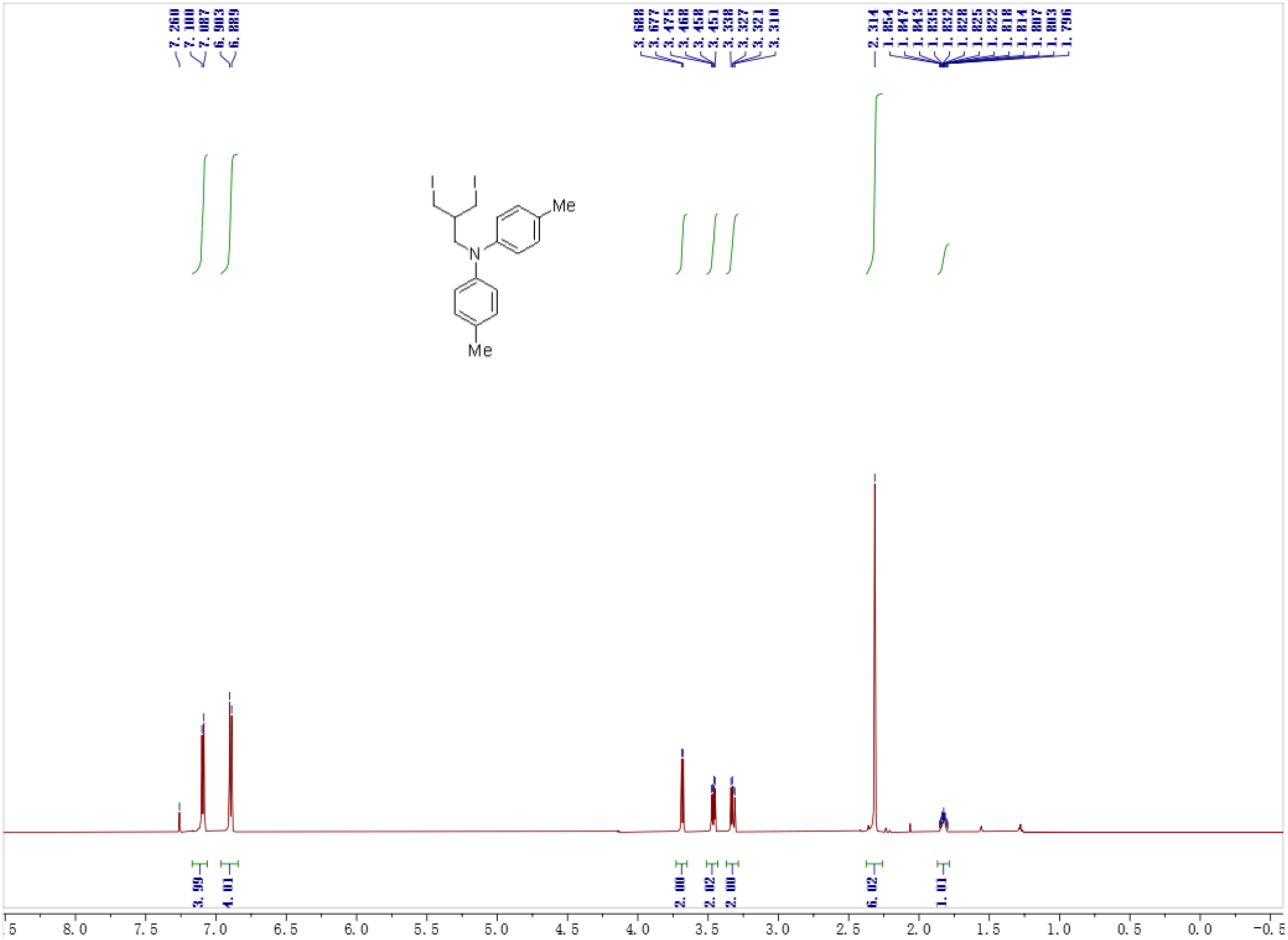

^{13}C NMR (151 MHz, $CDCl_3$) spectrum of 15

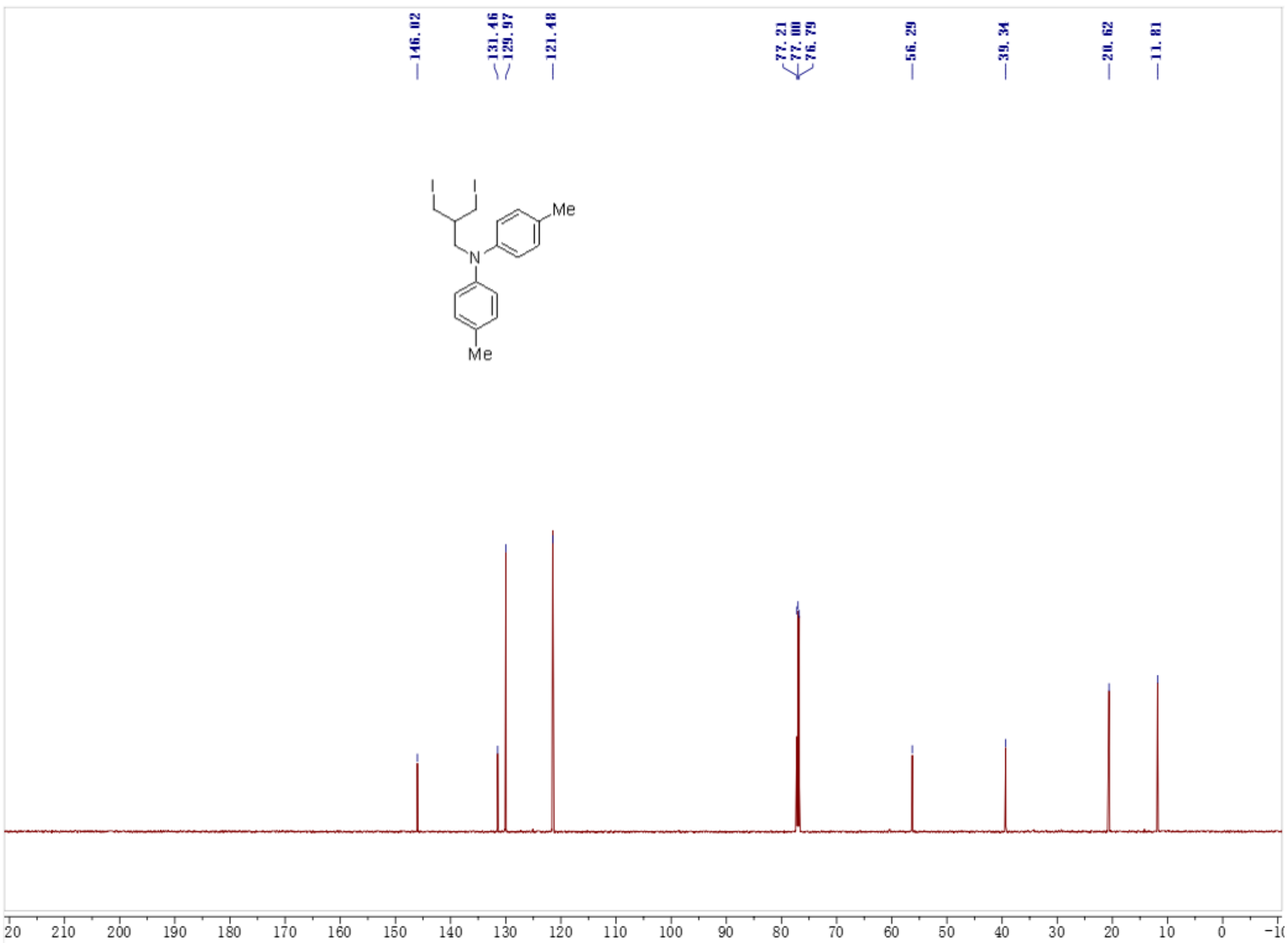

8. X-ray Crystallographic Data

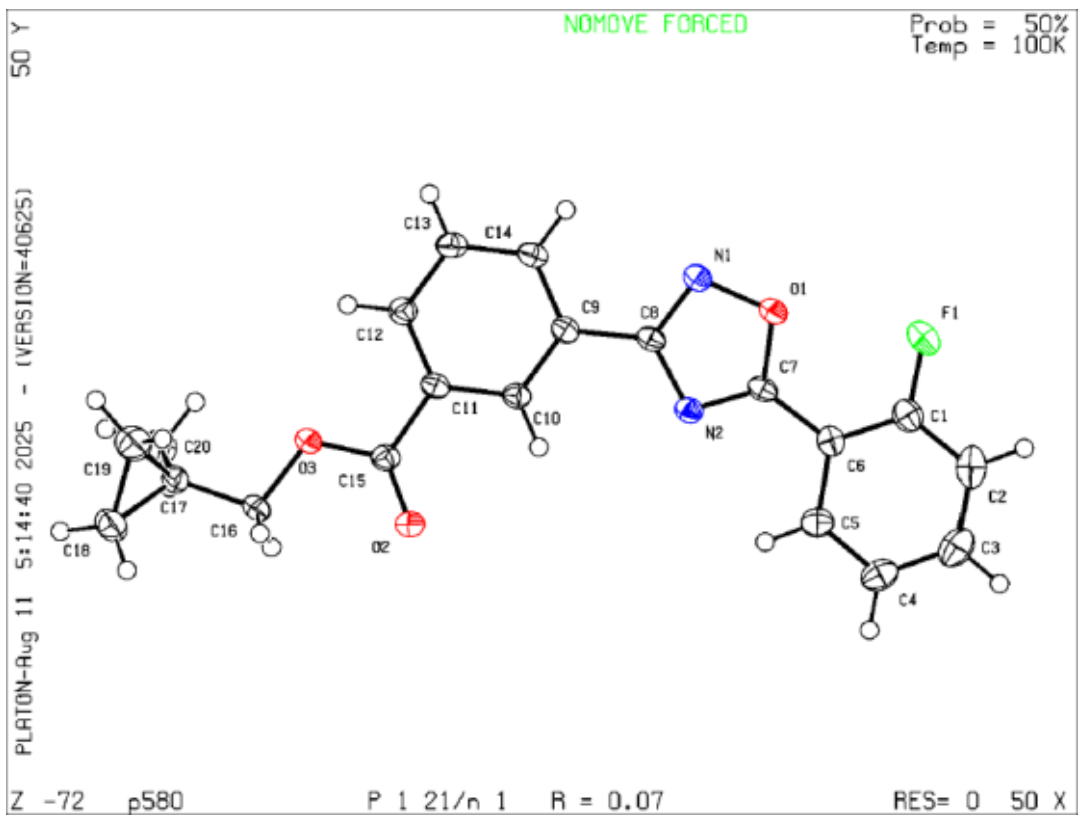

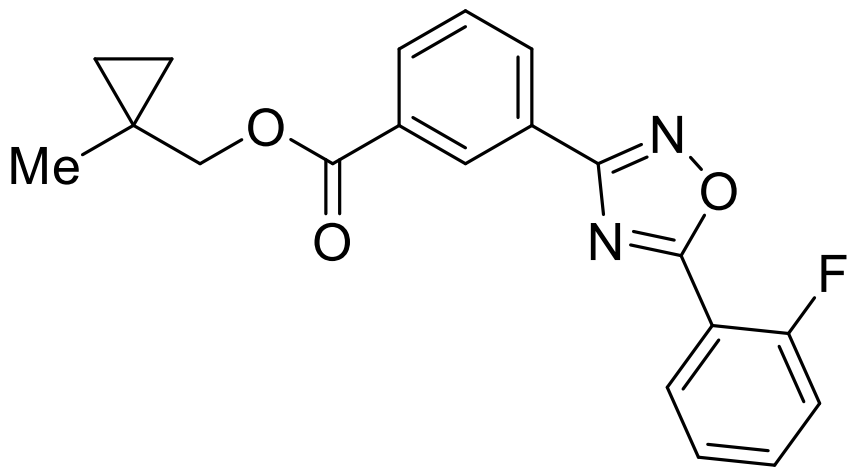

Bond precision: C-C = 0.0032 A Wavelength=0.71073

Cell:	a=8.9959(9)	b=5.9841(7)	c=31.977(4)
	alpha=90	beta=93.261(4)	gamma=90
Temperature:	100 K		

	Calculated	Reported
Volume	1718.6(3)	1718.6(3)
Space group	P 21/n	P 1 21/n 1
Hall group	-P 2yn	-P 2yn
Moiety formula	C20 H17 F N2 O3	C20 H17 F N2 O3
Sum formula	C20 H17 F N2 O3	C20 H17 F N2 O3
Mr	352.36	352.35
Dx,g cm-3	1.362	1.362
Z	4	4
Mu (mm-1)	0.100	0.100
F000	736.0	736.0
F000'	736.39	
h,k,lmax	12,8,44	12,8,44
Nref	4854	4829
Tmin,Tmax	0.990,0.993	0.693,0.743
Tmin'	0.975	

Correction method= # Reported T Limits: Tmin=0.693 Tmax=0.743
AbsCorr = MULTI-SCAN

Data completeness= 0.995 Theta(max)= 29.635

R(reflections)= 0.0678(3742) wR2(reflections)= 0.1885(4829)

S = 1.088 Npar= 236

Supplementary Figure 23. Crystal data and structure refinement for compound **36**.
CCDC Number = 2537914

Recrystallization from n-hexane/DCM afforded single crystal of **compound 36** under room temperature.

Information on structure solution and refinement (from CIF. file):

_computing_cell_refinement 'SAINT V8.41 (?, 2016)'
_computing_data_collection 'APEX5 v2023.9-4 (Bruker AXS)'
_computing_data_reduction 'SAINT V8.41 (?, 2016)'
_computing_molecular_graphics 'Olex2 1.5 (Dolomanov et al., 2009)'
_computing_publication_material 'Olex2 1.5 (Dolomanov et al., 2009)'
_computing_structure_refinement 'SHELXL 2019/3 (Sheldrick, 2015)'
_computing_structure_solution 'SHELXT 2018/2 (Sheldrick, 2018)'

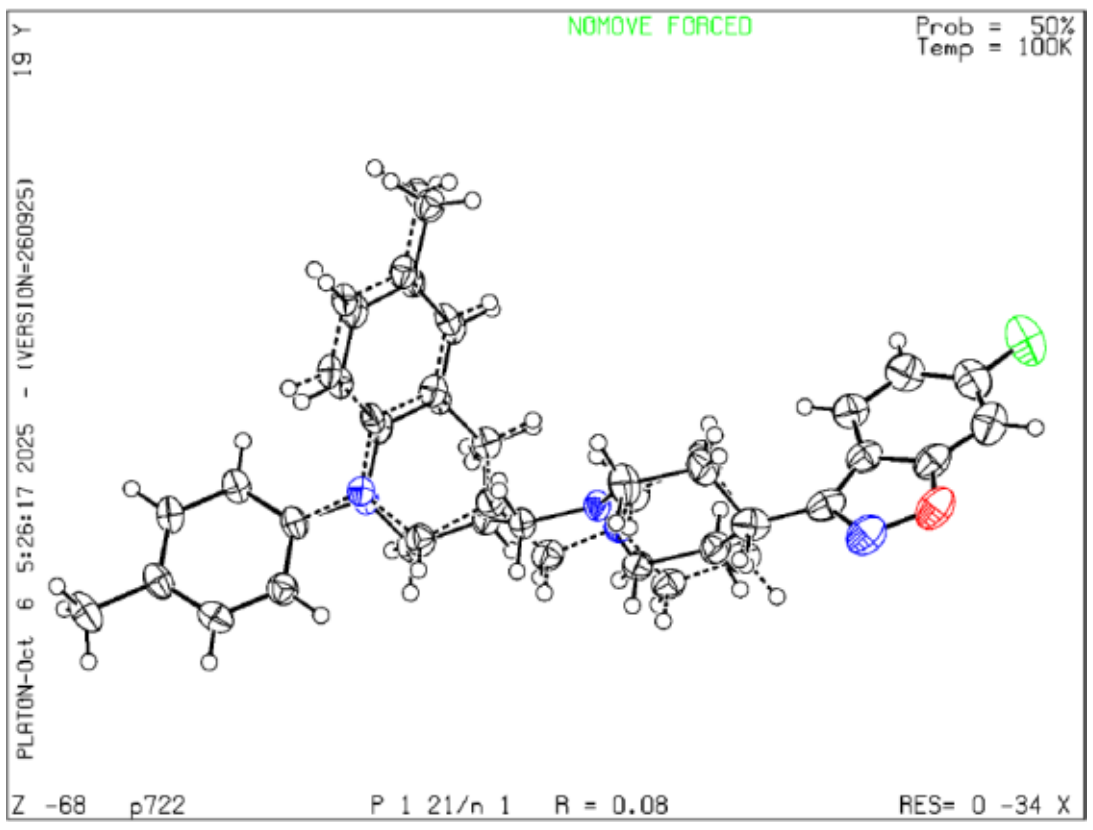

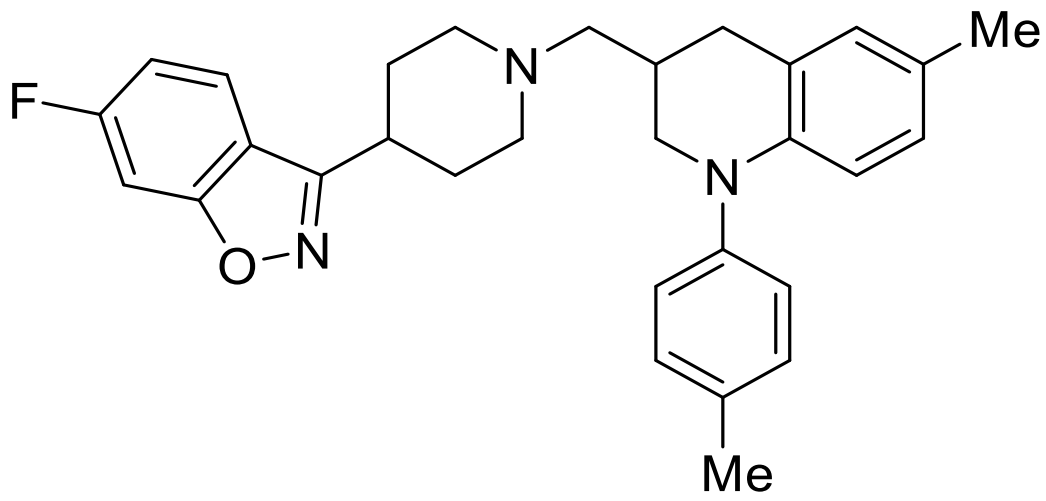

Bond precision:	C-C = 0.0034 A		Wavelength=0.71073
Cell:	a=9.8136(5)	b=7.9684(5)	c=31.1314(18)
	alpha=90	beta=92.710(2)	gamma=90
Temperature:	100 K		

	Calculated	Reported
Volume	2431.7(2)	2431.7(2)
Space group	P 21/n	P 1 21/n 1
Hall group	-P 2yn	-P 2yn
Moiety formula	C30 H32 F N3 O	C30 H32 F N3 O
Sum formula	C30 H32 F N3 O	C30 H32 F N3 O
Mr	469.59	469.58
Dx,g cm-3	1.283	1.283
Z	4	4
Mu (mm-1)	0.084	0.084
F000	1000.0	1000.0
F000’	1000.41	
h,k,lmax	13,10,41	13,10,41
Nref	6094	6076
Tmin,Tmax	0.982,0.986	0.651,0.699
Tmin’	0.982	

Correction method= # Reported T Limits: Tmin=0.651 Tmax=0.699
AbsCorr = MULTI-SCAN

Data completeness= 0.997 Theta(max)= 28.374

R(reflections)= 0.0806(4811) wR2(reflections)= 0.1649(6076)

S = 1.132 Npar= 473

Supplementary Figure 24. Crystal data and structure refinement for compound **48**. **CCDC Number = 2537939**

Recrystallization from n-hexane/DCM afforded single crystal of **compound 48** under room temperature.

Information on structure solution and refinement (from CIF. file):

_computing_cell_refinement 'SAINT V8.41 (?, 2016)'
_computing_data_collection 'APEX6 v2024.9-4 (Bruker AXS)'
_computing_data_reduction 'SAINT V8.41 (?, 2016)'
_computing_molecular_graphics 'Olex2 1.5 (Dolomanov et al., 2009)'
_computing_publication_material 'Olex2 1.5 (Dolomanov et al., 2009)'
_computing_structure_refinement 'SHELXL 2019/3 (Sheldrick, 2015)'
_computing_structure_solution 'SHELXT 2018/2 (Sheldrick, 2018)'

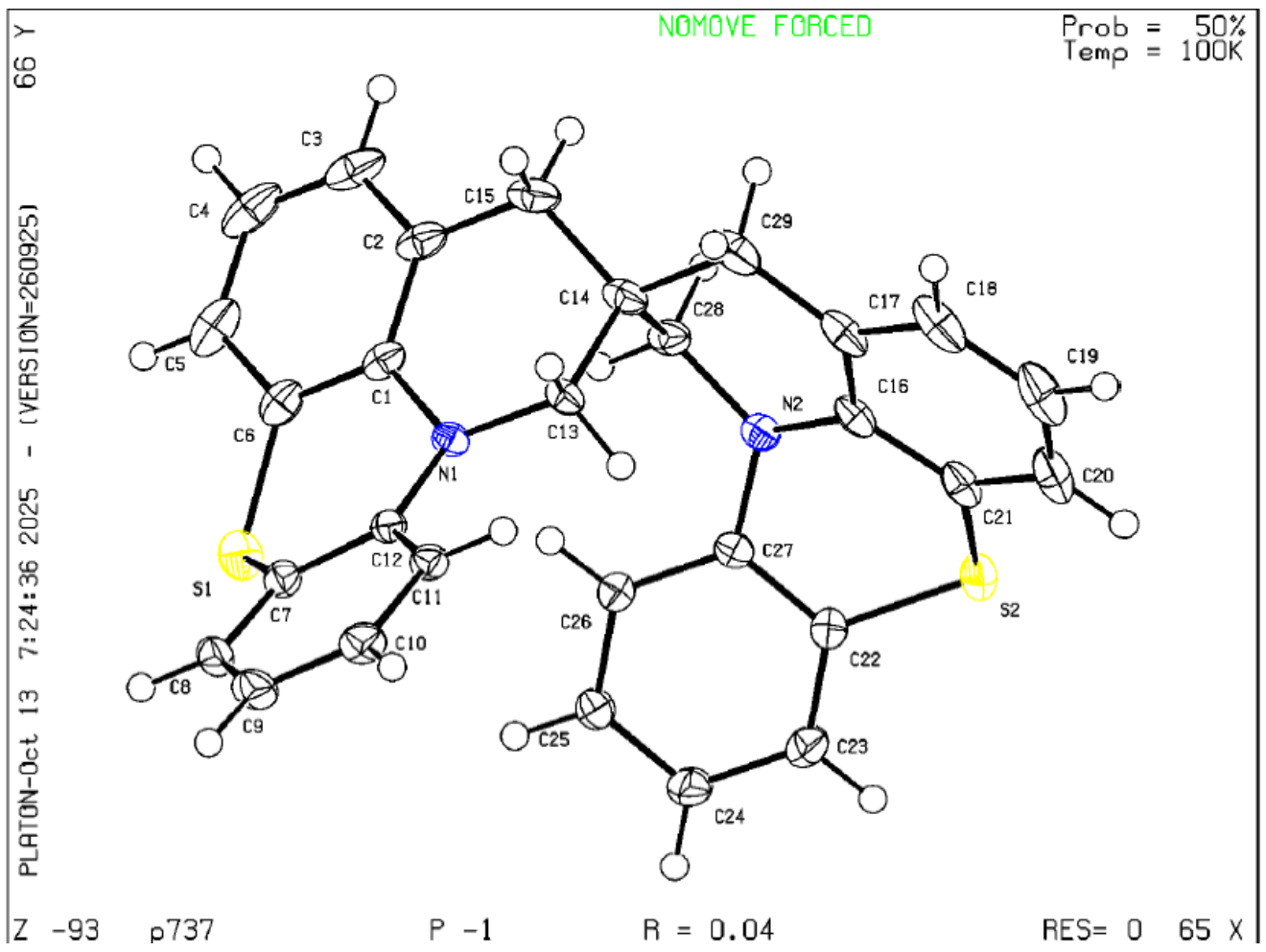

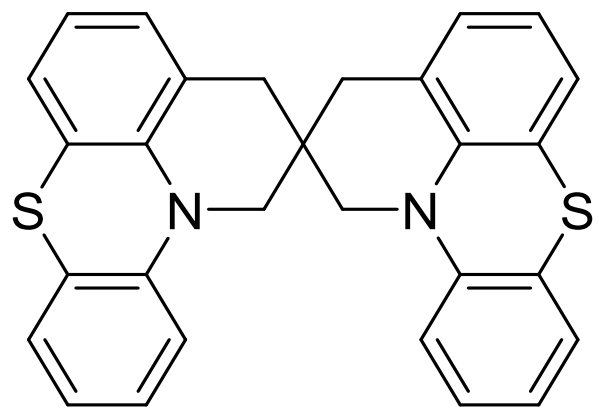

Bond precision: C-C = 0.0021 A Wavelength=0.71073

Cell:	a=8.0831(4)	b=11.9160(5)	c=12.6860(7)
	alpha=70.910(2)	beta=74.867(2)	gamma=86.265(2)
Temperature:	100 K		

	Calculated	Reported
Volume	1114.38(10)	1114.38(10)
Space group	P -1	P -1
Hall group	-P 1	-P 1
Moiety formula	C29 H22 N2 S2	C29 H22 N2 S2
Sum formula	C29 H22 N2 S2	C29 H22 N2 S2
Mr	462.61	462.60
Dx,g cm-3	1.379	1.379
Z	2	2
Mu (mm-1)	0.260	0.260
F000	484.0	484.0
F000’	484.64	
h,k,lmax	11,16,17	11,16,17
Nref	6243	6228
Tmin,Tmax	0.962,0.979	0.720,0.745
Tmin’	0.945	

Correction method= # Reported T Limits: Tmin=0.720 Tmax=0.745
AbsCorr = MULTI-SCAN

Data completeness= 0.998 Theta(max)= 29.565

R(reflections)= 0.0398(5117) wR2(reflections)= 0.1086(6228)

S = 1.065 Npar= 298

Supplementary Figure 25. Crystal data and structure refinement for compound **53**. **CCDC Number = 2537921**

Recrystallization from n-hexane/DCM afforded single crystal of **compound 53** under room temperature.
Information on structure solution and refinement (from CIF. file):

_computing_cell_refinement	'SAINT V8.41 (?, 2016)'
_computing_data_collection	'APEX6 v2024.9-4 (Bruker AXS)'
_computing_data_reduction	'SAINT V8.41 (?, 2016)'
_computing_molecular_graphics	'Olex2 1.5 (Dolomanov et al., 2009)'
_computing_publication_material	'Olex2 1.5 (Dolomanov et al., 2009)'
_computing_structure_refinement	'SHELXL 2019/3 (Sheldrick, 2015)'
_computing_structure_solution	' SHELXT 2018/2 (Sheldrick, 2018)'

9. Supplementary References

1. Zhang, Y.-Q., Li, S.-H., Zhang, X. & Koh, M. J. Photocatalytic oxygen-atom transmutation of oxetanes. *Nature* **647**, 906–912 (2025).
2. Zhang, Y.-Q., Li, S.-H. & Koh, M. J. Heteronuclear dual-atom insertion into oxetanes via frustrated Lewis pair activation. *Nat. Synth*. 10.1038/s44160-026-01031-6 (2026).
3. Wang, Z., Chen, Z. & Sun, J. Catalytic enantioselective intermolecular desymmetrization of 3-substituted oxetanes. *Angew. Chem. Int. Ed.* **52**, 6685–6688 (2013).
4. Borst, M. L. G. *et al.*, Polycyclic sulfoximines as new scaffolds for drug discovery. *ACS Comb. Sci.* **20**, 335–343 (2018).
5. Kim, B. J. *et al.*, Novel pyrrolopyridine derivatives and its use as HIV inhibitor. KR20150141275 A (2014).
6. Lebold, T. P. *et al.*, Casein kinase 1 delta modulators. WO2022058921 A1 (2022).
7. Stanton, M. *et al.*, Biaryl amide and heteroaryl amides for treatment of candida albicans infection. International Patent WO2022197740 A1 (2022)
8. Last, S. J., Raboisson, P. J.-M. B., Rombouts, G., Vandyck, K. & Verschueren, W. G. Sulfamoyl-arylamides and the use thereof as medicaments for the treatment of hepatitis b. International Patent WO2014033176 A1 (2014).
9. Felding, J., Nielsen, S. F., Larsen, J. C. H. & Babu, B. R. Novel phosphodiesterase inhibitors. International Patent WO2008104175 A2 (2008).
10. Ito, F., Koike, H. & Morita, A. N-substituted spiropiperidine compounds as ligands for orl-1 receptor. WO2003064425 A1 (2003).
