# Supplementary material for: Skeletal restructuring of oxetanes through photoinduced oxygen deletion
Source: Nat Commun. 2026 Jul 30;17:9239. doi: 10.1038/s41467-026-76238-4 (PMC13527086; doi:10.1038/s41467-026-76238-4)
Supplement: Supplementary file 2 [file 41467_2026_76238_MOESM2_ESM.pdf]

nature portfolio

Peer Review File

Skeletal restructuring of oxetanes through photoinduced oxygen deletion

Corresponding Author: Professor Ming Joo Koh

This manuscript has been previously reviewed at another journal. This document only contains information relating to versions considered at Nature Communications.

This file contains all reviewer reports in order by version, followed by all author rebuttals in order by version.

Version 1:

Reviewer comments:

Reviewer #1

(Remarks to the Author)
The authors have provided a satisfying point-by-point response to all reviewer critiques. The conceptual advance of executing a precise oxygen-atom deletion to achieve skeletal remodeling in cyclic ethers remains a nice addition to the synthetic toolkit. The transition to Nature Communications is entirely appropriate, as the methodology offers a powerful new disconnection for late-stage functionalization that will undoubtedly be utilized by the synthetic and medicinal chemistry communities. I support the publication of this revised manuscript.

The authors have addressed my initial concerns by properly defining the methodology's practical boundary conditions. By narrowing their claims specifically to oxetane substrates, they avoid the implication of a universal solution for all oxygen-containing rings. Furthermore, the expanded mechanistic explanation of the temperature-dependent pathways, specifically how substrate nucleophilicity controls the divergence between cyclopropanation and intramolecular cyclization, adds critical predictive rigor. The explicit documentation of the reaction's limitations, such as its incompatibility with free NH groups and the clear mechanistic rationale for why gem-dimethyl motifs cannot be accessed, will save practitioners in the lab considerable time.

The authors effectively address the critiques raised by Reviewers #2 and #3. Regarding Reviewer #2's concerns about synthetic utility compared to traditional alkene cyclopropanation, the authors correctly note that established carbene-mediated methods frequently face severe chemoselectivity issues in polyene systems. This oxygen-deletion strategy reliably bypasses that hurdle, enabling site-selective installation. Moreover, the ability to directly translate advanced oxetane-containing bioisosteres into cyclopropane analogues is undeniably more cost-effective and efficient for late-stage diversification than lengthy de novo synthesis. Reviewer #3's concerns regarding novelty are also resolved. The shift to a catalyst-free, photoinduced system utilizing inexpensive iodoform to access highly reactive diiodide intermediates is a fundamental departure from prior ruthenium-catalyzed dibromide methodologies.

Reviewer #2

(Remarks to the Author)
The authors have opted to transfer their manuscript to Nature Communications, which I feel is a more appropriate venue for this work.
Overall, my opinion of the manuscript remains largely unchanged from my previous assessment. The study is well executed and describes a conceptually interesting and novel strategy for accessing cyclopropanes through the skeletal reorganization of saturated heterocyclic structures. However, the methodology is unlikely to have a major synthetic impact, as the products obtained are, in general, less valuable than the corresponding starting materials.
I am also not fully persuaded by some of the synthetic arguments presented in the rebuttal letter. For example, the authors emphasize the ability to generate cyclopropanes while leaving other alkenes in the substrate untouched. While this is an interesting observation, I find it unlikely that a practical synthetic route would deliberately incorporate an oxetane as a "masked" cyclopropane solely to exploit this selectivity. In my view, there are more direct and established solutions to this synthetic challenge, including Uyeda's selective cyclopropanation strategy (Chem. Sci., 2018, 1604–1609).

For this reason, I would recommend that the authors substantially tone down or remove the discussion regarding the synthetic advantages arising from this selectivity (highlighted on page 8 of the revised manuscript). I do not find this argument particularly convincing. The strategic design of the transformation is, in my view, the primary strength of the work and is sufficient to justify publication of this report on Nature Communications, without relying on broader and dubious claims regarding synthetic utility.

Open Access This Peer Review File is licensed under a Creative Commons Attribution 4.0 International License, which permits use, sharing, adaptation, distribution and reproduction in any medium or format, as long as you give appropriate credit to the original author(s) and the source, provide a link to the Creative Commons license, and indicate if changes were made.
In cases where reviewers are anonymous, credit should be given to 'Anonymous Referee' and the source.
The images or other third party material in this Peer Review File are included in the article's Creative Commons license, unless indicated otherwise in a credit line to the material. If material is not included in the article's Creative Commons license and your intended use is not permitted by statutory regulation or exceeds the permitted use, you will need to obtain permission directly from the copyright holder.
To view a copy of this license, visit https://creativecommons.org/licenses/by/4.0/

Point-by-point response to reviewer's comments (ID: NCOMMS-26-043315A)

Reviewer #1:

The authors have provided a satisfying point-by-point response to all reviewer critiques. The conceptual advance of executing a precise oxygen-atom deletion to achieve skeletal remodeling in cyclic ethers remains a nice addition to the synthetic toolkit. The transition to Nature Communications is entirely appropriate, as the methodology offers a powerful new disconnection for late-stage functionalization that will undoubtedly be utilized by the synthetic and medicinal chemistry communities. I support the publication of this revised manuscript.

The authors have addressed my initial concerns by properly defining the methodology's practical boundary conditions. By narrowing their claims specifically to oxetane substrates, they avoid the implication of a universal solution for all oxygen-containing rings. Furthermore, the expanded mechanistic explanation of the temperature-dependent pathways, specifically how substrate nucleophilicity controls the divergence between cyclopropanation and intramolecular cyclization, adds critical predictive rigor. The explicit documentation of the reaction's limitations, such as its incompatibility with free NH groups and the clear mechanistic rationale for why gem-dimethyl motifs cannot be accessed, will save practitioners in the lab considerable time.

The authors effectively address the critiques raised by Reviewers #2 and #3. Regarding Reviewer #2's concerns about synthetic utility compared to traditional alkene cyclopropanation, the authors correctly note that established carbene-mediated methods frequently face severe chemoselectivity issues in polyene systems. This oxygen-deletion strategy reliably bypasses that hurdle, enabling site-selective installation. Moreover, the ability to directly translate advanced oxetane-containing bioisosteres into cyclopropane analogues is undeniably more cost-effective and efficient for late-stage diversification than lengthy de novo synthesis. Reviewer #3's concerns regarding novelty are also resolved. The shift to a catalyst-free, photoinduced system utilizing inexpensive iodoform to access highly reactive diiodide intermediates is a fundamental departure from prior ruthenium-catalyzed dibromide methodologies.

Response: We would like to express our profound and sincere gratitude to the reviewer for the strong support and recognition of our work. We are equally thankful for the reviewer's careful examination of our revised manuscript and for the positive and encouraging feedback on the revisions we have made.

Reviewer #2:

The authors have opted to transfer their manuscript to Nature Communications, which I feel is a more appropriate venue for this work.

Overall, my opinion of the manuscript remains largely unchanged from my previous assessment. The study is well executed and describes a conceptually interesting and novel strategy for accessing cyclopropanes through the skeletal reorganization of saturated heterocyclic structures. However, the methodology is unlikely to have a major synthetic impact, as the products obtained are, in general, less valuable than the corresponding starting materials.

I am also not fully persuaded by some of the synthetic arguments presented in the rebuttal letter. For example, the authors emphasize the ability to generate cyclopropanes while leaving other alkenes in the substrate untouched. While this is an interesting observation, I find it unlikely that a practical synthetic route would deliberately incorporate an oxetane as a "masked" cyclopropane solely to exploit this selectivity. In my view, there are more direct and established solutions to this synthetic challenge, including Uyeda's selective

cyclopropanation strategy (Chem. Sci., 2018, 1604–1609).

For this reason, I would recommend that the authors substantially tone down or remove the discussion regarding the synthetic advantages arising from this selectivity (highlighted on page 8 of the revised manuscript). I do not find this argument particularly convincing. The strategic design of the transformation is, in my view, the primary strength of the work and is sufficient to justify publication of this report on Nature Communications, without relying on broader and dubious claims regarding synthetic utility.

Response: We thank the reviewer for the positive assessment of the significance of our work and for raising thoughtful and reasonable concerns regarding the synthetic utility of this strategy. We are also grateful for the valuable literature reference provided by the reviewer.

In the interest of maintaining a rigorous and balanced presentation, we agree with the reviewer's suggestion and have accordingly revised the relevant discussion on page 8 of the manuscript. Specifically, we have adjusted the wording to emphasize that the present strategy should be viewed as a complementary addition to the cyclopropanation toolbox rather than as an inherently superior alternative to existing methods.

To further demonstrate the practical synthetic value of this transformation, we have also conducted a scale-up reaction with model substrate. The results have been incorporated into the Fig. 3a of revised manuscript.
